# Interplay of buried histidine protonation and protein stability in prion misfolding

**DOI:** 10.1038/s41598-017-00954-7

**Published:** 2017-04-13

**Authors:** Anatoly Malevanets, P. Andrew Chong, D. Flemming Hansen, Paul Rizk, Yulong Sun, Hong Lin, Ranjith Muhandiram, Avi Chakrabartty, Lewis E. Kay, Julie D. Forman-Kay, Shoshana J. Wodak

**Affiliations:** 1 Program in Molecular Structure and Function, Hospital for Sick Children, 555 University Ave, Toronto, ON M5G 1A8 Canada; 2 Department of Biochemistry, University of Toronto, Toronto, ON M5S 1A8 Canada; 3 Department of Molecular Genetics, University of Toronto, Toronto, ON M5S 1A8 Canada; 4 Department of Medical Biophysics, University of Toronto, Toronto, ON M5G 2M9 Canada; 5 Department of Chemistry, University of Toronto, Toronto, ON M5S 3H6 Canada; 6 University College London, Division of Biosciences, London, WC1E 6BT UK; 7 VIB Structural Biology Research Center, VUB, Pleinlaan 2, 1050 Brussels, Belgium

## Abstract

Misofolding of mammalian prion proteins (PrP) is believed to be the cause of a group of rare and fatal neurodegenerative diseases. Despite intense scrutiny however, the mechanism of the misfolding reaction remains unclear. We perform nuclear Magnetic Resonance and thermodynamic stability measurements on the C-terminal domains (residues 90–231) of two PrP variants exhibiting different pH-induced susceptibilities to aggregation: the susceptible hamster prion (GHaPrP) and its less susceptible rabbit homolog (RaPrP). The pKa of histidines in these domains are determined from titration experiments, and proton-exchange rates are measured at pH 5 and pH 7. A single buried highly conserved histidine, H187/H186 in GHaPrP/RaPrP, exhibited a markedly down shifted pKa ~5 for both proteins. However, noticeably larger pH-induced shifts in exchange rates occur for GHaPrP versus RaPrP. Analysis of the data indicates that protonation of the buried histidine destabilizes both PrP variants, but produces a more drastic effect in the less stable GHaPrP. This interpretation is supported by urea denaturation experiments performed on both PrP variants at neutral and low pH, and correlates with the difference in disease susceptibility of the two species, as expected from the documented linkage between destabilization of the folded state and formation of misfolded and aggregated species.

## Introduction

Prion diseases are a group of fatal neurodegenerative diseases that include scrapie in sheep, chronic wasting disease (CWD) in deer and elk, bovine spongiform encephalopathy (BSE) in cattle, as well as a host of human diseases^1^. These diseases result from the misfolding and aggregation of the prion protein (PrP). During prion disease the normal cellular form of the protein PrP^C^, converts to a disease-associated, scrapie isoform (PrP^Sc^). It is widely believed that PrP^Sc^ is the infectious agent and causes the conversion of other PrP^C^ molecules into PrP^Sc^ with no nucleic acid component present^1–4^.

PrP^C^ is monomeric, extracellular, and bound to the cell membrane through a glycosylphosphatidylinisotol (GPI) anchor. Its N-terminal region (23–121) is disordered *in vitro* while the C-terminal region (122–231) adopts a globular fold. NMR chemical shift assignments and high-resolution structures of the globular domain have been determined for a number of species^2, 5–14^. The globular domain of all species shows the same overall fold consisting of a small two-stranded antiparallel β-sheet (S1 and S2) and three α-helices (H1, H2, H3), and contains one disulphide bond between C179 and C214 (human numbering).

PrP^Sc^ differs from PrP^C^ in several ways. Preparations of PrP^Sc^ were shown to contain large fibrils^15^ and to exhibit a reduction in overall α-helix content (from 40% to 30%) and a large increase in β-sheet (from 3% to 45%)^16^. Evidence has been accumulating that misfolded PrP oligomers may be the neurotoxic and/or infective form of the protein (see ref. 17 for review). But otherwise no detailed structural information is available for PrP^Sc^.

The PrP unfolding pathway is highly sensitive to solution and sample conditions. At pH between ~5–7 and in low salt conditions, PrP^C^ from several species (including hamster and human) were shown to unfold via a simple two-state mechanism^18–20^. On the other hand, intermediate states of PrP have been identified under low pH, high salt and pressure conditions^19–22^. These intermediate(s) appear to be more highly populated in disease causing mutant forms of the protein (such as F198S, D178N or R208H in human PrP)^1, 23–25^ and destabilization of the folded state was shown to be correlated with higher populations of intermediates^23^. Characterizing the structural and energetic properties of these intermediates, as well as the stability to unfolding, may therefore shed light on the PrP^C^ to PrP^Sc^ conversion mechanism.

Most research has been carried out on the C-terminal residues 90–231 of PrP, as this region forms the protease-resistant core in PrP^Sc^ and is sufficient for infectivity^15^. Hydrogen exchange (HDX) data on human PrP^C^ measured at pH5.2 and 5.5 and at low denaturant concentrations suggest that the majority of the residues within H1, S2, H2, and H3 remain protected in the native cellular form of the protein^26, 27^ with the region adjacent to the disulphide bond forming a “hyper-stable” core that is likely retained upon unfolding. Other studies focus on specific regions of the protein that are particularly sensitive to external perturbations or are implicated in early protein unfolding events. This is the case for the region near S1^22, 26, 28–30^, the H1 helix^31^, or the entire S1-H1-S2 sub-domain^32–36^.

Several *in vitro* and *in vivo* studies have demonstrated that rabbits are much less susceptible to prion disease than are other mammals, including hamsters^37, 38^. Species-specific differences in susceptibility were suggested to originate from differences in sequence, structure and intramolecular interactions present in the folded and intermediate states of PrP of these animals^39, 40^. A correlation was shown to exist between the population of PrP intermediates and conversion susceptibility in hamster, mouse, and rabbit PrP^41^. In particular, analysis of the response of the more susceptible hamster protein to both acidic pH and urea has demonstrated higher intermediate populations and greater sensitivity to chemical denaturant than for rabbit PrP, the least susceptible protein.

Here two series of Nuclear Magnetic Resonance (NMR) experiments are carried out to investigate the difference in the pH-induced susceptibility to aggregation of the prion proteins (residues 90–231) from golden hamster (GHaPrP) and rabbit (RaPrP). Titration experiments are performed to measure the pKa of the five His residues in the C-terminal domains of the two PrP variants. Estimates for His pKa values in prion proteins were available from computational analyses^42^ and from chemical shift changes^35^, but have so far not been measured from titration experiments. Proton-exchange rates of amide groups are measured in absence of chemical denaturants for the two PrP variants at pH 5 and pH 7 respectively. Results reveal a single buried and highly conserved His residue (H187 in GHaPrP, and H186 in RaPrP) to have a markedly down shifted pKa ~5 in both PrP variants. On the other hand notable differences are observed in the pH-induced shifts in exchange rates in hamster versus rabbit PrP. Analysis of the exchange data indicates that protonation of the buried His is the primary event that destabilizes both PrP variants, but its effect is more drastic in the hamster protein, owing to its lower stability. This interpretation is supported by urea denaturation experiments carried out on both PrP variants at neutral and low pH, and by the documented linkage between destabilization of the folded state and formation of misfolded and aggregated species. In light of the highly conserved sequence properties and shared structural environments around the buried H186/187 residue across mammalian prion proteins, our findings lead to the proposal that protonation of this buried histidine and its effect on the global stability of PrP, represents a prevailing mechanism that underpins the disease susceptibility of mammalian prions.

## Results

### pKa values for His residues in GHaPrP_90–231_ and RaPrP_90–231_

**Figure 1:**
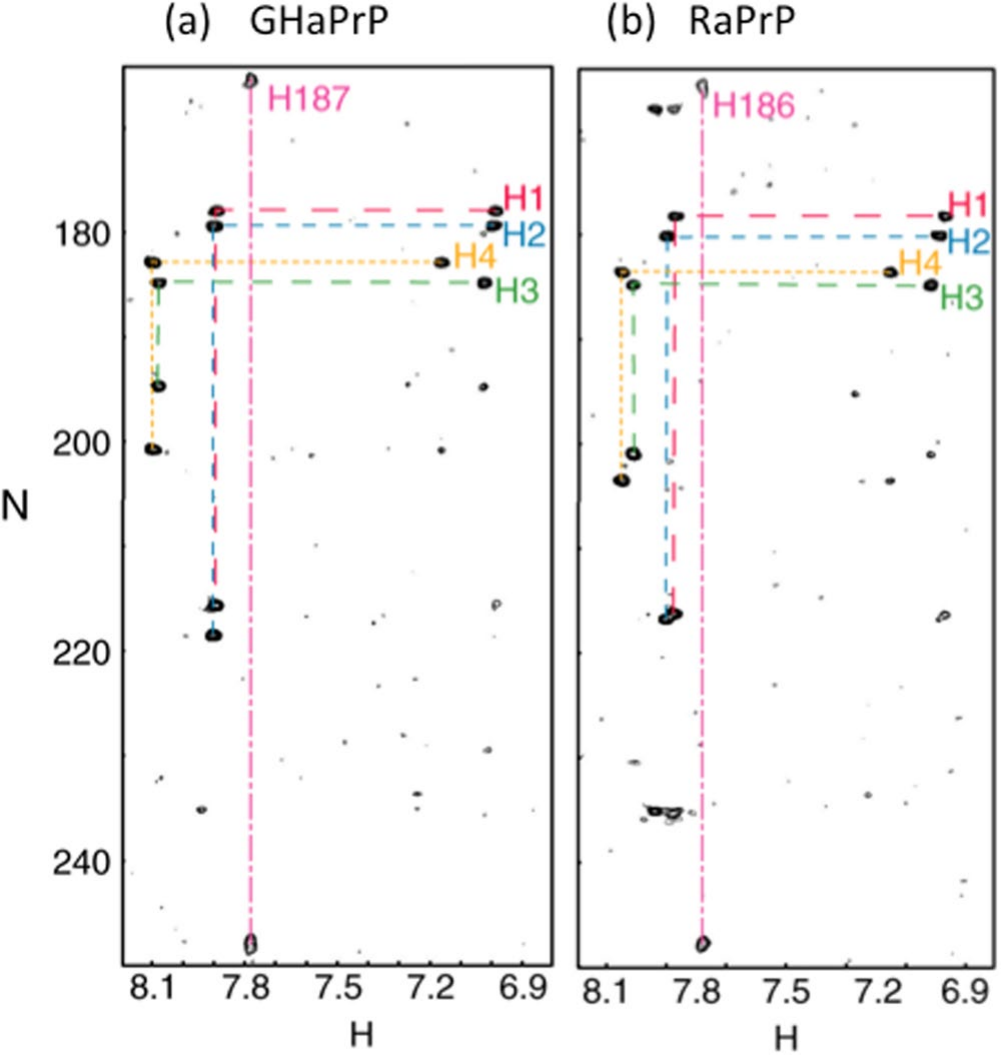
HMBC spectra of the 1 mM hamster (GHaPrP_90–231_) and 1 mM rabbit (RaPrP_90–231_) PrP proteins, recorded at pH 7.1 with resonances from the 5 histidine sidchains identified. (**a**) HMBC spectrum of GHaPrP_90–231_; (**b**) HMBC spectrum for RaPrP_90–231_. Note the similar pattern for the histidines in the spectra of both proteins. For each histidine, except H187 in GHaPrP, and H186 in RaPrP, a peak is observed at the intersection of Nδ1 and Hε1, Nε2 and Hε1 and Nε2 and Hδ2 resonance frequencies. For H187/H186 the Nε2 and Hδ2 peak is missing. H1–H4 correspond to residues H7, H11, H140 and H177 in hamster PrP numbering and have not been assigned ﻿in these experiments.

Since PrP protein stability is heavily influenced by pH and because histidine pKa is in the physiologically relevant range, histidine pKa values for hamster and rabbit PrP were measured by progressively lowering the pH of the samples. HSQC and HMBC experiments were used to observe backbone amide groups and the 5 PrP histidines respectively. The HSQC spectra were generally well dispersed, but showed evidence for progressive loss of amide proton dispersion as the pH dropped. Peak inhomogeneity became more pronounced near to pH 5. All of the 5 histidines, including two in the disordered portion and three in the structured region, were in the neutral ε-tautomeric state^43^ above pH 8 before transitioning to the positively charged state at lower pH (Fig. 1a and b).

Four of the histidine side chain resonances were unassigned in these experiments and described as H1 through H4. One histidine, H187/H186 (hamster/rabbit numbering referred to generally as H187), was assigned as described below. Equivalent histidine sidchains have similar resonance frequencies for hamster and rabbit PrP, allowing us to compare the histidines in both species. Chemical shifts for the Nε2 and Nδ1 and Hε1 and Hδ2 for pHs between 8.5 and 3.5 were obtained from the HMBC spectra and were fit to the Henderson Hasselbalch equation (Fig. 1a,b). Of note, the Nε2 Hδ2 correlation is very weak or not observed for H187 in most spectra, probably resulting from ms-μs timescale motions at this position. As seen in Table 1 (and in the corresponding titration curves in the Supplementary Figure S1), four of the histidines have standard pKa values, but H187/186 has an unusually low pKa of 5.1 ± 0.1 and 4.9 ± 0.1 in the rabbit and hamster proteins respectively. HSQC derived chemical shifts for the amide resonances of residues 185, 181, 156 and 157, which are near to the side chain of H187, were fit to a nearly identical low pKa of 4.9 ± 0.2. The similar titration curves for the H187 side chain and proximal backbone amide resonances allowed assignment of this side chain. The assignment is consistent with published data describing a H186R mutant in mouse PrP with substantially reduced chemical shift changes near residue 186 when transitioning from pH 5.5 to 3.5 when compared to changes observed in the wild type^35^. The nearly identical behavior of rabbit H186 to hamster H187 in terms of line shape, chemical shifts and pKa values, gives confidence in the rabbit H186 assignment. A few amide proton peaks close to the H186 side chain, including Y156, N158 and V188, also titrate with a pKa of 5.1, further confirming this assignment.

**Table 1:** Measured pKa values for His residues of hamster and rabbit PrP.

His residues	Hamster	Rabbit
His1	6.6 ± 0.1	6.7 ± 0.1
His2	6.5 ± 0.1	6.6 ± 0.1
His3	7.1 ± 0.1	7.0 ± 0.1
His4	7.0 ± 0.1	7.0 ± 0.1
His187/186	4.9 ± 0.1	5.1 ± 0.1

Only the side chain of H187/186 was assigned. His1–His4 correspond in an unknown order to residues His96, His111, His140 and His177 in hamster PrP.

Thus, comparing the rabbit and hamster proteins, all 5 histidines have the same pKa values within experimental error. Therefore, the difference in pH-induced susceptibility to aggregation between the rabbit and hamster PrP proteins^37, 38^ is not the direct consequence of differences in these pKa values.

It is however noteworthy that the pKa values obtained for H187/186 are shifted considerably downwards (by ~1.6 pH units) relative to the value expected for a solvated His side chain (pKa = 6.6 ± 0.1)^44^, suggesting that the imidazole group is embedded in an apolar environment in both proteins. H187/186 protonation is therefore expected to decrease PrP stability due to the increased cost of accommodating a positive charge in this environment^45^. Accessible surface area calculation readily confirms that of the 5 His residues in the two PrP variants only H187/186 is ~80–87% buried, (solvent accessible surface area of ~30–50 Å^2^) (Supplementary Table S12). This is not the case for the other two His residues in the structured domain, which remain accessible to the surrounding solvent; those in the unstructured domains are also expected to be well hydrated.

### Effect of pH on PrP local stability

Proton-deuterium exchange experiments were performed at pH7 and pH5 for hamster and rabbit PrP, respectively (see Methods). The measured exchange rates were used to derive the opening free energy of individual residues ${\rm{\Delta }}{G}_{j}^{op}$, representing the energetic cost of adopting the exchange competent conformation of the corresponding amide proton^46, 47^, and providing an estimate of stability at the residue level.

**Figure 2:**
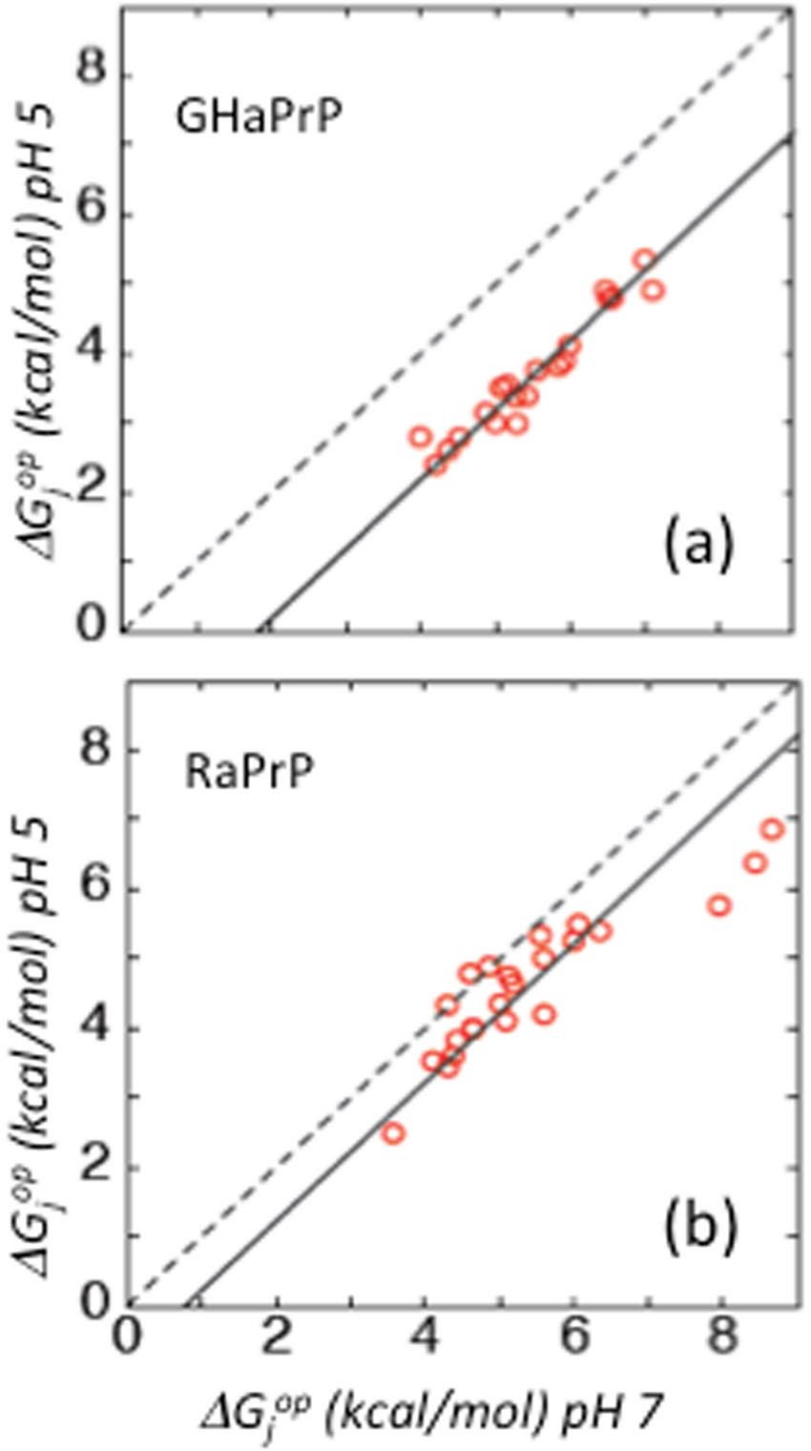
${\rm{\Delta }}{\rm{\Delta }}{G}_{j}^{op}$ plots illustrating residue specific change in ${\rm{\Delta }}{G}_{j}^{op}$, the opening free energy, derived from the measured exchange rates as the pH is reduced from 7 to 5 for the hamster (**a**) and rabbit (**b**) prion proteins. Higher ${\rm{\Delta }}{G}_{j}^{op}\,$ values reflect lower proton exchange rates. The dashed diagonal corresponds to no change in ${\rm{\Delta }}{G}_{j}^{op}\,$ as a function of pH. Solid lines represent the lines of best fit to the ${\rm{\Delta }}{G}_{j}^{op}\,$ values.

Comparison of the ${\rm{\Delta }}{G}_{j}^{op}$ values for hamster and rabbit PrP at the two pH values is illustrated in Fig. 2. Proximity to the diagonal indicates little-to-no change in residue stability (${\rm{\Delta }}{G}_{j}^{op}$) with the change in pH. A linear fit of the GHaPrP data points in Fig. 2a shows that the majority of the values lies along a line below and roughly parallel to the diagonal, indicating a uniform reduction in ${\rm{\Delta }}{G}_{j}^{op}$ throughout the C-terminal domain. In contrast, with a few exceptions, the RaPrP data points are located closer to the diagonal (Fig. 2b), indicating an overall small dependence of ${\rm{\Delta }}{G}_{j}^{op}$ values on pH conditions. However, some variability in the pH dependence of the ${\rm{\Delta }}{G}_{j}^{op}$ values is apparent from the larger spread of data points around the line of best fit.

### The role of His protonation in promoting PrP pH susceptibility

To explain the experimental hydrogen exchange data we developed a model, where the apparent ${\rm{\Delta }}{G}_{j}^{op}\,$ results from a mixture of two sub-states *i* = *1*,*2*, with population level *P*_*i*_. Changes in experimental conditions, such as pH, alter the relative populations of the different sub-states. Assuming that the interconversion between the sub-states occurs on a much faster timescale than the measurable exchange rates, the observed rates are a linear combination of the underlying rates for each sub-state (Equations 9–11 of Methods). To simplify the model, we assume that ${\rm{\Delta }}{G}_{j,i}^{op}$ (the opening free energy of residue *j* in sub-state *i*) changes negligibly with pH, although we realize that this may in general not be the case. Using this simplification, the differences in sub-state population levels *P*_*i*_ are the main parameters affecting the pH-induced difference in exchange rates.

**Figure 3:**
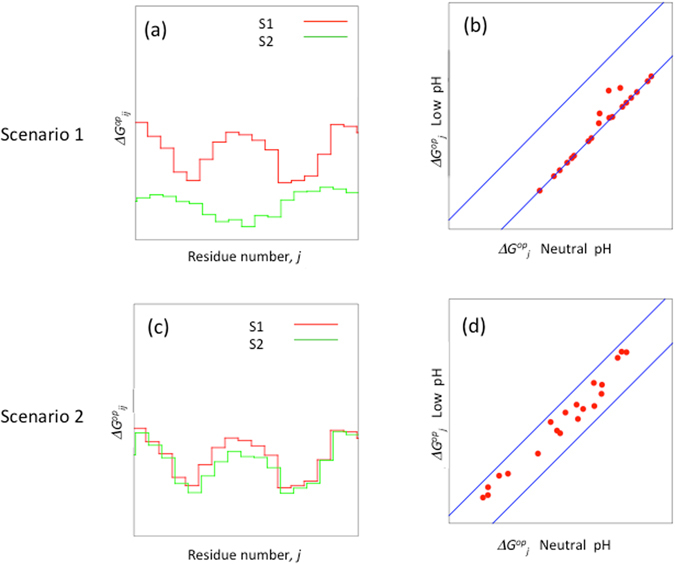
A model of pH induced changes in ${\rm{\Delta }}{G}_{j}^{op}$ values explaining the PrP hydrogen exchange data for two different scenarios in a system comprising two sub-states. Scenario 1 (**a,b**). The two sub-states S_1_ (red) and S_2_ (green) interconvert rapidly, with sub-state S_2_ being less stable than sub-state S_1_ at both neutral and low pH conditions (**a**). Hence only S_2_ contributes to the measured exchange rates and the corresponding ${\rm{\Delta }}{G}_{j}^{op}\,$ values. (**b**) Suppose that lowering the pH increases the population of S_2_, without influencing the individual exchange rates. In this case ${\rm{\Delta }}{G}_{j}^{op}\,$ values are shifted as shown in a correlation plot of residue specific ${\rm{\Delta }}{G}_{j}^{op}\,$ vs pH. This shift depends solely on the ratio of the populations of S_2_ at neutral versus low pH and is represented by the down shifted diagonal (reflecting the increase in S_2_ population at low pH), along which the majority of the ${\rm{\Delta }}{G}_{j}^{op}\,$ values are aligned (shift = −$RTln\frac{{P}_{2}(low\,pH)}{{P}_{2}(pH7)}$). The upper diagonal represents the expected shift in ${\rm{\Delta }}{G}_{j}^{op}\,$ values for the reverse case where hydrogen exchange rates are dominated by state *S*_*1*_ (shift = −$RTln\frac{{P}_{1}(low\,pH)}{{P}_{1}(pH7)}$). Scenario 2 (**c,d**). Suppose that the two sub-states *S*_*1*_ and *S*_*2*_ have similar stabilities, and therefore both contribute to the measured exchange rates and ${\rm{\Delta }}{G}_{j}^{op}\,$ values. ${\rm{\Delta }}{G}_{j}^{op}$ values for sub-states *S*_*1*_ and *S*_*2*_ in this case are shown in (**c**). (**d**) Correlation plots of ${\rm{\Delta }}{G}_{j}^{op}\,$ values at neutral and low pH. The up and down shifted diagonals are as in (**b**).

On the basis of this mixture model, we envisage two scenarios. In the first scenario, the system comprises two rapidly interconverting sub-states denoted *S*_*1*_ and *S*_*2*_. Sub-state *S*_*2*_ is less stable than *S*_*1*_ at both pH 7 and pH 5 and thus features faster exchange rates than *S*_*1*_. As a result, *S*_*2*_ is the only sub-state that contributes to the measured exchange rates and ${\rm{\Delta }}{G}_{j}^{op}$ values under both pH conditions. This is illustrated in Fig. 3a, where with a few exceptions, the ${\rm{\Delta }}{G}_{j,i}^{op}$ values for *S*_*2*_ are systematically lower than those of *S*_*1*_, at both neutral and low pH. Now let us assume that the population of *S*_*2*_ increases whereas that of *S*_*1*_ decreases when the pH is reduced from 7 to 5. Plotting ${\rm{\Delta }}{G}_{j,i}^{op}$ values at pH 5 versus those at pH 7 will then yield a series of points, most of which lie along a single line shifted down from the diagonal by an amount corresponding to the difference in *S*_*2*_ population, as given by the relation ${\rm{\Delta }}{G}_{j,2}^{op}(pH5)-{\rm{\Delta }}{G}_{j,2}^{op}(pH7)=-RTln\frac{{P}_{2}(pH5)}{{P}_{2}(pH7)}$, where *P*_*2*_ is the population of S_2_ (Fig. 3b).

In the second scenario, illustrated in Fig. 3c, the ${\rm{\Delta }}{G}_{i,j}^{op}\,$ values for *S*_*1*_ and *S*_*2*_ display smaller relative differences. Both sub-states therefore contribute to the measured exchange rates, with either *S*_*1*_ or *S*_*2*_ making a dominant contribution for a particular residue depending on which state has a lower ${\rm{\Delta }}{G}_{i,j}^{op}\,$ value at a given residue. Thus, even though the populations of the states (either *S*_*1*_ or *S*_*2*_) change upon reducing the pH, there will be no uniform shift in ${\rm{\Delta }}{G}_{j}^{op}$ values. As a result, a plot comparing the ${\rm{\Delta }}{G}_{j}^{op}\,$ values of individual residues at pH 5 versus those at pH 7 (Fig. 3d **)** will have points scattered both above and below the diagonal.

The changes in apparent residue stabilities, or ${\rm{\Delta }}{\rm{\Delta }}{G}_{j}^{op}$, with pH for GHaPrP and RaPrP, which are displayed in Fig. 2, closely resemble the two scenarios of the multi-state exchange mechanism illustrated in Fig. 3. The ${\rm{\Delta }}{\rm{\Delta }}{G}_{j}^{op}\,$ values shown correspond to residues for which exchange rates could be measured under both pH conditions.

### GHaPrP

The uniform change in ${\rm{\Delta }}{G}_{j}^{op}\,$ values (and the corresponding exchange rates) of GHaPrP (Fig. 2a) follows the behavior described in scenario one (Fig. 3a,b), suggesting that a single PrP sub-state dominates the measured exchange rates at both pH conditions. The increase in exchange rates at low pH implies that it is the protontated sub-state that has the higher exchange rates. Furthermore, the magnitude of the rate increase should match the increase in the population of the protonated form, as illustrated in Fig. 3a,b.

A linear fit to the data points of Fig. 2a yields a line whose intercept with the abscissa corresponds to the shift in ${\rm{\Delta }}{G}_{j}^{op}$ as a function of pH, denoted as ${\rm{\Delta }}{\rm{\Delta }}{G}_{j}^{op}(shift)$. Correcting this intercept for the error associated with the experimental ${\rm{\Delta }}{G}_{j}^{op}\,$ values (see Supplementary Figure S2 for detail) yields the following value:

1 $${\rm{\Delta }}{\rm{\Delta }}{G}_{j}^{op}(Shift)=1.85\pm 0.7\,kcal/mol$$

Next, using the well-known definitions of the pH and *pKa*, we estimate ${\rm{\Delta }}{\rm{\Delta }}{G}_{j}^{op}$ values as a function of pH solely on the basis of the measured His187 pKa values (see Supplementary Material), yielding the following expression:

2 $${\rm{\Delta }}{G}_{j}^{op}(pH7)-{\rm{\Delta }}{G}_{j}^{op}(pH5)={\rm{\Delta }}{\rm{\Delta }}{G}_{j}^{op}=RTln\,\frac{1+{10}^{(7-pKa)}}{1+{10}^{(5-pKa)}}$$

We thus see that the difference between the ${\rm{\Delta }}{G}_{j}^{op}\,$ values is independent of the measured exchange rates. It only depends on the His187 pKa, and is therefore the same for every residue of the polypeptide for which exchange rates were measured at both pH values.

Replacing the His pKa by the measured experimental value (pKa = 4.94 ± 0.2) in Eq. 2, we obtain the following estimate for ${\rm{\Delta }}{\rm{\Delta }}{G}_{j}^{op}$:

3 $${\rm{\Delta }}{\rm{\Delta }}{G}_{j}^{op}=2.35\pm 0.15\,kcal/mol$$

This estimate is in reasonable agreement with the value of ${\rm{\Delta }}{\rm{\Delta }}{G}_{j}^{op}(shift)$ of Eq. 1 based on the linear fit to the data points in Fig. 2a, and supports the contention that the observed exchange in GHaPrP occurs from only one sub-state of the protein, corresponding to that with a protonated His 187 residue. This state exhibits lower ${\rm{\Delta }}{G}_{j}^{op}\,$ values across the polypeptide and is therefore less stable than the native neutral state. It might therefore qualify as an intermediate along the unfolding or aggregation pathway of the protein. The main effect of reducing the pH from 7 to 5 is to increase the population of this sub-state (by a factor of ~50), resulting in the pronounced and uniform pH-dependent behavior of the exchange rates of the protein as seen in Fig. 2a.

**Figure 4:**
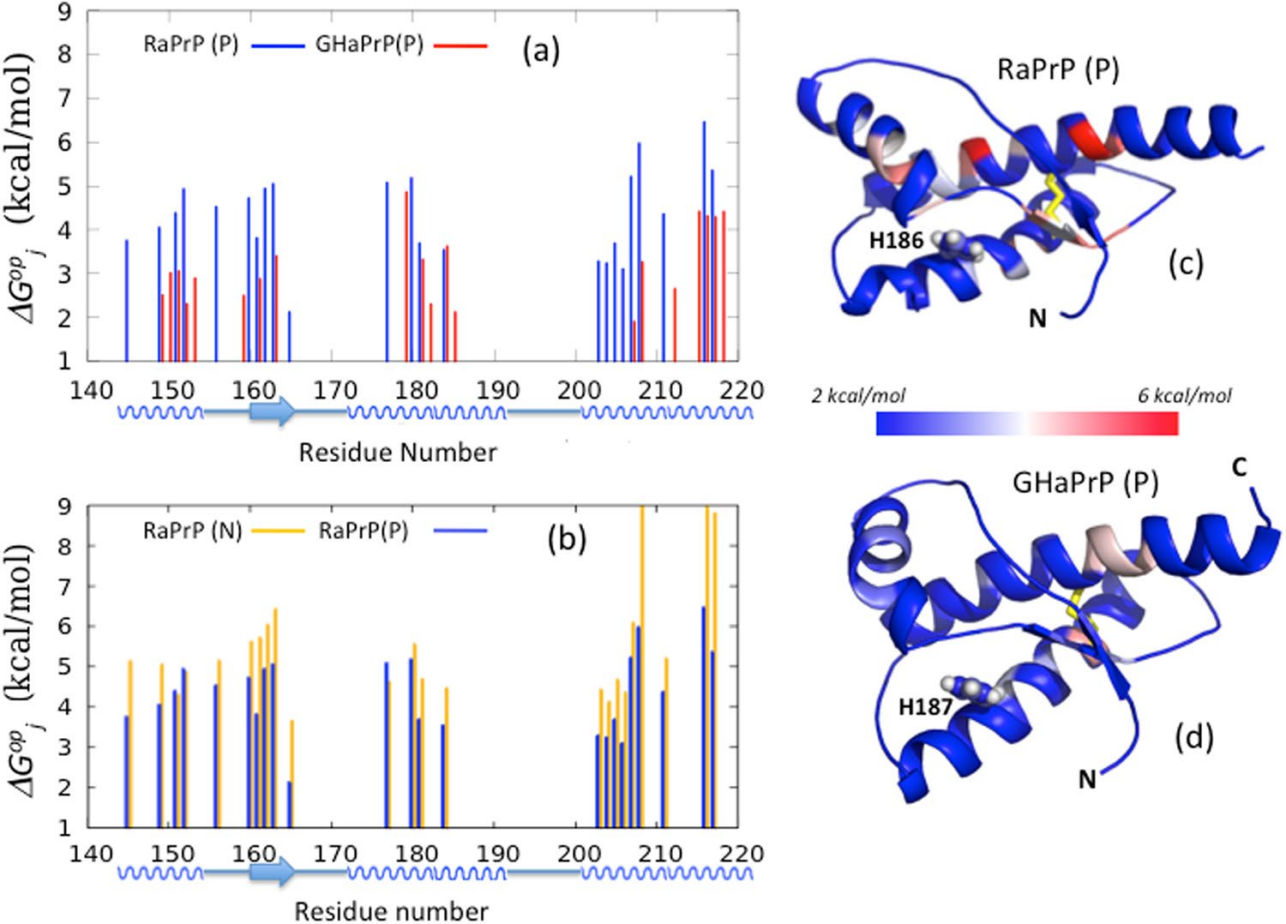
Plots of the de-mixed ${\rm{\Delta }}{G}_{j}^{op}\,$ values of the protonated forms $({\rm{\Delta }}{G}_{j}^{op}(P))$ of rabbit and hamster PrP (**a**). Plots for de-mixed ${\rm{\Delta }}{G}_{j}^{op}\,$ values for the neutral $({\rm{\Delta }}{G}_{j}^{op}(N))$ and protonated forms $({\rm{\Delta }}{G}_{j}^{op}(P))$ of Rabbit PrP (**b**). Limits of secondary structure elements, β-strands (arrow) and α-helices (wiggles) are depicted along the horizontal axis. Projection of the ${\rm{\Delta }}{G}_{j}^{op}(P)$ values (colored according to the shown scale) onto the rabbit PrP (**c**) and hamster PrP (**d**) backbones, respectively. The disulfide bridge (residues 179–214, hamster numbering) is highlighted in yellow, and the side chain of the buried His 187/186 residue is depicted in atomic detail.

Using the measured H187 pKa value of 4.94 ± 0.2, we can now derive the ${\rm{\Delta }}{\rm{\Delta }}{G}_{j}^{op}(P)$ values for individual residues in the ‘pure’ protonated state of GHaPrP (Fig. 4a) as:

4 $${\rm{\Delta }}{G}_{j}^{op}(P)={\rm{\Delta }}{G}_{j}^{op}(pH5)+RTln{P}_{Hi{s}^{+}}(pH5)$$

where *P*_*His*+_ *(pH5)* is the population of protonated H187 species at pH 5, and ${\rm{\Delta }}{G}_{j}^{op}(pH5)$ are the ${\rm{\Delta }}{G}_{j}^{op}\,$ values derived from the exchange data measured at pH 5 (see Supplementary Material). Since the neutral His 187 GHaPrP form does not contribute to the experimental exchange data according to our model, the corresponding ${\rm{\Delta }}{G}_{j}^{op}(N)$ values cannot be computed. All that can be said is that these values are sufficiently higher than those of ${\rm{\Delta }}{G}_{j}^{op}(P)$, so as not to contribute to the measured exchange rates (Fig. 3a).

### RaPrP

The behavior of RaPrP on the other hand, resembles that of the second scenario of our two-state model (Fig. 3c,d). According to this scenario the two RaPrP sub-states, corresponding to the neutral and protonated forms of His 186, feature similar amide exchange rates and hence exhibit similar ${\rm{\Delta }}{G}_{j}^{op}\,$ values. Both states therefore contribute to the measured exchange rates at both pH values. As a result, most points in the ${\rm{\Delta }}{\rm{\Delta }}{G}_{j}^{op}\,$ plot of Fig. 2b are located close to the diagonal, with a majority displaying only a small shift as a function of pH (<1 kcal), as in Fig. 3d. This suggests that the H186 protonated state of the protein exhibits native-like local stability and hence that the folded state of the rabbit variant is less perturbed upon lowering the pH than in GHaPrP, leading to a smaller pH-induced effect overall.

This is apparent from Fig. 4b, which plots the opening free energy values of the ‘pure’ protonated $({\rm{\Delta }}{G}_{j}^{op}(P))$ and neutral $({\rm{\Delta }}{G}_{j}^{op}(N))$ forms of RaPrP, obtained as described in the Supplementary Material. The ${\rm{\Delta }}{G}_{j}^{op}\,$ values of both forms are quite similar overall, except for three residues, V208, Q216, Y217, which are also outlier points in Fig. 2b. These are the most slowly exchanging residues (with the highest ${\rm{\Delta }}{G}_{j}^{op}\,$ values) in RaPrP under both pH conditions. All three are along helix H3 close to the H2/H3 contact region. This region is close to the 178–213 disulfide bridge, and represents the ‘hyper-stable’ core of PrP^26, 27^. The pH induced ${\rm{\Delta }}{\rm{\Delta }}{G}_{j}^{op}$ values for these outliers is ~2.5 kcal/mol, close to the value (2.3 ± 0.15 kcal/mol) estimated from the shift in the populations of PrP with a protonated His 186 residue when the pH changes from 7 to 5 (Eq. 3). This suggests that the exchange of these outliers is dominated by the contribution from the purely protonated form of RaPrP, whereas most other RaPrP residues exhibit ${\rm{\Delta }}{\rm{\Delta }}{G}_{j}^{op}\,$ values corresponding to the mixture of the neutral and protonated forms of the protein.

### Local stability properties of *His* + PrP deduced from the exchange data

Finally, to better understand the difference in pH susceptibility of the hamster and rabbit proteins, it is useful to compare the ‘pure’ de-mixed ${\rm{\Delta }}{G}_{j}^{op}(P)$ values of the protonated GHaPrP and RaPrP species, plotted in Fig. 4a, and mapped onto the PrP backbones in Fig. 4c,d.

We find that, in both proteins, regions with high ${\rm{\Delta }}{G}_{j}^{op}(P)$ values are generally centered on elements of PrP^C^ secondary structure, in line with the fact that protection from exchange is primarily afforded by hydrogen bonding within such structural elements and in agreement with previous findings^26, 27^. A notable exception is β-strand S1. Proton exchange of S1 residues is only quantifiable in RaPrP at pH 5 and otherwise occurs within the dead time of the experiment (see Supplementary Figure S3). The highest single residue stability often used as an estimate of global unfolding energy^48^, is lower for the His187/186 protonated forms of GHaPrP (4.8 ± 0.7 kcal/mol) than for RaPrP (6.5 ± 0.7 kcal/mol). This trend is in approximate agreement with the difference in global thermodynamic stability measurements reported below.

We also see that fewer residues have detectable levels of protection in the protonated from of GHaPrP (18 residues) than for RaPrP (23 residues) (Fig. 4a). The additional residues protected in RaPrP are primarily located in the N-terminal sub-domain (H1S2), including residues at the termini of H1 and S2. Additional protection in RaPrP versus GHaPrP occurs along the first half of H3, and to a lesser extent at the terminus of H2.

### Global thermodynamic stability of the neutral and protonated forms of GHaPrP and RaPrP

To obtain an independent measure of the destabilizing effect of His protonation, we used CD monitored urea denaturation experiments to derive the global thermodynamic stability, defined as the unfolding free energy (${\rm{\Delta }}{G}^{unfolding}$), of the rabbit and hamster PrP proteins. The experiments were carried out at pH7 and pH4 and in low salt concentration conditions under which the PrP proteins undergo minimal aggregation and follow a two-state unfolding reaction.

To derive the desired quantities from the denaturation data, we assume that at a given pH and urea concentration the PrP solution represents a mixture of two forms: a neutral form (*PrP*_*N*_) and a protonated form (*PrP*_*P*_), with each form adopting two states, folded and unfolded, present in different proportions during the denaturation reaction. Accordingly, the measured ellipticity as a function of pH and urea concentration (listed in the Supplementary Table S9) was expressed as an ensemble average of the ellipticities of the different forms and states of the protein. Taking into account the dependence of the free energy values on pH and urea concentration, the ensemble average expression was fitted to the observed ellipticity data as a function of 7 parameters, using a linear extrapolation model (see Supplementary Material). This procedure differs from the classical 2-state linear extrapolation model^49^, which does not distinguish between the protonated and neutral forms of the protein.

**Figure 5:**
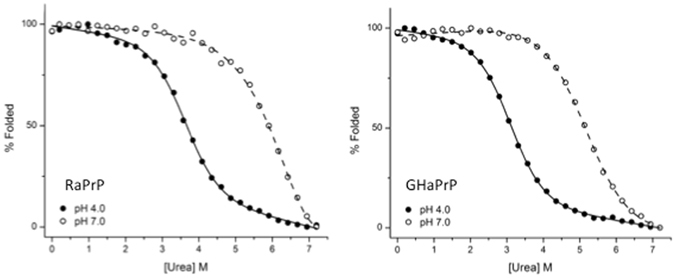
Urea denaturation curves of RaPrP and GHaPrP measured at pH 7 and pH 4. Experimental values (empty and filled circles for values at pH 7 and pH 4, respectively) were fitted (shown curves) assuming that at a given pH and urea concentration the solution represents a mixture of neutral and protonated forms of PrP, with each form adopting two states (folded and unfolded) in different proportions. The measured ellipticity as a function of pH and urea concentration (listed in the Supplementary Table S9) was expressed as an ensemble average of the ellipticities of the different forms and states of the protein. The ensemble average expression was fitted to the observed ellipticity data as a function of 7 parameters, using a linear extrapolation model, as described in the Methods Details section of the Supplementary Material. The unfolding free energy values and key parameters derived from the fit are listed in Table 2.

The unfolding free energies and values of other relevant parameters are listed in Table 2. The values of all the parameters derived from the fit can be found in the Supplementary Table S10. Notably, the m-values (*m* ^*u*^_*P*_ and *m* ^*u*^_*N*_), which represent the slopes of the urea concentration dependence of the unfolding free energies for the protonated and neutral PrP forms, respectively, are both around ~1.1 kcal mol^−1^ M^−1^, consistent with a 2-state unfolding model for both protein forms. The fitted denaturation curves for GHaPrP and RaPrP at pH 7 and pH 4 are displayed in Fig. 5.

**Table 2:** Unfolding free energies and key parameters derived from the fit to the urea denaturation data of hamster and rabbit PrP.

Protein	*ΔG*_*N*_^*unfolding*^ (kcal.mol^−1^)	*ΔG*_*P*_^*unfolding*^ (kcal.mol^−1^)	pKa^f^ (set values)	*m* ^*u*^_*N*_ (kcal.mol^−1^.M)	*m* ^*u*^_*P*_ (kcal.mol^−1^.M)
RaPrP	7.7 (0.56)	4.2 (0.19)	5.1	1.18 (0.05)	1.07 (0.05)
GHaPrP	6.1 (0.21)	3.4 (0.12)	4.9	1.15 (0.03)	1.09 (0.03)

*ΔG*_*N*_^*unlfoding*^ and *ΔG*_*P*_^*unfolding*^ are the unfolding free energies of the neutral and protonated forms of the PrP proteins. pKa^f^ are the pKa values of the native folded forms of the PrP proteins, set to those measured by the NMR titration experiments reported here. *m* ^*u*^_*N*_ and *m* ^*u*^_*P*_ are the unfolding free energy slopes ﻿for the protonated and neutral PrP forms as a function of urea concentration. The standard errors of the various parameters are in parentheses. Details of the fitting procedure are given in the Supplementary Material and values of the full set of fitted parameters are listed in the Supplementary Table S10.

We see that the unfolding free energy of the neutral form of RaPrP is markedly higher $({\rm{\Delta }}{G}_{N}^{unfolding}=7.7kcal/mol)$ than that of neutral GHaPrP ${\rm{\Delta }}{G}_{N}^{unfolding}=6.1\,kcal/mol$). Both proteins are substantially destabilized (by ~2.7–3.5 kcal/mol) upon His protonation. Protonation decreases the stability of RaPrP to 4.2 kcal/mol and that of GHaPrP to 3.4 kcal/mol. While the difference in stability between the protonated forms of the two proteins is only ~0.8 kcal/mol, the very low unfolding free energy of protonated GHaPrP, is likely associated with a more substantial perturbation of the native state than in RaPrP. This interpretation is supported by our analysis of the exchange data, which shows the protonated form of GHaPrP to feature lower local stability $({\rm{\Delta }}{G}_{j}^{op}(P))$ values and fewer residues for which exchange rates could be measured than for its protonated rabbit homolog (Fig. 4a).

Overall, the unfolding free energy values reported here are in good general agreement with previous reports on the lower stability of hamster PrP relative to the rabbit variant^50^. They are consistent with the proposed ranking in order of decreasing stability of different PrP variants: Rabbit ≥ mouse > hamster^39^. Likewise, a value of ~4.7 kcal/mol was reported for hamster PrP at pH 5.2 and 20 mM sodium acetate^27^, a somewhat higher salt concentration than the one used here. This value approaches those of ~4 kcal/mol and 5.1 kcal/mol derived for hamster PrP applying the classical linear extrapolation model to our urea denaturation data at pH 4 and pH 7, respectively (see Supplementary Table S11), an indication that our urea denaturation data are consistent with previous findings.

## Discussion

Analysis of the hydrogen exchange data, and the discovery that a single buried His residue in each protein (H187 in GHaPrP, and H186 in RaPrP) features a substantially down shifted pKa value of ~5, led to the rational that the pH-induced response of both PrP proteins is driven by a close link between the destabilizing effect of H187/186 protonation and the global intrinsic stability of the proteins. Since His186/187 have nearly the same down shifted pKa value in both PrP variants, the decrease in protein stability due to His protonation should likewise be similar. However, a similar drop in stability has a more drastic effect on the PrP variant with lower global stability, enhancing its susceptibility to aggregation.

**Figure 6:**
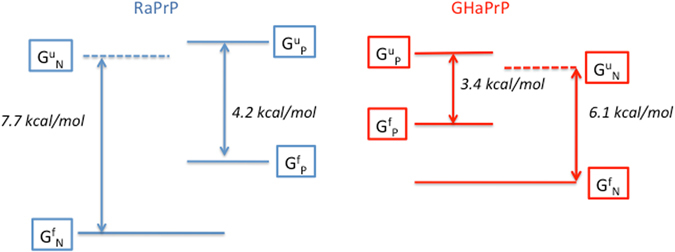
Schematic representation of the global thermodynamic stabilities (unfolding free energies) of the neutral and protonated forms of rabbit and hamster PrP. Horizontal lines indicate the free energy levels of the neutral (G^f^_N_), protonated (G^f^_P_) states of the folded hamster and rabbit PrP proteins, and those of the corresponding unfolded states (G^u^_N_, G^u^_P_). The free energies of the unfolded states of the protonated and neutral forms of the proteins differ by the cost of the His186/187 protonation. The values of the folding free energies of the neutral and protonated forms of the proteins are those derived in this study from the urea denaturation data at pH 4 and pH 7 (see Supplementary Material for details) and listed in Table 2.

Our rational was validated by independent measurements of the unfolding free energies of the PrP proteins using CD monitored urea denaturation experiments. These measurements confirmed that the neutral form of GHaPrP is less stable (by ~1.6 kcal/mol) than that of RaPrP, and showed that both proteins are destabilized by His protonation. Owing to the lower global stability of GHaPrP, its protonated form is driven further down the unfolding free energy scale than protonated RaPrP, resulting in a marginally stable state, as summarized schematically in Fig. 6.

**Figure 7:**
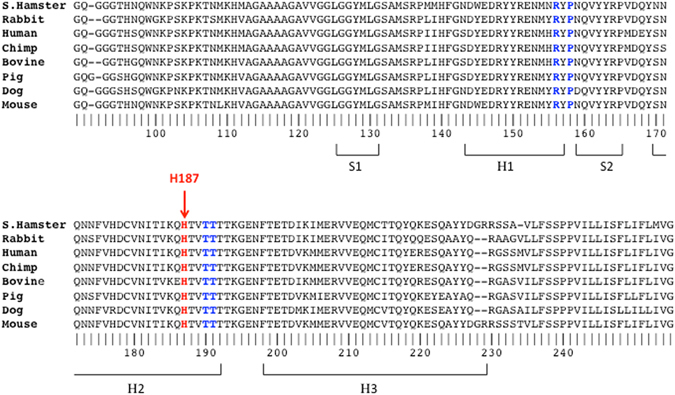
Multiple sequence alignment of prion proteins from eight mammalian species. The amino acid residues in the sequence (limited to residues 90–255) are numbered using the hamster PrP numbering. Limits of secondary structures (helices H1–H3, and β-strands S1, S2) are indicated. Highlighted are the conserved H187 residue (red fonts) and the residues that make contact (contact areas ≥ 10 Å^2^) with H187 in the three dimensional structures of PrP from hamster, rabbit, mouse, and human (blue fonts) (see Supplementary Table S13 for detail). The alignment was produced using the EggNoG server^60^.

Our findings on the role of His protonation are in excellent agreement with previous observations on the destabilizing effect of H186 protonation in *wt* mouse PrP^35^, and on the pH dependence of misfolding and oligomerization of *wt* mouse PrP^31^, which reveals an apparent pKa of 4.7 for the moPrP oligomerization reaction. The close correspondence of this pKa value with the pKa values of ~4.9–5.1 measured here for H186/187, thus confirms the identity of this buried His, which is widely conserved across mammalian PrP (Fig. 7) as the residue undergoing the critical protonation reaction required for prion misfolding and oligomerization. A more recent study^51^ shows indeed, that substituting His186 with the non-polar Phe in mouse PrP reduces the rate of misfolding, whereas the pathogenic H186R mutation has the opposite effect, confirming the destabilizing role of a positive charge at this buried position. It is noteworthy that this position is located in the region between helix 2–3 that is protected in the fiber^52^ and probably also in the oligomer^53^. The same region is also predicted to form amyloid structures (data not shown) by the Rosetta software^54^.

There is also ample evidence for the link between protein stability to unfolding and prion misfolding. An investigation of four point mutants in the C-terminus of helix 2, of mouse PrP^31^ showed that the extent of misfolding and aggregation displayed by the mutant proteins was inversely related to their global stability. These results join the ranks of previous findings on the link between the intrinsic global stability of PrP variants and their propensity to undergo conversion to the β-rich form^55^. Notably, a single residue substitution (V209M) in the protein core was shown to increase the stability of human PrP by ~2.4 kcal/mol, essentially abolishing conversion^56^. On the other hand, the F198S mutation in human PrP associated with the familial Gerstmann-Sträussler-Scheinker disease decreases PrP stability^55^. A similar mechanism of decreasing PrP stability may be at play in other sequence variants such as those that ablate or weaken the H2 helix N-cap in hamster versus rabbit PrP^41, 50^, which are also linked to increased disease susceptibility and the ease of pH- and/or denaturant-induced conversion to a β-rich form.

**Figure 8:**
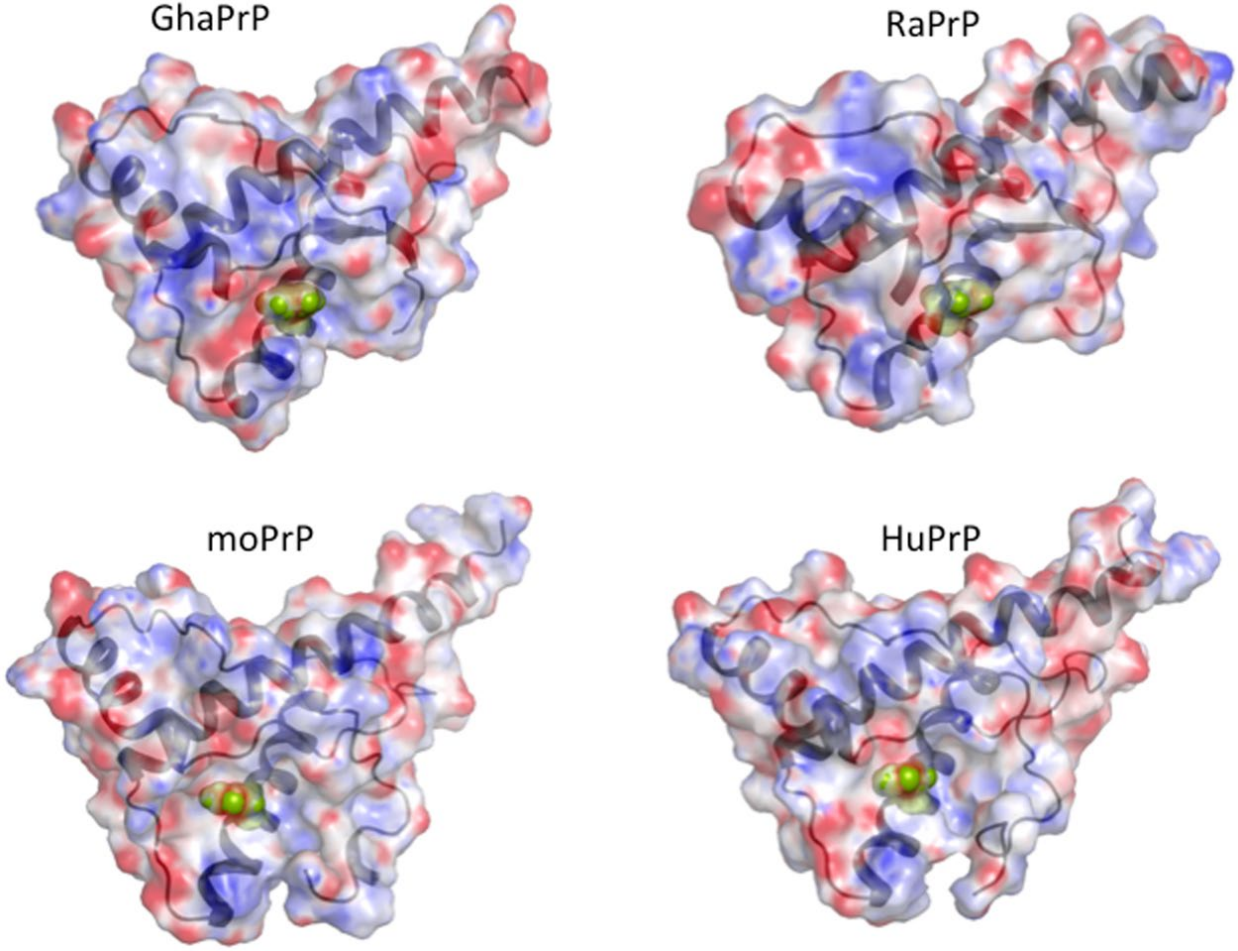
The electrostatic potential mapped onto the accessible surface area of PrP protein structures from hamster, rabbit mouse and human. Regions of negative potential are colored red, those of positive potential are colored blue, neutral regions are shown in grey. The protein backbone (ribbon representation) is depicted in dark grey. The H187/186 residue (green) is represented in full atomic detail. The surface representations illustrate the degree of burial of H187/186, and the similarity of the structural and electrostatic environments of this residue in the four mammalian PrP proteins. The electrostatic potential surface calculations were performed using the Delphi webserver^61^. The electrostatic calculations employed the non-linear Poisson-Boltzmann solver and the AMBER atomic charges and radii (with neutral His side chains). Results are visualized using the PyMOL software. The calculations were performed using a representative model from NMR ensembles of the corresponding PDB entries produced by the OLDERADO server (www.ebi.ac.uk/pdbe/nmr/olderado/ *)* (see Supplementary Table S13 for detail).

We therefore believe that the interplay between the protonation of the buried H186/187 residue and the global protein stability to unfolding is of general relevance to prion misfolding and aggregation. The buried His residue in question is highly conserved across mammalian PrP, as illustrated by the multiple sequence alignment in Fig. 7. Also evident from this alignment is the high level of sequence conservation across the PrP C-terminal globular domain (residues 122–231), including in the vicinity of the buried H186/187. In line with their high sequence identity, the globular domains of mammalian PrP adopt very similar 3D structures, where H186/187 is largely buried (see Fig. 8 and Supplementary Table S12) and engages in conserved tertiary contacts with surrounding residues (Fig. 7, and Supplementary Table S13).

We furthermore speculate that the protonated form of PrP represents a very early intermediate along the unfolding or conversion pathways of the protein. It is reasonable to assume that this early intermediate is monomeric, since the NMR-measured exchange rates, which led to its discovery, represent those of the monomeric species. With a pKa~5 for the buried His, its protonated form will be sufficiently populated even at neutral pH (~1% at pH 7) to make a contribution to the misfolding pathway under physiological conditions.

We did not investigate the role of acidic residues (Glu and Asp). Analysis of the available 3D structures for the hamster and rabbit proteins^7, 57^ and many others, the majority of which are solution structures, indicates that none of the 13 acidic residues are sufficiently shielded from solvent to expect a significant pKa shift that would destabilize the native protein upon protonation^45^. The low pH-induced misfolding transition of the H186F moPrP mutant, mentioned above, was indeed shown to feature an apparent pKa of ~3.8^51^, a virtually identical value to the pKa of solvated acidic residues. This rules out the possibility that protonation of these residues destabilize the monomeric state of PrP. Protonation of specific acidic residues may nonetheless play an important role in stabilizing PrP aggregates or oligomers at low pH, since neutralizing the negative charge of such residues would be equivalent to screening these charges by high salt concentration, which is known to foster aggregation (see Supplementary Material for detail discussion).

Lastly, the very presence of the buried His residue in the core of PrP may be the hallmark sequence feature required for the conversion to the scrapie form at physiological pH. Doppel, a homologue of PrP that features a very low global stability of ~3 kcal/mol does not undergo conversion to infectious prion^27^. Interestingly, inspection of the aligned sequences of Doppel and related PrP proteins indicates that it conserves the disulfide bridge in the stable protein core, but lacks some of the histidines, notably the buried His 187 (in GHaPrP), which is Asn 117 in Doppel.

## Methods

### Protein Sample Preparation

The C terminal domains (90–231) of wild-type rabbit PrP (RaPrP) and golden hamster PrP (GHaPrP) were expressed from a pProEx-Htb vector with an N-terminal 6xHIS tag linked by a TEV cleavage site. The proteins were expressed in *E. coli* BL21 cells expressing tRNA for rare E. coli codons. The constructs were optimized by replacing rare codons with those more common to *E. coli*. Cell cultures were grown in M9 minimal media supplemented with 25 µg/ml chloramphenicol and 100 µg/ml ampicillin as well as ^15^N NH_4_Cl and/or ^13^C glucose. M9 cultures were grown at 37 °C for 6–8 h until OD_600_ ~ 0.7–0.8 at which point temperature was decreased to 25 °C and IPTG was added to a final concentration of 1 mM to induce overnight protein expression.

The protein was extracted from the insoluble pellet using 6 M guanidinium chloride and purified through Ni-affinity chromatography under denaturing conditions. Refolding was accomplished by a two-step method. The Ni elution was diluted 10-fold drop-wise into refolding buffer 1 (1.1 M guanidinium chloride, 55 mM Tris, 21 mM NaCl, 0.88 mM KCl, 1 mM EDTA, 1 mM reduced glutathione, 1 mM oxidized glutathione, pH 8.2) at 25 °C. The sample was then dialyzed against refolding buffer 2 (20 mM Tris, 150 mM NaCl, pH 8.0) at 4 °C. Final refolding yields were approximately 70–80%. Refolding yields were reduced to approximately 30% under more reducing conditions pointing to the critical role of disulphide formation in PrP^C^ folding.

Protein samples were dialyzed against TEV cleavage buffer (20 mM Sodium phosphate, 150 mM NaCl, pH 7.0) at 4 °C overnight. Prior to dialysis 1 mg TEV protease was added to the sample to remove the N-terminal 6xHIS tag. Following cleavage, protein samples were concentrated and subjected to size-exclusion chromatography. An additional Ni-affinity column was utilized to remove residual uncleaved protein as necessary. The sample was dialyzed into the NMR buffer used for the HDX experiments (20 mM sodium phosphate, pH 7, or 20 mM sodium acetate, pH 5) and concentrated to ~0.5–1 mM immediately prior to NMR experiments. Assignments for the GhaPrP90-231 and RaPrP90-231 were made using standard triple resonance experiments^58^ at pH 7 and pH titrations to pH 5 were used to follow resonances and transfer assignments from pH 7. Assignments previously deposited in the BMRB (Biological Magnetic Resonance Bank) were helpful during the assignment process, but because the chemical shifts vary substantially depending on the ionic strength, temperature and pH of the samples, we needed to assign the chemical shifts under our conditions to be confident of the assignments. Our assignments for RaPrP_90–231_ and GHaPrP_90–231_ were deposited in the BMRB, with accession numbers 26998 and 27047, respectively.

### Experimental determination of pKa of His residues in GHaPrP_90–231_ and RaPrP_90–231_

In order to measure histidine pKa values, ^15^N labeled GHaPrP_90–231_ or RaPrP_90–231_ were exchanged into a buffer containing 36 mM sodium phosphate, 36 mM sodium citrate and 10% D_2_0, pH 8.5. 5 mM of 1-methylimidazole was added as an internal NMR observable pH control and 1 mM of 4,4-dimethyl-4-silapentane-1-sulfonic acid (DSS) was added as a chemical shift reference. During the titration, the pH of the samples was adjusted by the addition of aliquots of 1 M HCl followed by rapid mixing and measurement of the pH using a pH meter. At each pH, HSQC and HMBC^43^ experiments were recorded to determine the folding status and histidine protonation state of the PrP protein. All the experiments were recorded at a protein concentration of 1 mM and a temperature of 20 °C. For RaPrP_90–231_ experiments were recorded at pH 8.45, 7.98, 7.71, 7.42, 7.12, 6.85, 6.52, 6.28, 6.0, 5.71, 5.42, 5.12, 4.84, 4.51, 4.17, 3.85 and 3.51. GHaPrP_90–231_ experiments were recorded at pH 8.21, 7.71, 7.41, 7.08, 6.74, 6.39, 6.02, 5.71, 5.39, 5.05 4.70, 4.36, 3.99 and 3.57. Histidine side chain chemical shifts obtained from the HMBC experiment were fit to the Henderson-Hasselbalch equation to obtain separate histidine pKa values for each histidine.

### Proton-deuterium exchange experiments

Slow proton exchange (on the order of hours) was measured using proton-deuterium exchange experiments. Exchange was monitored for both GHaPrP_90–231_ and RaPrP_90–231_ at pH 7 and pH 5 at 500 MHz field strength. Calibration of pulse widths for HSQC experiments were performed on lyophilized samples dissolved in buffer containing 10% D_2_O. Experimental spectra were performed on lyophilized samples dissolved in 100% D_2_O-containing NMR buffer.

The dead time for each set of HSQC experiments was the time the sample was dissolved in 100% D_2_O buffer to the initiation of the first experiment and was generally 3–5 minutes. Data points were collected by recording successive HSQC spectra with variable number of transients (nt) recorded per experiment. Early time-points were measured frequently (5 min experiments with nt = 2) while later time-points were measured less frequently (160 min experiments with nt = 64) allowing for an increase in the signal to noise ratio required to detect diminished signals. Time points used to calculate exchange rates were taken at the completion time of each experiment. The hydrogen exchange decay curves are shown in the Supplementary Figs S4–S7.

### Analysis of Proton-Deuterium Exchange Data

Peak intensities for single amide resonances at each time-point were measured. The decay of the signal as a function of time was fit to the following equation:

5 $${I}_{t}={I}_{0}{e}^{-{k}_{ex}t}$$

where *I*_*t*_ is the peak intensity at time *t, I*_*0*_ is the initial peak intensity, and *k*_*ex*_ is the measured proton exchange rate. The fitted curves are provided as supplementary material.

In general, the standard local unfolding model can be used to describe amide proton exchange with bulk solvent^48, 59^:

6 $$A(cl)\frac{\mathop{\to }\limits^{{k}_{op}}}{\mathop{\leftarrow }\limits_{{K}_{cl}}}A(op)\mathop{\to }\limits^{{k}_{hx}}B$$

where *A*(*cl*) represents the ‘closed’, exchange-incompetent state, of the amide proton, *A*(*op*) represents the ‘open’, exchange-competent state, and *B* represents the state (or states) after proton exchange with the bulk solvent has taken place. *k*_*op*_ and *k*_*cl*_ are the associated rate constants for the local conformational transitions of the protein at a given residue position *j*. *k*_*hx*_ is the intrinsic proton exchange rate, determined using random-coil models^46^, taking into account the pH dependence of this rate (a decrease by a factor of 10 per pH unit, as the pH is lowered from 7 to 5^48, 59^).

At or below neutral pH, most proteins exchange via an EX2 mechanism where the closed state is favored (*k*_*cl*_ ≫ *k*_*hx*_ *)* and the observed proton exchange rate of residue *j* (*k*_*ex,j*_ *)* is expressed as follows:

7 $${k}_{ex,j}={k}_{op,j}.{k}_{hx}/{k}_{cl,j}={K}_{op,j}.{k}_{hx}$$

The equilibrium constant (*K*_*op,j*_) is then related to the free energy of opening *ΔG* ^*op*^_*j*_, by the following equation^46, 47^:

8 $${\rm{\Delta }}{G}_{j}^{op}=-RTln({K}_{op,j})=-RTln({k}_{ex,j}/{k}_{hx})$$

here we present a variant of the standard model, which considers that the native state of the protein comprises a mixture of two distinct sub-states {*A1*, *A2*}. Each sub-state *A*_*i*_ has a different equilibrium constants between the open *A*_*i*_(*op*) and closed *A*_*i*_(*cl*) conformations of individual amide groups (see Supplementary Figure S9).

The observed per-residue exchange rate is then given by the sum of the per-residue rates *k*_*i,j*_, times the relative population of each sub-state *P*_*i*_:

9 $${k}_{ex,j}=\sum _{i=1,2}{P}_{i}{k}_{i,j}$$

*k*_*i,j*_ for each residue and sub-state *i* is then given by:

10 $${k}_{i,j}={k}_{hx}{e}^{\frac{-{\rm{\Delta }}{G}_{i,j}^{op}}{RT}}$$

with ${\rm{\Delta }}{G}_{i,j}^{op}$ being the opening free energy of residue *j* in sub-state *i*. and the overall ${\rm{\Delta }}{G}_{j}^{op}$ derived from the experimentally measured rates than becomes:

11 $${\rm{\Delta }}{G}_{j}^{op}=-RTln\frac{{K}_{ex,j}}{{k}_{hx}}=-RTln\sum _{i=1,2}{P}_{i}{e}^{\frac{-\Delta {G}_{i,j}^{op}}{RT}}$$

The right hand side of Eq. 11 expresses the fact that the overall apparent ${\rm{\Delta }}{G}_{j}^{op}\,$ results from a mixture of two sub-states, each present at a different population level *P*_*i*_. Changes in the experimental conditions, such as reducing the pH from 7 to 5, may bring about changes in the relative populations of the different sub-states. Assuming that these changes occur on a much faster timescale than the measurable exchange rates, these rates must be evaluated independently for different sub-states and linearly combined as stipulated by Eq. 9.

### Urea denaturation experiments

GHaPrP_90–231_ and RaPrP_90–231_ were prepared in 50 mM Tris-HCl, 150 mM NaCl, 1 mM EDTA, pH 8.5 as described above. The proteins were concentrated and buffer exchanged by centrifugal filter concentrators (Millipore®) into 4 stocks: 90 μM GHaPrP_90–231_ in 10 mM sodium acetate pH 4.0; 90 μM GHaPrP_90–231_ in 10 mM sodium phosphate pH 7.0; 90 μM RaPrP_90–231_ in 10 mM sodium acetate pH 4.0; 90 μM RaPrP_90–231_ in 10 mM sodium phosphate pH 7.0. The low salt conditions were used to minimize PrP aggregation during the unfolding process^31^, especially at low pH. pH 4, was chosen because at this pH the protonated form represents 90% of the monomeric PrP molecular species. For each stock, twenty-nine 500 μL samples were prepared independently, each containing 9 μM protein, 10 mM sodium phosphate (pH 7) or 10 mM sodium acetate (pH 4), and urea at concentrations ranging from 0–7.2 M. Samples were incubated for 5 days at room temperature and Circular Dichroism (CD) at 220 nm was measured using an Aviv CD spectrometer model 62DS in a 1-mm path-length quartz cuvette. Values were time-averaged over 60 s, normalized, and converted to % folded for curve fitting. The measured ellipticity values are listed in Supplementary Table S9. Not sure what was added. I verified all references and they seem to be exactly the same as those in the submitted material. I saw that ranges of reference numbers were converted to explicit numbers. These conversions were correct.

## Electronic supplementary material

Supplementary information

Supplementary Figure S4

Supplenetary Figure S5

Supplementary Figure S6

Supplementary Figure S7

## References

[1] Prusiner SB (1998). Prions. Proc Natl Acad Sci USA.

[2] Griffith JS (1967). Self-replication and scrapie. Nature.

[3] Prusiner SB (1982). Novel proteinaceous infectious particles cause scrapie. Science.

[4] Prusiner SB (1998). The prion diseases. Brain Pathol.

[5] Li J, Mei FH, Xiao GF, Guo CY, Lin DH (2007). 1H, 13C and 15N resonance assignments of rabbit prion protein (91–228). J Biomol NMR.

[6] Zahn R (2000). NMR solution structure of the human prion protein. Proc Natl Acad Sci U S A.

[7] Liu H (1999). Solution structure of Syrian hamster prion protein rPrP(90-231). Biochemistry.

[8] Donne DG (1997). Structure of the recombinant full-length hamster prion protein PrP(29-231): the N terminus is highly flexible. Proc Natl Acad Sci USA.

[9] Christen B, Perez DR, Hornemann S, Wuthrich K (2008). NMR structure of the bank vole prion protein at 20 degrees C contains a structured loop of residues 165-171. J Mol Biol.

[10] Hornemann S, Schorn C, Wuthrich K (2004). NMR structure of the bovine prion protein isolated from healthy calf brains. EMBO Rep.

[11] Riek R (1996). NMR structure of the mouse prion protein domain PrP(121–231). Nature.

[12] Lysek DA (2005). Prion protein NMR structures of cats, dogs, pigs, and sheep. Proc Natl Acad Sci USA.

[13] Calzolai L, Lysek DA, Perez DR, Guntert P, Wuthrich K (2005). Prion protein NMR structures of chickens, turtles, and frogs. Proc Natl Acad Sci USA.

[14] Gossert AD, Bonjour S, Lysek DA, Fiorito F, Wuthrich K (2005). Prion protein NMR structures of elk and of mouse/elk hybrids. Proc Natl Acad Sci USA.

[15] Prusiner SB (1983). Scrapie prions aggregate to form amyloid-like birefringent rods. Cell.

[16] Pan KM (1993). Conversion of alpha-helices into beta-sheets features in the formation of the scrapie prion proteins. Proc Natl Acad Sci USA.

[17] Singh J, Udgaonkar JB (2015). Molecular Mechanism of the Misfolding and Oligomerization of the Prion Protein: Current Understanding and Its Implications. Biochemistry.

[18] Baskakov IV, Legname G, Gryczynski Z, Prusiner SB (2004). The peculiar nature of unfolding of the human prion protein. Protein Sci.

[19] Hornemann S, Glockshuber R (1998). A scrapie-like unfolding intermediate of the prion protein domain PrP(121–231) induced by acidic pH. Proc Natl Acad Sci USA.

[20] Swietnicki W, Petersen R, Gambetti P, Surewicz WK (1997). pH-dependent stability and conformation of the recombinant human prion protein PrP(90–231). J Biol Chem.

[21] Jackson GS (1999). Multiple folding pathways for heterologously expressed human prion protein. Biochim Biophys Acta.

[22] Kremer W, Kachel N, Kuwata K, Akasaka K, Kalbitzer HR (2007). Species-specific differences in the intermediate states of human and Syrian hamster prion protein detected by high pressure NMR spectroscopy. J Biol Chem.

[23] Apetri AC, Surewicz K, Surewicz WK (2004). The effect of disease-associated mutations on the folding pathway of human prion protein. J Biol Chem.

[24] Collinge J (2001). Prion diseases of humans and animals: their causes and molecular basis. Annu Rev Neurosci.

[25] Weissmann C (1996). The Ninth Datta Lecture. Molecular biology of transmissible spongiform encephalopathies. FEBS Lett.

[26] Hosszu LL (1999). Structural mobility of the human prion protein probed by backbone hydrogen exchange. Nat Struct Biol.

[27] Nicholson EM, Mo H, Prusiner SB, Cohen FE, Marqusee S (2002). Differences between the prion protein and its homolog Doppel: a partially structured state with implications for scrapie formation. J Mol Biol.

[28] Hosszu LL (2005). Definable equilibrium states in the folding of human prion protein. Biochemistry.

[29] Kachel N, Kremer W, Zahn R, Kalbitzer HR (2006). Observation of intermediate states of the human prion protein by high pressure NMR spectroscopy. BMC Struct Biol.

[30] Torrent J, Alvarez-Martinez MT, Liautard JP, Balny C, Lange R (2005). The role of the 132-160 region in prion protein conformational transitions. Protein Sci.

[31] Singh J, Kumar H, Sabareesan AT, Udgaonkar JB (2014). Rational stabilization of helix 2 of the prion protein prevents its misfolding and oligomerization. J Am Chem Soc.

[32] van der Kamp MW, Daggett V (2010). Pathogenic mutations in the hydrophobic core of the human prion protein can promote structural instability and misfolding. J Mol Biol.

[33] van der Kamp MW, Daggett V (2010). Influence of pH on the human prion protein: insights into the early steps of misfolding. Biophys J.

[34] Viles JH (2001). Local structural plasticity of the prion protein. Analysis of NMR relaxation dynamics. Biochemistry.

[35] Bae SH (2009). Prion proteins with pathogenic and protective mutations show similar structure and dynamics. Biochemistry.

[36] Kuwata K, Kamatari YO, Akasaka K, James TL (2004). Slow conformational dynamics in the hamster prion protein. Biochemistry.

[37] Barlow RM, Rennie JC (1976). The fate of ME7 scrapie infection in rats, guinea-pigs and rabbits. Res Vet Sci.

[38] Gibbs CJ, Gajdusek DC (1973). Experimental subacute spongiform virus encephalopathies in primates and other laboratory animals. Science.

[39] Julien O (2011). Relative and regional stabilities of the hamster, mouse, rabbit, and bovine prion proteins toward urea unfolding assessed by nuclear magnetic resonance and circular dichroism spectroscopies. Biochemistry.

[40] Sweeting B, Khan MQ, Chakrabartty A, Pai EF (2010). Structural factors underlying the species barrier and susceptibility to infection in prion disease. Biochem Cell Biol.

[41] Khan MQ (2010). Prion disease susceptibility is affected by beta-structure folding propensity and local side-chain interactions in PrP. Proc Natl Acad Sci USA.

[42] Langella E, Improta R, Barone V (2004). Checking the pH-induced conformational transition of prion protein by molecular dynamics simulations: effect of protonation of histidine residues. Biophys J.

[43] Pelton JG, Torchia DA, Meadow ND, Roseman S (1993). Tautomeric states of the active-site histidines of phosphorylated and unphosphorylated IIIGlc, a signal-transducing protein from Escherichia coli, using two-dimensional heteronuclear NMR techniques. Protein Sci.

[44] Tanokura M (1983). 1H-NMR study on the tautomerism of the imidazole ring of histidine residues. I. Microscopic pK values and molar ratios of tautomers in histidine-containing peptides. Biochim Biophys Acta.

[45] Isom DG, Castaneda CA, Cannon BR, Garcia-Moreno B (2011). Large shifts in pKa values of lysine residues buried inside a protein. Proc Natl Acad Sci USA.

[46] Bai Y, Milne JS, Mayne L, Englander SW (1993). Primary structure effects on peptide group hydrogen exchange. Proteins.

[47] Connelly GP, Bai Y, Jeng MF, Englander SW (1993). Isotope effects in peptide group hydrogen exchange. Proteins.

[48] Bai Y, Milne JS, Mayne L, Englander SW (1994). Protein stability parameters measured by hydrogen exchange. Proteins.

[49] Pace CN, Shaw KL (2000). Linear extrapolation method of analyzing solvent denaturation curves. Proteins.

[50] Sweeting B, Brown E, Khan MQ, Chakrabartty A, Pai EF (2013). N-terminal helix-cap in alpha-helix 2 modulates beta-state misfolding in rabbit and hamster prion proteins. PLoS One.

[51] Singh J, Udgaonkar JB (2016). Unraveling the Molecular Mechanism of pH-Induced Misfolding and Oligomerization of the Prion Protein. J Mol Biol.

[52] Lu X, Wintrode PL, Surewicz WK (2007). Beta-sheet core of human prion protein amyloid fibrils as determined by hydrogen/deuterium exchange. Proc Natl Acad Sci USA.

[53] Larda ST, Simonetti K, Al-Abdul-Wahid MS, Sharpe S, Prosser RS (2013). Dynamic equilibria between monomeric and oligomeric misfolded states of the mammalian prion protein measured by 19F NMR. J Am Chem Soc.

[54] Goldschmidt L, Teng PK, Riek R, Eisenberg D (2010). Identifying the amylome, proteins capable of forming amyloid-like fibrils. Proc Natl Acad Sci USA.

[55] Vanik DL, Surewicz WK (2002). Disease-associated F198S mutation increases the propensity of the recombinant prion protein for conformational conversion to scrapie-like form. J Biol Chem.

[56] Kong Q (2013). Thermodynamic stabilization of the folded domain of prion protein inhibits prion infection *in vivo*. Cell Rep.

[57] Wen Y (2010). Unique structural characteristics of the rabbit prion protein. J Biol Chem.

[58] Kanelis V, Forman-Kay JD, Kay LE (2001). Multidimensional NMR methods for protein structure determination. IUBMB Life.

[59] Englander SW, Kallenbach NR (1983). Hydrogen exchange and structural dynamics of proteins and nucleic acids. Q Rev Biophys.

[60] Huerta-Cepas J (2016). eggNOG 4.5: a hierarchical orthology framework with improved functional annotations for eukaryotic, prokaryotic and viral sequences. Nucleic Acids Res.

[61] Smith N (2012). DelPhi web server v2: incorporating atomic-style geometrical figures intothe computational protocol. Bioinformatics.

