# Supplementary material for: Interplay of buried histidine protonation and protein stability in prion misfolding
Source: Sci Rep. 2017 Apr 13;7:882. doi: 10.1038/s41598-017-00954-7 (PMC5429843; doi:10.1038/s41598-017-00954-7)
Supplement: Supplementary file 1 — Supplementary information [file 41598_2017_954_MOESM1_ESM.pdf]

Interplay of buried histidine protonation and protein stability in prion misfolding

Supplementary Material

A. Malevanets[1], P. Andrew Chong[1,2], D.F. Hansen[2,3,+], P. Rizk[1,2], Y. Sun[4], H. Lin[1], R. Muhandiram[2,3], A. Chakrabartty[4], Lewis E. Kay[1,2,3,5], J.D. Forman-Kay[1,2,**] and Shoshana J. Wodak[1,2,3§**]

[1]Program in Molecular Structure and Function, Hospital for Sick Children, 555 University Ave., Toronto, ON, Canada M5G 0A4

[2]Department of Biochemistry and [3]Department of Molecular Genetics, University of Toronto, Toronto, ON, Canada M5S 1A8

[4]Department of Medical Biophysics, University of Toronto, Toronto, ON, Canada M5G 2M9

[5]Department of Chemistry, University of Toronto, Toronto, ON, Canada, M5S 3H6

[+]Current address: University College London, Division of Biosciences, London WC1E 6BT UK

[§]Current address: VIB Structural Biology Research Center, VUB, Pleinlaan 2 1050 Brussels, Belgium

** Corresponding authors.

Supplementary Tables

Table S1: Rabbit PrP exchange rates (k_{ex}) at pH 5.
Intrinsic rates k_{hx} were obtained from the SPHERE server (Zhang, 1995) and pH was taken into account. '*' denote amides where the offset and the exchange rate are strongly correlated due a slow decay rate (<1e^{-5} /s). In these cases the offset was fixed to 0. 'error (k_{ex})' represents the uncertainty associated with the fit (see legend of **Figs. S5-S8** for further details)

res #	k_{ex}(sec^{-1})	error (k_{EX})	k_{hx} (sec^{-1})	
128N-HN	6.203565e-04	2.289300e-05	0.464E-01	
130N-HN	7.689914e-04	5.879046e-05	0.486E-01	
138N-HN	1.244310e-03	2.257561e-05	0.533E-02	
146N-HN	1.527668e-04	2.067222e-06	0.226E-01	
147N-HN	1.452624e-04	2.338645e-06	0.412E-01	
149N-HN	1.414793e-05	1.259979e-06	0.258E-01	
150N-HN	3.671416e-05	1.811921e-06	0.570E-01	
151N-HN	1.603292e-05	1.107152e-06	0.265E-01	
152N-HN	1.371325e-05	5.619094e-06	0.106E+00	
153N-HN	1.952088e-04	9.717015e-06	0.860E-01	
155N-HN	9.643211e-05	4.713832e-06	0.570E-01	
156N-HN	1.110133e-05	4.295052e-07	0.378E-01	*
159N-HN	2.646162e-04	2.430830e-06	0.101E+00	
160N-HN	2.577756e-06	1.687355e-07	0.138E-01	*
161N-HN	1.668851e-05	1.562778e-06	0.168E-01	
162N-HN	3.432462e-06	2.493997e-07	0.258E-01	*
163N-HN	5.174089e-06	2.230025e-07	0.570E-01	*
165N-HN	7.492745e-05	8.115147e-07	0.533E-02	
168N-HN	1.152883e-03	4.311690e-05	0.362E-01	
172N-HN	2.474007e-04	2.660412e-06	0.206E+00	
174N-HN	1.207197e-04	3.873229e-06	0.486E-01	
175N-HN	3.346880e-05	2.224202e-06	0.101E-01	
177N-HN	4.854614e-05	2.153016e-06	0.172E+00	
180N-HN	1.474538e-05	2.029775e-07	0.943E-01	*
181N-HN	1.711200e-05	9.177196e-07	0.168E-01	
184N-HN	4.082144e-05	1.500601e-06	0.282E-01	
198N-HN	1.484144e-03	6.048330e-05	0.414E-01	
203N-HN	4.744372e-05	1.622685e-06	0.230E-01	
204N-HN	2.563479e-05	8.818710e-07	0.108E-01	
205N-HN	3.126626e-05	1.658950e-06	0.246E-01	
206N-HN	5.281722e-05	5.763950e-07	0.207E-01	
207N-HN	3.494794e-06	2.468780e-07	0.415E-01	*
208N-HN	2.129929e-07	1.266514e-07	0.144E-01	*
209N-HN	1.049294e-06	1.024238e-07	0.658E-02	*
211N-HN	1.124603e-05	2.026445e-07	0.396E-01	*
212N-HN	2.661674e-06	1.385109e-07	0.654E-01	*
216N-HN	4.710731e-07	1.071999e-07	0.767E-01	*
217N-HN	1.666844e-06	2.494605e-07	0.362E-01	*

Table S2: Rabbit PrP ΔG_j^{op} values and associated errors at pH 5.
ΔG_j^{op} values were calculated using Eq. (4) of the main text (Methods section). The listed errors are those carried over form the errors in k_{ex} values.

Res #	ΔG^{op} (kcal/mol)	error (ΔG^{op}) (kcal/mol)
128N-HN	2.51351	0.0214074
130N-HN	2.41538	0.0443495
138N-HN	0.847459	0.0105248
146N-HN	2.91082	0.00784986
147N-HN	3.28997	0.00933931
149N-HN	4.37402	0.0516623
150N-HN	4.28028	0.0286292
151N-HN	4.31676	0.0400588
152N-HN	5.21537	0.237700
153N-HN	3.54651	0.0288760
155N-HN	3.71774	0.0283567
156N-HN	4.73778	0.0224438
159N-HN	3.46295	0.00532895
160N-HN	5.00138	0.0379724
161N-HN	4.02791	0.0543229
162N-HN	5.19907	0.0421496
163N-HN	5.42177	0.0250023
165N-HN	2.48428	0.00628288
168N-HN	2.00789	0.0216953
172N-HN	3.91735	0.00623809
174N-HN	3.49401	0.0186122
175N-HN	3.32611	0.0385512
177N-HN	4.76093	0.0257274
180N-HN	5.10496	0.00798538
181N-HN	4.01331	0.0311109
184N-HN	3.80856	0.0213246
198N-HN	1.93895	0.0236408
203N-HN	3.60224	0.0198408
204N-HN	3.52048	0.0199562
205N-HN	3.88434	0.0307795
206N-HN	3.47837	0.00633065
207N-HN	5.46548	0.0409793
208N-HN	6.47869	0.344944
209N-HN	5.09352	0.0566249
211N-HN	4.75734	0.0104530
212N-HN	5.88907	0.0301879
216N-HN	6.99069	0.132011
217N-HN	5.81716	0.0868181

Table S3: Rabbit PrP exchange rates at pH7
See legend of Table S1 for details.

res #	$k_{ex}(sec^{-1})$	error (k_{ex})	k_{hx} (sec^{-1})	
145N-HN	2.399691e-04	7.574641e-06	0.146E+01	
149N-HN	4.696097e-04	2.002792e-05	0.252E+01	
151N-HN	1.381861e-03	2.253184e-04	0.215E+01	
152N-HN	2.283949e-03	5.619590e-04	0.917E+01	
156N-HN	5.755341e-04	2.178279e-05	0.373E+01	
160N-HN	9.023813e-05	2.592398e-06	0.132E+01	
161N-HN	1.007690e-04	5.902648e-06	0.163E+01	
162N-HN	8.456200e-05	3.102738e-06	0.252E+01	
163N-HN	1.032587e-04	3.666396e-06	0.565E+01	
165N-HN	1.007744e-03	1.104269e-04	0.481E+00	
177N-HN	1.333910e-03	2.263558e-04	0.388E+01	
178N-HN	1.276309e-05	2.386261e-06	0.984E+01	
180N-HN	6.860990e-04	1.746542e-05	0.937E+01	
181N-HN	5.744060e-04	3.186567e-05	0.163E+01	
183N-HN	1.434396e-05	3.818992e-07	0.132E+01	*
184N-HN	1.386215e-03	1.911615e-04	0.277E+01	
203N-HN	1.303617e-03	1.396908e-04	0.225E+01	
204N-HN	9.014487e-04	8.146282e-05	0.103E+01	
205N-HN	7.621571e-04	6.287844e-05	0.241E+01	
206N-HN	1.015759e-03	3.477654e-05	0.167E+01	
207N-HN	1.099560e-04	2.059633e-06	0.357E+01	
208N-HN	9.235361e-07	2.690062e-07	0.139E+01	*
210N-HN	3.896461e-06	1.502040e-07	0.940E+00	*
211N-HN	4.603764e-04	1.886219e-05	0.341E+01	
212N-HN	1.878046e-04	8.299582e-06	0.648E+01	
215N-HN	5.597237e-05	1.931531e-06	0.210E+01	
216N-HN	3.037216e-06	2.485169e-07	0.762E+01	*
217N-HN	4.238102e-06	2.062532e-07	0.356E+01	*

Table S4: ΔG_j^{op} values and standard deviations for Rabbit PrP at pH7
See legend of Table S2 for details.

res #	ΔG^{op} (kcal/mol)	error (ΔG^{op}) (kcal/mol)
145N-HN	5.07591	0.0183109
149N-HN	5.00276	0.0247401
151N-HN	4.28154	0.094588
152N-HN	4.83378	0.142732
156N-HN	5.11272	0.0219556
160N-HN	5.58694	0.0166654
161N-HN	5.64553	0.0339800
162N-HN	6.00147	0.0212850
163N-HN	6.35545	0.0205976
165N-HN	3.59319	0.0635665
177N-HN	4.64602	0.0984393
178N-HN	7.89654	0.108459
180N-HN	5.54693	0.0147671
181N-HN	4.63162	0.0321816
183N-HN	6.65830	0.0154448
184N-HN	4.42731	0.0799969
203N-HN	4.34197	0.0621615
204N-HN	4.10169	0.0524230
205N-HN	4.69467	0.0478587
206N-HN	4.31366	0.0198609
207N-HN	6.05141	0.0108661
208N-HN	8.28623	0.168971
210N-HN	7.21973	0.0223622
211N-HN	5.19052	0.0237674
212N-HN	6.08684	0.0256362
215N-HN	6.13564	0.0200185
216N-HN	8.5839	0.0474661
217N-HN	7.9465	0.0282314

Table S5: Hamster PrP exchange rates at pH 5
See legend of Table S1 for details.

Res #	k_{ex} (sec^{-1})	error (k_{ex})	k_{hx} (sec^{-1})	
146N-HN	1.334995e-03	5.433507e-04	0.127E-01	
149N-HN	1.880029e-04	2.038872e-05	0.378E-01	
150N-HN	5.307911e-05	3.567567e-06	0.258E-01	
151N-HN	1.090412e-04	5.593800e-06	0.570E-01	
152N-HN	1.890219e-04	1.335834e-05	0.265E-01	
153N-HN	2.691355e-04	2.733969e-05	0.106E+00	
156N-HN	1.155483e-03	3.481031e-04	0.106E+00	
157N-HN	5.410136e-04	8.500842e-05	0.378E-01	
159N-HN	3.847614e-04	4.493594e-05	0.750E-01	
161N-HN	3.619985e-05	1.196829e-06	0.138E-01	
162N-HN	2.786822e-05	2.443872e-06	0.168E-01	
163N-HN	2.694358e-05	1.711565e-06	0.258E-01	
178N-HN	8.797771e-04	2.147473e-04	0.172E+00	
179N-HN	9.601299e-06	5.409262e-06	0.120E+00	
181N-HN	1.121435e-04	5.670235e-06	0.943E-01	
182N-HN	1.220093e-04	8.359311e-06	0.168E-01	
184N-HN	3.764956e-06	3.238333e-07	0.129E-01	*
185N-HN	2.283864e-04	9.932602e-06	0.230E-01	
203N-HN	1.119591e-05	8.597151e-07	0.682E-02	*
206N-HN	1.665248e-04	1.271683e-05	0.246E-01	
207N-HN	3.009563e-04	3.078227e-05	0.207E-01	
208N-HN	5.519215e-05	2.019707e-06	0.415E-01	
209N-HN	1.151967e-06	3.248900e-07	0.144E-01	*
211N-HN	2.092972e-06	1.880957e-07	0.118E-01	*
212N-HN	1.537903e-04	1.021509e-05	0.396E-01	
215N-HN	1.005299e-05	3.388216e-07	0.103E+00	*
216N-HN	2.022374e-04	2.455513e-05	0.570E-01	
217N-HN	1.653234e-05	2.859394e-06	0.767E-01	
218N-HN	8.739393e-06	4.113754e-07	0.362E-01	

Table S6: Hamster PrP ΔG_j^{op} values pH 5
See legend of Table S2 for details.

Res #	ΔG^{op} (kacl/mol)	error (ΔG^{op}) (kcal/mol)
146N-HN	1.31227	0.236104
149N-HN	3.08955	0.0629113
150N-HN	3.60378	0.0389899
151N-HN	3.64615	0.0297591
152N-HN	2.87951	0.0409962
153N-HN	3.48124	0.0589286
156N-HN	2.63244	0.174762
157N-HN	2.47382	0.0911501
159N-HN	3.07150	0.0677495
161N-HN	3.46224	0.0191791
162N-HN	3.72920	0.0508713
163N-HN	3.99877	0.0368503
178N-HN	3.07323	0.141598
179N-HN	5.49529	0.326822
181N-HN	3.92308	0.0293312
182N-HN	2.86902	0.0397449
184N-HN	4.74142	0.0498959
185N-HN	2.68679	0.0252288
203N-HN	3.73527	0.0445450
206N-HN	2.90999	0.0443000
207N-HN	2.46468	0.0593336
208N-HN	3.85793	0.0212283
209N-HN	5.49538	0.163606
211N-HN	5.03154	0.0521337
212N-HN	3.23367	0.0385316
215N-HN	5.37952	0.0195515
216N-HN	3.28631	0.0704343
217N-HN	4.91799	0.100333
218N-HN	4.85195	0.0273062

Table S7: Hamster PrP exchange rates at pH 7
See legend of Table S1 for details.

res #	k_{ex} (sec^{-1})	error(k_{ex})	k_{hx}(sec^{-1})	
150N-HN	3.296351e-04	4.717225e-05	0.252E+01	
151N-HN	6.397152e-04	1.599004e-04	0.565E+01	
152N-HN	1.901192e-03	7.781606e-04	0.215E+01	
159N-HN	6.739058e-04	1.291400e-04	0.745E+01	
161N-HN	1.220356e-04	9.197076e-06	0.132E+01	
163N-HN	7.429065e-05	5.853642e-06	0.252E+01	
179N-HN	4.249758e-05	3.674640e-06	0.984E+01	
180N-HN	3.698405e-06	3.356384e-07	0.241E+01	*
181N-HN	3.159923e-04	3.773683e-05	0.937E+01	
182N-HN	5.738067e-04	1.209855e-04	0.163E+01	
184N-HN	1.292651e-05	4.125749e-07	0.124E+01	*
185N-HN	1.012104e-03	3.264017e-04	0.225E+01	
207N-HN	1.024540e-03	2.975505e-04	0.167E+01	
208N-HN	2.021406e-04	1.264284e-05	0.357E+01	
209N-HN	6.079018e-06	2.805419e-07	0.139E+01	*
211N-HN	1.050663e-05	4.128153e-07	0.940E+00	*
212N-HN	6.407382e-04	1.297489e-04	0.341E+01	
214N-HN	7.598989e-06	3.940751e-07	0.191E+02	*
215N-HN	3.675762e-05	2.460672e-06	0.103E+02	
216N-HN	8.484169e-04	1.730411e-04	0.565E+01	
217N-HN	7.596887e-05	6.401004e-06	0.762E+01	
218N-HN	3.857385e-05	3.369312e-06	0.356E+01	

Table S8: Hamster PrP ΔG_j^{op} values at pH 7
See legend of Table S2 for details.

res #	ΔG^{op} (kcal/mol)	error (ΔG^{op}) (kcal/mol)
150N-HN	5.20893	0.083015
151N-HN	5.29303	0.144999
152N-HN	4.09568	0.237436
159N-HN	5.42380	0.111164
161N-HN	5.41110	0.0437187
163N-HN	6.07691	0.0457083
179N-HN	7.19582	0.0501596
180N-HN	7.79858	0.0526454
181N-HN	5.99857	0.0692775
182N-HN	4.63223	0.122313
184N-HN	6.68250	0.0185151
185N-HN	4.48942	0.187081
207N-HN	4.30865	0.168475
208N-HN	5.69671	0.0362823
209N-HN	7.18851	0.0267712
211N-HN	6.64189	0.0227927
212N-HN	4.99795	0.117470
214N-HN	8.58497	0.0300834
215N-HN	7.30696	0.0388338
216N-HN	5.12855	0.118316
217N-HN	6.70849	0.0488783
218N-HN	6.65998	0.0506701

Table S9: Raw data (mdeg ellipticity (θ) at 220 nm) from urea denaturation experiments carried out for GHaPrP and RaPrP at pH 4 and pH 7, respectively (see Methods section of the main text for experiment details). Blanks for pH 7 is 10 mM sodium phosphate; blanks for pH 4 is 10 mM sodium acetate

[Urea]	GHaPrP pH4	GHaPrP pH7	RaPrP pH4	RaPrP pH7
7.2	-9.712026	-10.501394	-9.603494	-11.725901
6.9408	-9.778841	-10.819844	-9.438538	-12.55791
6.6816	-9.931731	-11.4916	-9.644207	-14.007431
6.4224	-10.200906	-11.91921	-9.810363	-15.611677
6.1632	-10.284033	-12.783742	-10.033079	-17.636793
5.904	-10.600281	-13.972198	-10.457837	-19.611335
5.6448	-10.51252	-15.176893	-10.881813	-21.146658
5.3856	-10.539579	-17.09136	-11.14182	-22.795382
5.1264	-10.866207	-19.134699	-11.642997	-23.916381
4.8672	-11.166	-21.068605	-12.032997	-24.581715
4.608	-11.545695	-22.70933	-13.040524	-24.471604
4.3488	-11.9566	-23.967176	-13.871044	-25.522467
4.0896	-12.786795	-24.93107	-15.342214	-26.043917
3.8304	-13.723706	-25.709953	-17.264884	-26.826876
3.5712	-15.119477	-26.263794	-19.052041	-26.090513
3.312	-16.634332	-26.523821	-21.567046	-26.386787
3.0528	-18.720763	-26.512613	-23.040661	-26.8549
2.7936	-20.759561	-26.863411	-24.286899	-27.345269
2.5344	-22.357653	-27.098488	-24.889369	-26.775418
2.2752	-23.678544	-27.008543	-25.710755	-27.163211
2.016	-24.462882	-27.289728	-25.894632	-27.005288
1.7568	-24.958962	-26.883007	-26.10483	-27.193382
1.4976	-25.36961	-27.158891	-26.726599	-27.307304
1.2384	-25.548928	-27.198124	-26.921796	-27.410631
0.9792	-25.739154	-27.035854	-27.732858	-26.986054
0.72	-26.112012	-26.670188	-27.554526	-27.505939
0.4608	-26.435232	-26.422002	-27.609754	-27.439999
0.2016	-26.5281	-26.2999	-27.237789	-27.515538
0	-26.070299	-26.954505	-27.07849	-26.983142
blank	-6.460539	-7.145355	-6.223689	-7.112294

Table S10: Unfolding free energies and other parameters derived from the fit to the urea denaturation data of GHaPrP and RaPrP.

$\Delta G_N^{unfolding}$ and $\Delta G_P^{unfolding}$ are the unfolding free energies of the neutral and protonated forms of the PrP proteins. pKa^f represent the pKa values of the native (folded) forms of the PrP proteins, which are set to those measured by the NMR titration experiments reported here. $m^u{}_N$ and $m^u{}_P$ are the slopes of the unfolding free energies as a function of urea concentration. The standard errors of the various parameters are in parentheses. A^f is the ellipticity of the folded proteins (zero urea concentration); R^f and R^u are the slopes of the urea dependent ellipticity values (assumed to be the same for the protonated and neutral forms of the proteins; see **Methods Details**).

Protein	***$\Delta G_N^{ulfodling}$ (kcal.mol^{-1})***	***$\Delta G_P^{unfolding}$ (kcal.mol^{-1})***	***pKa^f (set value)***	***$m^u{}_N$ (kcal.mol^{-1}.M)***	***$m^u{}_p$ (kcal.mol^{-1}.M)***	***A^f***	***R^f***	***R^u***
RaPrP	7.7 (0.56)	4.2 (0.19)	5.1	1.18 (0.05)	1.07 (0.05)	0.76 (0.02)	-0.01 ($0.3\ 10^{-2}$)	0.023 ($0.3\ 10^{-2}$)
GHaPrP	6.1 (0.21)	3.4 (0.12)	4.9	1.15 (0.03)	1.09 (0.03)	0.76 (0.01)	$0.30\ 10^{-2}$ ($0.3\ 10^{-2}$)	-0.016 ($0.2\ 10^{-2}$)

Table S11: Unfolding free energies, $\Delta G^{unfolding}$ and the slope (m_G) of the free energies as a function of urea concentration, derived from the urea denaturation data at pH 4 and pH7 (see main text for details) using the classical linear extrapolation model (Pace & Shaw, 2000). Standard error values are in parentheses**.**

Protein	$\Delta G^{unfolding}$ (kcal·mol^{-1})	m_G (kcal·mol^{-1}·M)
GHa-PrP-pH4	3.99 (0.19)	1.29 (0.06)
GHa-PrP-pH7	5.10 (0.29)	0.99 (0.07)
Ra-PrP-pH4	4.53 (0.40)	1.26 (0.11)
Ra-PrP-pH7	6.28 (0.54)	0.88 (0.07)

Table S12 : Burial of His 186/187 residues (or equivalent) in PrP from hamster, Rabbit, mouse and human
ASA values (column 2) represent static accessible surface areas computed from representative conformations of the NMR ensembles deposited in the PDB produced by the OLDERADO server (www.ebi.ac.uk/pdbe/nmr/**olderado/**).

Prion protein	ASA H186/187	Buried fraction (% of total surface area for His, in G-H-G)
GHaPrP (1B10):	51.3 Å^2	80%
RaPrP (2FJ3):	29.9 Å^2	87%
mPrP (2L1H):	44.7 Å^2	80%
HuPrP (2LSB):	41.3 Å^2	81%

Table S13: Residues in contact with His 186/187 in known PrP structures, from the indicated species.
Residue numbering is as in the corresponding PDB entries (PDB-RCSB code is indicated). Contact calculations were performed using *a representative model from each PDB entry as produced by the OLDERADO server (www.ebi.ac.uk/pdbe/nmr/**olderado/**)*. Column 3 lists the pairwise contact area (Å^2); only residues with contact area ≥10Å^2 are listed. Note that the contact areas are only indicative, given that they were derived using one of the conformations from the NMR ensemble. Contacts with adjacent residues in Helix 2 (T182/183, T190/191), and those with P158/157 and R155/156 are well conserved. In GHaPrP the contact with R156 is slightly weaker (7.6 Å^2).

Golden hamster, GHaPrP (1B10)

Res 1	Res 2	Contact area (Å^2)
HIS 187A	ARG 156A	(7.6)
HIS 187A	PRO 158A	44.9
HIS 187A	ASN 159A	10.7
HIS 187A	THR 183A	25.3
HIS 187A	THR 190A	11.3
HIS 187A	THR 191A	57.9

Rabbit, RaPrP (2Fj3)

HIS 186A	TYR 154A	10.5
HIS 186A	ARG 155A	14.8
HIS 186A	PRO 157A	41.5
HIS 186A	THR 182A	11.0
HIS 186A	THR 190A	56.8
HIS 186A	THR 191A	16.8

Mouse, mPrP(2L1H)

Res 1	Res 2	Contact area
HIS 187A	ARG 156A	12.8
HIS 187A	PRO 158A	47.0
HIS 187A	THR 183A	23.5
HIS 187A	THR 190A	32.4
HIS 187A	THR 191A	40.5
HIS 187A	PHE 198A	36.3
HIS 187A	MET 206A	10.9

Human , HuPrP (2LSB)

HIS 187A	ARG 156A	31.6
HIS 187A	PRO 158A	36.0
HIS 187A	THR 183A	17.4
HIS 187A	THR 190A	30.1
HIS 187A	THR 191A	45.3

Legends of Supplementary Figures.

Figure S1. Fits of pH titration curves for hamster (a: nitrogen and b: proton) and rabbit (c: nitrogen and d: proton) histidine sidechain resonances for histidines H1 H2 H3 H4 and H187 as illustrated. H1-H4 correspond to H7 H11 H140 and H177 residues in hamster PrP in any order as they were not assigned in the NMR experiment. Blue circles are data points and the red line is the fit line. The Nε2 and Hδ2 is missing for both rabbit and hamster H187/H186. In the rabbit case only the Nε2-Hε1 resonance had enough reliable data points to fit the pKa.

Figure S2: ΔG_j^{op} (ΔG_{hx}) for hamster and rabbit PrP, derived from the hydrogen deuterium exchange data at pH 7 and pH 5 as described in the main text. The associated standard errors (listed in Tables S2, S4, S6, S8) are the values delimited by two small horizontal bars at the top of each line representing the ΔG_j^{op} value. The secondary structure elements of the PrP protein are schematically outlined between the two panels.

Figure S3: $\Delta\Delta G_j^{op}$ plots illustrating the change in ΔG_j^{op} values as the pH is reduced from 7 to 5 for the hamster and rabbit prion proteins. Higher ΔG_j^{op} values reflect lower proton exchange rates. The dashed diagonal corresponds to no change in ΔG_j^{op} as a function of pH. Solid lines represent the lines of best fit to the ΔG_j^{op} values. This Figure is the same as Fig. 2 of the main text except that here the errors associated

with experimentally derived ΔG_j^{op} values are displayed as vertical and horizontal error bars. These errors were estimated as follows: A linear fit to the data point of Fig. 2 (main text) (excluding that for Glu152) yields a line whose intercept with the abscissa corresponds to the shift in ΔG_j^{op} as a function of pH, denoted as $\Delta\Delta G_j^{op}$ *(shift).* From this intercept we obtain:

$$\Delta\Delta G_j^{op}(Shift) = 1.85 \pm 0.1\ kcal/mol \qquad (1)$$

Considering the error range for these values to be $\delta pH=0.2$ we estimate the error in ΔG^{op} values as:

$$\delta\Delta G_j^{op} = RT\ln 10\ \delta pH = 0.3\ kcal/mol \qquad (2)$$

Taking into account the error in ΔG_j^{op} values of Eq.(2) we obtain the following corrected estimate for $\Delta\Delta G_j^{op}(Shift)$*as :*

$$\Delta\Delta G_j^{op}(Shift) = 1.85 \pm 0.7\ kcal/mol \qquad (3)$$

where the value of 0.7 = (0.1 + 2 x 0.3) kcal/mol represents the cumulative errors from the linear fit and the experimental pH values. With a few exceptions this error is an order of magnitude larger than the errors listed in Tables S2,S4,S6,S8. These latter errors were therefore not taken into account here.

Figures S4-S7: Hydrogen exchange decay curves for hamster and rabbit PrP at pH 5

and pH 7. A total of 4 files (2 for each PrP protein) are provided as separate supplementary files.

Decay curves of peak intensities in 15N-1H correlation maps (O) vertical bars represent the uncertainty of the obtained intensity. The intensities were obtained from a simultaneous analysis and global fit of all spectra with different tau as described previously (Hansen et al (2007)). The solid lines are obtained from best-fits of an exponential decay function I(t)=I0*exp(-k_{ex}*tau) + offset to the data shown. For peaks with a decay rate slower than ~1e-5 /s the offset and the k_{ex} rate are strongly correlated and the offset was therefore fixed to 0 for these peaks.

Figure S8: The thermodynamic cycle describing the processes linking the different states and form forms the PrP proteins considered in fitting the urea denaturation data following the procedure described in **Methods Details** (below).

The four corners of the cycle represent the free energies of the 4 forms/states of PrP: G_N^u, G_N^f, G_P^f, G_P^u . The vertical direction concerns the unfolding process of the neutral and protonated forms of PrP respectively, with the corresponding free energies ($\Delta G_N^{unfolding}$ and $\Delta G_P^{unfolding}$). The horizontal directions represent the protonation process of the folded and unfolded states of PrP, respectively, as formulated in Eq. (14). The various other free energy differences are those used in fitting the urea denaturation data. They are computed relative to the free energy of the neutral folded state (G_N^f), used as the common reference (Eq.15).

Figure S9: Schematic illustration of the two sub-states model for hydrogen deuterium exchange. $A^j(cl)$ and $A^j(op)$ represent the 'closed' and 'open' states of an individual amide-proton of residue *j*. Subscripts 1,2 represent different sub-states sampled by the system. The individual rate constants for each sub-state represent those of individual residues of the protein.

Methods Details

Computing the ΔG^{op} values of the 'pure' protonated form of hamster PrP and the protonated and neutral forms of rabbit PrP.

We start from Eq. (7) of the main text (Methods section) that describes the experimentally derived per-residue opening free energy in the multi-state model:

$$\Delta G_j^{op} = -RTln\frac{K_{ex\,j}}{k_{hx}} = -RTln\sum_i P_i e^{\frac{-\Delta G_{i\,j}^{op}}{RT}} \quad (4)$$

We then recast this equation for the case where the PrP system samples only 2 sub-states corresponding to those where His 187/186 is either neutral or protonated respectively and the per-residue opening free energies are derived from experiments performed at pH7 and pH5 respectively:

$$\Delta G_j^{op}(pH7) = -RTln[p_1 e^{\frac{-\Delta G_{j,N}^{op}}{RT}} + (1-p_1)e^{\frac{-\Delta G_{j,P}^{op}}{RT}}] \quad (5)$$

$$\Delta G_j^{op}(pH5) = -RTln[p_2 e^{\frac{-\Delta G_{j,N}^{op}}{RT}} + (1-p_2)e^{\frac{-\Delta G_{j,P}^{op}}{RT}}] \quad (6)$$

$\Delta G_j^{op}(pH)$ are the experimentally derived opening free energies per residue at the set pH and p_1 and p_2 are the populations of species with neutral His residues at pH7 and pH5 respectively. $\Delta G_{j,N}^{op}$ and $\Delta G_{j,P}^{op}$ are the opening free energies of the 'pure' (de-mixed) species that are typically computed in protein simulations.

The populations p_1 (and p_2) of neutral His species at a given pH denoted here as [His] can be obtained from the chemical equilibrium equation and the measured pKa values:

$$[His] = \frac{1}{1+10^{pKa-pH}} \quad (7)$$

Introducing the values of p_1 and p_2 derived from Eq. (7) into Eq. (2) then allows to derive the values of the per-residue opening free energies of the 'pure' species:

$$\Delta G_{j,N}^{op} = -RTln\frac{1}{p1-p2}[(1-p_2)e^{\frac{-\Delta G_j^{op}(pH7)}{RT}} - (1-p_1)e^{\frac{-\Delta G_j^{op}(\mathrm{pH5})}{RT}}] \quad (8)$$

$$\Delta G_{j,p}^{op} = -RTln\frac{1}{p1-p2}[-p_2\, e^{\frac{-\Delta G_j^{op}(pH7)}{RT}} + p_1 e^{\frac{-\Delta G_j^{op}(\mathrm{pH5})}{RT}}] \quad (9)$$

For the solution of Eqs (8) and (9) to exist the values of the expressions whose

logarithm is computed must be positive. This in turn imposed the following restrictions on the value of the difference *ΔG(pH7) – ΔG (pH5):*

$$RTln\frac{1-p_2}{1-p_1} \geq \Delta G_j^{op}(pH7) - \Delta G_j^{op}(pH5) \geq RTln\ \frac{P_2}{p_1} \qquad (10)$$

For rabbit PrP, Eqs (8) and (9) and the experimentally measured pKa=5.1 are used to derive the 'pure' ΔG_j^{op} values of the neutral and protonated PrP species (Fig. 4b of the main text and rabbit ΔG_p^{op} plot of Fig. 4a).

When the proton exchange occurs only through the protonated sub-state as for hamster PrP one of the inequalities in Eq. (10) becomes an equality. Formally this can be expressed as:

$$p_1 e^{-\Delta G_{j,N}^{op}/RT} \ll (1-p_1)e^{-\Delta G_{j,p}^{op}/RT} \qquad (11)$$

Using Eq. (11), Eq. (9) and populations from Eq. (7) computed with pKa=4.9, we then derive the $\Delta G^{op}{}_p$ values for the hamster protein (Fig. 4a of the main text):

$$\Delta G_{j,p}^{op} = \Delta G_j^{op}(pH5) + RT\ln(1-p_2) \qquad (12)$$

Deriving the values for the unfolding free energies of the neutral and protonated forms of GHaPrP and RaPrP, from urea denaturation experiments carried out at pH4 and pH7.

Urea denaturation curves were measured for GHaPrP and RaPrP , at pH7 and pH4 respectively, following the experimental procedures described in Methods (main text). The raw data are listed in **Table S9**.

Non-linear least squares fits were used to derive the unfolding free energy ΔG of the neutral and protonated forms of the PrP proteins. To this end it was considered that the GHaPrP and RaPrP, solutions at each pH represent a mixture of 2 forms: a neutral form PrP_N , and a protonated form PrP_P corresponding to the forms of PrP in which buried H187/186 is in its neutral and protonated state, respectively. We assume that each of these forms follows a two-state denaturation reaction.

Since the pKa of His 187/186 depends on its environment in the protein, the relative population of the neutral and protonated forms of the His residue, and hence of the PrP protein, change as a function of pH, and of the concentration of denaturant (urea), as the unfolded form of the protein accumulates.

Thus we have:

$$\frac{[N]}{[P]} = 10^{(pH-pK_a)} \qquad (13)$$

Where [N] and [P] are the concentration of the neutral and protonated forms of the His residue and PrP protein, and the pKa is that of the H186/187, residues.

Using Eq (13) we derive the free energy difference between the two protein forms in the folded and unfolded states, respectively:

$$G_N^u - G_P^u = RTln10[pK_a^u - pH] \quad (14)$$
$$G_N^f - G_P^f = RTln10\left[pK_a^f - pH\right]$$

The right hand sides of Eq. (14) represent the free energy difference between the protonated and neutral form of the protein in the unfolded state (top equation) and in the folded state (bottom equation). pKa^f and pKa^u, are respectively, the H186/187 pKa in the folded and unfolded states of the proteins.

It is convenient to express the free energies in terms of the difference relative to the free energy of the neutral folded state:

$$\Delta G_y^x = G_y^x - G_N^f \quad (15)$$

Where the superscript *x* designates the protein state (folded, *f*, or unfolded, *u*), and the subscript *y* designates the protein form (neutral, *N*, or protonated *P*).

The thermodynamic processes linking the free energies of the different states and forms are represented by the thermodynamic cycle of **Fig. S9**

Taking into account the pH dependence of the free energy values (Eq. 14) and their dependence on the urea concentration denoted as C, where the m_y^x, and m_y values represent the slopes of this dependence) we have the following relationships:

$$\Delta G_N^f = 0$$

$$\Delta G_N^u(C) = \Delta G_N^{unfolding} - m_N^u C$$

$$\Delta G_P^f(C,pH) = RTln10\left(pH - pK_a^f\right) - m_P^f C \qquad (16)$$

$$\Delta G_p^u(C,pH) = \Delta G_p^{unfolding} + RT\,ln10\,(pH - pK_a^f) - m_P^u C$$

Where $\Delta G_N^{unfolding}$ and $\Delta G_P^{unfolding}$ are the unfolding free energies of the neutral and protonated form of PrP respectively, with $\Delta G_N^{unfolding} = \Delta G_N^u\,(H2O)$, and (H20) indicates zero urea concentration (see Fig. S9). In the main text $\Delta G_N^{unfolding}$ and $\Delta G_P^{unfolding}$ are denoted as ΔG_N and ΔG_P .

The measured response (ellipticity at 220 nm) as a function of pH and urea concentration can then be expressed as an ensemble average of the responses of the different forms and states of the protein:

$$A(C,pH) = \frac{1}{Q(C,pH)}\begin{Bmatrix} A_N^f(C)e^{-\frac{1}{RT}\Delta G_N^f} + A_N^u(C)e^{-\frac{1}{RT}\Delta G_N^u(C)} \\ +A_P^f(C)e^{-\frac{1}{RT}\Delta G_P^f(C,pH)} + A_P^u(C)e^{-\frac{1}{RT}\Delta G_P^u(C,pH)} \end{Bmatrix} \qquad (17)$$

$$Q(C,pH) = e^{-\frac{1}{RT}\Delta G_N^f} + e^{-\frac{1}{RT}\Delta G_N^u(C)} + e^{-\frac{1}{RT}\Delta G_P^f(C,pH)} + e^{-\frac{1}{RT}\Delta G_P^u(C,pH)}$$

The fit was performed using F_{app} *(C, pH):*

$$F_{app}(C,pH) = \frac{A(C,pH)}{A(0,pH)} \qquad (18)$$

Where *A(C,pH) and A(0,pH)* are the measured responses as a function of urea concentration *C (and in absence of urea)* and *pH*, using the following additional 2 assumptions and settings:

1-The measured response (ellipticity) for the different states and forms varies linearly with urea concentration:

$$A_y^x\,(C) = A_y^x(H2O) + R_y^x C \qquad (19)$$

Where the meaning of x and y is the same as in Eq. (15), C is the urea concentration and R_y^x is the slope of the measured ellipticity as a function of urea concentration.

2-The protonated and neutral forms of the protein give rise to the same response when in the native, or the unfolded state, respectively.

$$A_p^f\,(C) = A_N^f\,(C)\,; \qquad (20)$$

$$A_p^u\,(C) = A_N^u\,(C)$$

3- The pKa of the neutral form was set to the experimental values of 5.1 for RaPrP and 4.9 for GHaPrP.

On the basis of the above equations and assumptions the values of the following 7 parameters were derived by least squares fit of the expression in Eq. (18) (detailed in Eq. 16) to the experimental data for the two PrP proteins measured at pH 4 and pH7, respectively:

$$\Delta G_N^{unfolding} = \Delta G_N^u(H2O)\,;\ \Delta G_P^{unfolding};\ m_{N,p}^{u,f}\,;\ A^f, A^u;\ R^f, R^u$$

$$with\ m_N^f = 0\ ;\ \ m_P^u = \ m_N^u + m_P^f;\ \ A^f + A^u = 1; \qquad (21)$$

The resulting fit is displayed in **Fig. 5** of the main text, and the values of the various parameters are listed in **Table S10** and **Table 2** of the main text.

Estimating the $\Delta\Delta G^{op}{}_j$ values as a function of pH, and His187 pKa:

We use the well-known definitions of the pH and *pKa*, we estimate the $\Delta\Delta G^{op}{}_j$ values as a function of pH solely on the basis of the measured His187 pKa values as follows:

$$pH = -log_{10}[H^+];\ \ pK_a = \ log_{10}\frac{[His^+]}{[His][H^+]} \quad (22)$$

where [H^+] stands for the proton concentration, and *[His^+]* and *[His]* stand for the concentrations (populations) of protonated and neutral His 187 species, respectively, we derive the fraction of the population with protonated His residues as a function of pH:

$$P_{His^+}(pH) = \frac{1}{1+\ 10^{(pH-pKa)}} \quad (23)$$

Assuming that exchange occurs only through the *His^+* sub-state (**Fig. 3a-b)** we have:

$$P_{His^+}e^{\frac{-\Delta G^{op}_{His^+}}{RT}} \gg P_{His}e^{\frac{-\Delta G^{op}_{His}}{RT}} \ (24)$$

The ratio of the populations with *[His^+]*, at pH 5 and pH 7, can then be obtained from Eq. (23), and the corresponding free energy difference can be directly calculated from Eq. (7) of the main text, as follows:

$$\Delta G_j^{op}(pH7) - \Delta G_j^{op}(pH5) = \Delta\Delta G_j^{op} = RTln\ \frac{1+10^{(7-pKa)}}{1+10^{(5-pKa)}} \quad (25)$$

Replacing the His pKa by the measured experimental value (pKa=4.94 ±0.2) in

Eq. (25) (identical to Eq. (2) in the main text) yields the following estimate for $\Delta\Delta G_j^{op}$:

$$\Delta\Delta G_j^{op} = 2.35 \pm 0.15\; kcal/mol \qquad (26)$$

Eq. (26) is identical to Eq. (3) in the main text.

Possible role of acidic residues protonation

As stated in the text we did not examine the role of acidic residues (Glu and Asp). Inspection of the rabbit and hamster PrP solution structures indicates that none of the 13 acidic residues were sufficiently shielded from solvent in the NMR conformational ensembles to feature a shifted pKa. This suggests in turn that protonation of these residues would not destabilize the monomeric PrP fold. The role of the other 4 His residues of the considered PrP domain (residue 90-231) was not considered for the same reason.

A recent study (Singh & Udgaonkar, 2016) finds indeed, that the pH-induced misfolding transition of H186F moPrP (a stabilizing mutation, of the single buried His residue in PrP) has an apparent p**K**a of ~3.8. This pKa value is virtually identical to the pKa of solvated acidic residues, confirming our initial conclusion that the pKa of these residues is not shifted in the folded protein. Yet, rather surprisingly, the same study also shows that two single mutants of mouse PrP D177N and D201N, which mimic the neutral form of the Asp side chains, are essentially misfolded over a wide pH range (pH 2-6). The large apparent effect of the D177N and D201N mutation on PrP misfolding is therefore intriguing. High salt conditions are known

to foster PrP oligomerization in denaturation experiments as mentioned in the main text, likely by screening net charges. The D->N replacements may act similarly, and their effect may hence stabilize PrP oligomers rather than destabilize the monomer.

In any case, the observed behavior of the asp mutants does not apply to the *wt* Asp residues. Their protonated from will be significantly populated only at low pH (<5), but remains too low (≤0.1%, according to Eq. (23)) to contribute to the unfolding reaction of monomeric PrP at neutral pH. Asp protonation should also marginally affect the stability of monomeric PrP, measured at low pH, and instead significantly stabilize the oligomeric form, thereby depleting the monomeric form and driving the aggregation reaction.

References

Zhang Y.-Z. (1995). Protein and peptide structure and interactions studied by hydrogen exchange and NMR. Ph.D. thesis University of Pennsylvania.

Hansen DF. Yang D. Feng H. Zhou Z. Wiesner S. Bai Y. Kay LE. (2007), An exchange-free measure of 15N transverse relaxation: an NMR spectroscopy application to the study of a folding intermediate with pervasive chemical exchange, J Am Chem Soc. 129(37):11468-79.

Pace CN, Shaw KL. (2000),Linear extrapolation method of analyzing solvent denaturation curves, Proteins. ;Suppl 4:1-7 (PMID: 11013396).

Singh J, Udgaonkar JB. Unraveling the Molecular Mechanism of pH-Induced Misfolding and Oligomerization of the Prion Protein. J Mol Biol 2016;428(6):1345-1355.

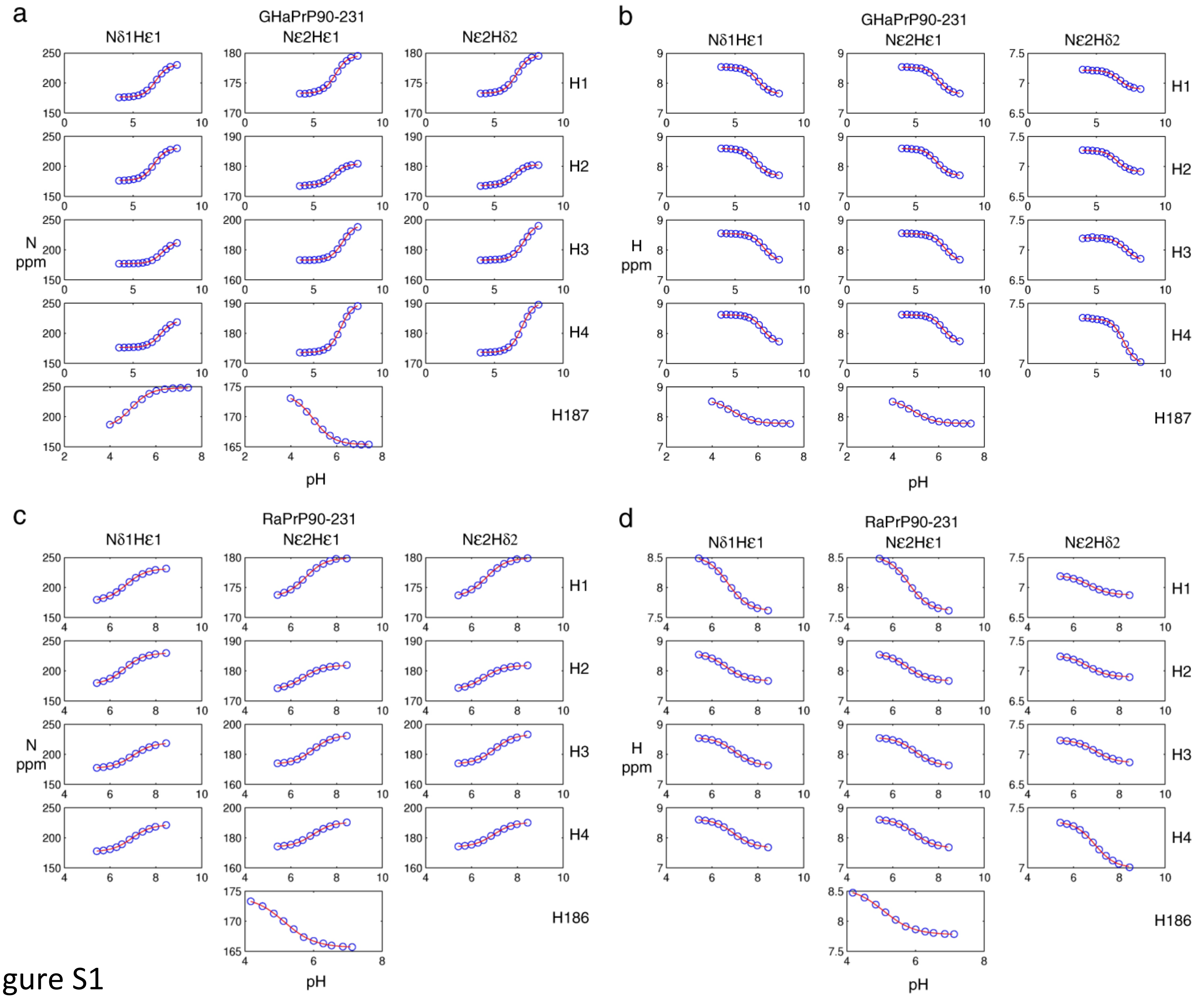
a
GHaPrP90-231
Nδ1Hε1
Nε2Hε1
Nε2Hδ2
H1
H2
H3
H4
H187
N ppm
pH
b
GHaPrP90-231
Nδ1Hε1
Nε2Hε1
Nε2Hδ2
H1
H2
H3
H4
H187
H ppm
pH
c
RaPrP90-231
Nδ1Hε1
Nε2Hε1
Nε2Hδ2
H1
H2
H3
H4
H186
N ppm
pH
d
RaPrP90-231
Nδ1Hε1
Nε2Hε1
Nε2Hδ2
H1
H2
H3
H4
H186
H ppm
pH

Figure S1

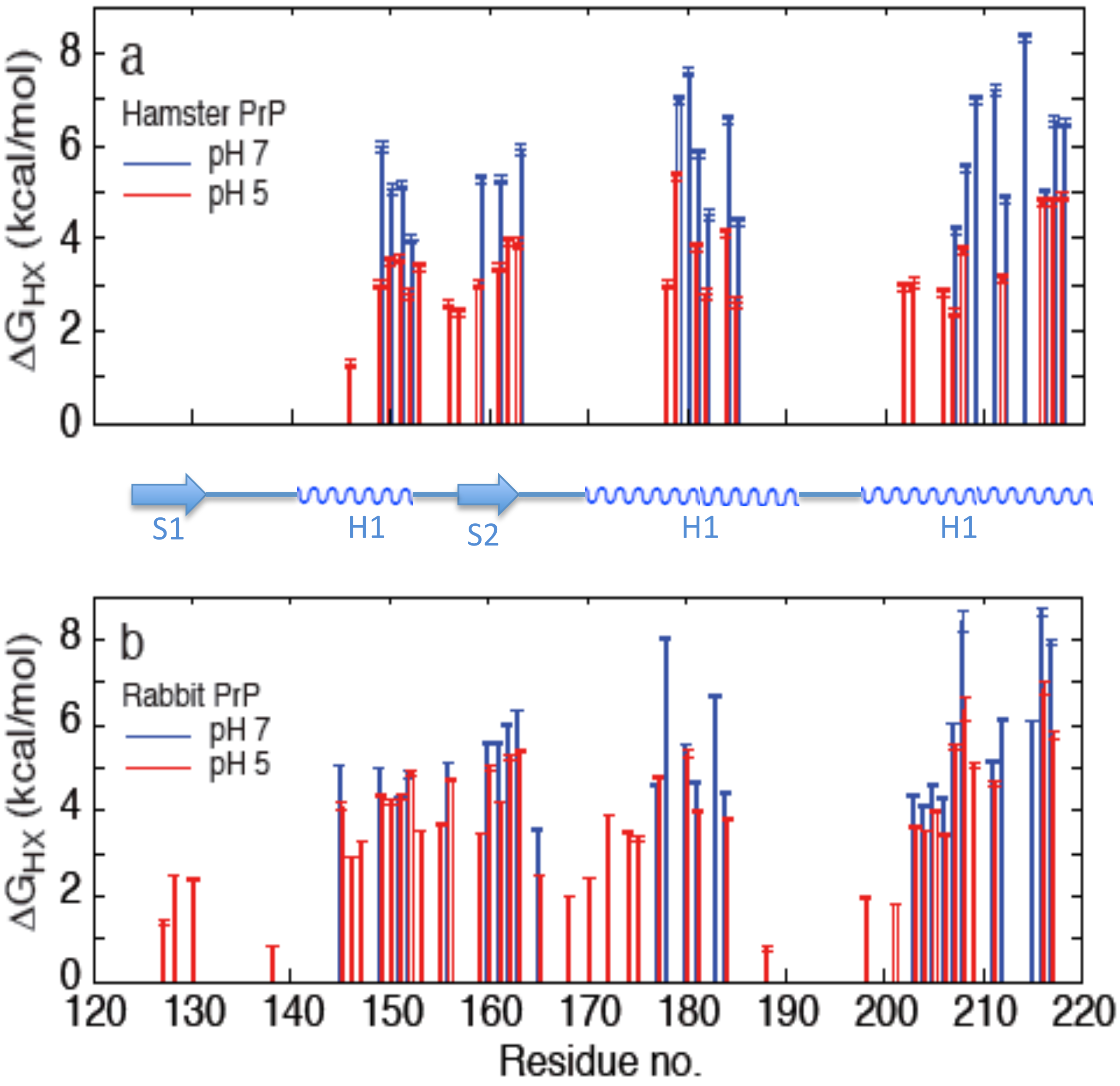
a
Hamster PrP
pH 7
pH 5
ΔG$_{HX}$ (kcal/mol)
S1
H1
S2
H1
H1
b
Rabbit PrP
pH 7
pH 5
ΔG$_{HX}$ (kcal/mol)
Residue no.

Figure S2

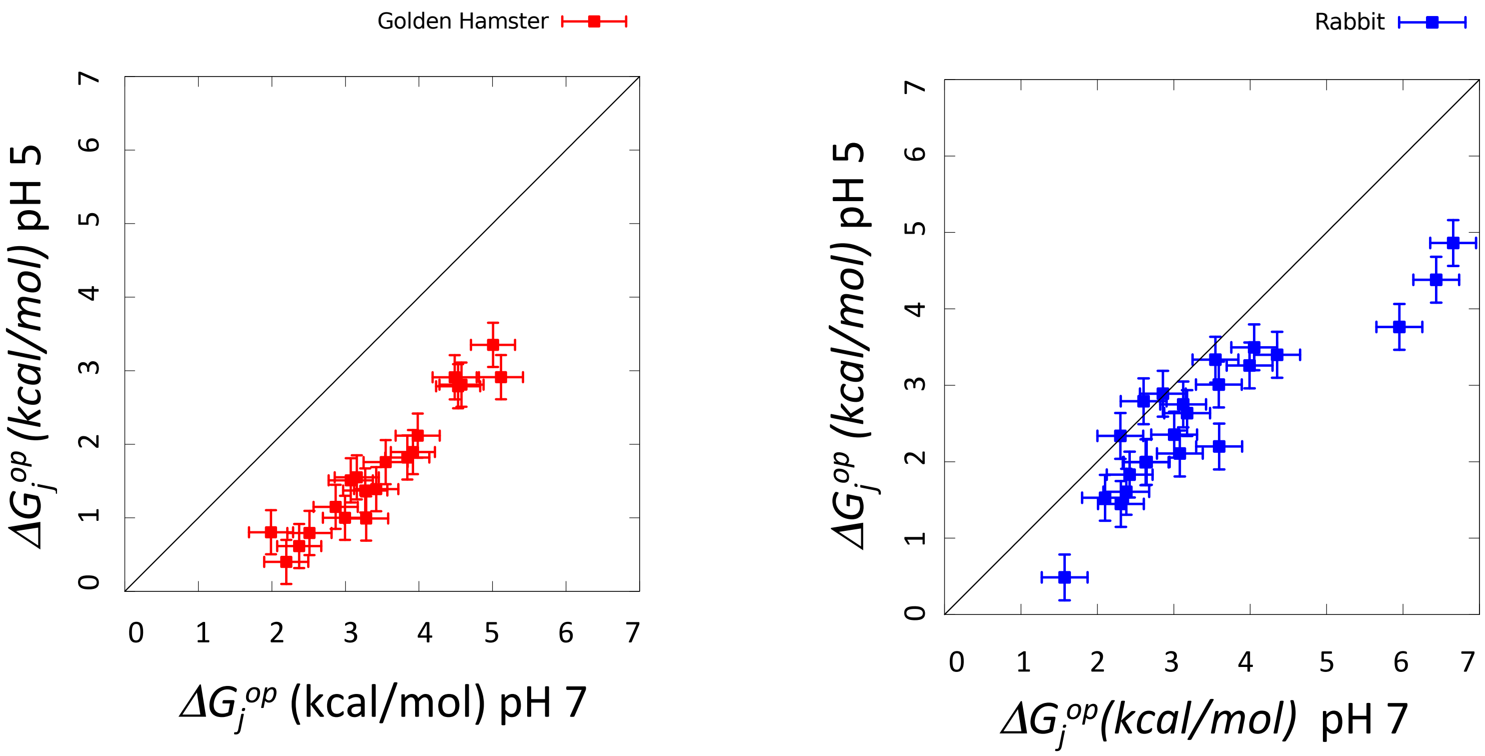

Golden Hamster
ΔG_j^op (kcal/mol) pH 5
ΔG_j^op (kcal/mol) pH 7
0 1 2 3 4 5 6 7
Rabbit
ΔG_j^op (kcal/mol) pH 5
ΔG_j^op(kcal/mol) pH 7
0 1 2 3 4 5 6 7

Figure S3

Figures S4 – S7 are provided as separate supplementary files

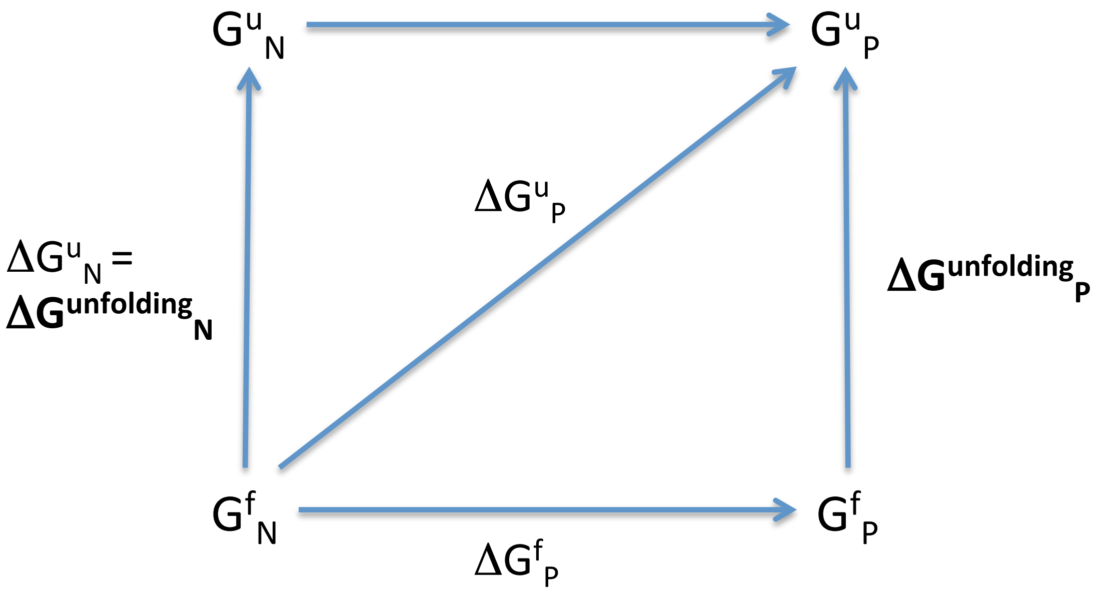
G^u_N
G^u_P
ΔG^u_P
$\Delta G^u_N =$
$\mathbf{\Delta G^{unfolding}_N}$
$\mathbf{\Delta G^{unfolding}_P}$
G^f_N
G^f_P
ΔG^f_P

Figure S8

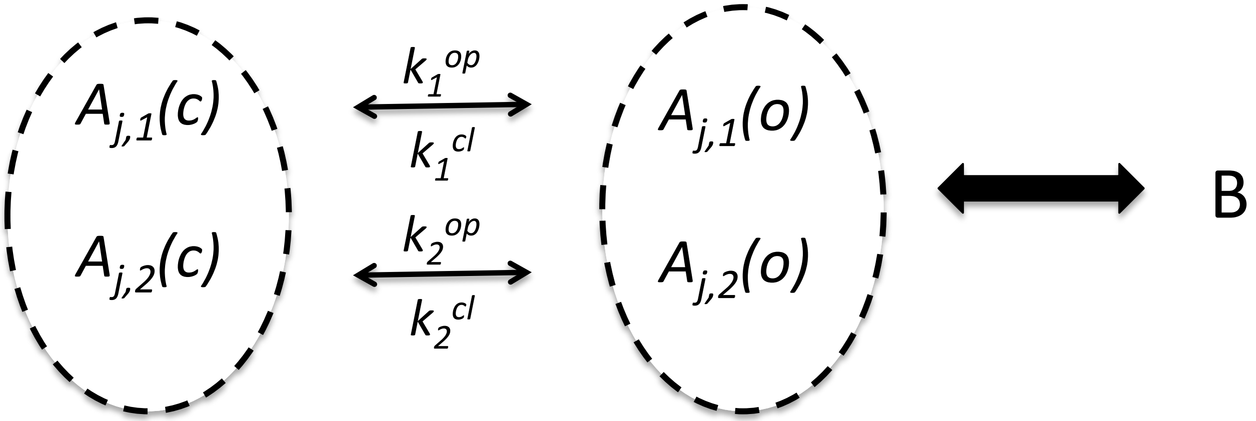

Fig. S9
