# Supplementary material for: Interplay of buried histidine protonation and protein stability in prion misfolding
Source: Sci Rep. 2017 Apr 13;7:882. doi: 10.1038/s41598-017-00954-7 (PMC5429843; doi:10.1038/s41598-017-00954-7)
Supplement: Supplementary file 3 — Supplenetary Figure S5 [file 41598_2017_954_MOESM3_ESM.pdf]

Interplay of buried histidine protonation and protein stability in prion misfolding

Supplementary Figure S5

A. Malevanets[1], P. Andrew Chong[1,2], D.F. Hansen[2,3,+], P. Rizk[1,2], Y. Sun[4], H. Lin[1], R. Muhandiram[2,3], A. Chakrabartty[4], Lewis E. Kay[1,2,3,5], J.D. Forman-Kay[1,2,**] and Shoshana J. Wodak[1,2,3§**]

[1]Program in Molecular Structure and Function, Hospital for Sick Children, 555 University Ave., Toronto, ON, Canada M5G 0A4

[2]Department of Biochemistry and [3]Department of Molecular Genetics, University of Toronto, Toronto, ON, Canada M5S 1A8

[4]Department of Medical Biophysics, University of Toronto, Toronto, ON, Canada M5G 2M9

[5]Department of Chemistry, University of Toronto, Toronto, ON, Canada, M5S 3H6

[+]Current address: University College London, Division of Biosciences, London WC1E 6BT UK

[§]Current address: VIB Structural Biology Research Center, VUB, Pleinlaan 2 1050 Brussels, Belgium

** Corresponding authors.

Figures S5. Hydrogen exchange decay curves for hamster PrP at pH 5.

Decay curves of peak intensities in 15N-1H correlation maps (O) vertical bars represent the uncertainty of the obtained intensity. The intensities were obtained from a simultaneous analysis and global fit of all spectra with different tau as described previously (Hansen et al (2007)). The solid lines are obtained from best-fits of an exponential decay function I(t)=I0*exp(-k_{ex}*tau) + offset to the data shown. For peaks with a decay rate slower than ~1e-5 /s the offset and the k_{ex} rate are strongly correlated and the offset was therefore fixed to 0 for these peaks.

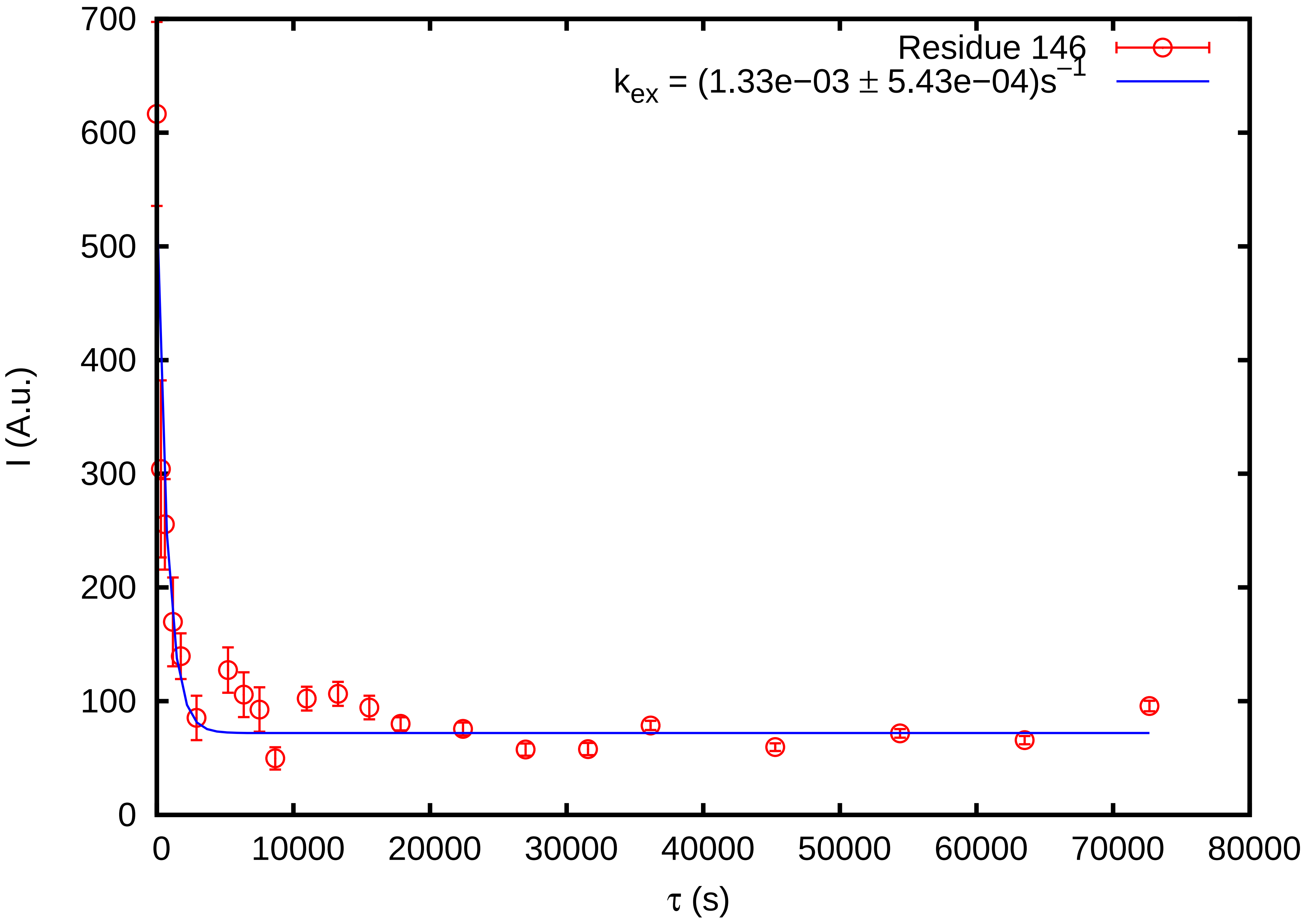

Residue 146
k_{ex} = (1.33e−03 ± 5.43e−04)s^{-1}
I (A.u.)
τ (s)
0
100
200
300
400
500
600
700
0
10000
20000
30000
40000
50000
60000
70000
80000

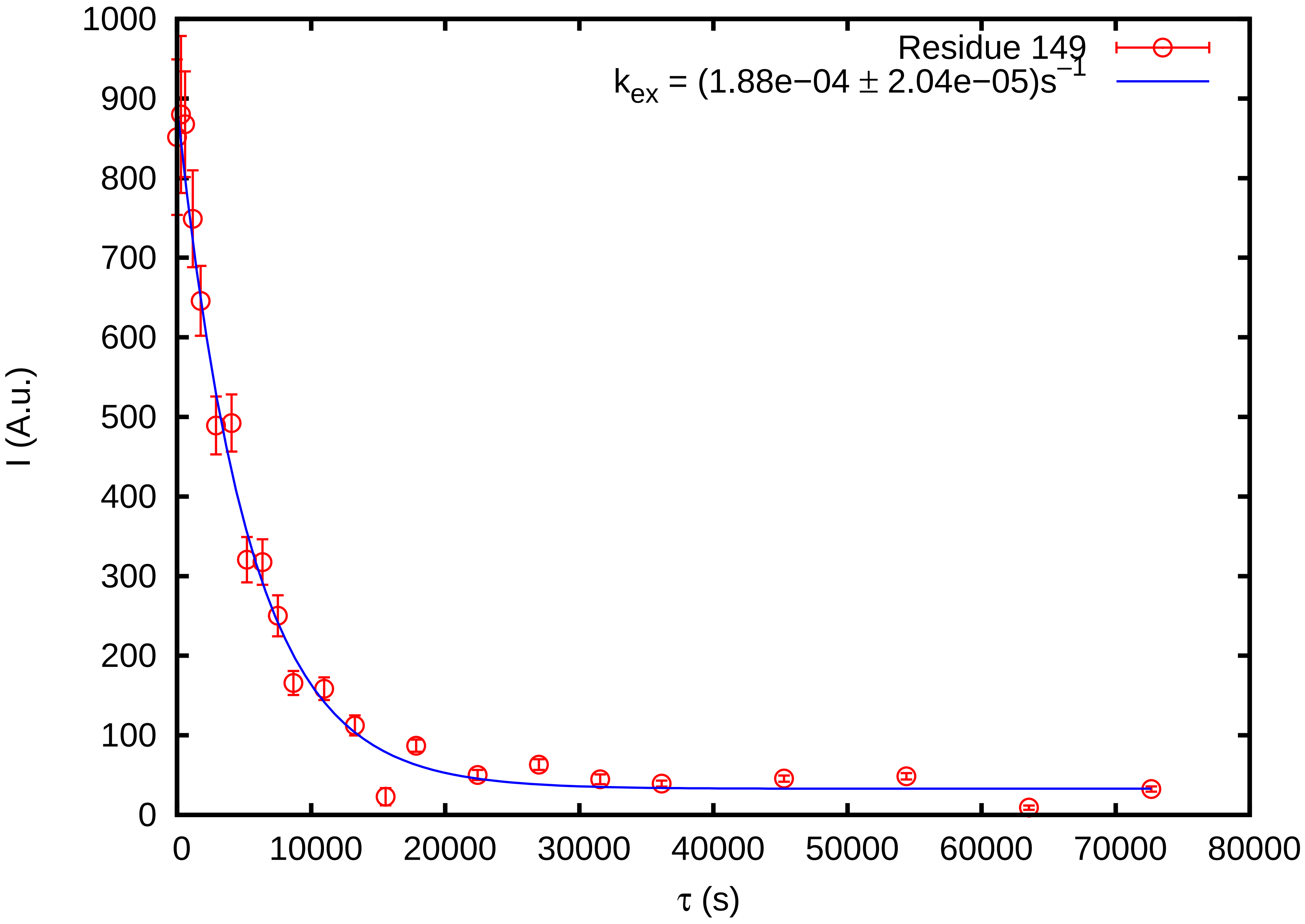

Residue 149
k_ex = (1.88e−04 ± 2.04e−05)s^−1
I (A.u.)
τ (s)
0
100
200
300
400
500
600
700
800
900
1000
10000
20000
30000
40000
50000
60000
70000
80000

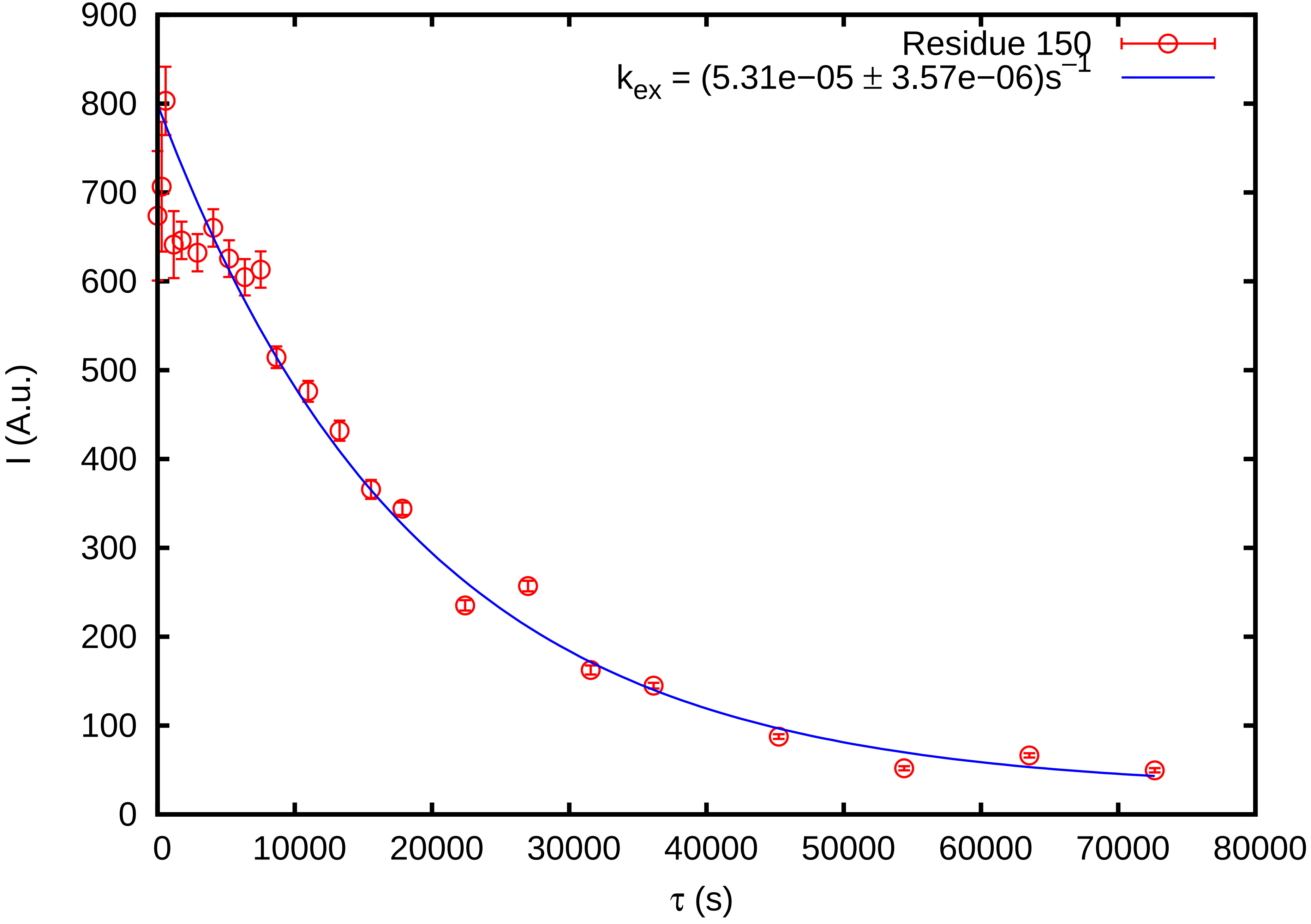

Residue 150
k_{ex} = (5.31e−05 ± 3.57e−06)s^{-1}
900
800
700
600
500
400
300
200
100
0
I (A.u.)
0
10000
20000
30000
40000
50000
60000
70000
80000
τ (s)

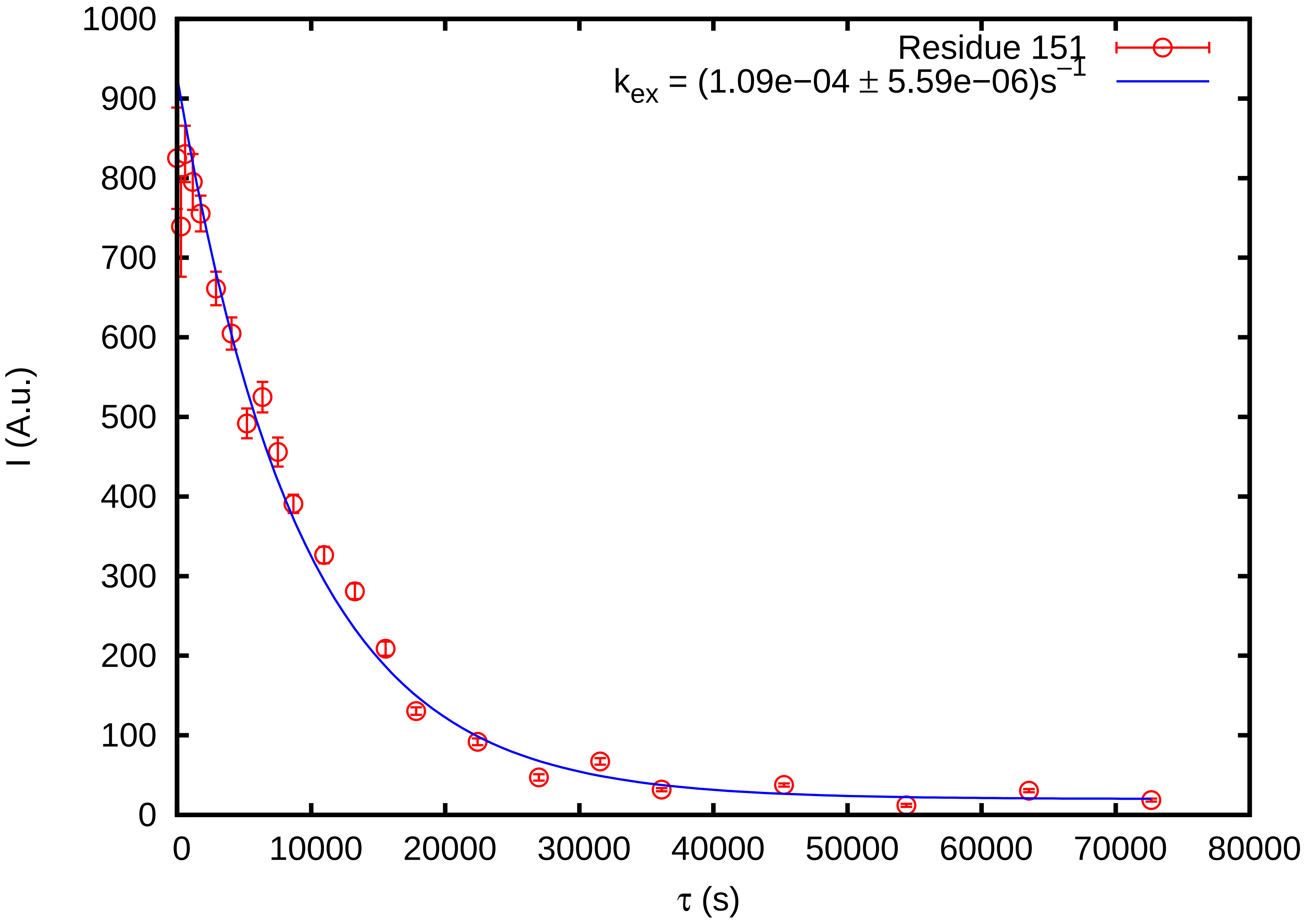

Residue 151
k_ex = (1.09e−04 ± 5.59e−06)s^−1
I (A.u.)
τ (s)
0
100
200
300
400
500
600
700
800
900
1000
0
10000
20000
30000
40000
50000
60000
70000
80000

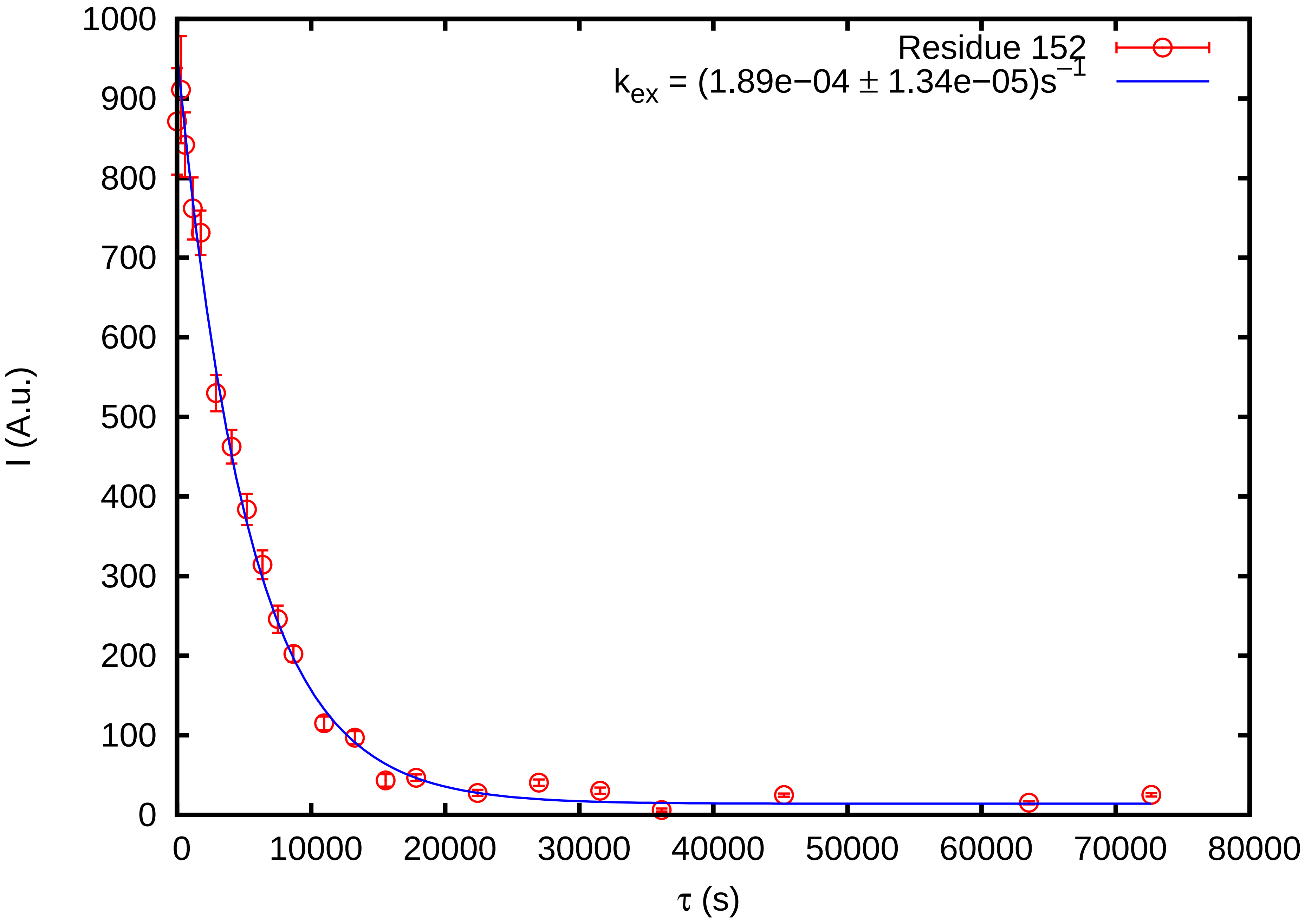

Residue 152
k_ex = (1.89e−04 ± 1.34e−05)s^−1
I (A.u.)
τ (s)
0
100
200
300
400
500
600
700
800
900
1000
0
10000
20000
30000
40000
50000
60000
70000
80000

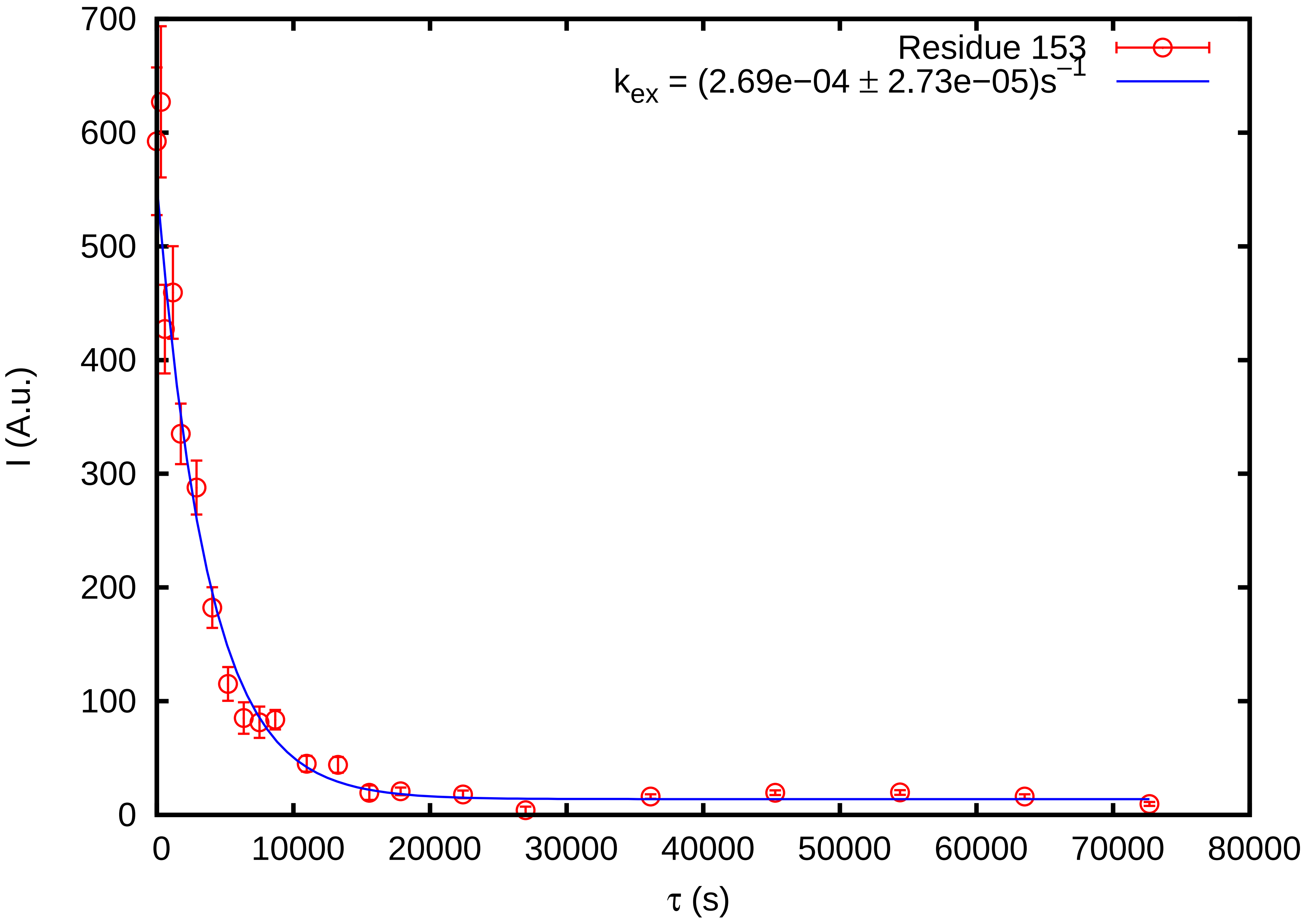

Residue 153
k_{ex} = (2.69e−04 ± 2.73e−05)s^{-1}
I (A.u.)
τ (s)
0
100
200
300
400
500
600
700
0
10000
20000
30000
40000
50000
60000
70000
80000

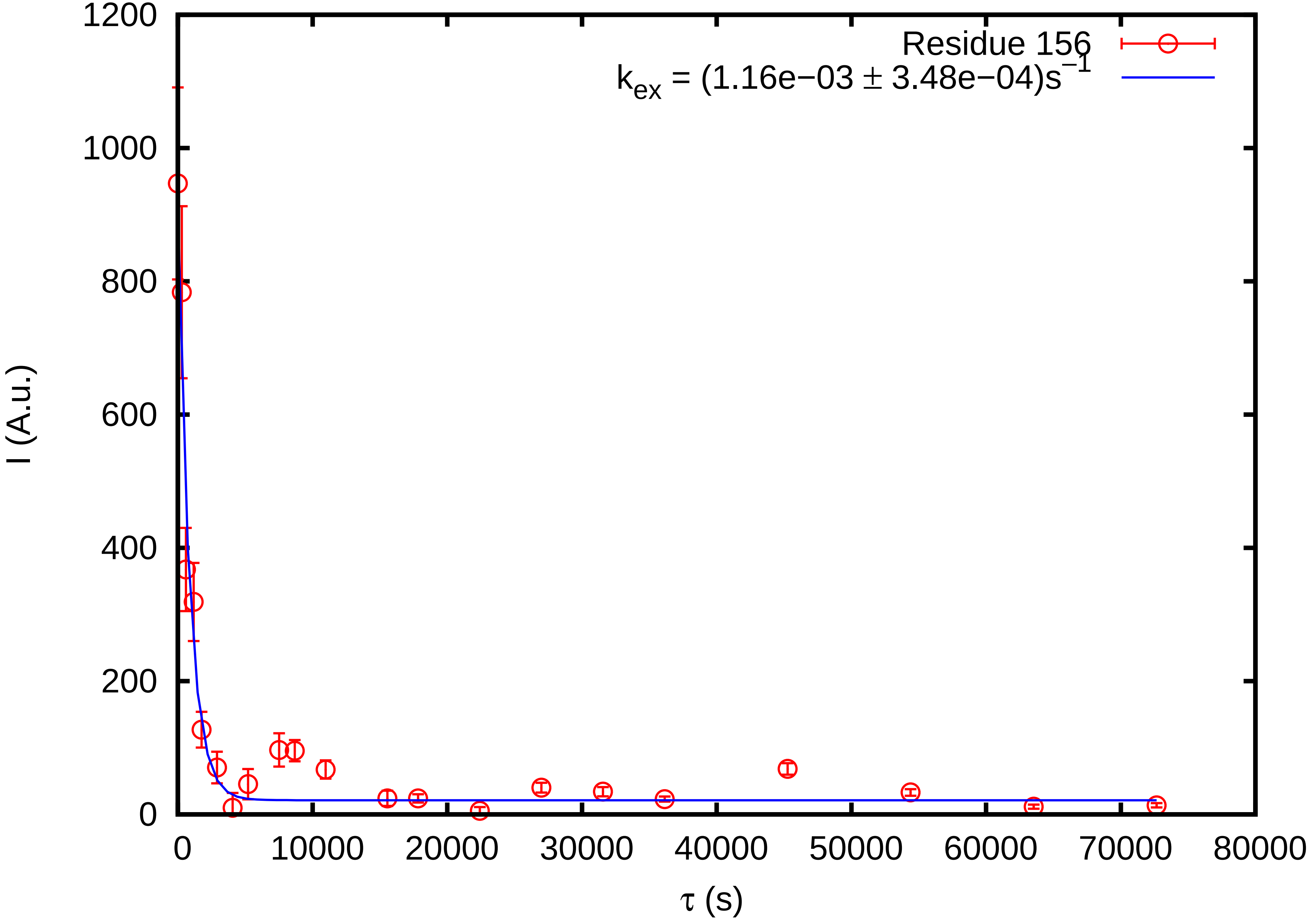

Residue 156
k_ex = (1.16e−03 ± 3.48e−04)s^−1
I (A.u.)
τ (s)
0
200
400
600
800
1000
1200
0
10000
20000
30000
40000
50000
60000
70000
80000

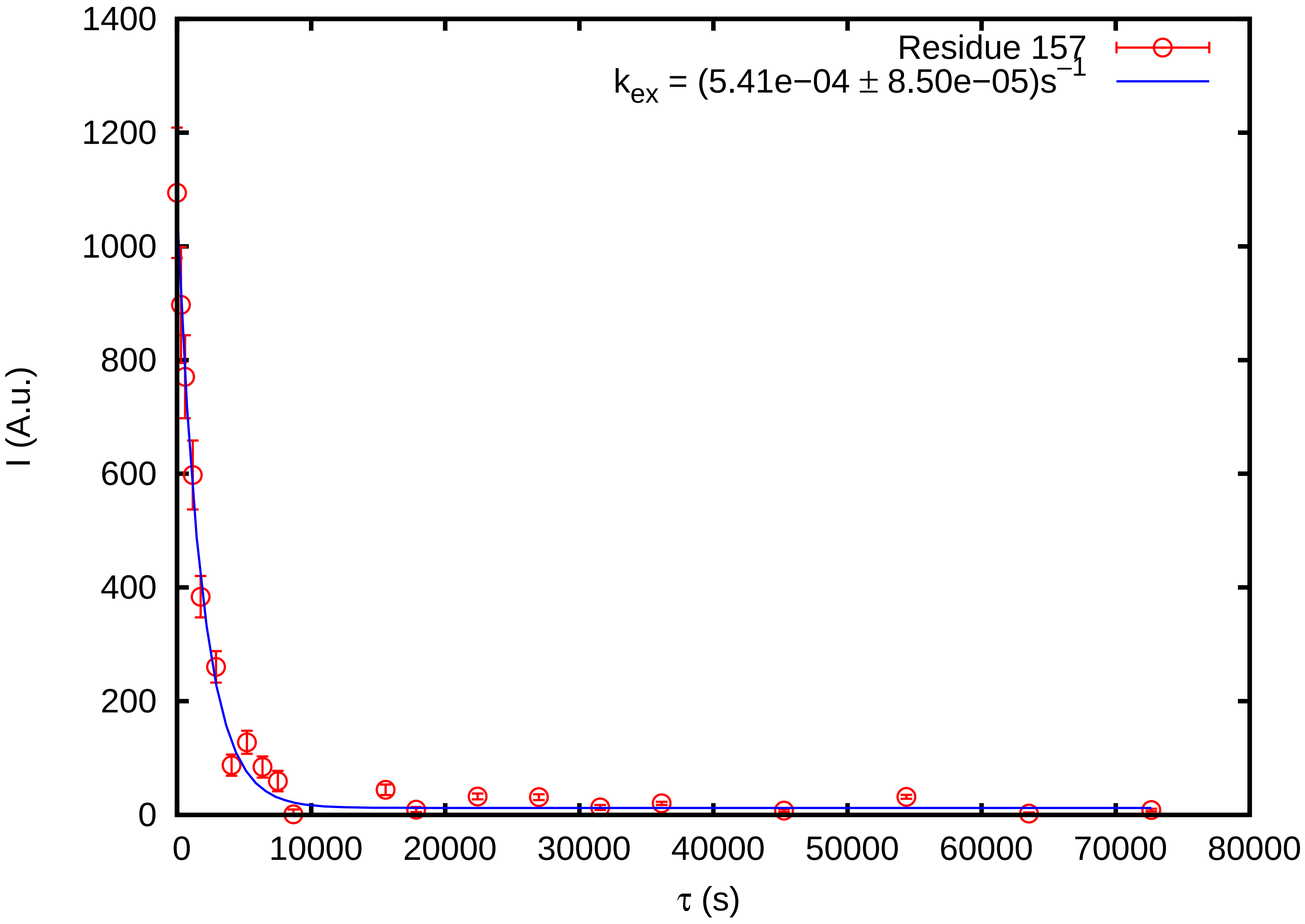

Residue 157
k_ex = (5.41e−04 ± 8.50e−05)s^−1
I (A.u.)
τ (s)
0
200
400
600
800
1000
1200
1400
0
10000
20000
30000
40000
50000
60000
70000
80000

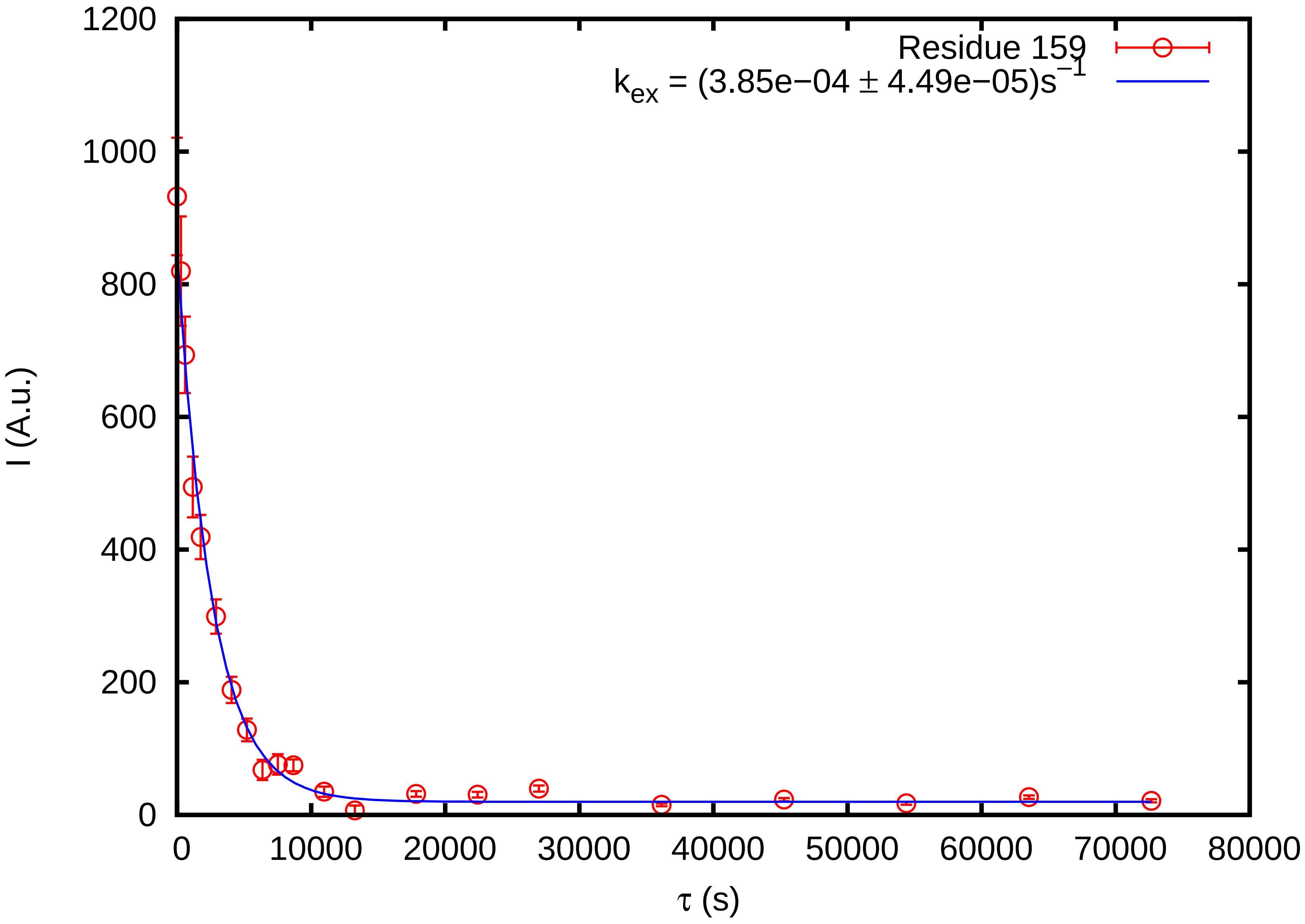

Residue 159
k_ex = (3.85e−04 ± 4.49e−05)s^−1
I (A.u.)
τ (s)
0
200
400
600
800
1000
1200
0
10000
20000
30000
40000
50000
60000
70000
80000

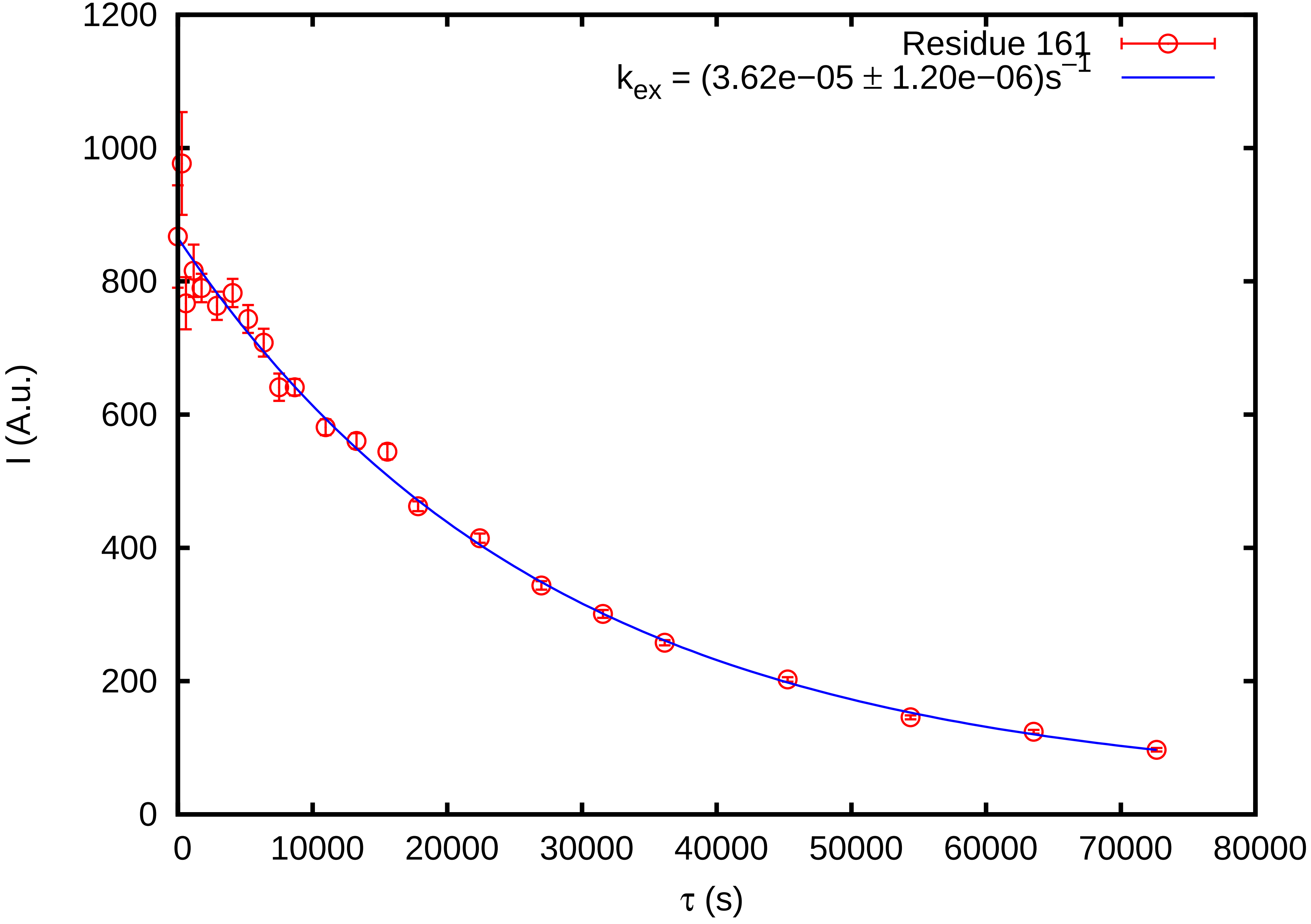

Residue 161
k_{ex} = (3.62e−05 ± 1.20e−06)s^{-1}
I (A.u.)
τ (s)
0
200
400
600
800
1000
1200
0
10000
20000
30000
40000
50000
60000
70000
80000

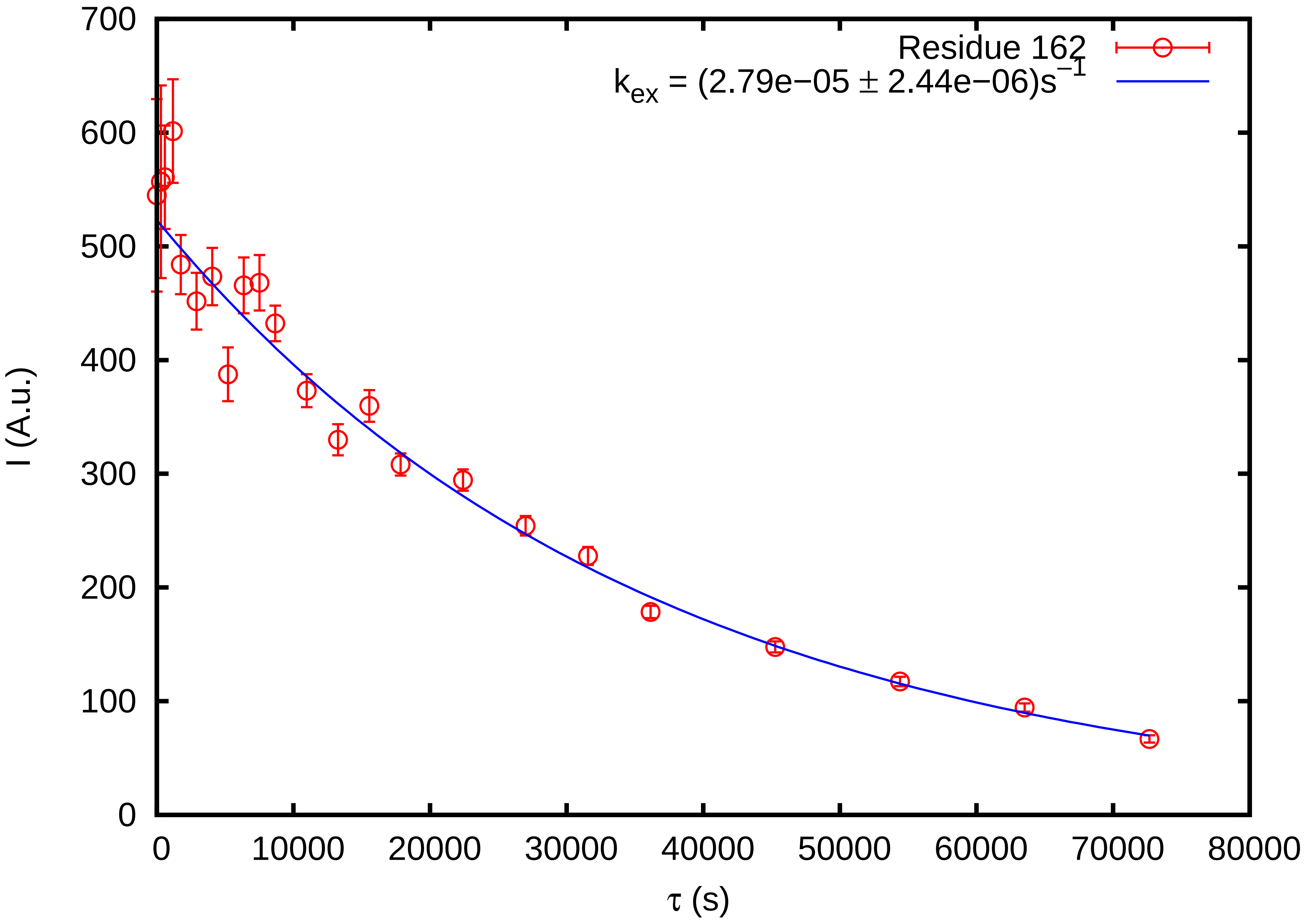

Residue 162
k_{ex} = (2.79e−05 ± 2.44e−06)s^{-1}
I (A.u.)
τ (s)
0
100
200
300
400
500
600
700
0
10000
20000
30000
40000
50000
60000
70000
80000

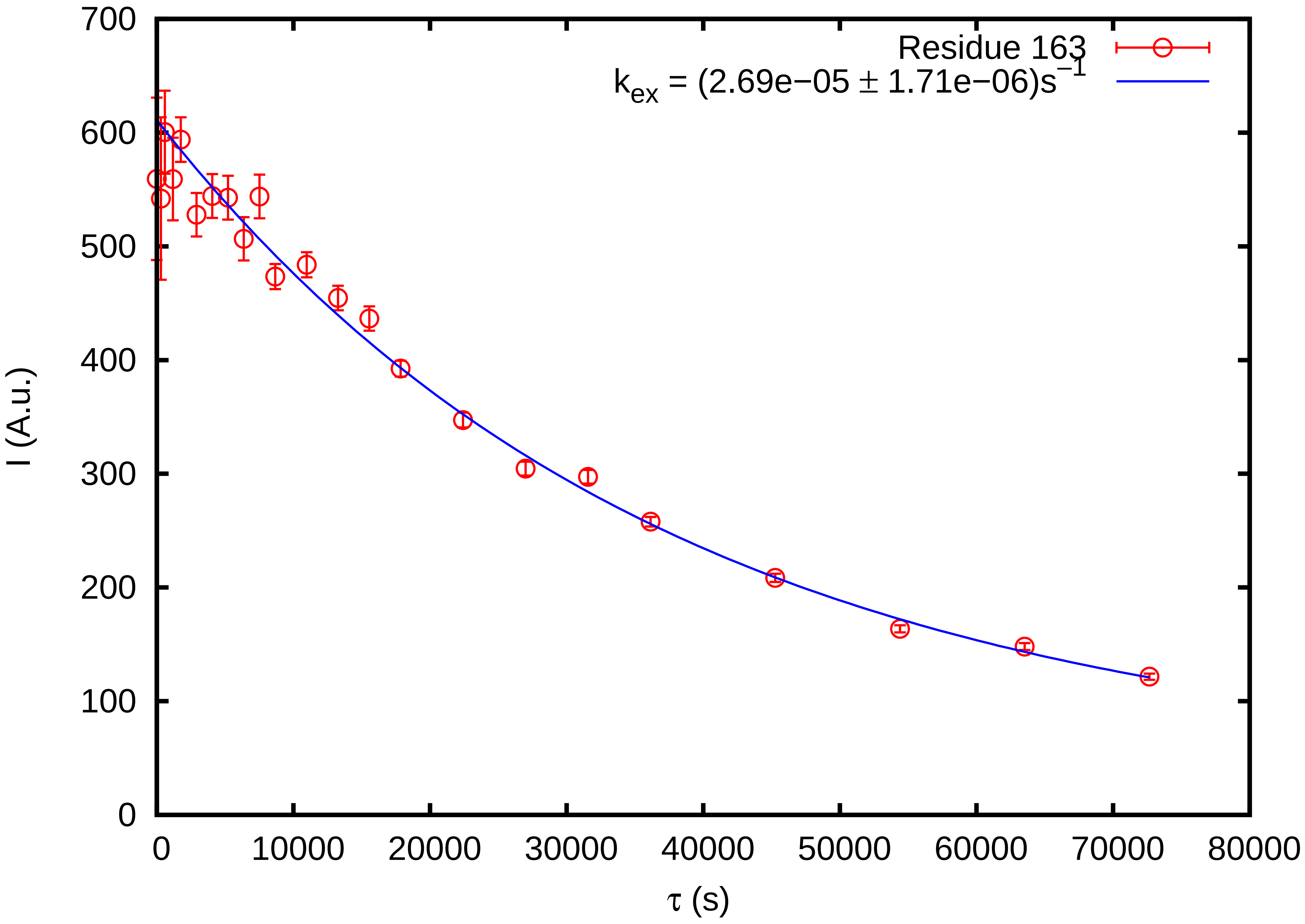

Residue 163
k_{ex} = (2.69e−05 ± 1.71e−06)s^{-1}
I (A.u.)
τ (s)
0
100
200
300
400
500
600
700
0
10000
20000
30000
40000
50000
60000
70000
80000

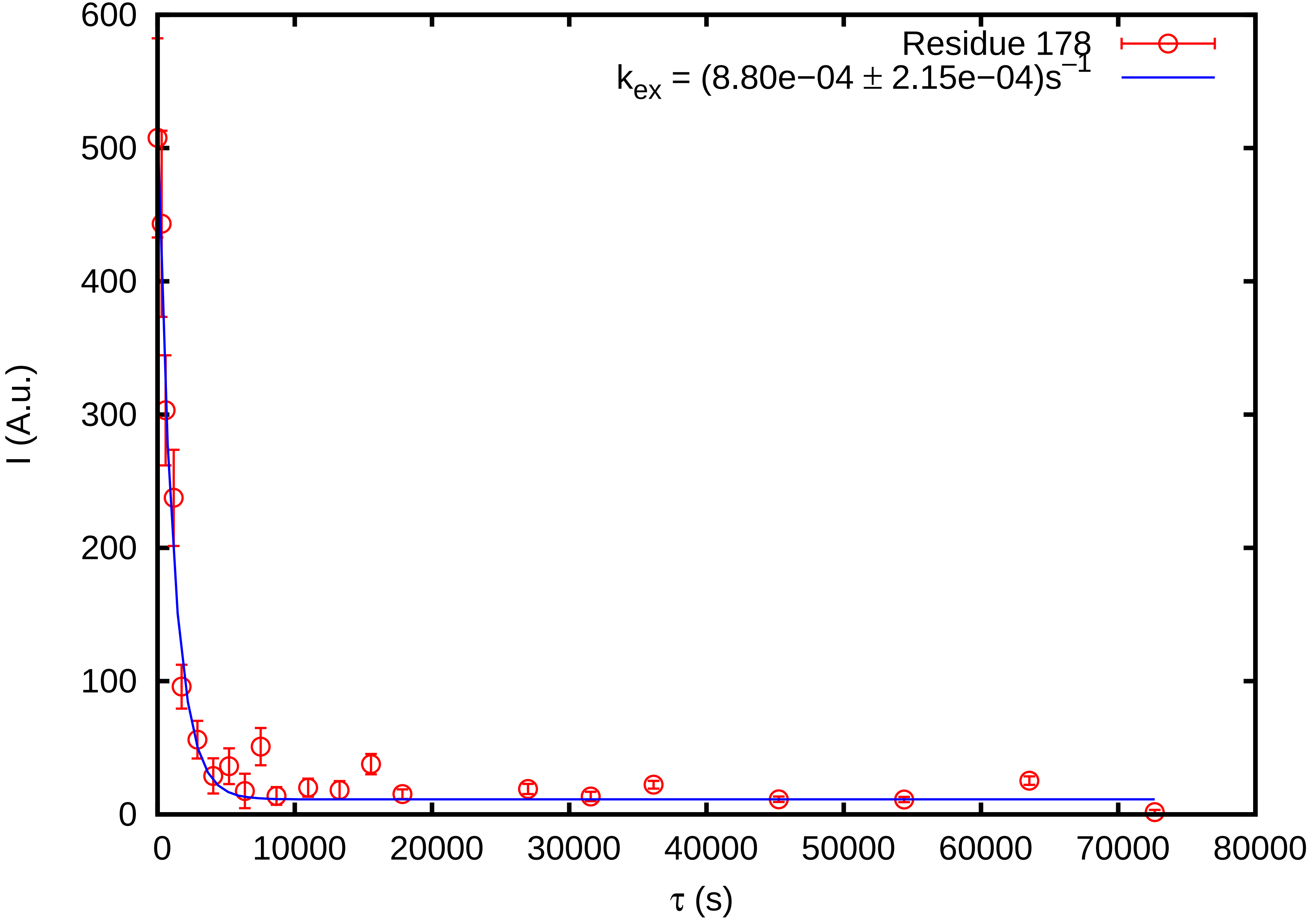

Residue 178
k_{ex} = (8.80e−04 ± 2.15e−04)s^{-1}
I (A.u.)
τ (s)
0
100
200
300
400
500
600
0
10000
20000
30000
40000
50000
60000
70000
80000

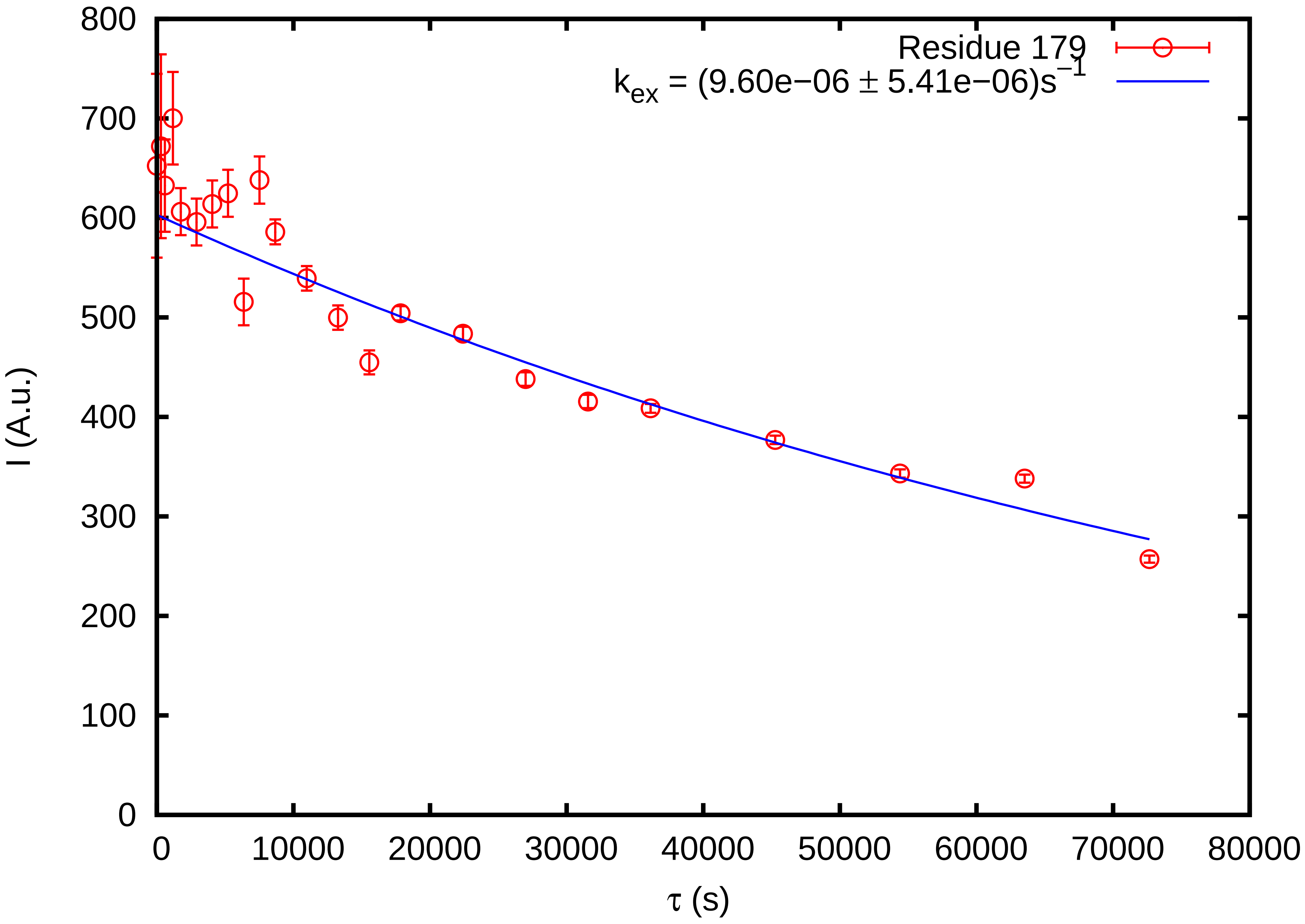

Residue 179
k_{ex} = (9.60e−06 ± 5.41e−06)s^{-1}
I (A.u.)
τ (s)
0
100
200
300
400
500
600
700
800
0
10000
20000
30000
40000
50000
60000
70000
80000

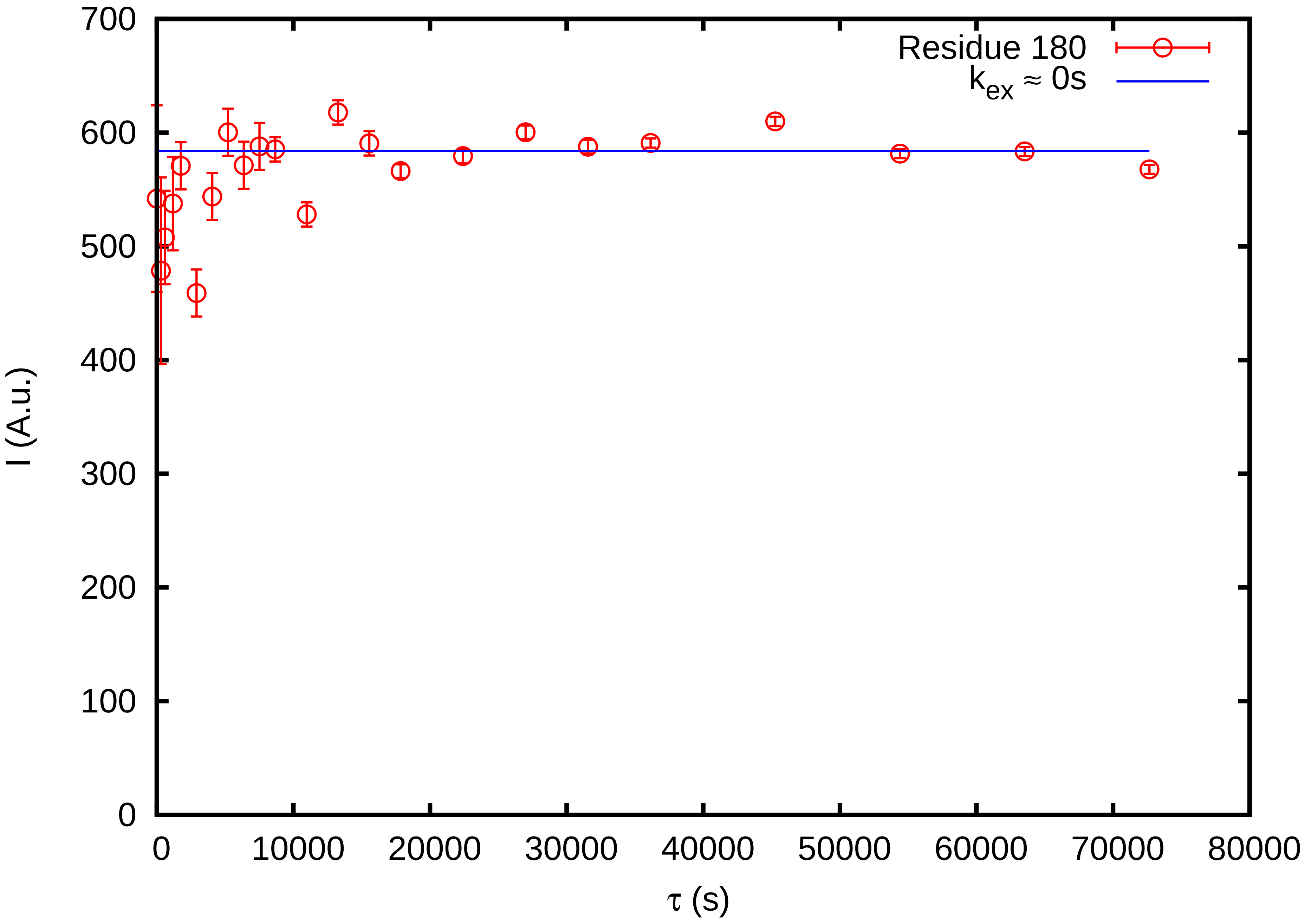

Residue 180
$k_{ex} \approx 0s$
I (A.u.)
τ (s)
0
100
200
300
400
500
600
700
0
10000
20000
30000
40000
50000
60000
70000
80000

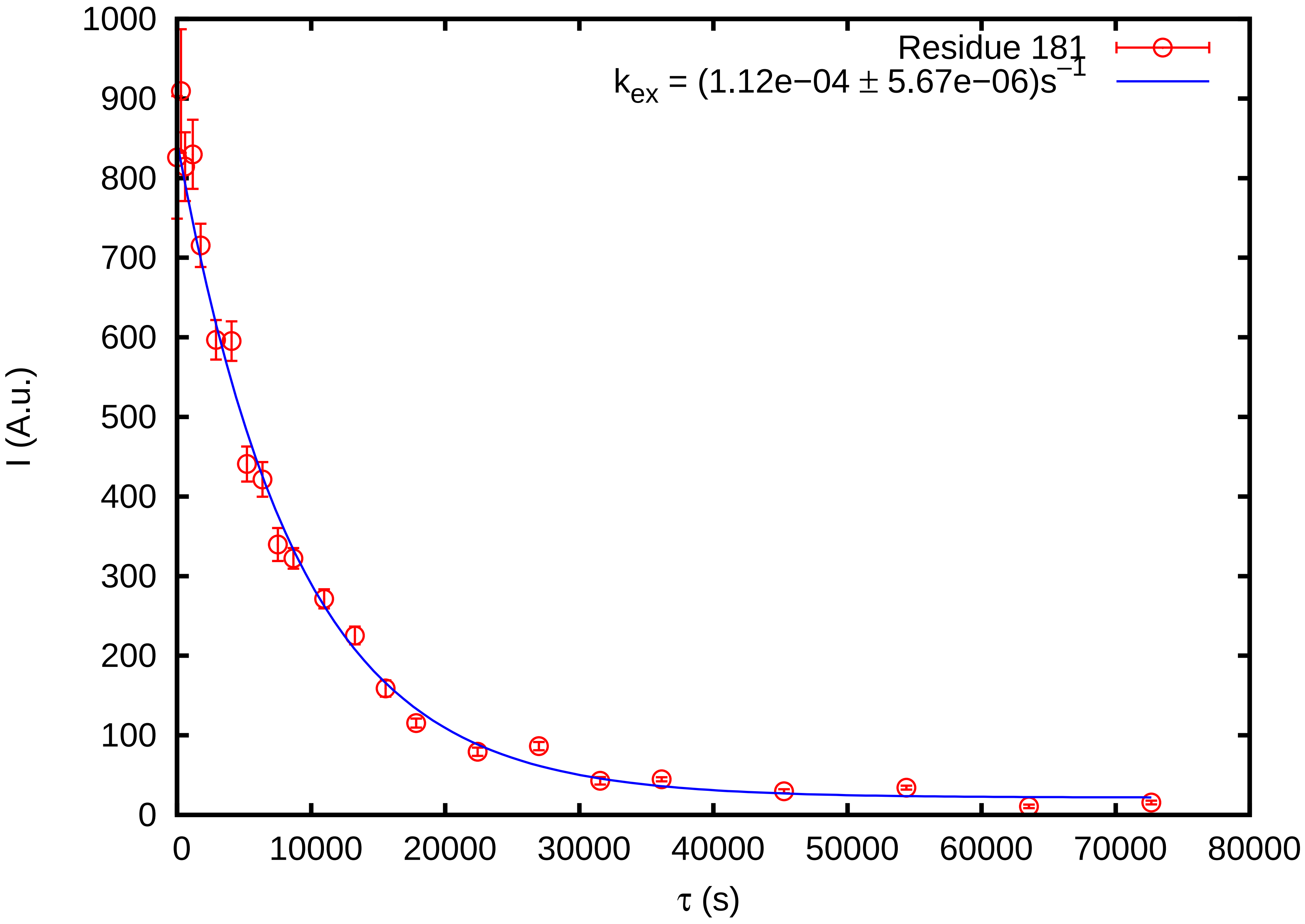

Residue 181
k_{ex} = (1.12e−04 ± 5.67e−06)s^{-1}
I (A.u.)
τ (s)
0
100
200
300
400
500
600
700
800
900
1000
10000
20000
30000
40000
50000
60000
70000
80000

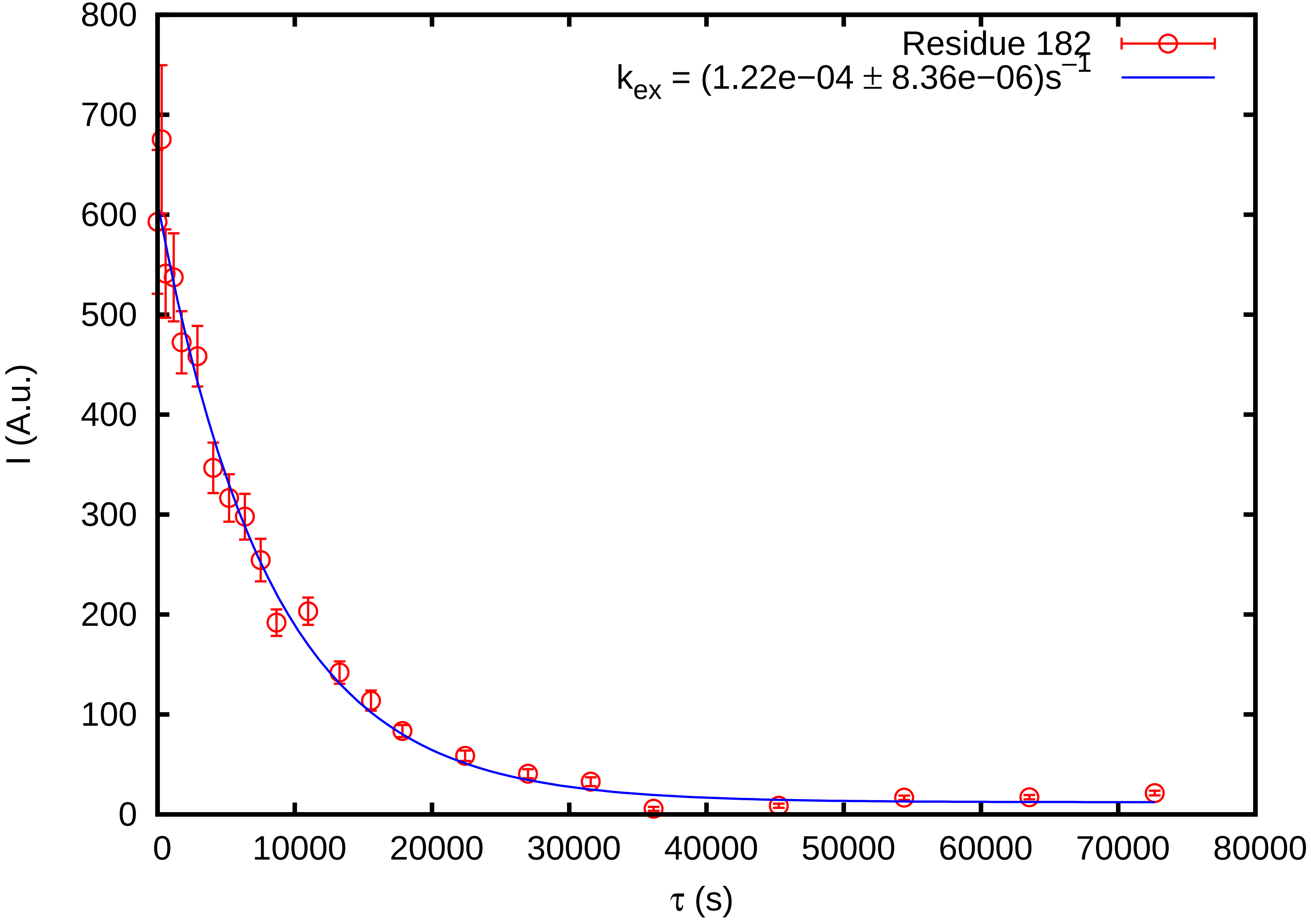

Residue 182
k_ex = (1.22e−04 ± 8.36e−06)s^−1
I (A.u.)
τ (s)
0
100
200
300
400
500
600
700
800
0
10000
20000
30000
40000
50000
60000
70000
80000

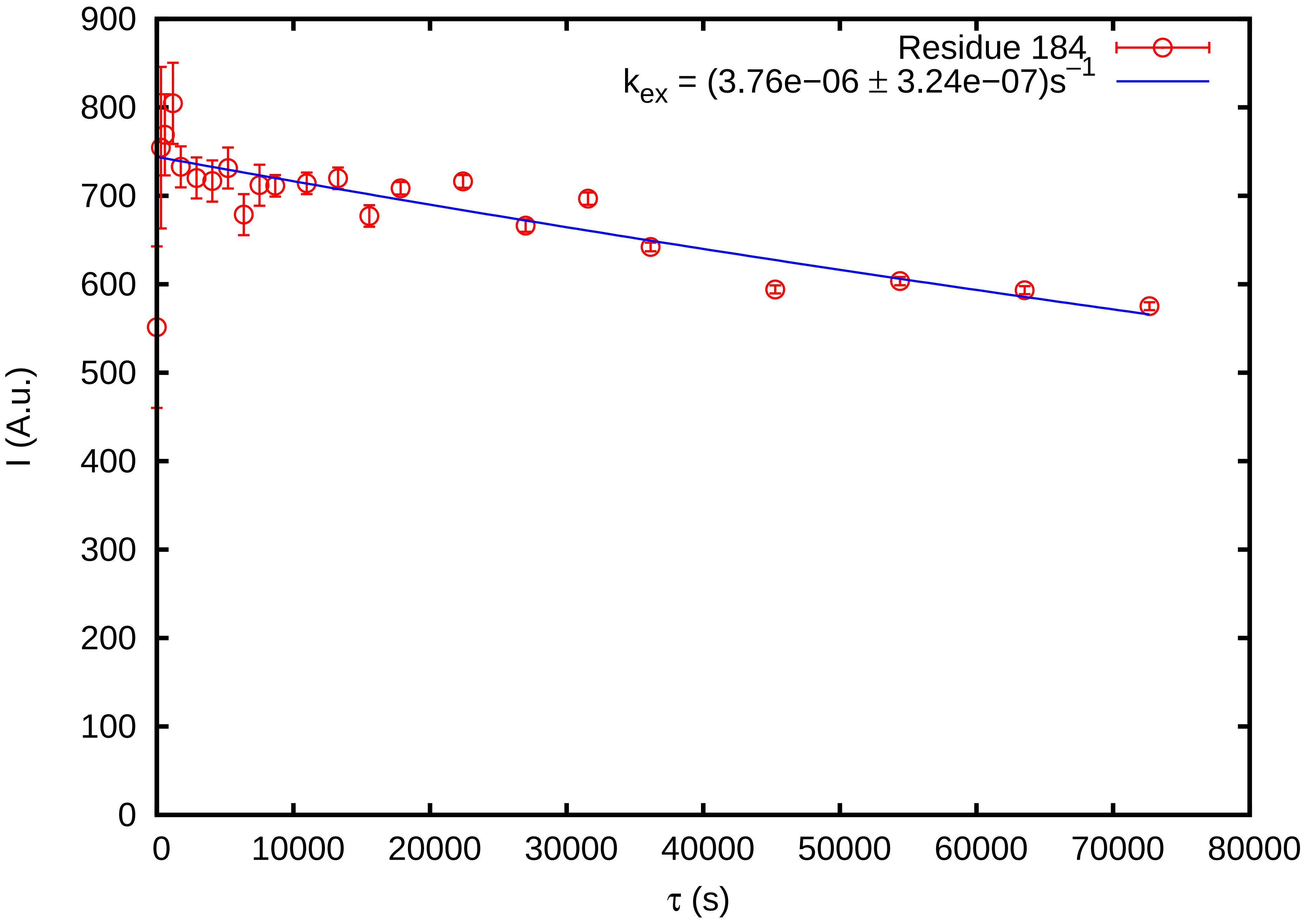
Residue 184
k_{ex} = (3.76e−06 ± 3.24e−07)s^{-1}
I (A.u.)
τ (s)
0
100
200
300
400
500
600
700
800
900
0
10000
20000
30000
40000
50000
60000
70000
80000

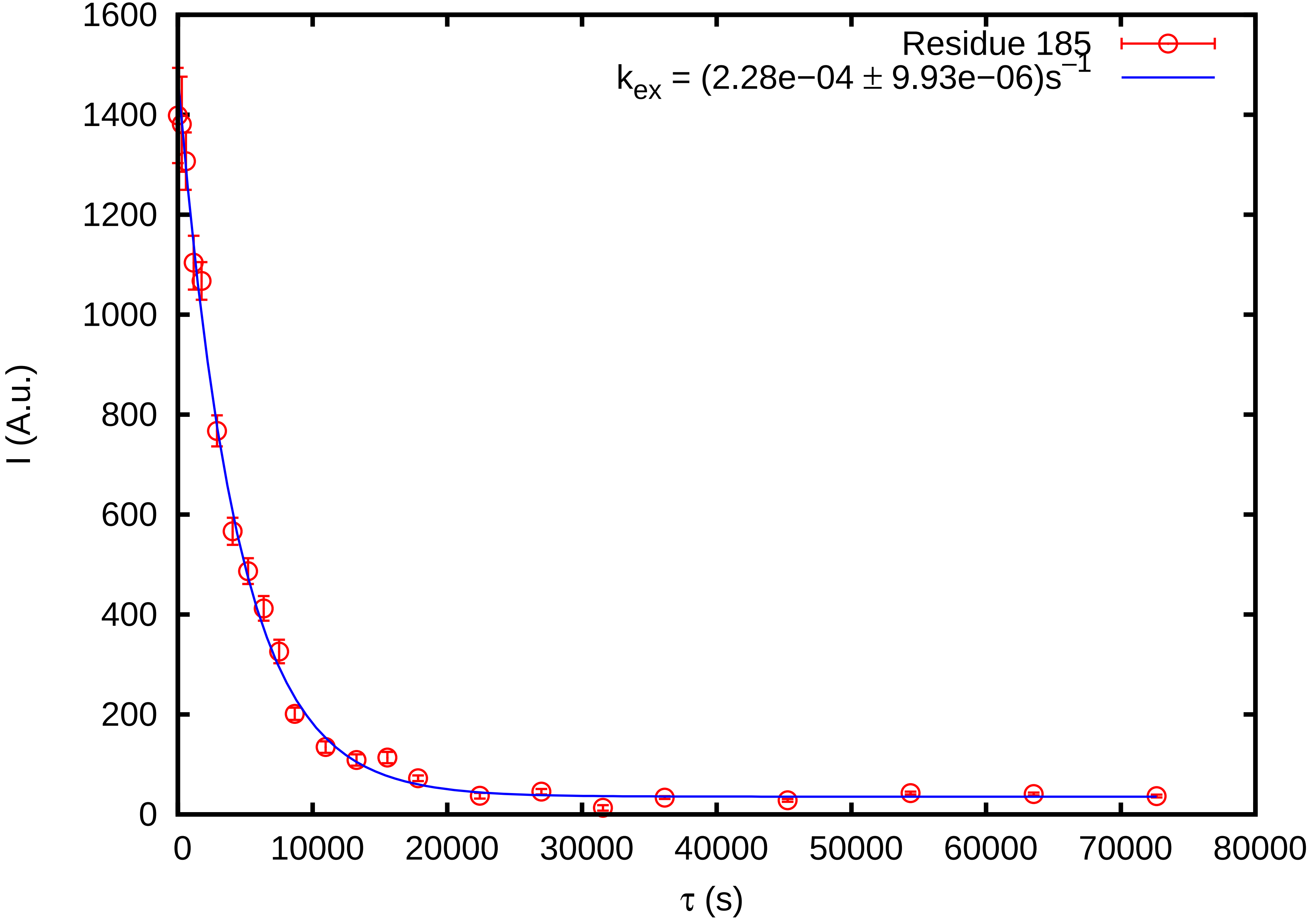

Residue 185
k_ex = (2.28e−04 ± 9.93e−06)s^−1
I (A.u.)
τ (s)
0
200
400
600
800
1000
1200
1400
1600
0
10000
20000
30000
40000
50000
60000
70000
80000

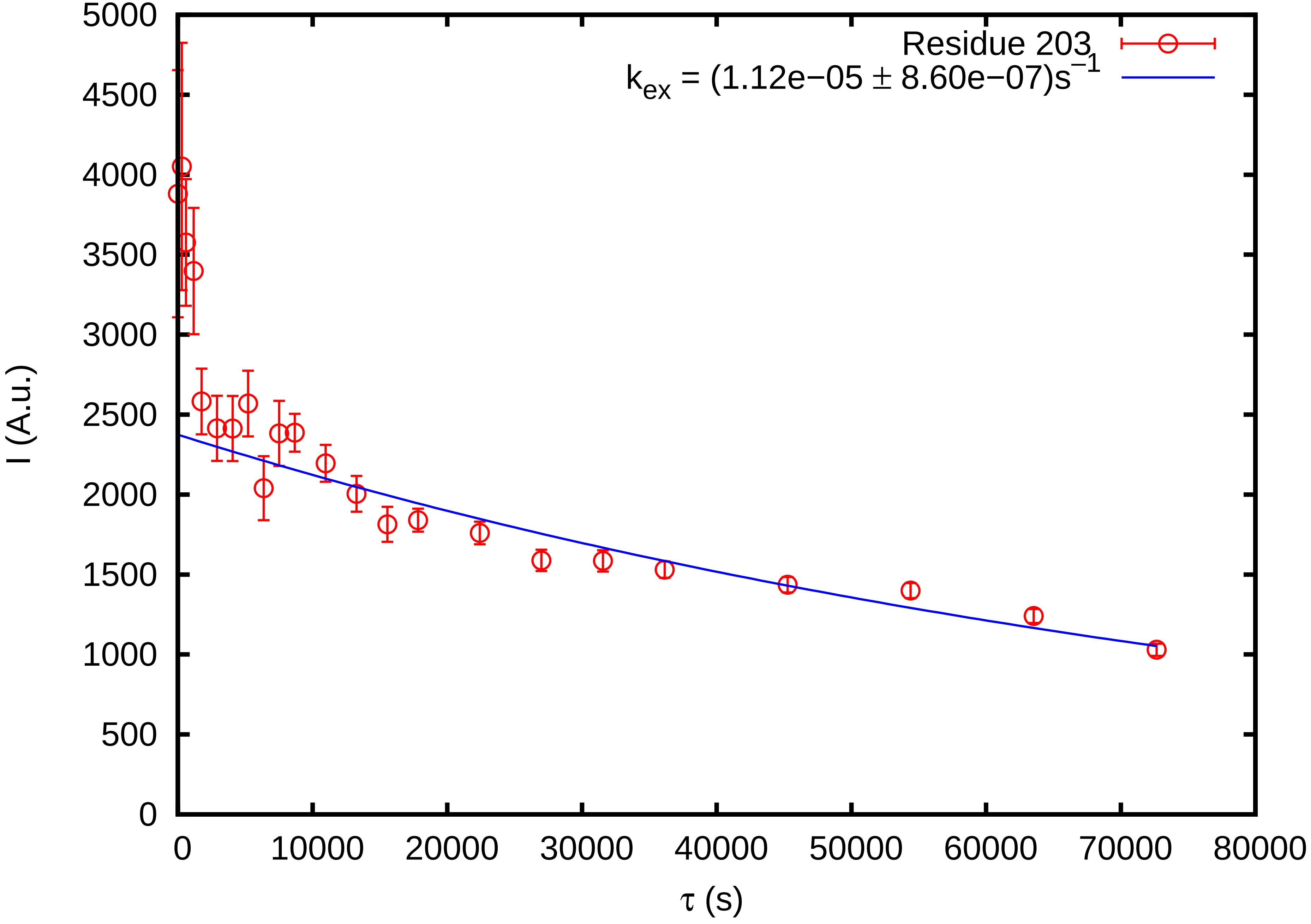

Residue 203
k_{ex} = (1.12e−05 ± 8.60e−07)s^{-1}
I (A.u.)
τ (s)
0
500
1000
1500
2000
2500
3000
3500
4000
4500
5000
0
10000
20000
30000
40000
50000
60000
70000
80000

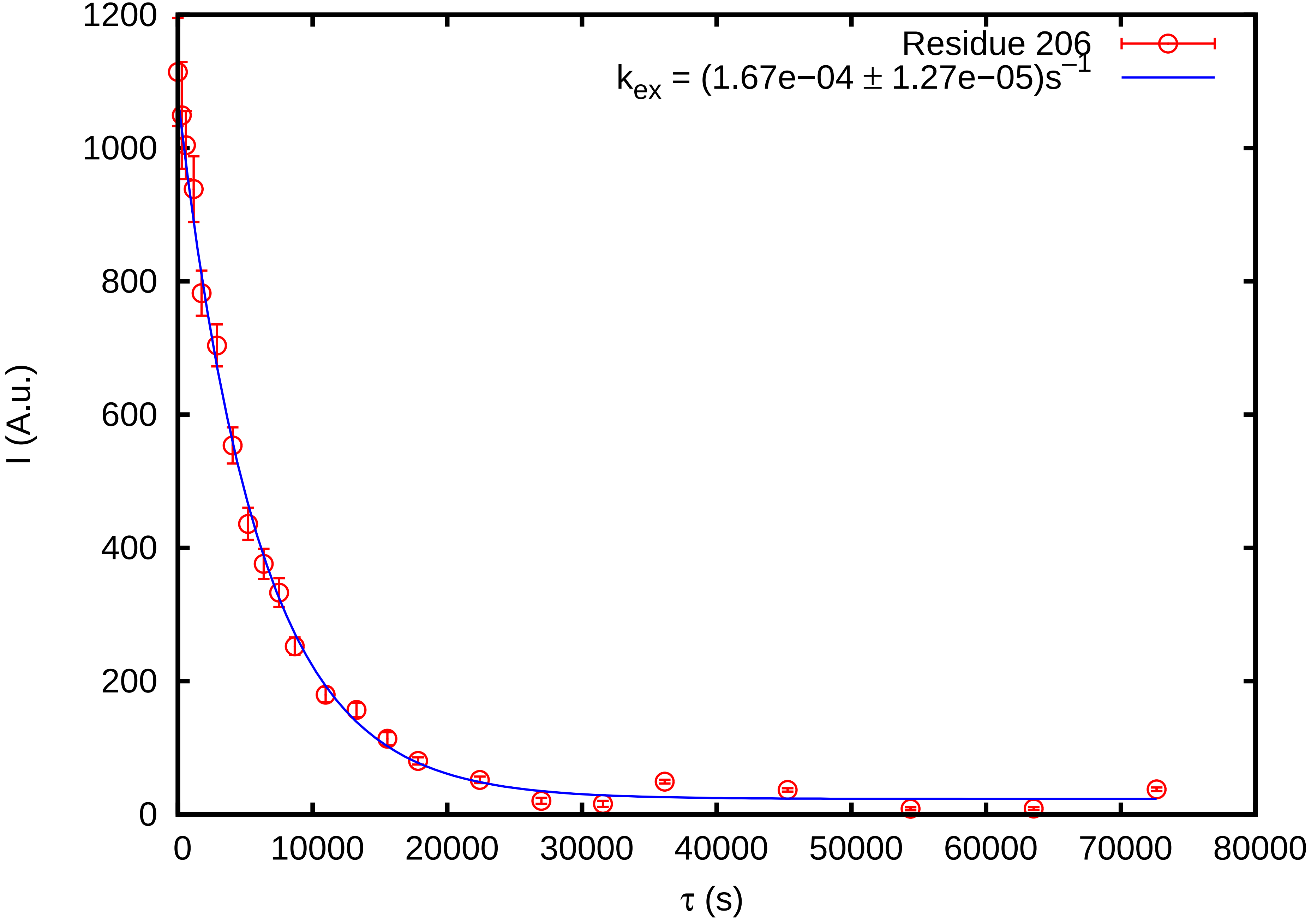

Residue 206
k_{ex} = (1.67e−04 ± 1.27e−05)s^{-1}
I (A.u.)
τ (s)
0
200
400
600
800
1000
1200
0
10000
20000
30000
40000
50000
60000
70000
80000

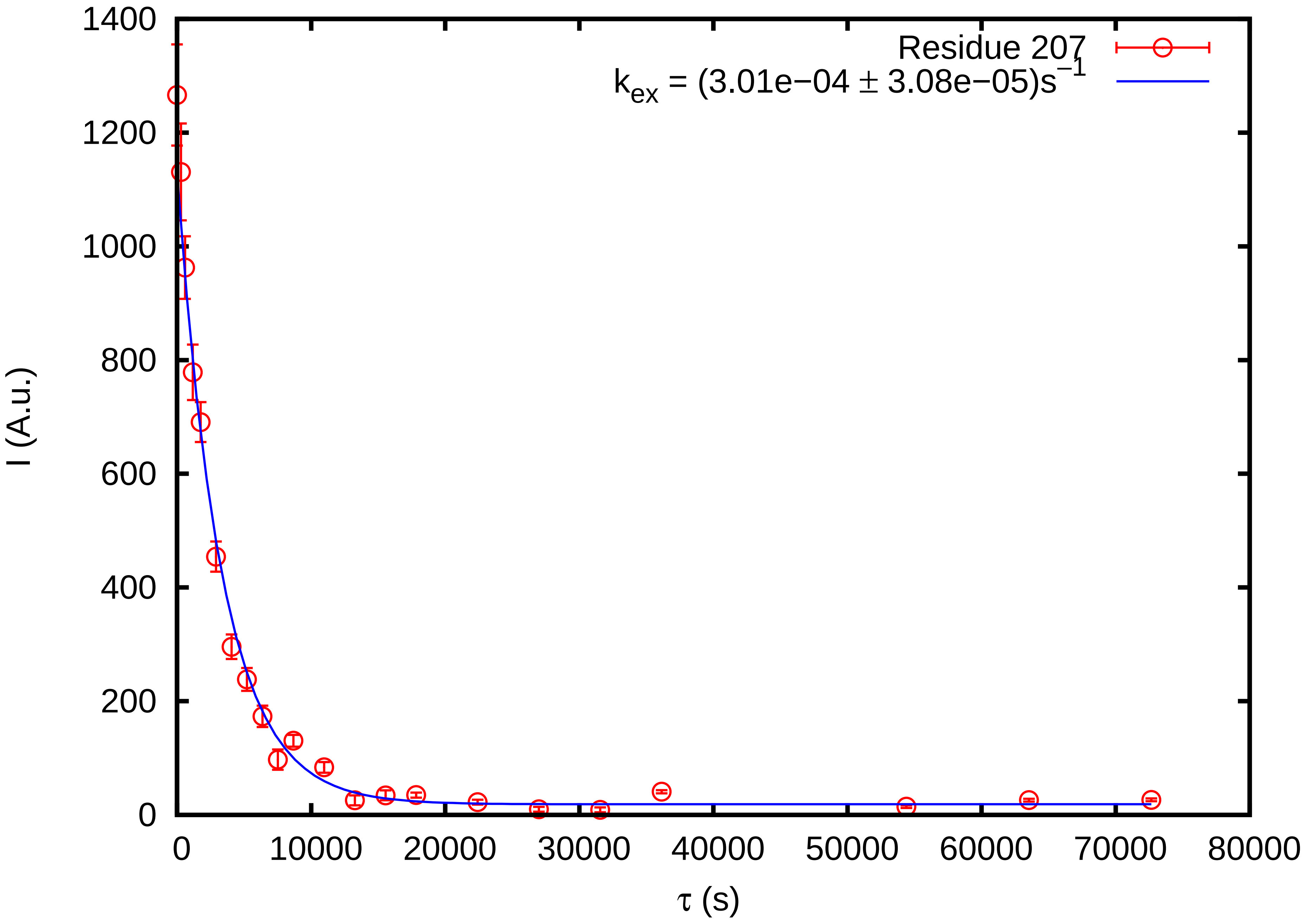

Residue 207
k_{ex} = (3.01e−04 ± 3.08e−05)s^{-1}
I (A.u.)
τ (s)
1400
1200
1000
800
600
400
200
0
10000
20000
30000
40000
50000
60000
70000
80000

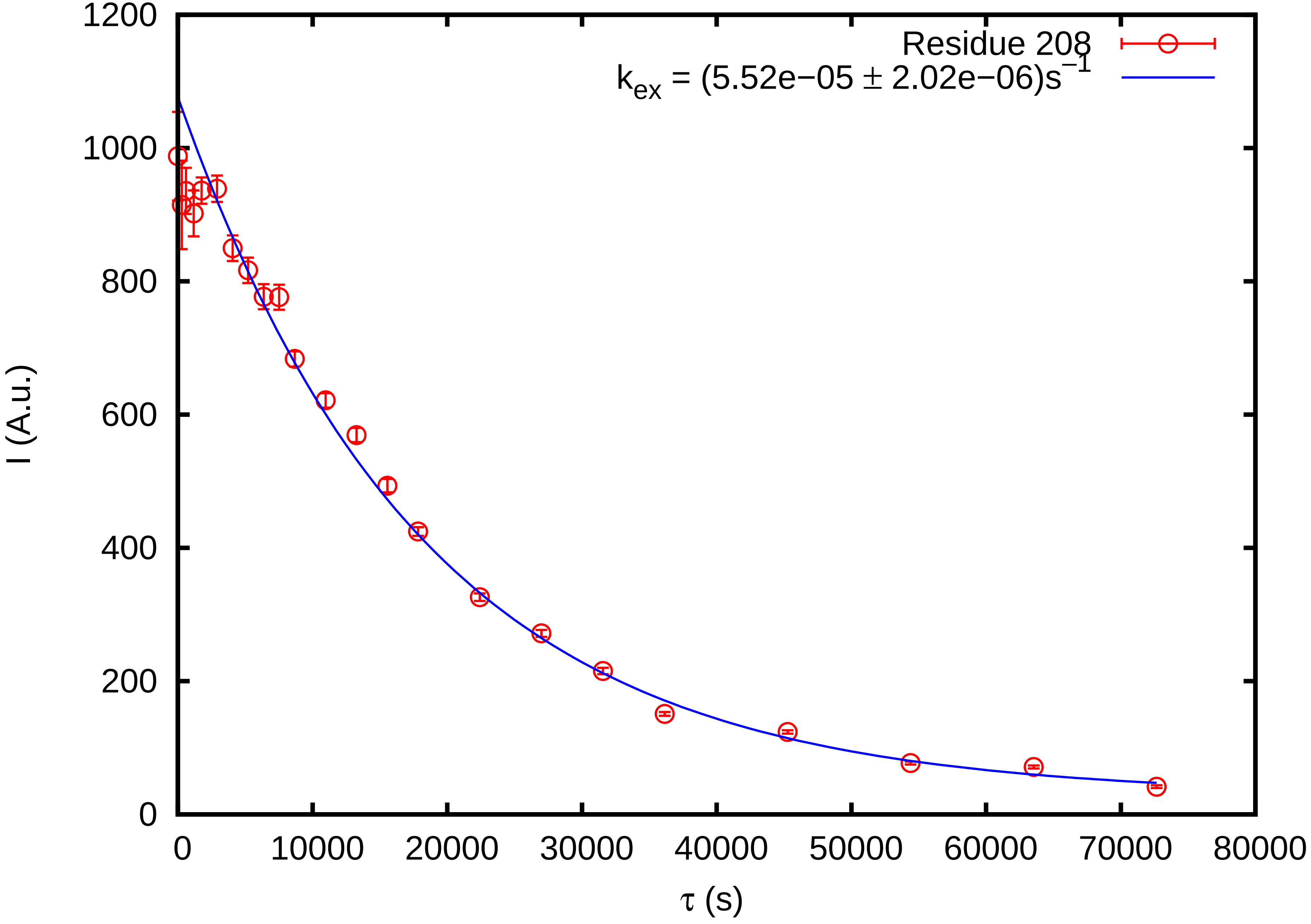

Residue 208
k_ex = (5.52e−05 ± 2.02e−06)s^−1
I (A.u.)
τ (s)
0
200
400
600
800
1000
1200
0
10000
20000
30000
40000
50000
60000
70000
80000

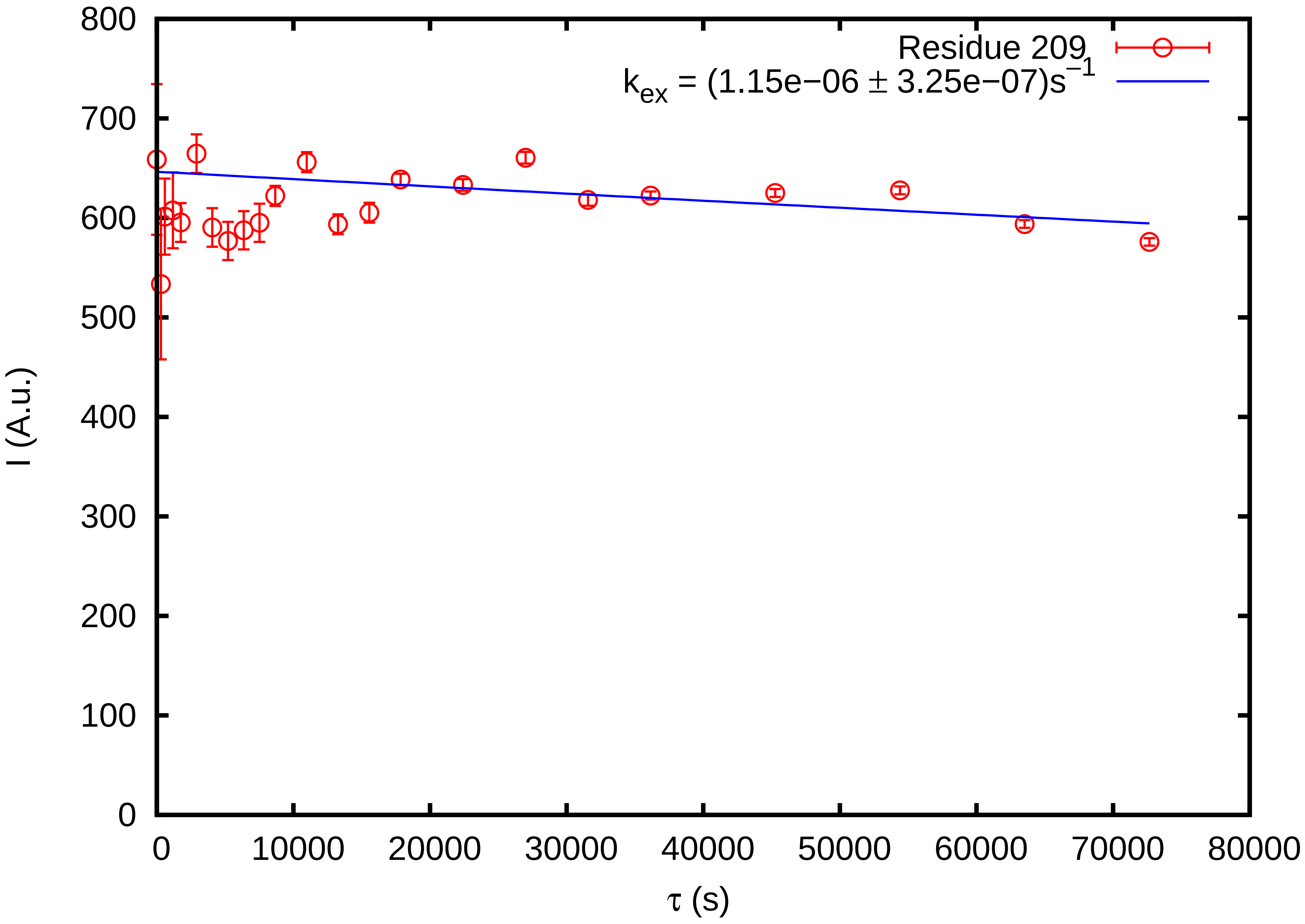

Residue 209
k_ex = (1.15e−06 ± 3.25e−07)s^−1
I (A.u.)
τ (s)
0
100
200
300
400
500
600
700
800
0
10000
20000
30000
40000
50000
60000
70000
80000

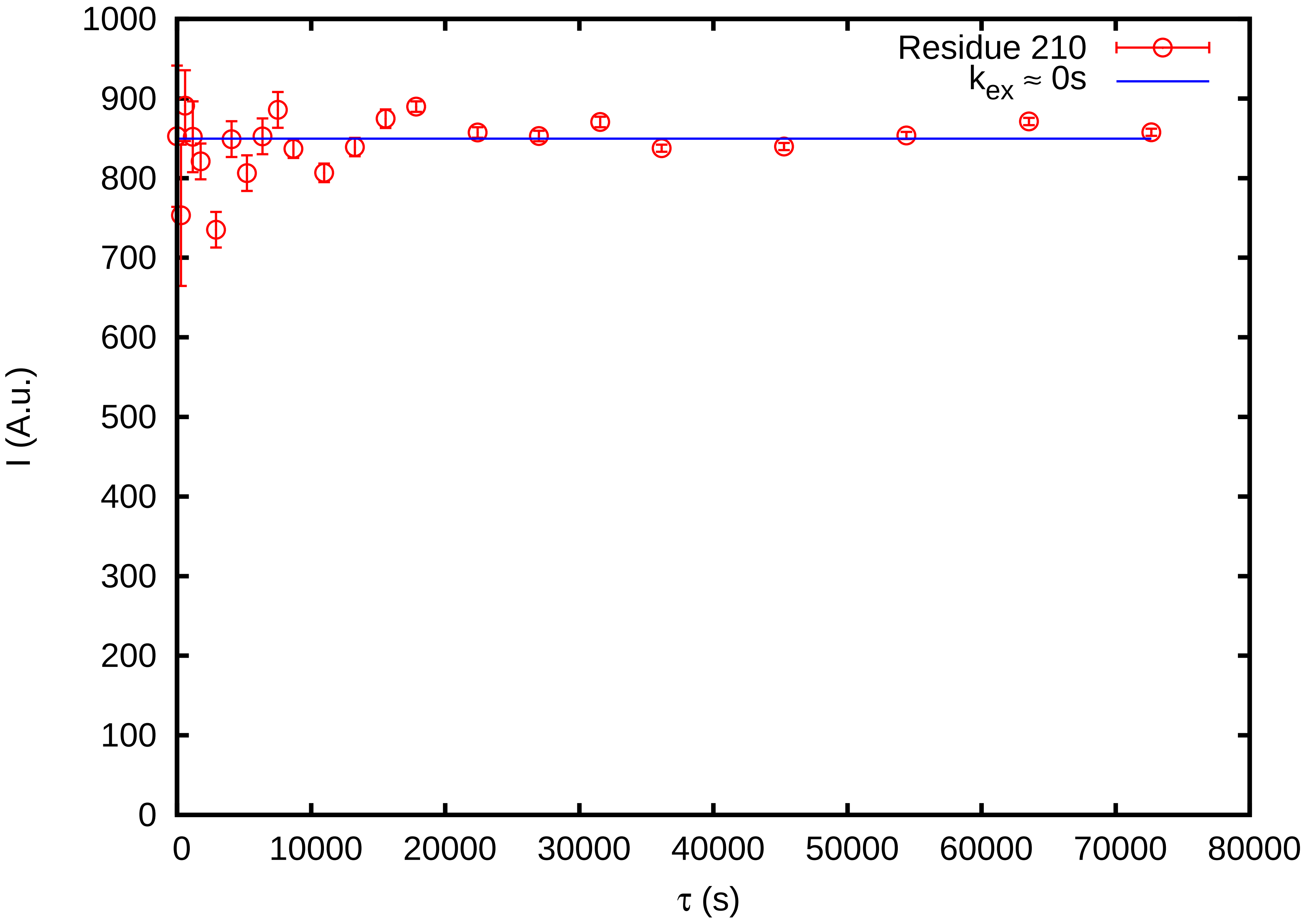

Residue 210
$k_{ex} \approx 0s$
I (A.u.)
τ (s)
0
100
200
300
400
500
600
700
800
900
1000
0
10000
20000
30000
40000
50000
60000
70000
80000

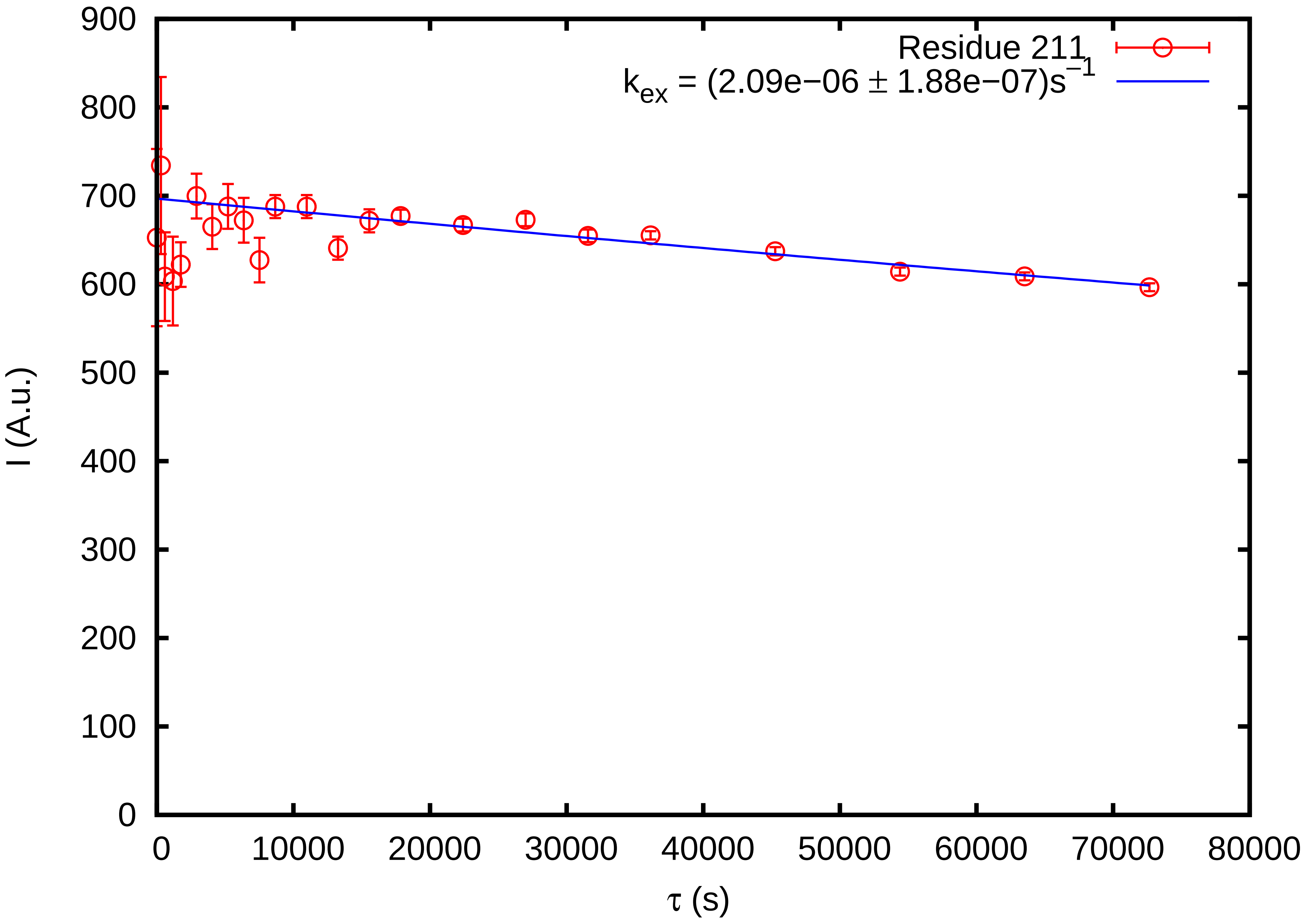

Residue 211
k_{ex} = (2.09e−06 ± 1.88e−07)s^{-1}
I (A.u.)
τ (s)
0
100
200
300
400
500
600
700
800
900
0
10000
20000
30000
40000
50000
60000
70000
80000

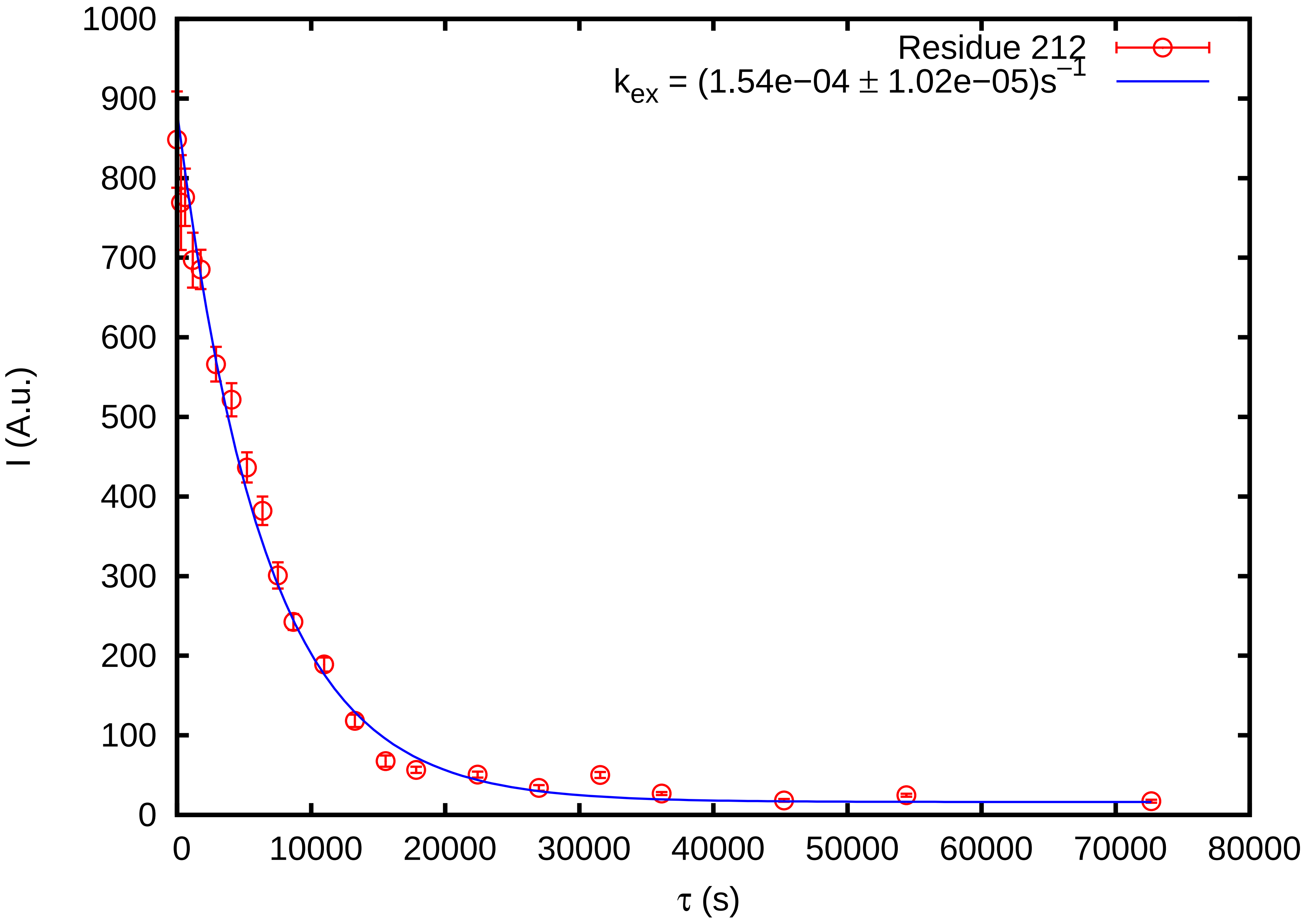

Residue 212
k_{ex} = (1.54e−04 ± 1.02e−05)s^{-1}
I (A.u.)
τ (s)
0
100
200
300
400
500
600
700
800
900
1000
0
10000
20000
30000
40000
50000
60000
70000
80000

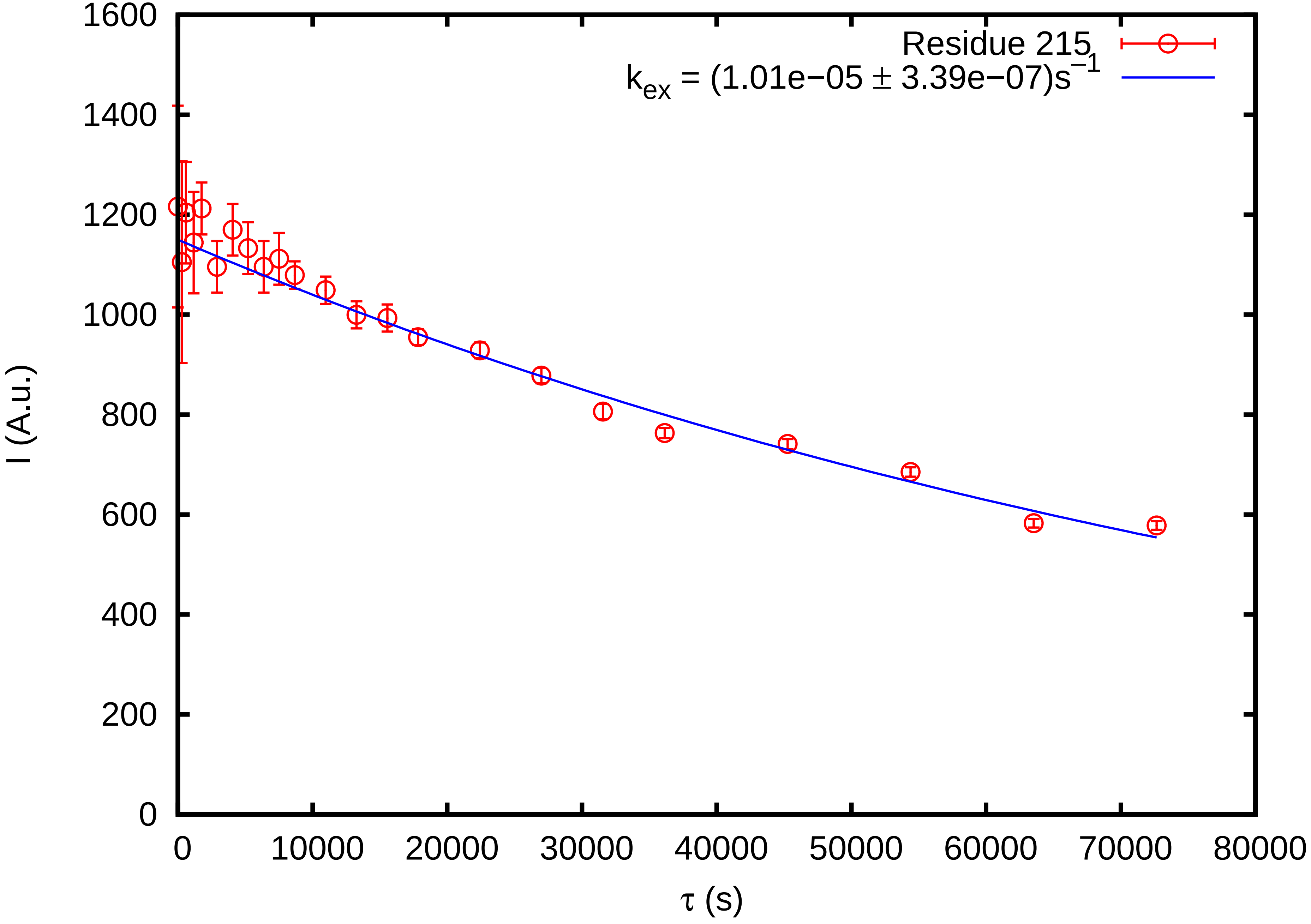

Residue 215
k_{ex} = (1.01e−05 ± 3.39e−07)s^{-1}
I (A.u.)
τ (s)
0
200
400
600
800
1000
1200
1400
1600
0
10000
20000
30000
40000
50000
60000
70000
80000

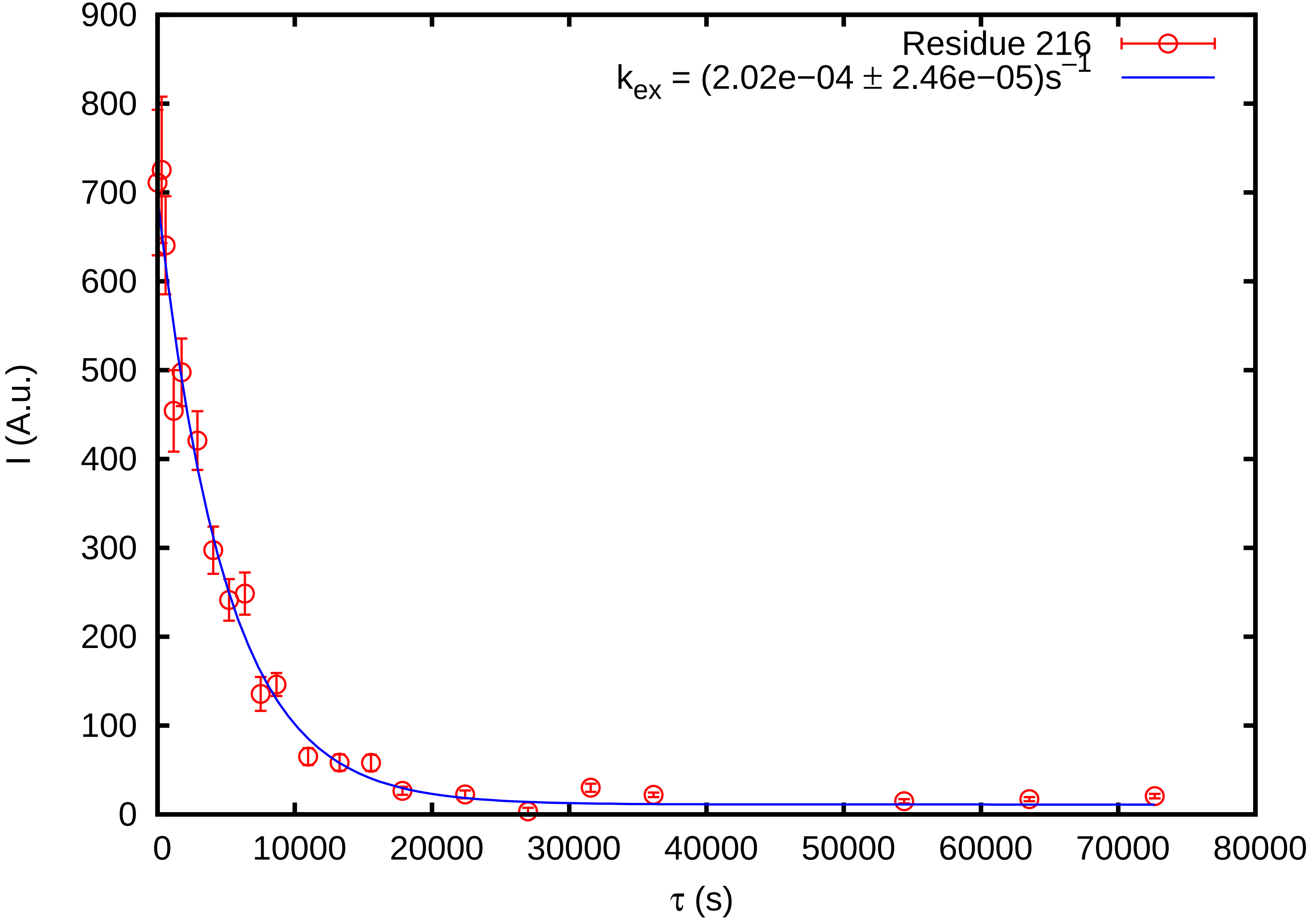

Residue 216
k_{ex} = (2.02e−04 ± 2.46e−05)s^{-1}
I (A.u.)
τ (s)
0
100
200
300
400
500
600
700
800
900
10000
20000
30000
40000
50000
60000
70000
80000

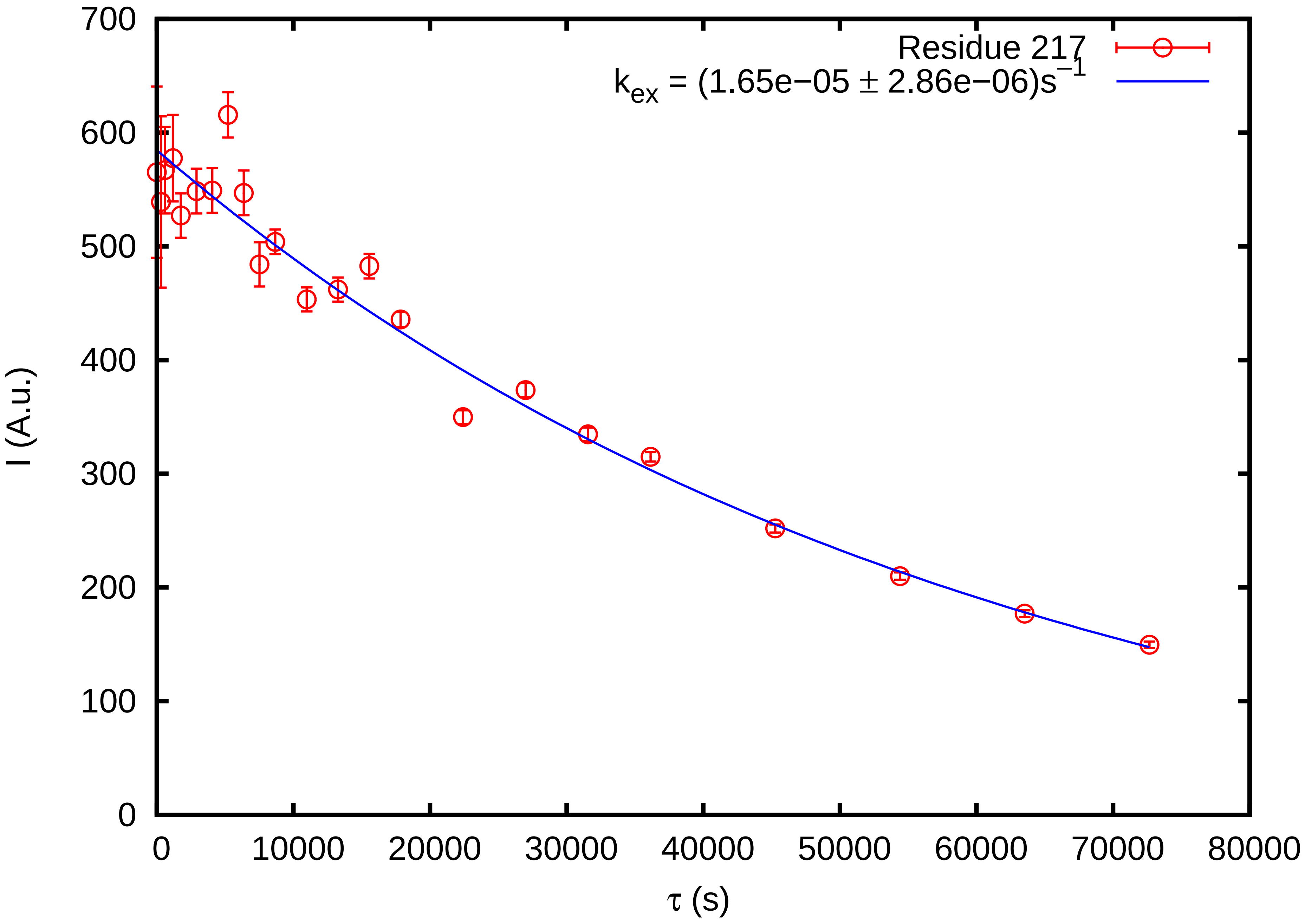

Residue 217
k_{ex} = (1.65e−05 ± 2.86e−06)s^{-1}
I (A.u.)
τ (s)
0
100
200
300
400
500
600
700
0
10000
20000
30000
40000
50000
60000
70000
80000

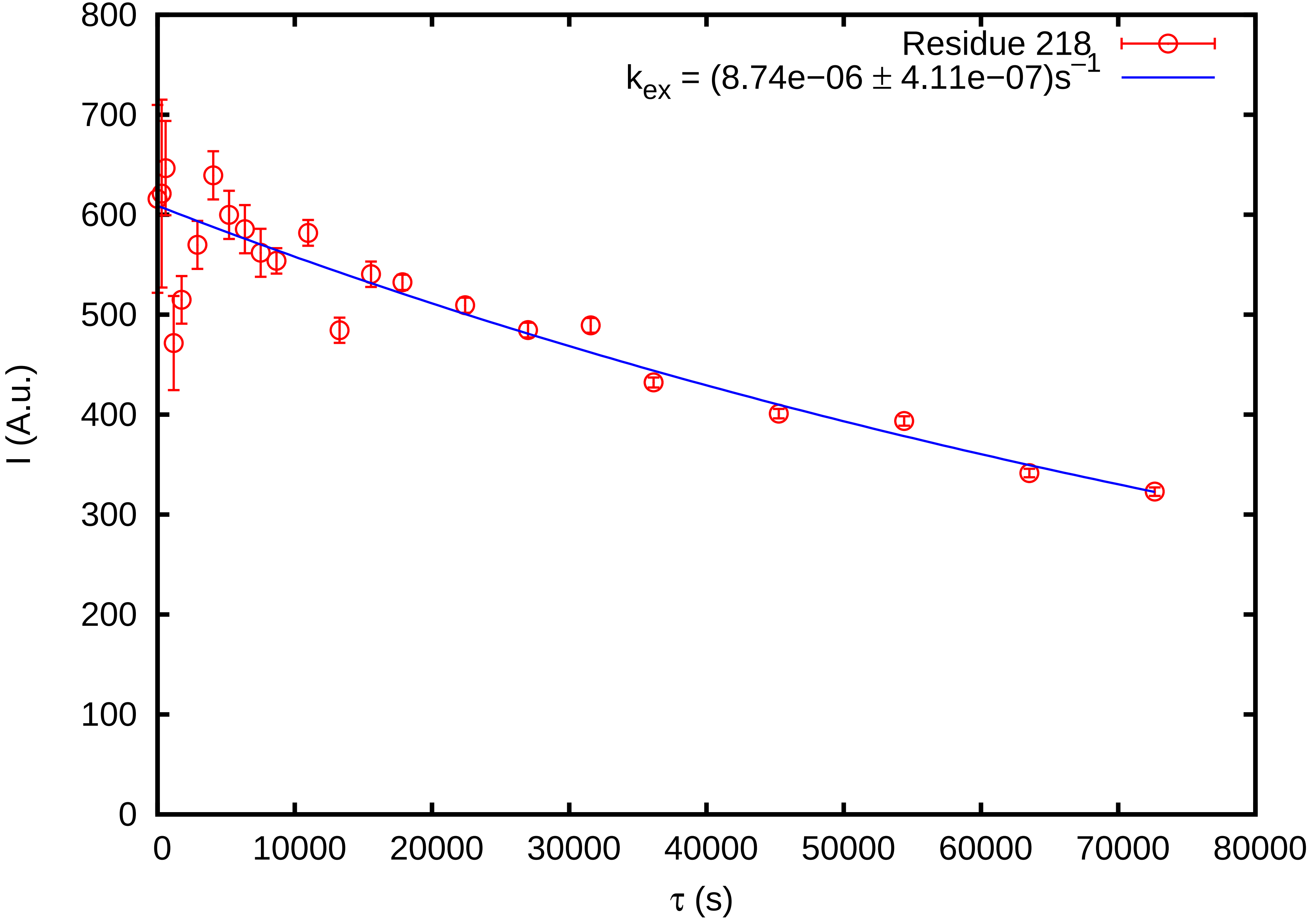

Residue 218
k_{ex} = (8.74e−06 ± 4.11e−07)s^{-1}
I (A.u.)
τ (s)
0
100
200
300
400
500
600
700
800
0
10000
20000
30000
40000
50000
60000
70000
80000
