# Supplementary material for: Interplay of buried histidine protonation and protein stability in prion misfolding
Source: Sci Rep. 2017 Apr 13;7:882. doi: 10.1038/s41598-017-00954-7 (PMC5429843; doi:10.1038/s41598-017-00954-7)
Supplement: Supplementary file 4 — Supplementary Figure S6 [file 41598_2017_954_MOESM4_ESM.pdf]

Interplay of buried histidine protonation and protein stability in prion misfolding

Supplementary Figure S6

A. Malevanets[1], P. Andrew Chong[1,2], D.F. Hansen[2,3,+], P. Rizk[1,2], Y. Sun[4], H. Lin[1], R. Muhandiram[2,3], A. Chakrabartty[4], Lewis E. Kay[1,2,3,5], J.D. Forman-Kay[1,2,**] and Shoshana J. Wodak[1,2,3§**]

[1]Program in Molecular Structure and Function, Hospital for Sick Children, 555 University Ave., Toronto, ON, Canada M5G 0A4

[2]Department of Biochemistry and [3]Department of Molecular Genetics, University of Toronto, Toronto, ON, Canada M5S 1A8

[4]Department of Medical Biophysics, University of Toronto, Toronto, ON, Canada M5G 2M9

[5]Department of Chemistry, University of Toronto, Toronto, ON, Canada, M5S 3H6

[+]Current address: University College London, Division of Biosciences, London WC1E 6BT UK

[§]Current address: VIB Structural Biology Research Center, VUB, Pleinlaan 2 1050 Brussels, Belgium

** Corresponding authors.

Figures S6. Hydrogen exchange decay curves for rabbit PrP at pH 7.

Decay curves of peak intensities in 15N-1H correlation maps (O) vertical bars represent the uncertainty of the obtained intensity. The intensities were obtained from a simultaneous analysis and global fit of all spectra with different tau as described previously (Hansen et al (2007)). The solid lines are obtained from best-fits of an exponential decay function $I(t)=I0*\exp(-k_{ex}*tau) + offset$ to the data shown. For peaks with a decay rate slower than ~1e-5 /s the offset and the k_{ex} rate are strongly correlated and the offset was therefore fixed to 0 for these peaks.

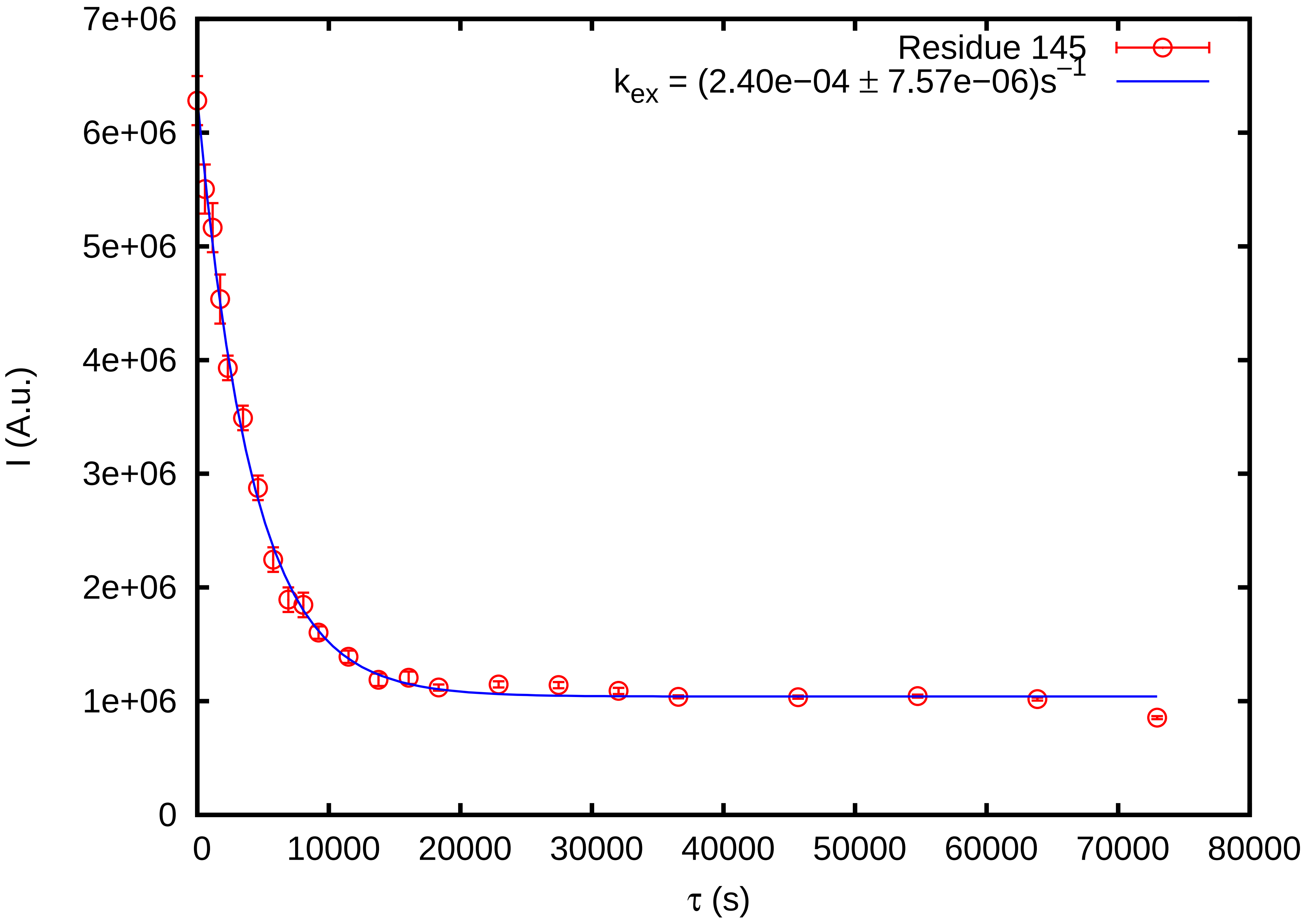

Residue 145
k_ex = (2.40e−04 ± 7.57e−06)s^−1
I (A.u.)
τ (s)
0
1e+06
2e+06
3e+06
4e+06
5e+06
6e+06
7e+06
0
10000
20000
30000
40000
50000
60000
70000
80000

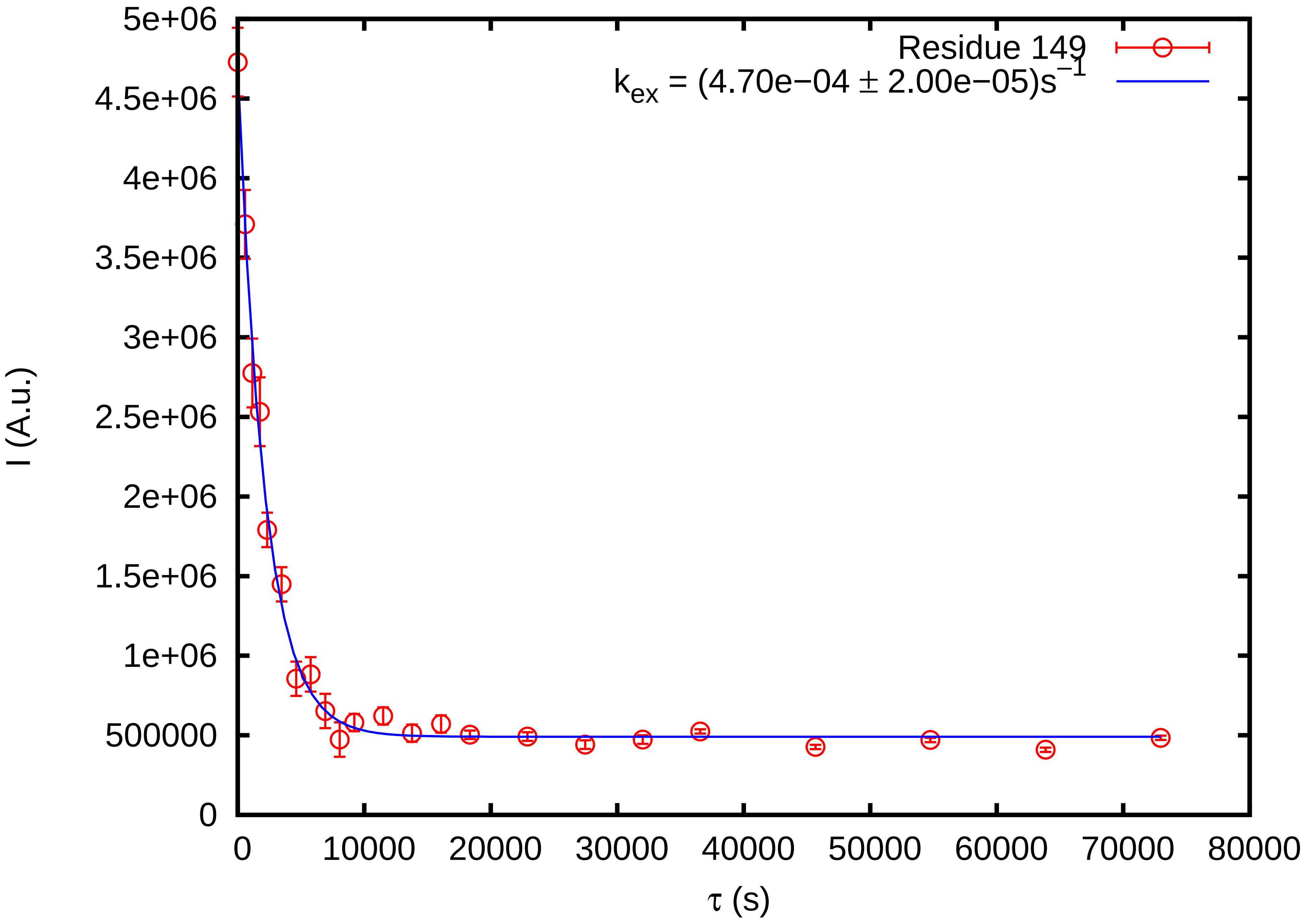

Residue 149
k_{ex} = (4.70e−04 ± 2.00e−05)s^{-1}
I (A.u.)
τ (s)
5e+06
4.5e+06
4e+06
3.5e+06
3e+06
2.5e+06
2e+06
1.5e+06
1e+06
500000
0
10000
20000
30000
40000
50000
60000
70000
80000

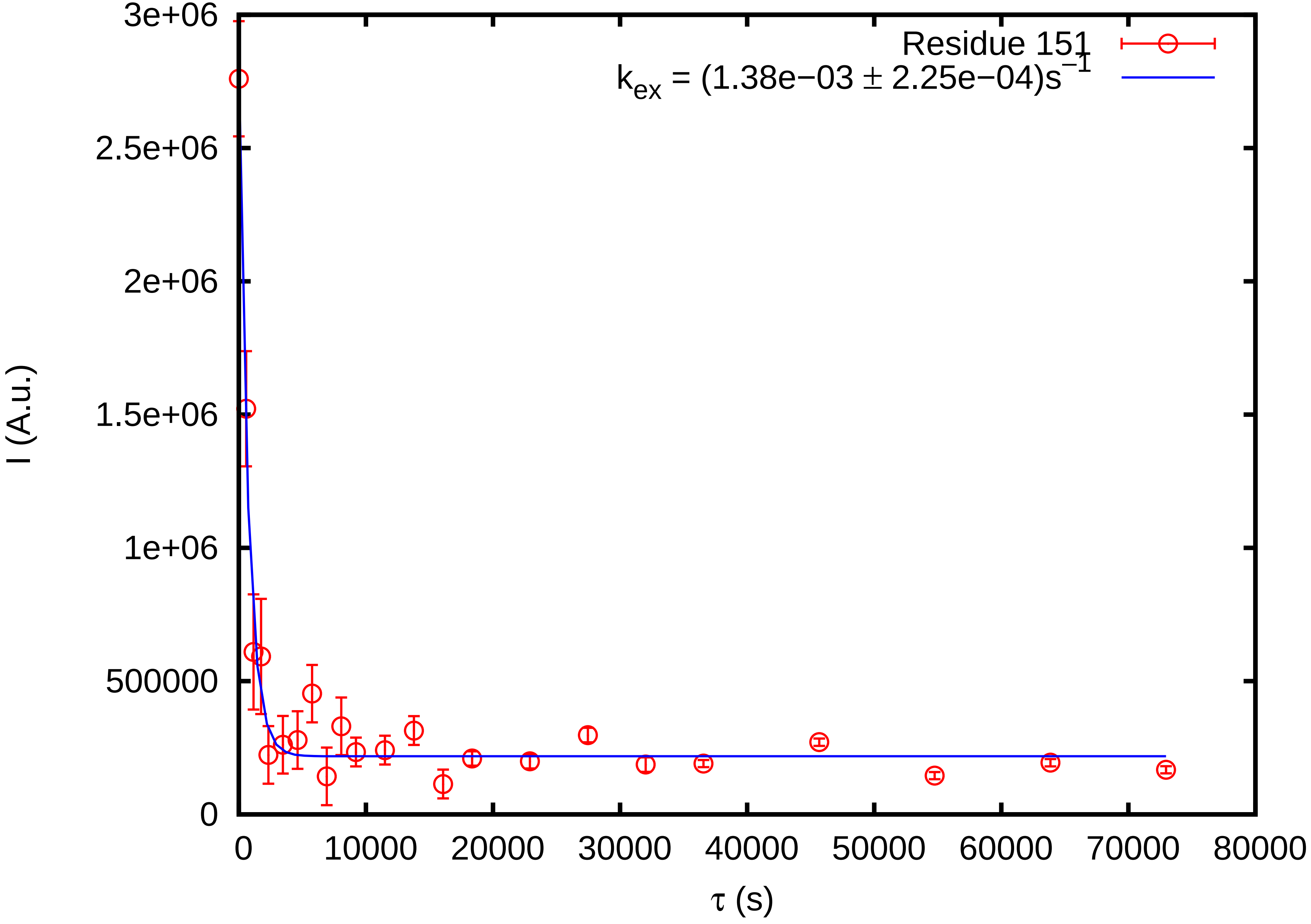

Residue 151
k_{ex} = (1.38e−03 ± 2.25e−04)s^{-1}
I (A.u.)
τ (s)
0
500000
1e+06
1.5e+06
2e+06
2.5e+06
3e+06
0
10000
20000
30000
40000
50000
60000
70000
80000

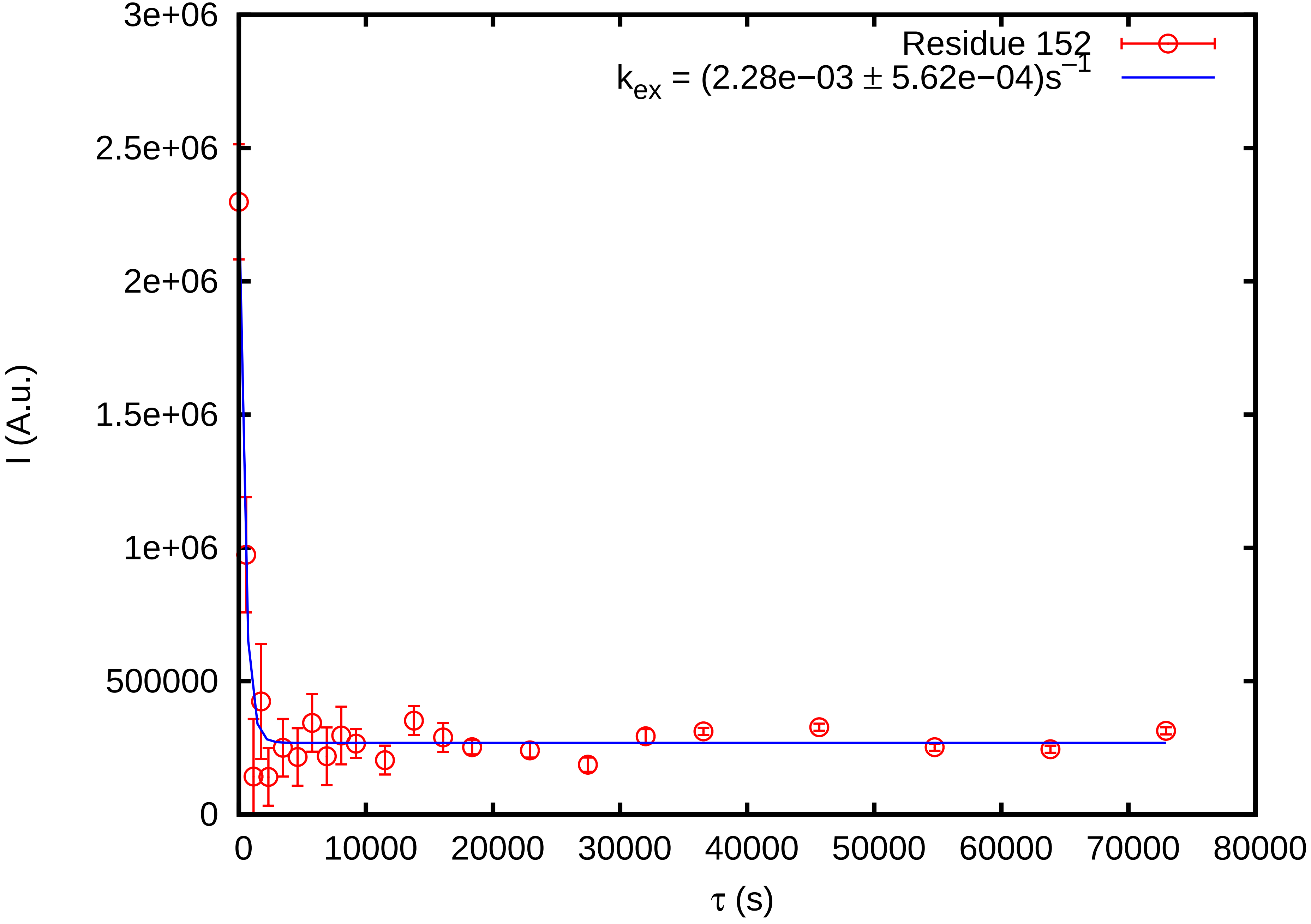

Residue 152
k_{ex} = (2.28e−03 ± 5.62e−04)s^{-1}
I (A.u.)
τ (s)
3e+06
2.5e+06
2e+06
1.5e+06
1e+06
500000
0
10000
20000
30000
40000
50000
60000
70000
80000

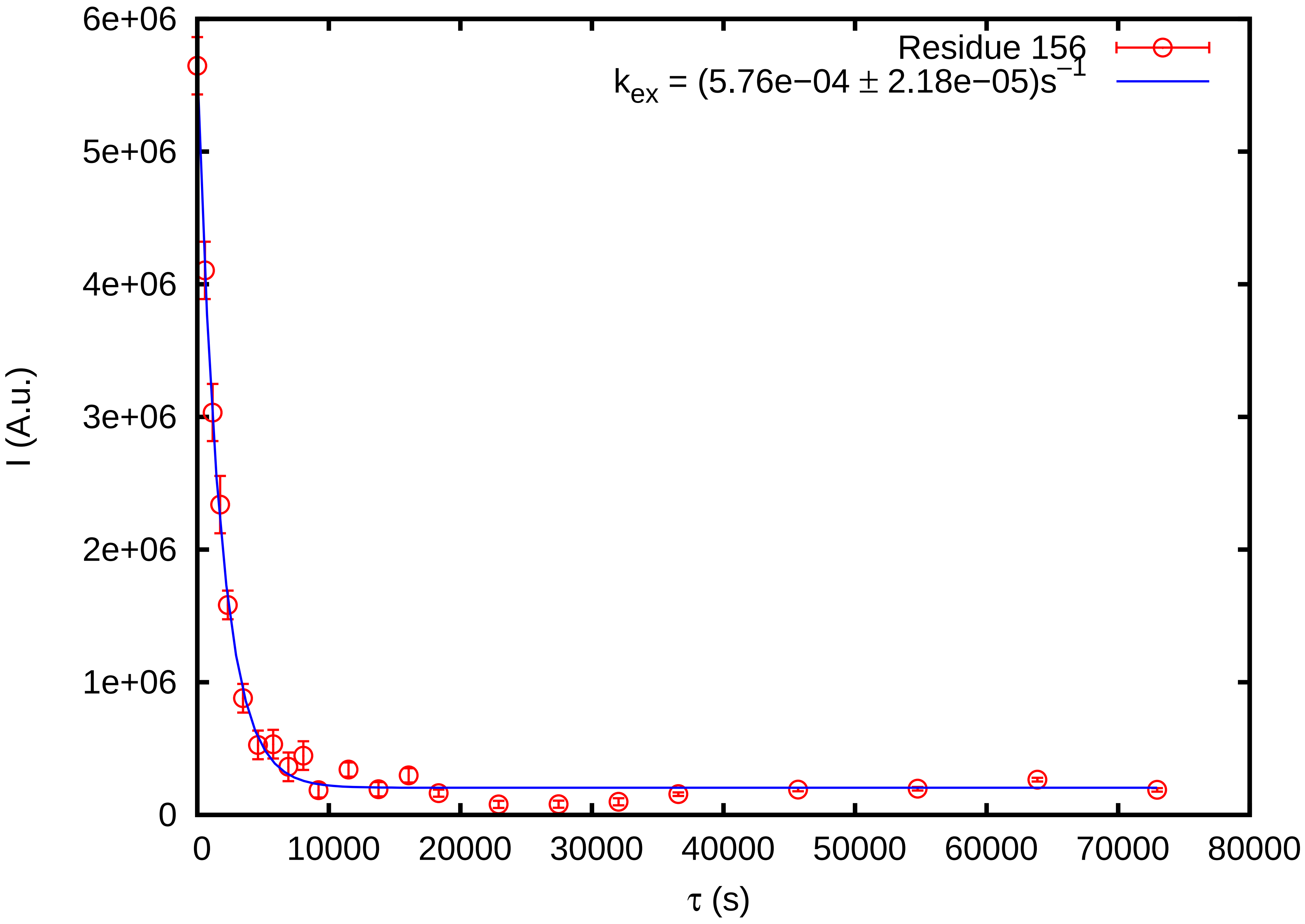

Residue 156
k_{ex} = (5.76e−04 ± 2.18e−05)s^{-1}
I (A.u.)
τ (s)
6e+06
5e+06
4e+06
3e+06
2e+06
1e+06
0
0
10000
20000
30000
40000
50000
60000
70000
80000

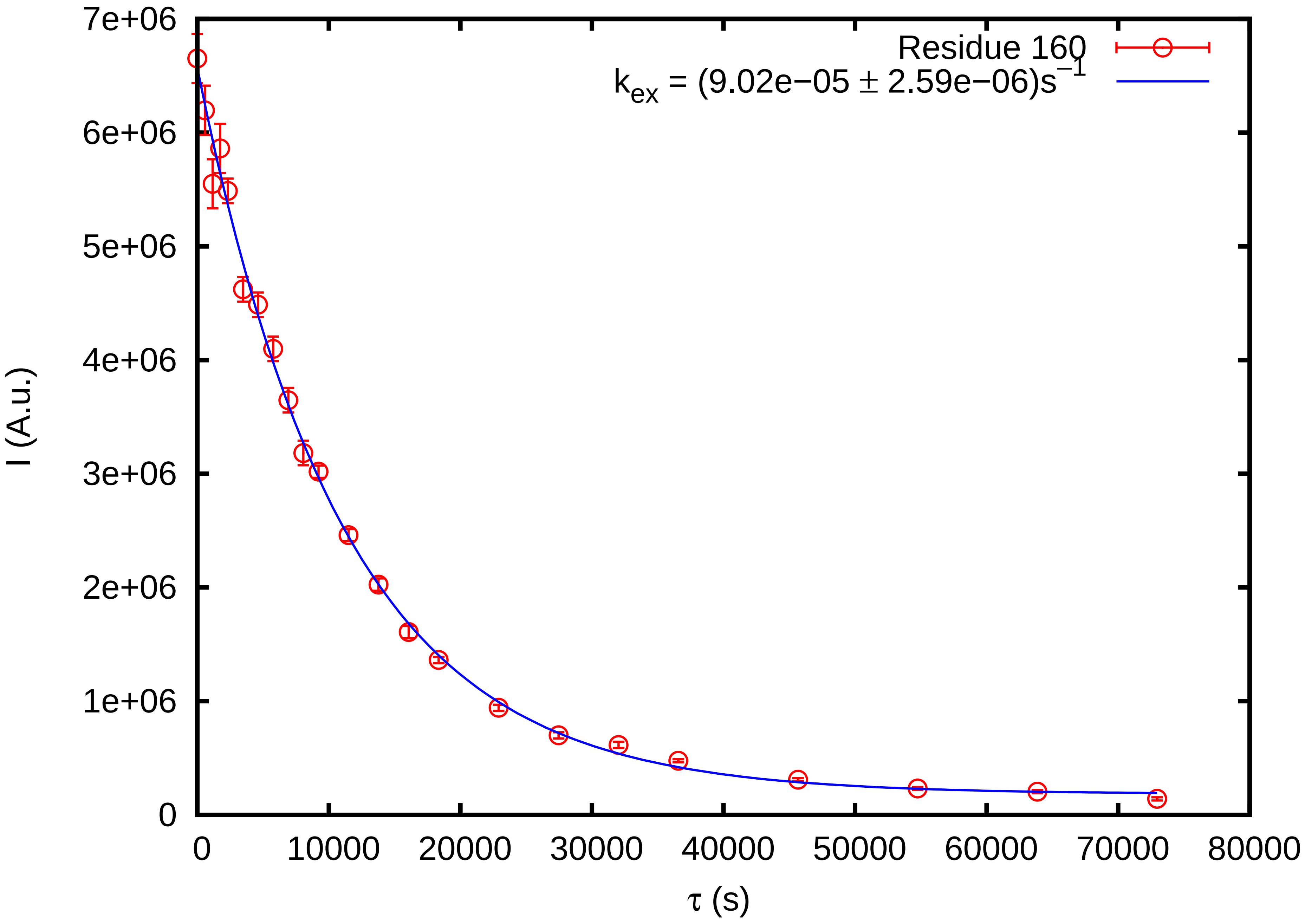

Residue 160
$k_{ex} = (9.02e{-}05 \pm 2.59e{-}06)s^{-1}$
I (A.u.)
τ (s)
0
1e+06
2e+06
3e+06
4e+06
5e+06
6e+06
7e+06
0
10000
20000
30000
40000
50000
60000
70000
80000

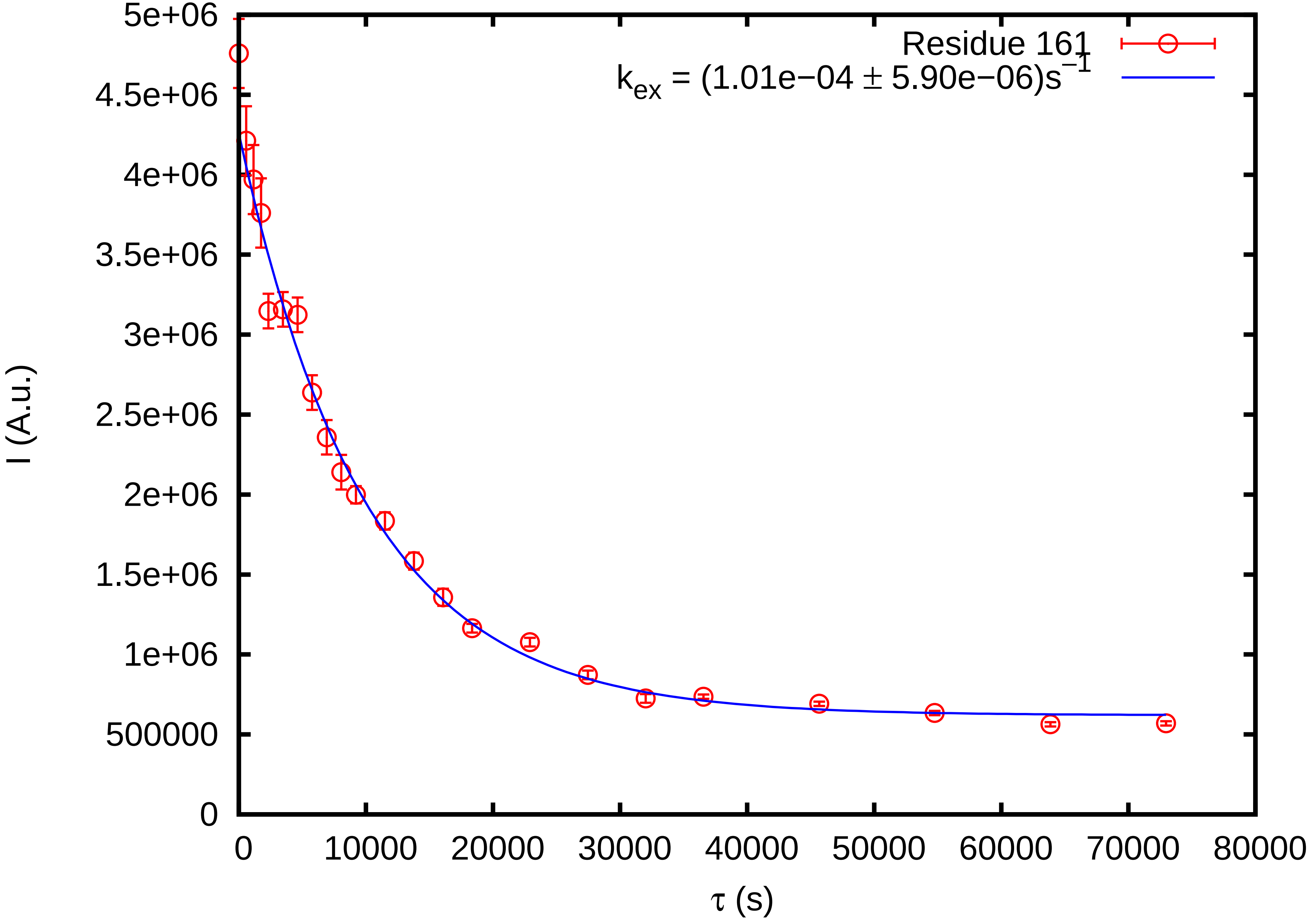

Residue 161
k_{ex} = (1.01e−04 ± 5.90e−06)s^{-1}
I (A.u.)
τ (s)
5e+06
4.5e+06
4e+06
3.5e+06
3e+06
2.5e+06
2e+06
1.5e+06
1e+06
500000
0
10000
20000
30000
40000
50000
60000
70000
80000

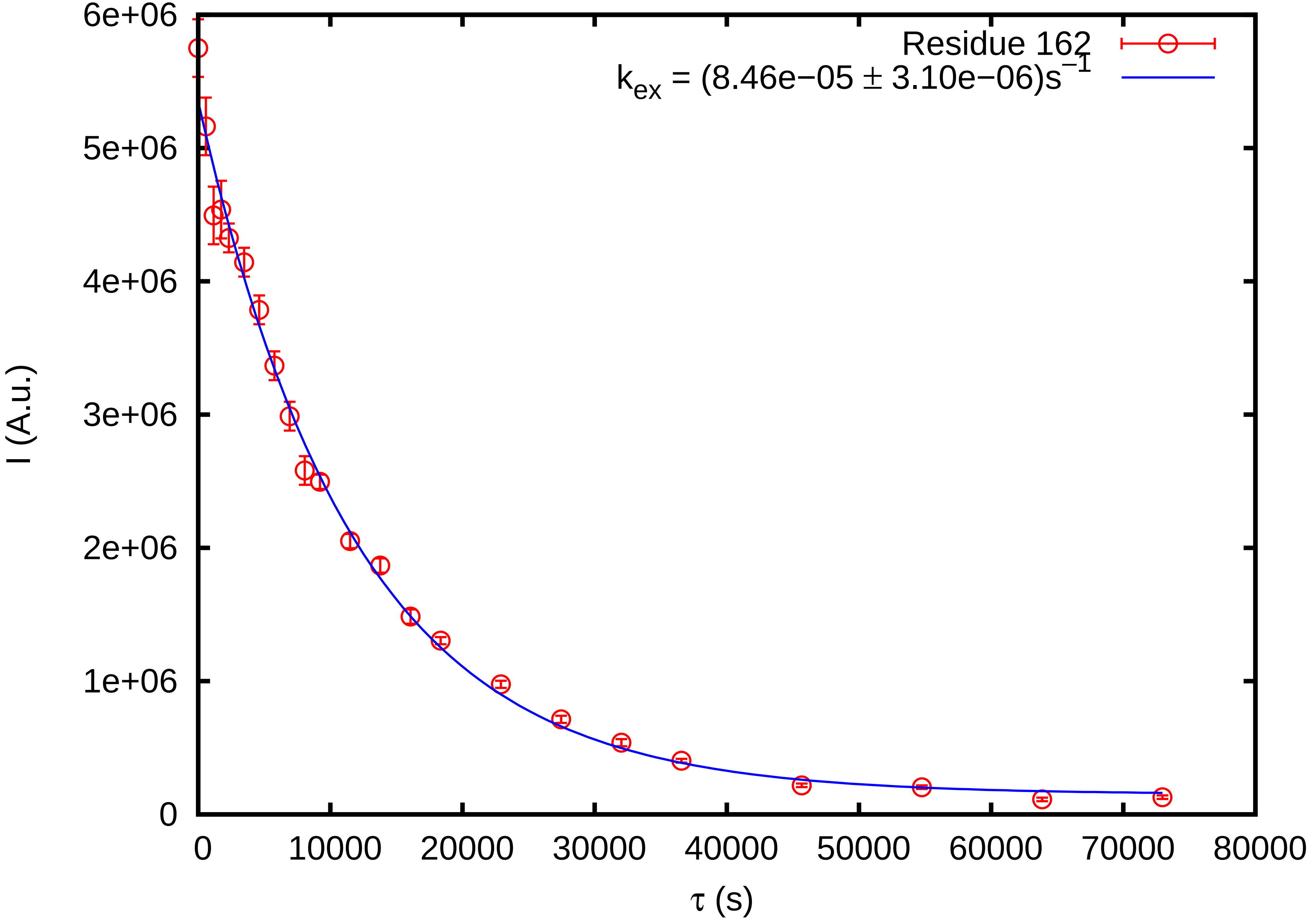

Residue 162
k_ex = (8.46e−05 ± 3.10e−06)s^−1
I (A.u.)
τ (s)
0
1e+06
2e+06
3e+06
4e+06
5e+06
6e+06
0
10000
20000
30000
40000
50000
60000
70000
80000

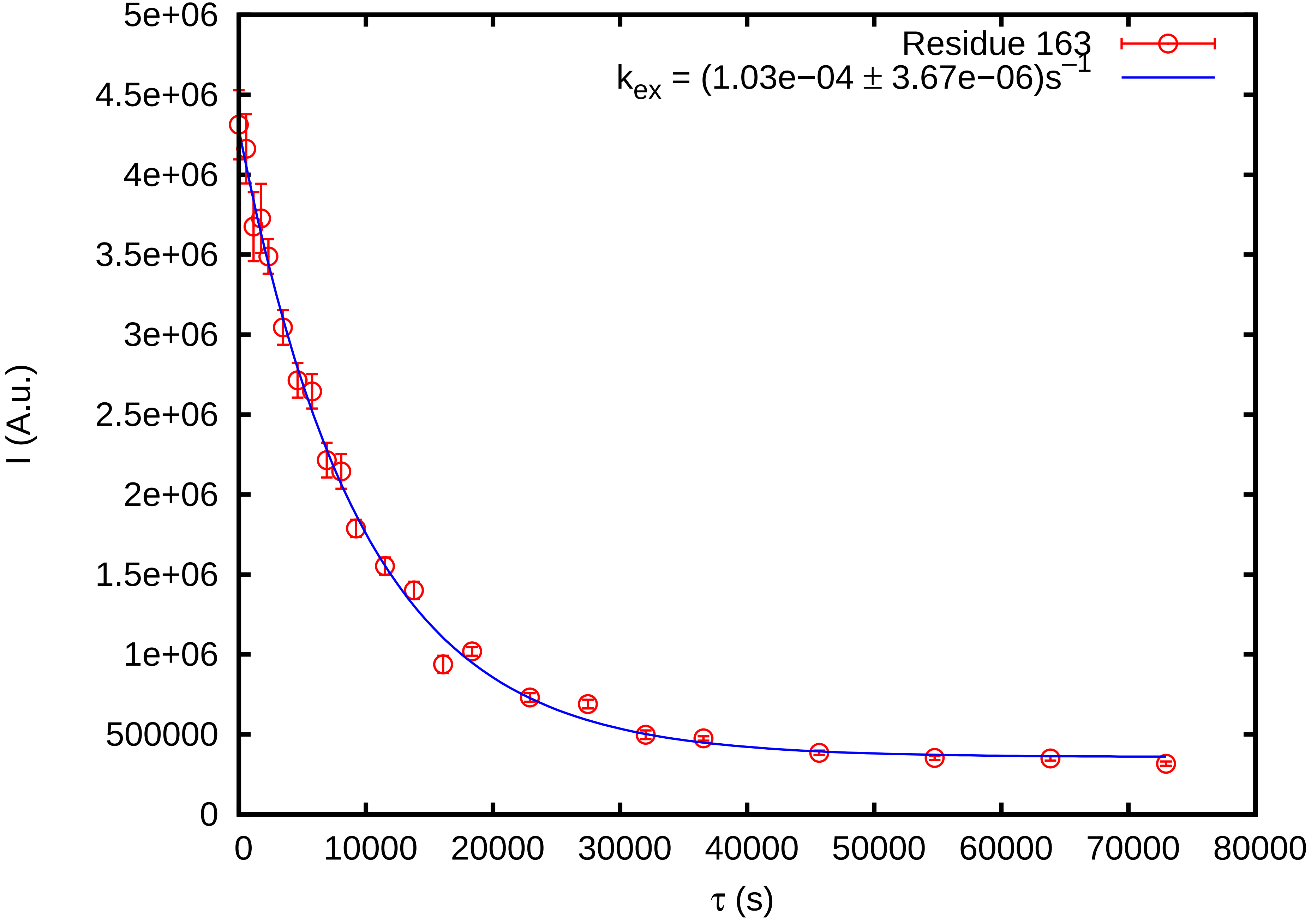

Residue 163
k_{ex} = (1.03e−04 ± 3.67e−06)s^{-1}
I (A.u.)
τ (s)
5e+06
4.5e+06
4e+06
3.5e+06
3e+06
2.5e+06
2e+06
1.5e+06
1e+06
500000
0
10000
20000
30000
40000
50000
60000
70000
80000

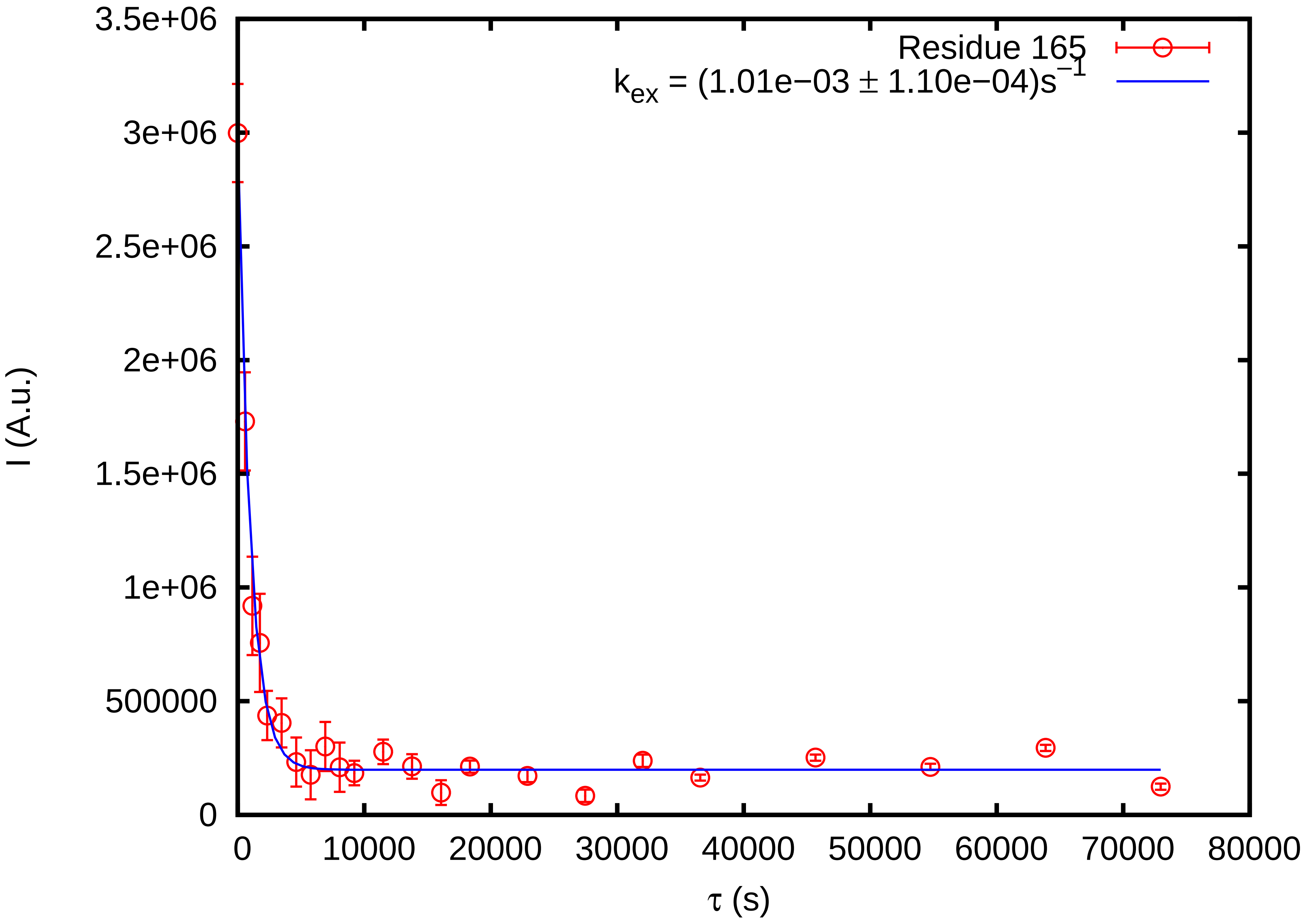

Residue 165
k_{ex} = (1.01e−03 ± 1.10e−04)s^{-1}
I (A.u.)
τ (s)
3.5e+06
3e+06
2.5e+06
2e+06
1.5e+06
1e+06
500000
0
0
10000
20000
30000
40000
50000
60000
70000
80000

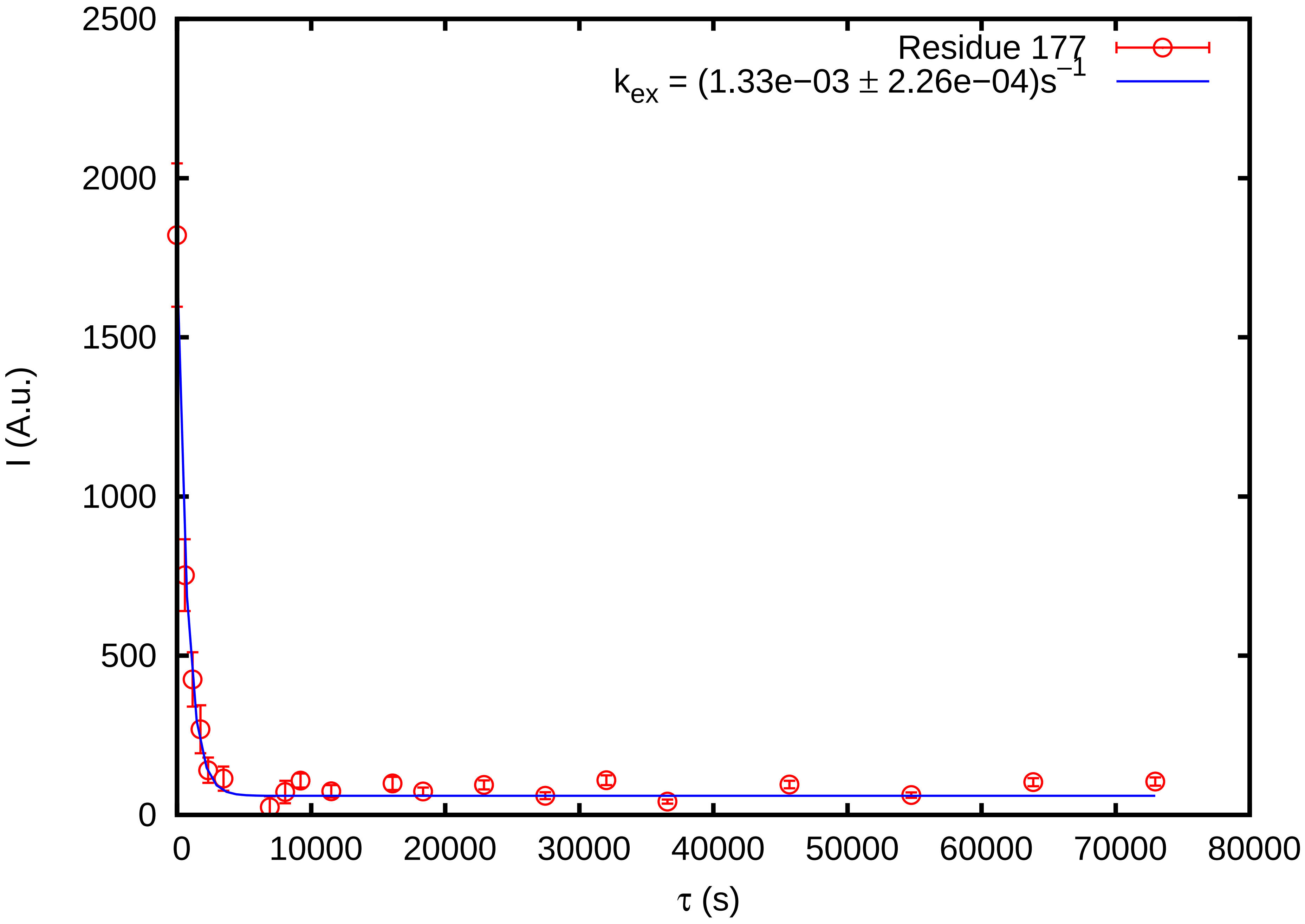

Residue 177
k_{ex} = (1.33e−03 ± 2.26e−04)s^{-1}
2500
2000
1500
1000
500
0
I (A.u.)
0
10000
20000
30000
40000
50000
60000
70000
80000
τ (s)

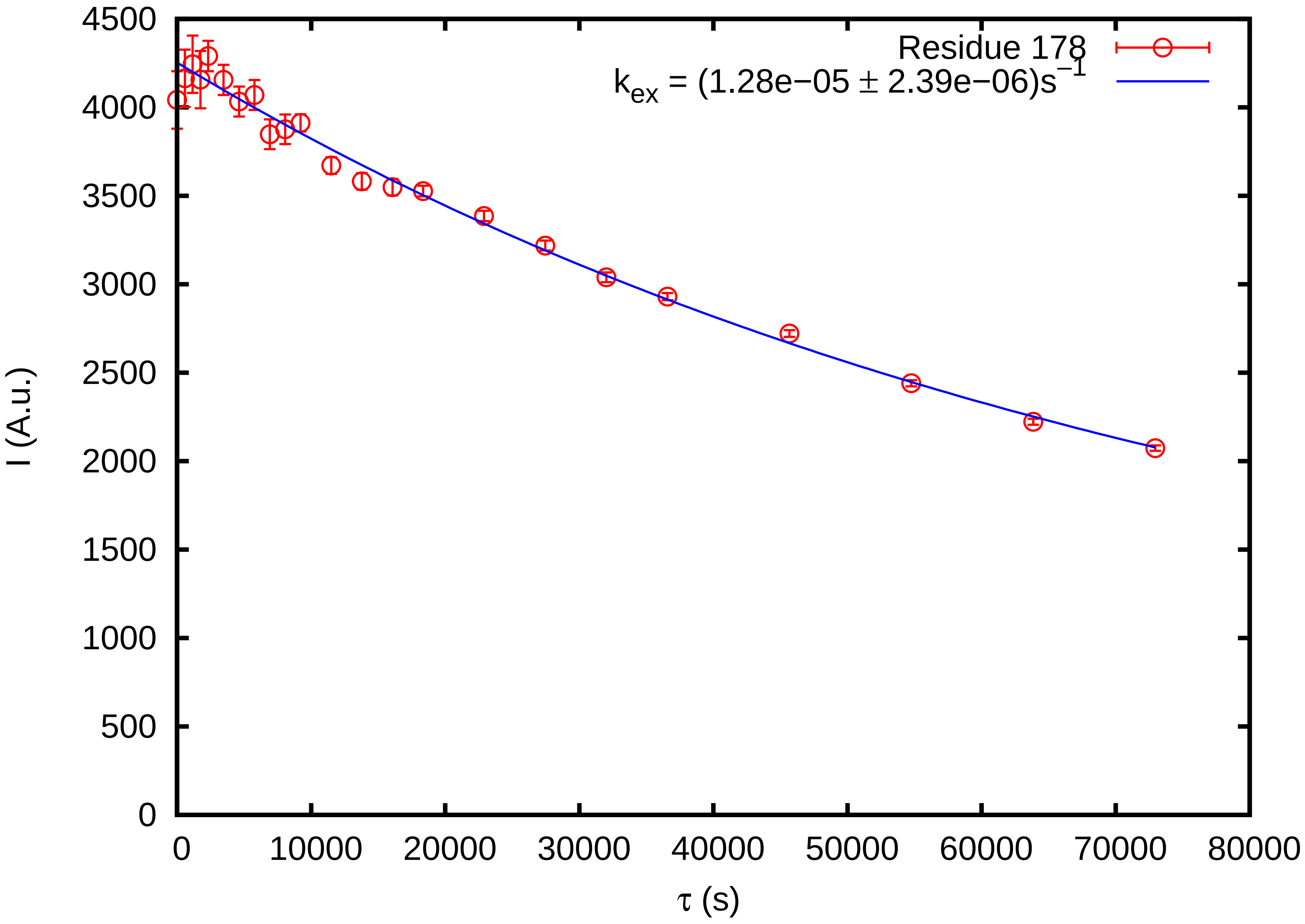

Residue 178
k_{ex} = (1.28e−05 ± 2.39e−06)s^{-1}
I (A.u.)
τ (s)
0
500
1000
1500
2000
2500
3000
3500
4000
4500
0
10000
20000
30000
40000
50000
60000
70000
80000

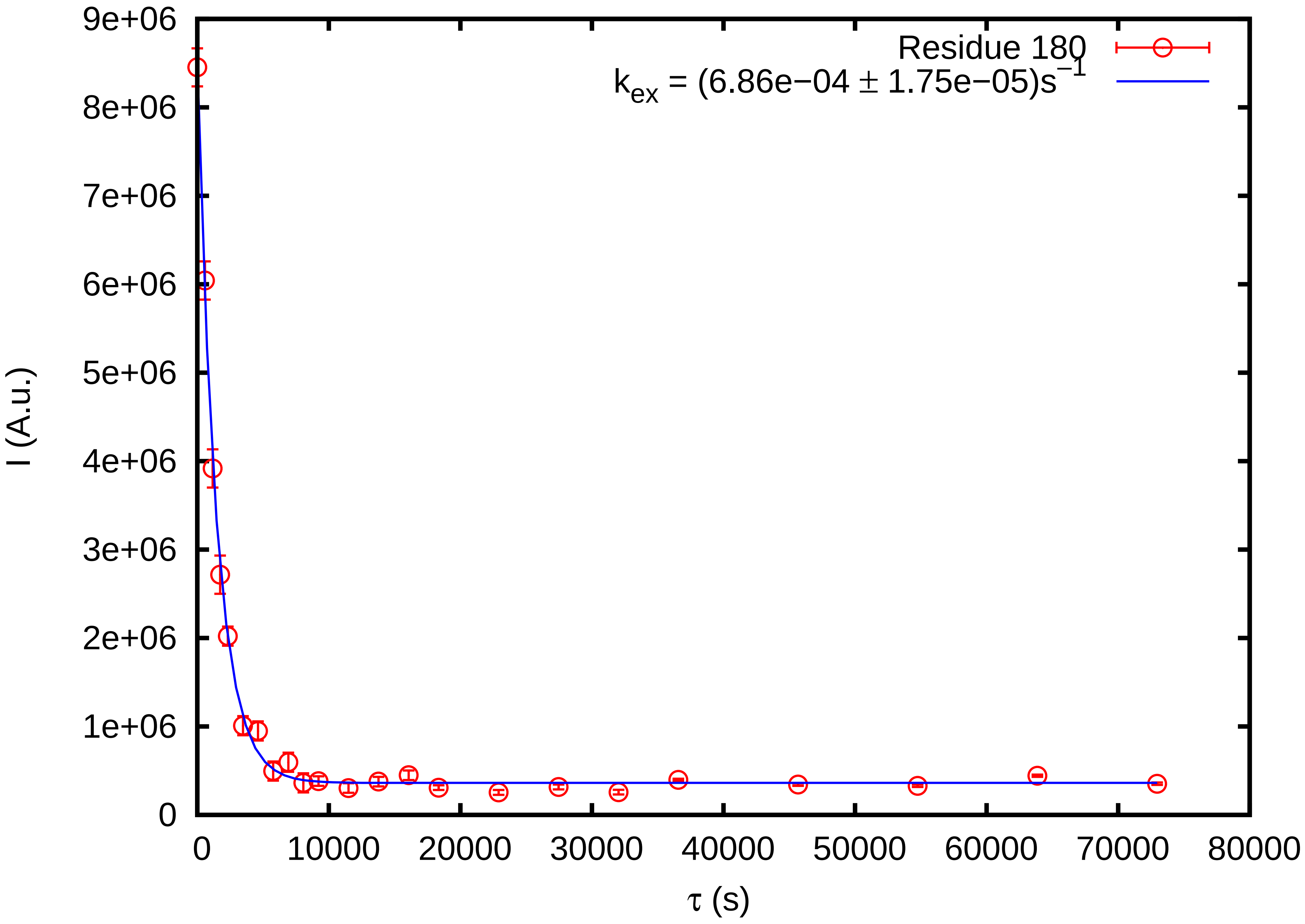

Residue 180
k_{ex} = (6.86e−04 ± 1.75e−05)s^{-1}
9e+06
8e+06
7e+06
6e+06
5e+06
4e+06
3e+06
2e+06
1e+06
0
I (A.u.)
0
10000
20000
30000
40000
50000
60000
70000
80000
τ (s)

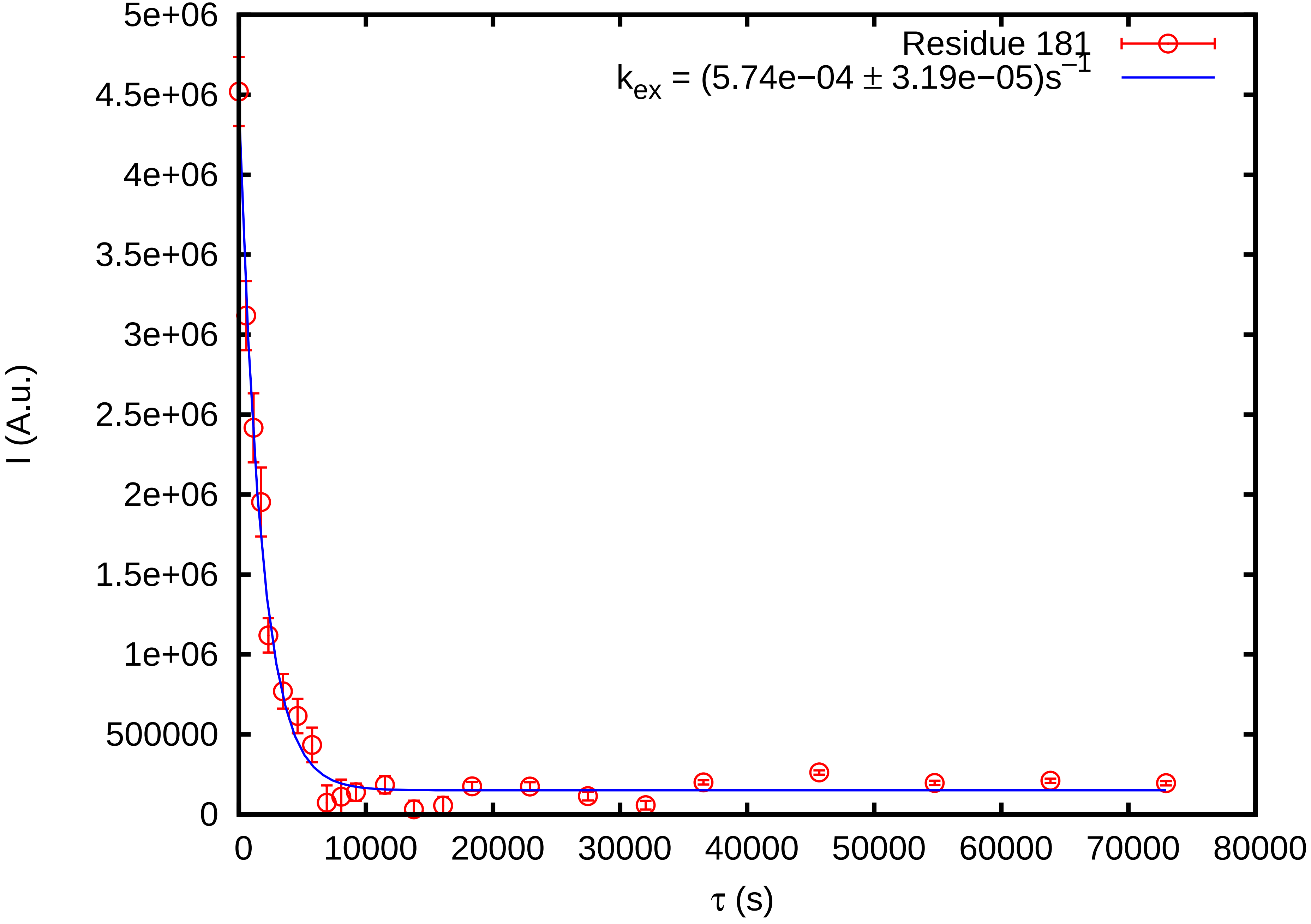

Residue 181
k_{ex} = (5.74e−04 ± 3.19e−05)s^{-1}
I (A.u.)
τ (s)
5e+06
4.5e+06
4e+06
3.5e+06
3e+06
2.5e+06
2e+06
1.5e+06
1e+06
500000
0
10000
20000
30000
40000
50000
60000
70000
80000

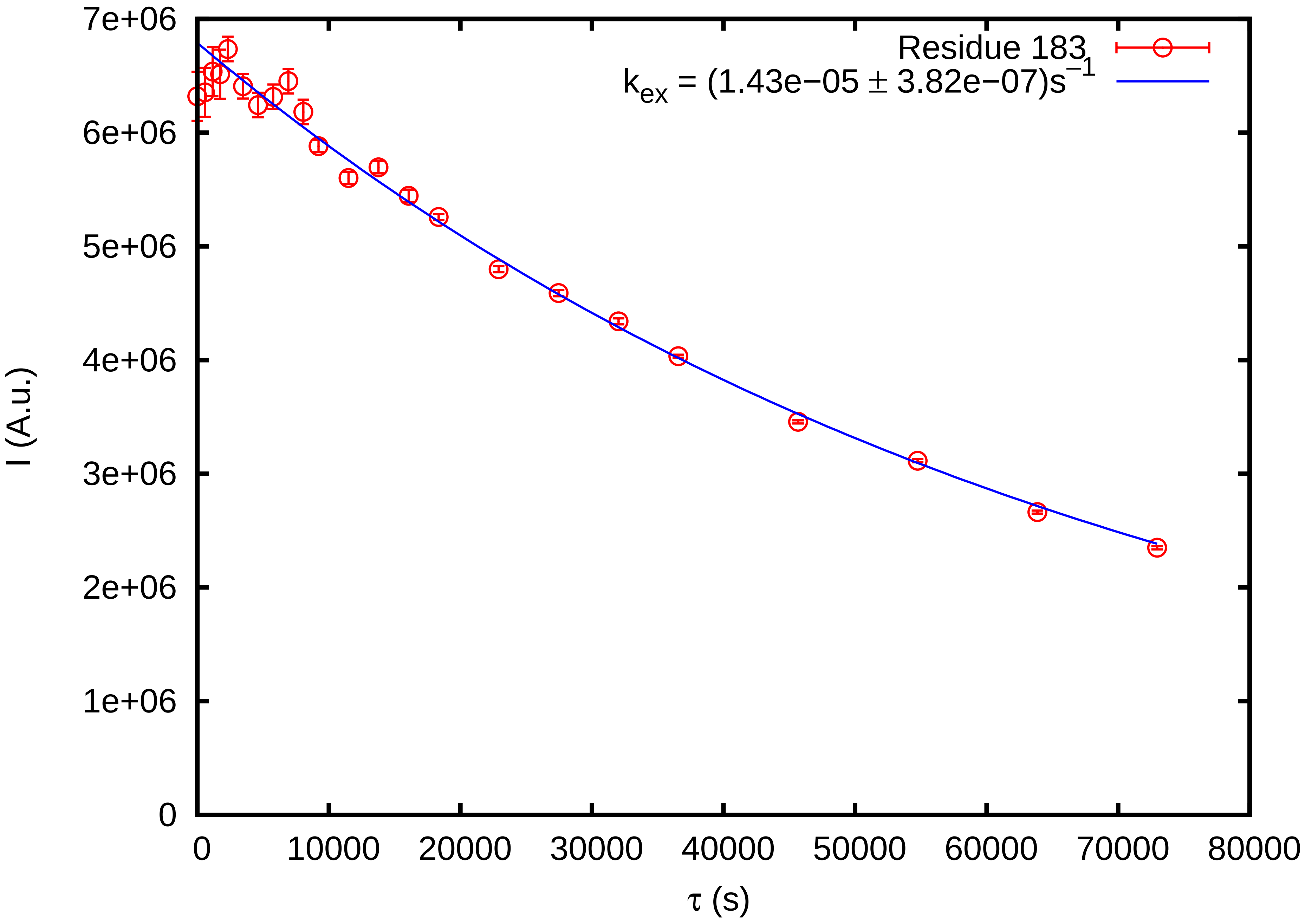

Residue 183
k_{ex} = (1.43e−05 ± 3.82e−07)s^{-1}
I (A.u.)
τ (s)
0
1e+06
2e+06
3e+06
4e+06
5e+06
6e+06
7e+06
0
10000
20000
30000
40000
50000
60000
70000
80000

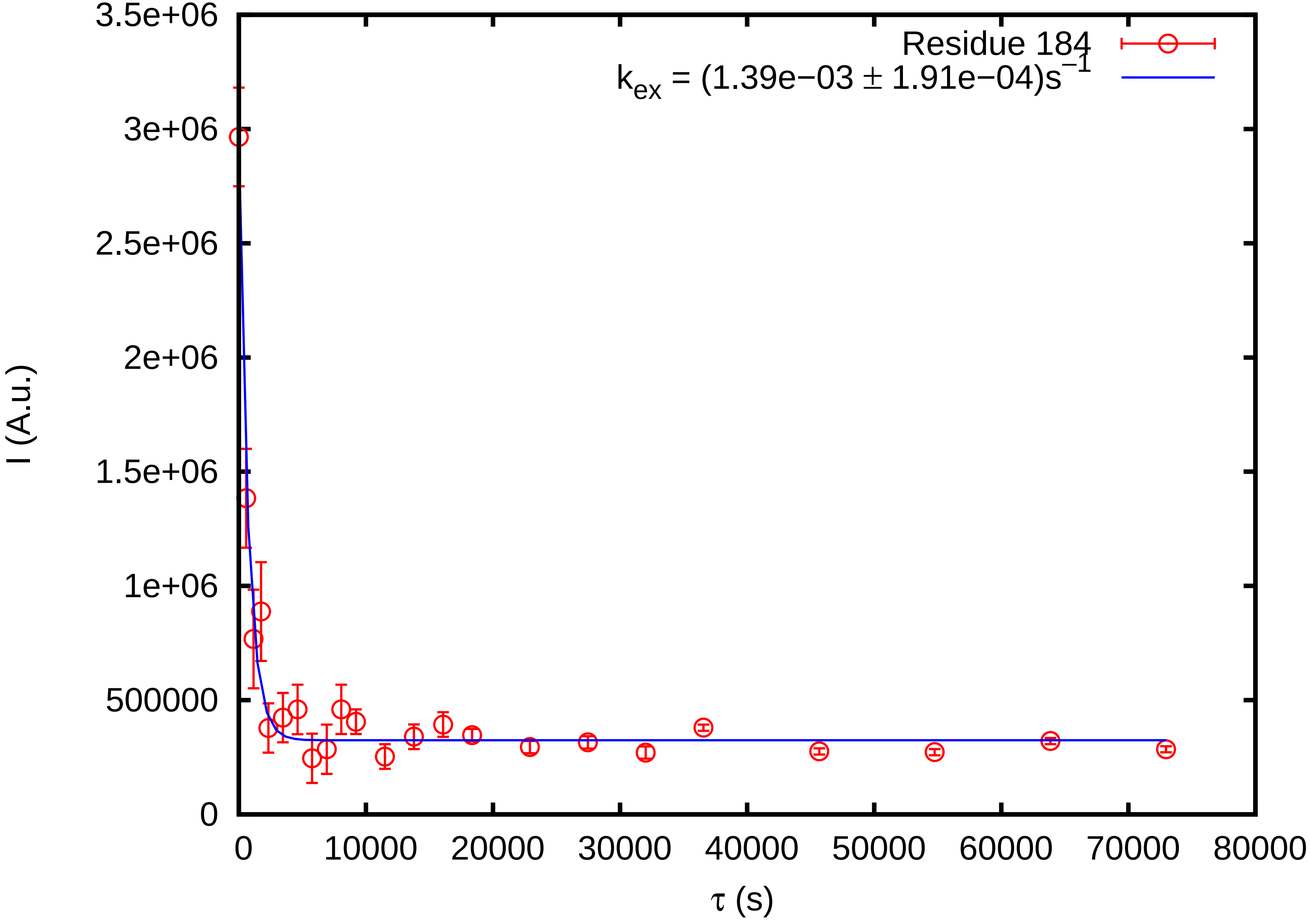

Residue 184
k_{ex} = (1.39e−03 ± 1.91e−04)s^{-1}
I (A.u.)
τ (s)
3.5e+06
3e+06
2.5e+06
2e+06
1.5e+06
1e+06
500000
0
0
10000
20000
30000
40000
50000
60000
70000
80000

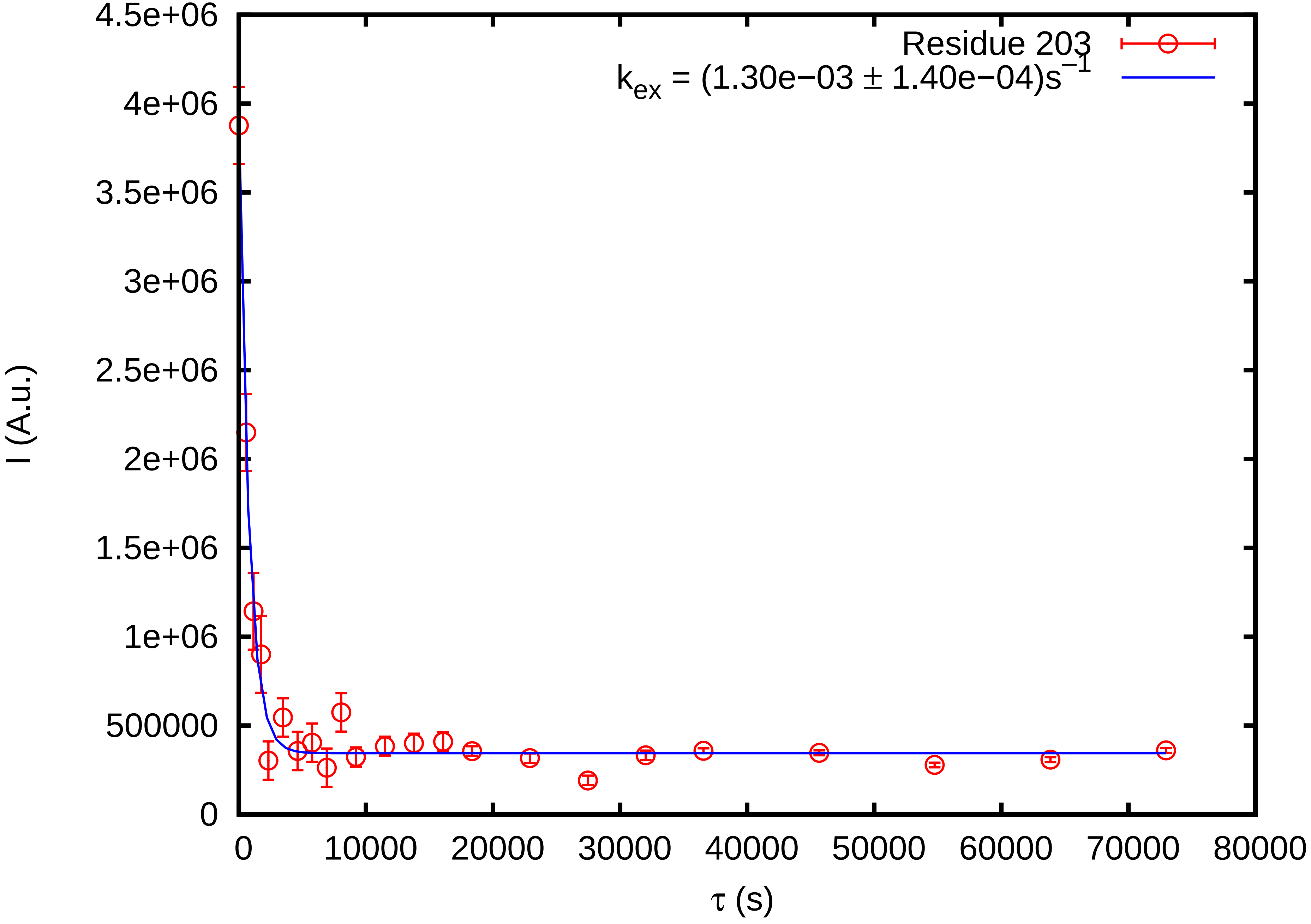

Residue 203
k_{ex} = (1.30e−03 ± 1.40e−04)s^{-1}
I (A.u.)
τ (s)
4.5e+06
4e+06
3.5e+06
3e+06
2.5e+06
2e+06
1.5e+06
1e+06
500000
0
0
10000
20000
30000
40000
50000
60000
70000
80000

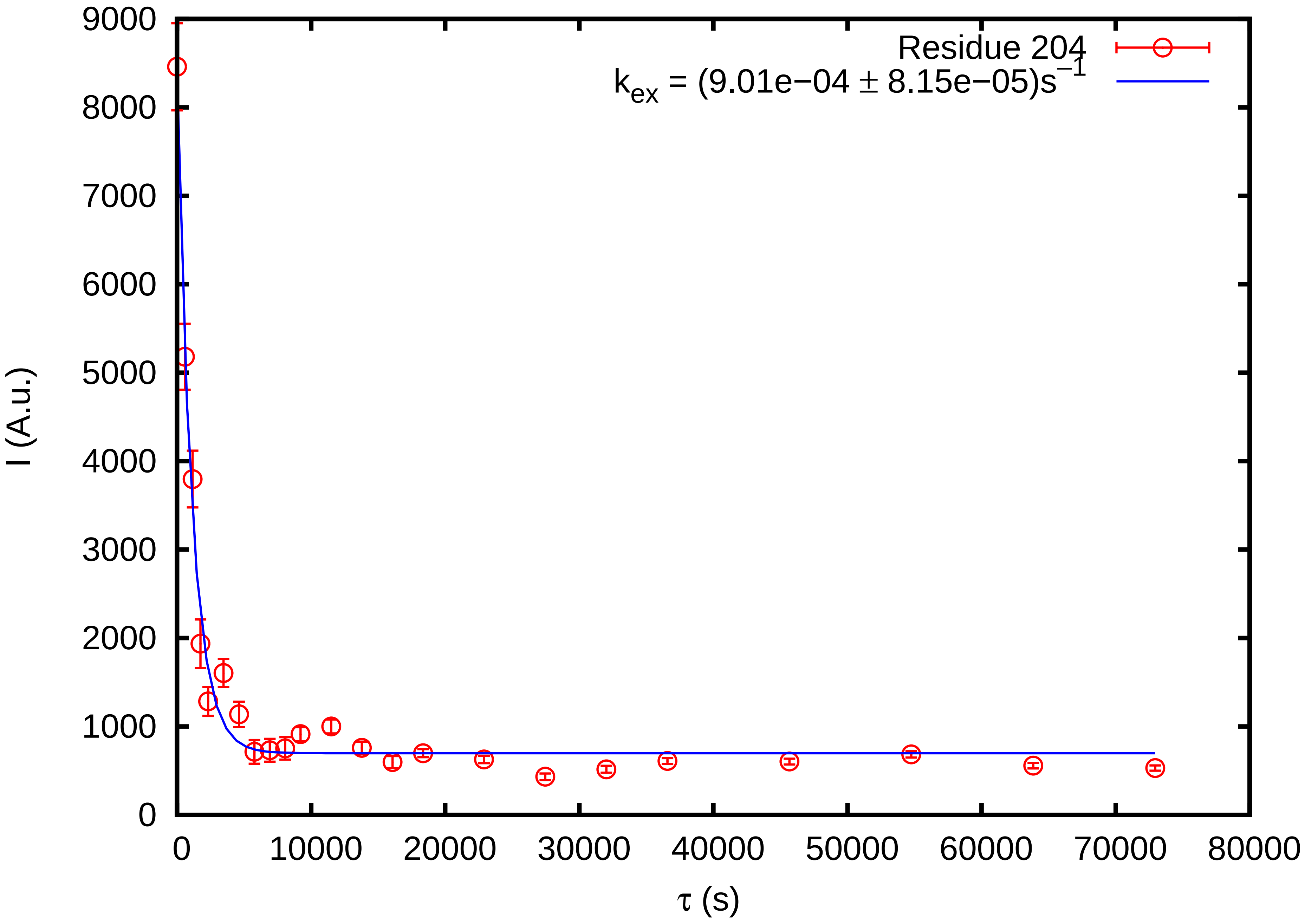

Residue 204
k_{ex} = (9.01e−04 ± 8.15e−05)s^{-1}
I (A.u.)
τ (s)
0
1000
2000
3000
4000
5000
6000
7000
8000
9000
0
10000
20000
30000
40000
50000
60000
70000
80000

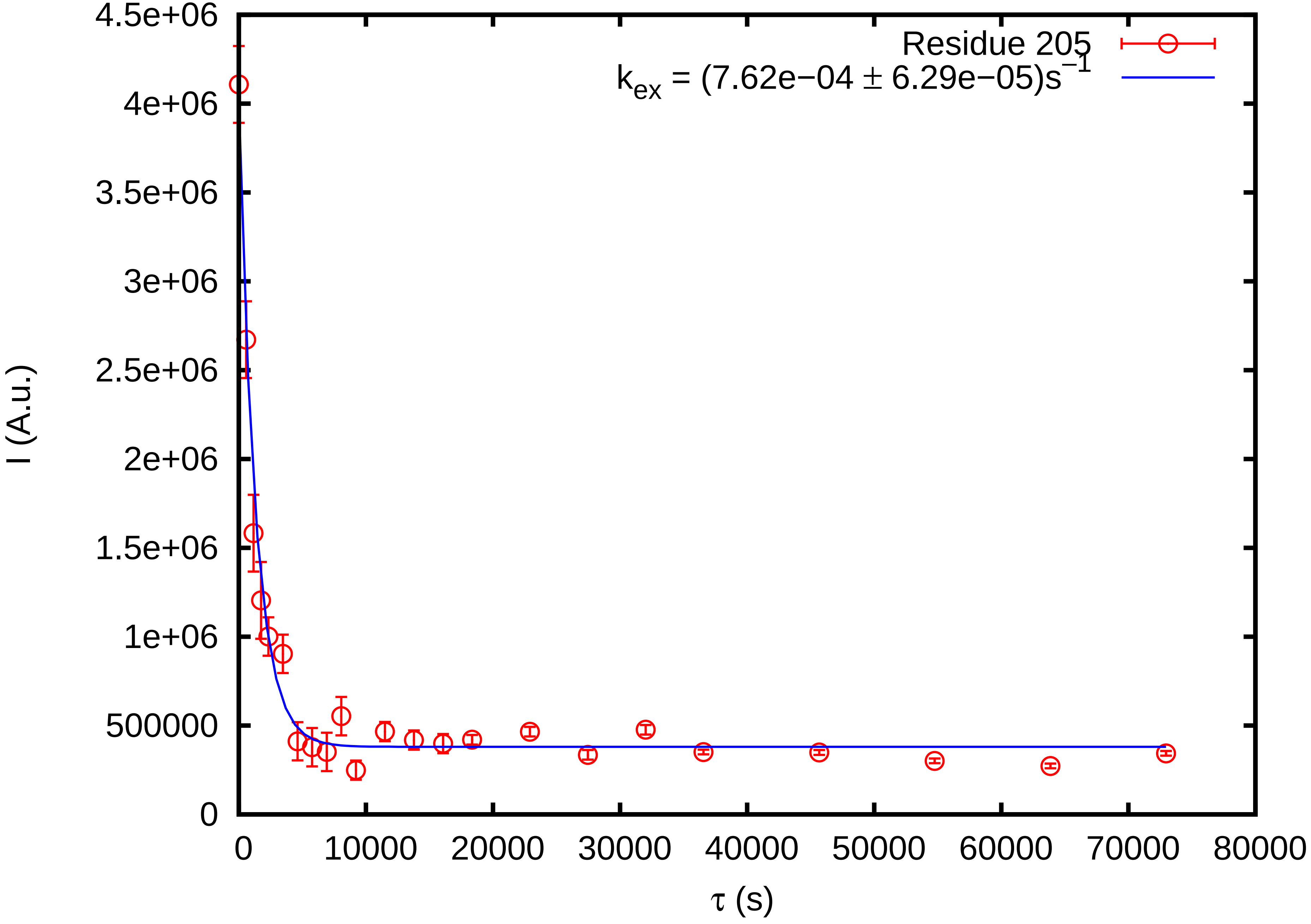

Residue 205
k_ex = (7.62e−04 ± 6.29e−05)s^−1
I (A.u.)
τ (s)
4.5e+06
4e+06
3.5e+06
3e+06
2.5e+06
2e+06
1.5e+06
1e+06
500000
0
10000
20000
30000
40000
50000
60000
70000
80000

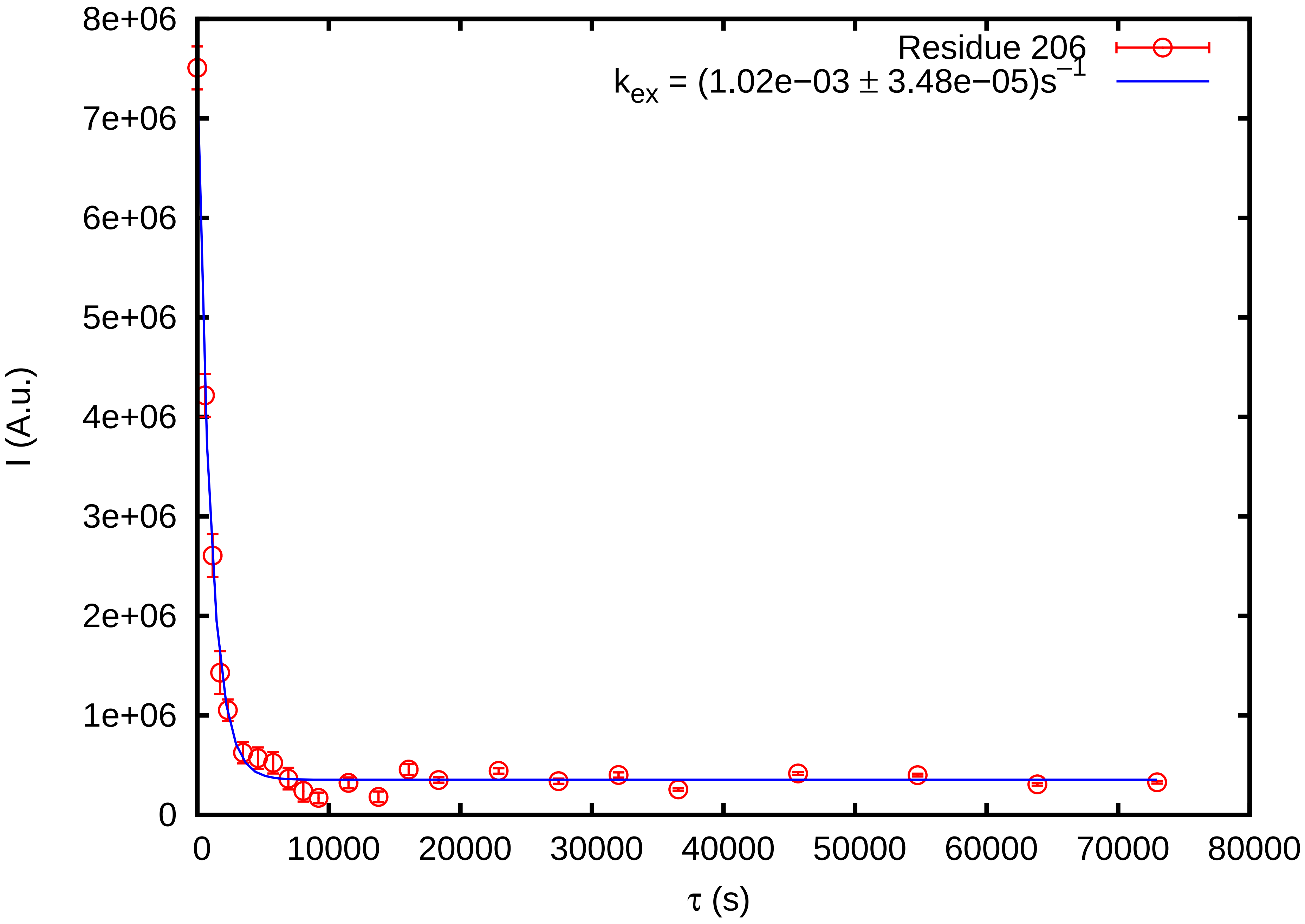

Residue 206
k_{ex} = (1.02e−03 ± 3.48e−05)s^{-1}
I (A.u.)
τ (s)
8e+06
7e+06
6e+06
5e+06
4e+06
3e+06
2e+06
1e+06
0
0
10000
20000
30000
40000
50000
60000
70000
80000

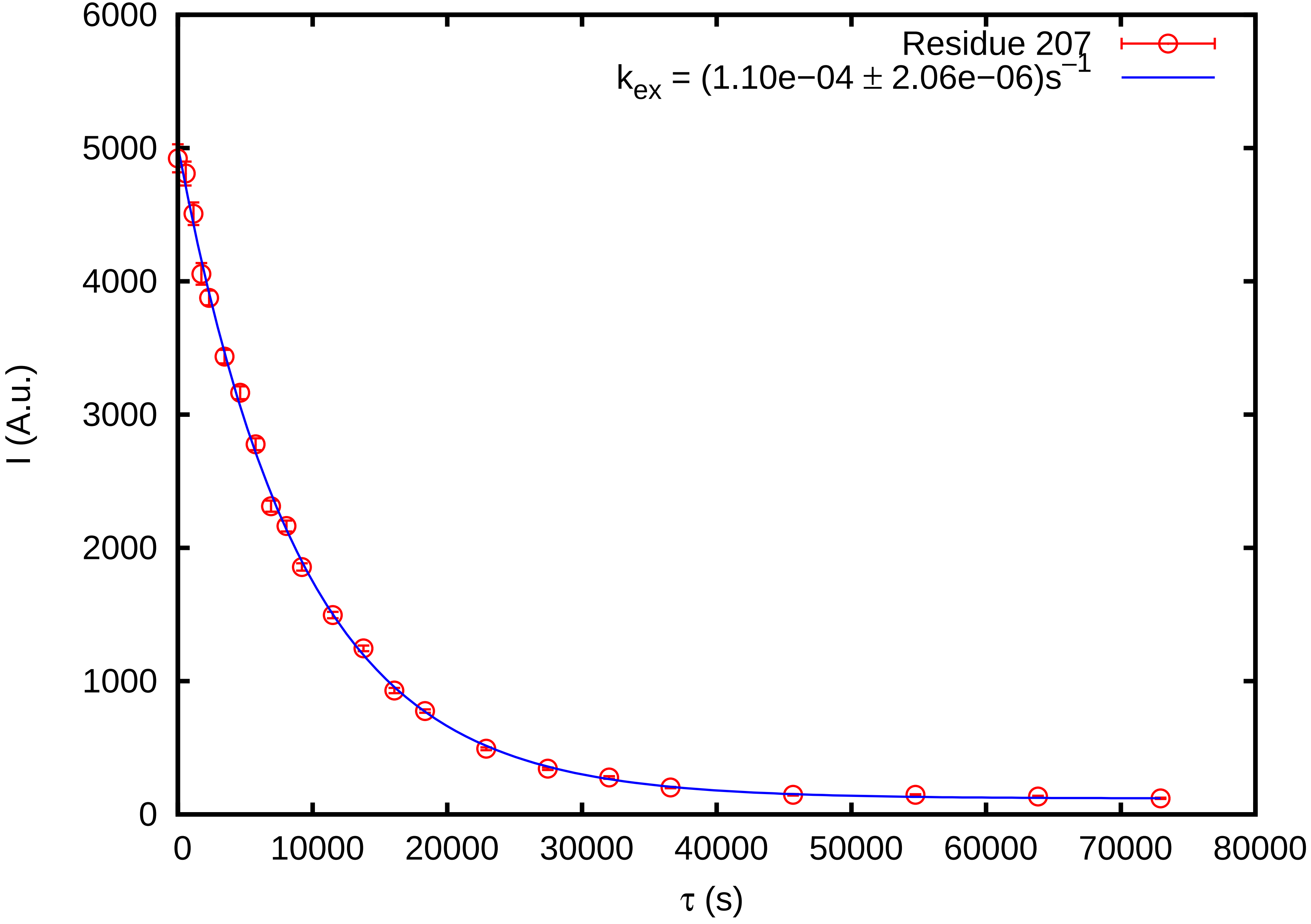

Residue 207
k_{ex} = (1.10e−04 ± 2.06e−06)s^{-1}
I (A.u.)
τ (s)
0
1000
2000
3000
4000
5000
6000
0
10000
20000
30000
40000
50000
60000
70000
80000

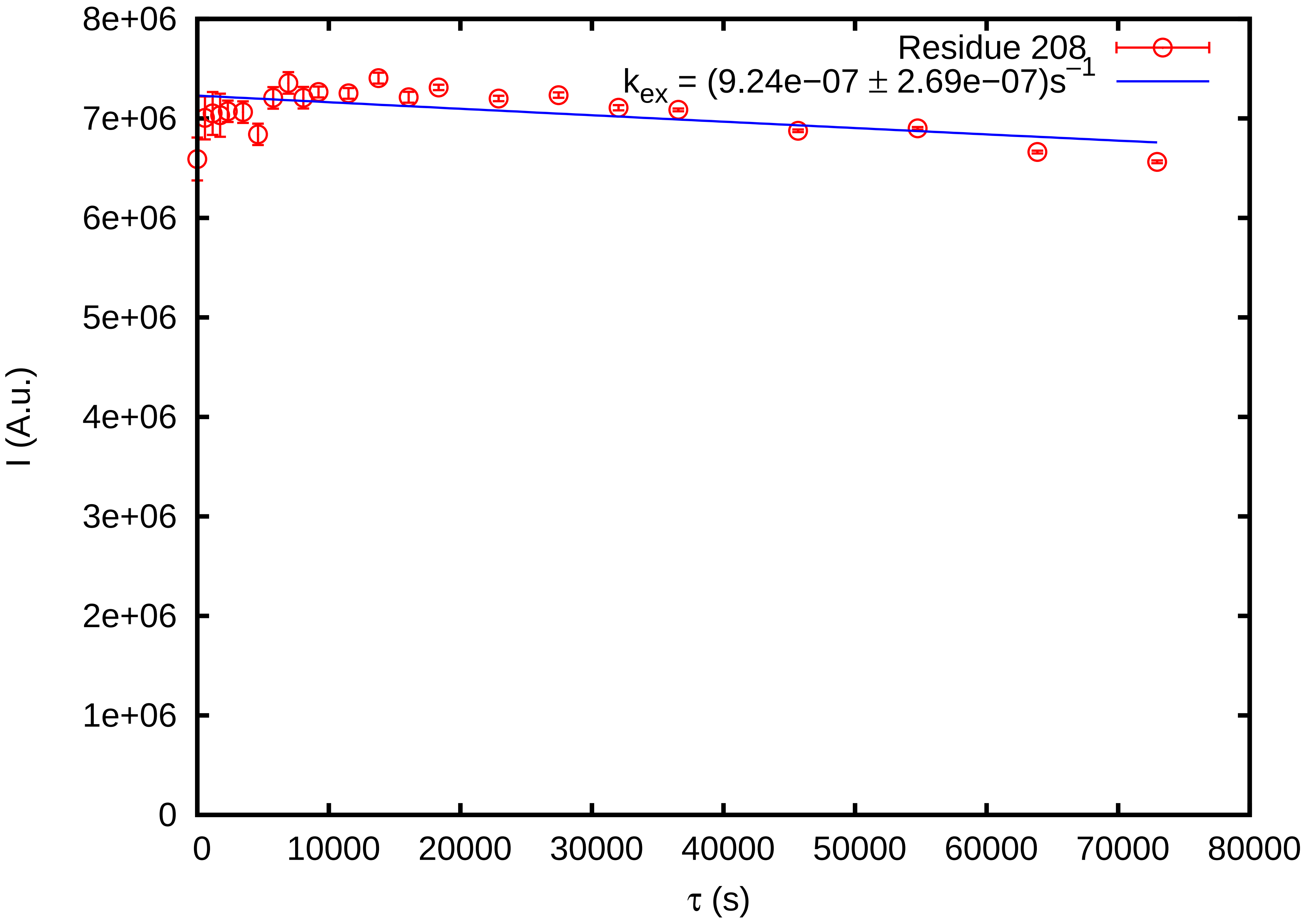
Residue 208
k_ex = (9.24e−07 ± 2.69e−07)s^−1
8e+06
7e+06
6e+06
5e+06
4e+06
3e+06
2e+06
1e+06
0
I (A.u.)
0
10000
20000
30000
40000
50000
60000
70000
80000
τ (s)

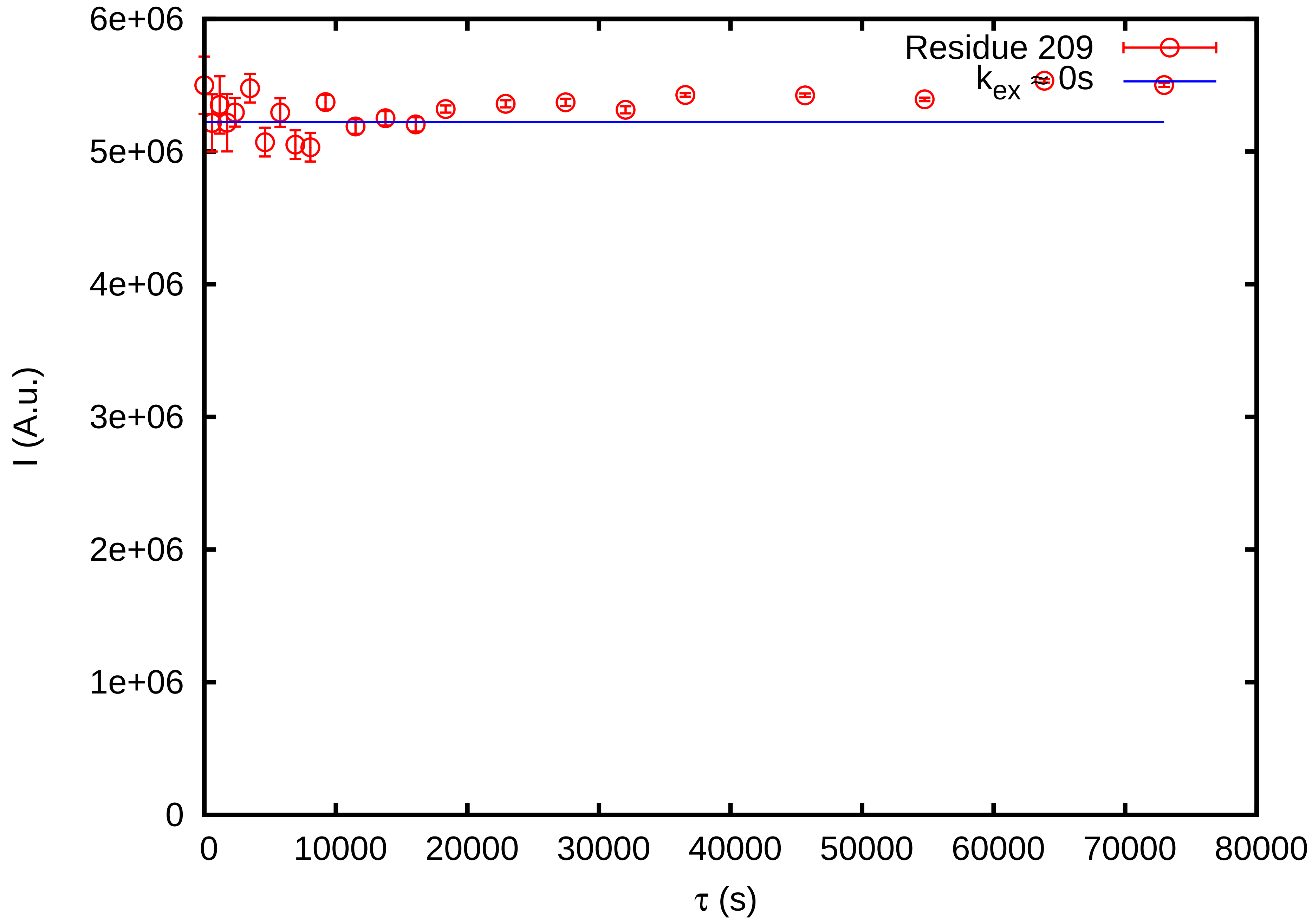

Residue 209
k_{ex} = 0s
I (A.u.)
τ (s)
0
1e+06
2e+06
3e+06
4e+06
5e+06
6e+06
0
10000
20000
30000
40000
50000
60000
70000
80000

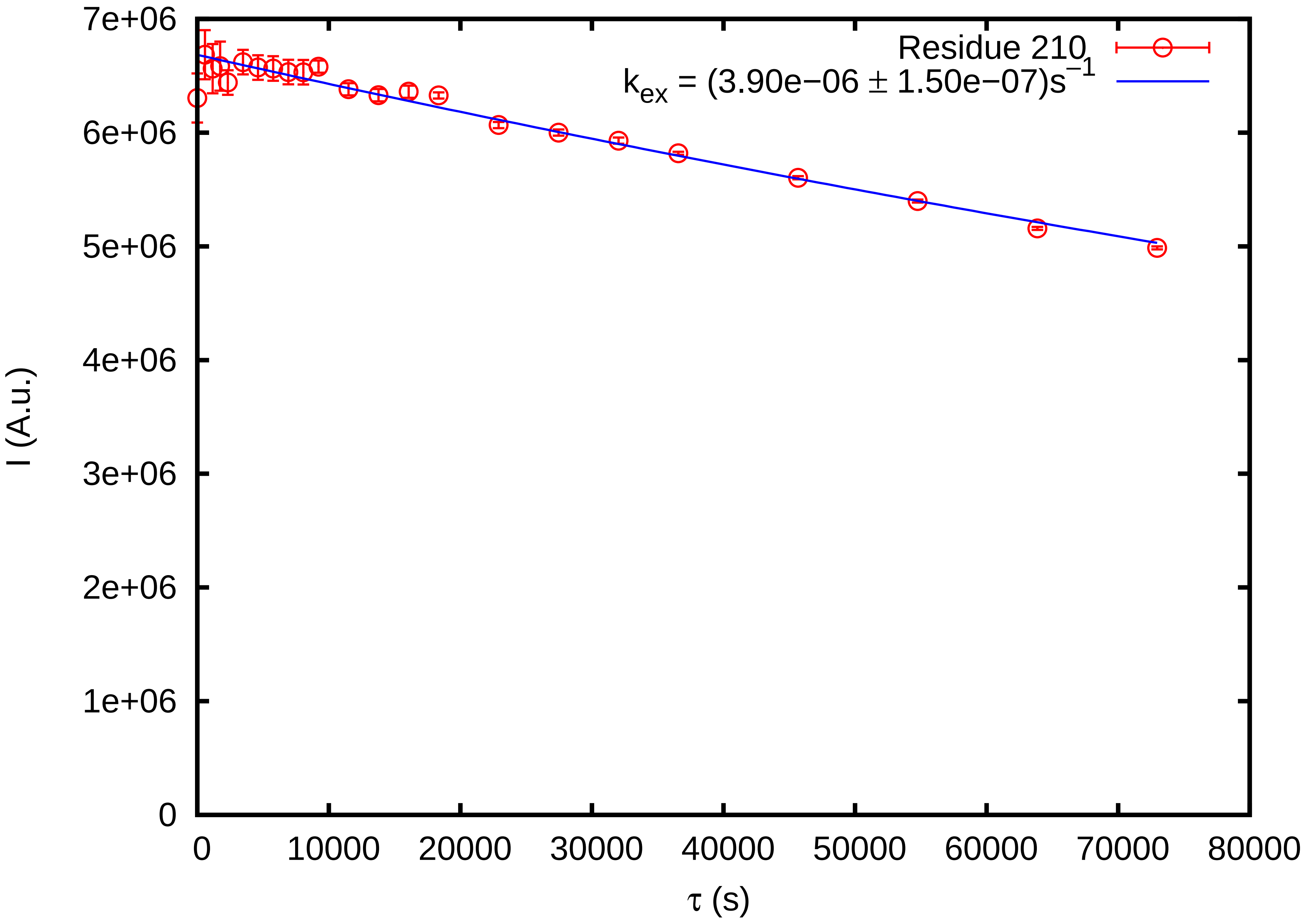

Residue 210
k_{ex} = (3.90e−06 ± 1.50e−07)s^{-1}
I (A.u.)
τ (s)
7e+06
6e+06
5e+06
4e+06
3e+06
2e+06
1e+06
0
0
10000
20000
30000
40000
50000
60000
70000
80000

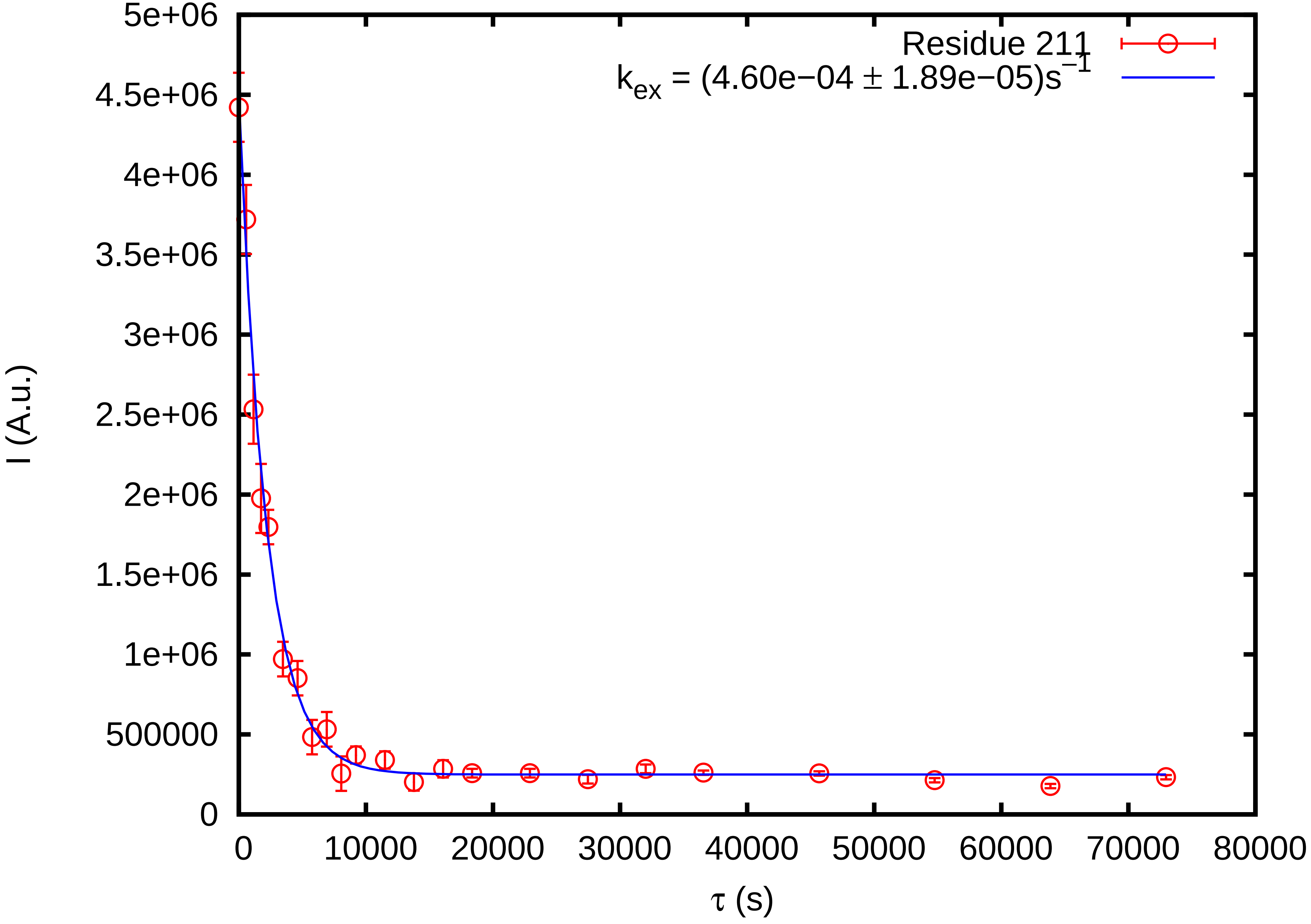

Residue 211
k_{ex} = (4.60e−04 ± 1.89e−05)s^{-1}
I (A.u.)
τ (s)
5e+06
4.5e+06
4e+06
3.5e+06
3e+06
2.5e+06
2e+06
1.5e+06
1e+06
500000
0
10000
20000
30000
40000
50000
60000
70000
80000

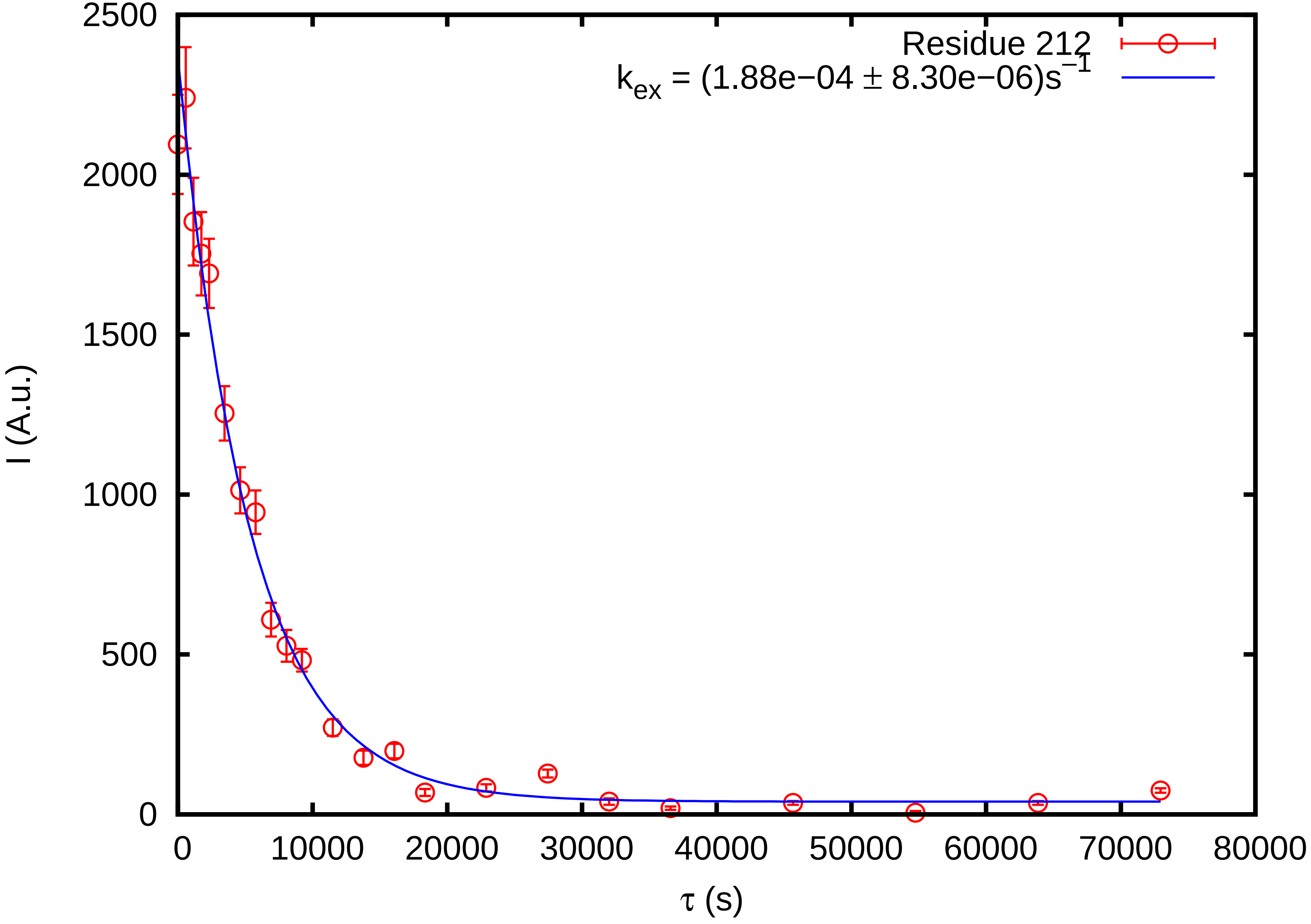

Residue 212
k_{ex} = (1.88e−04 ± 8.30e−06)s^{-1}
I (A.u.)
τ (s)
2500
2000
1500
1000
500
0
0
10000
20000
30000
40000
50000
60000
70000
80000

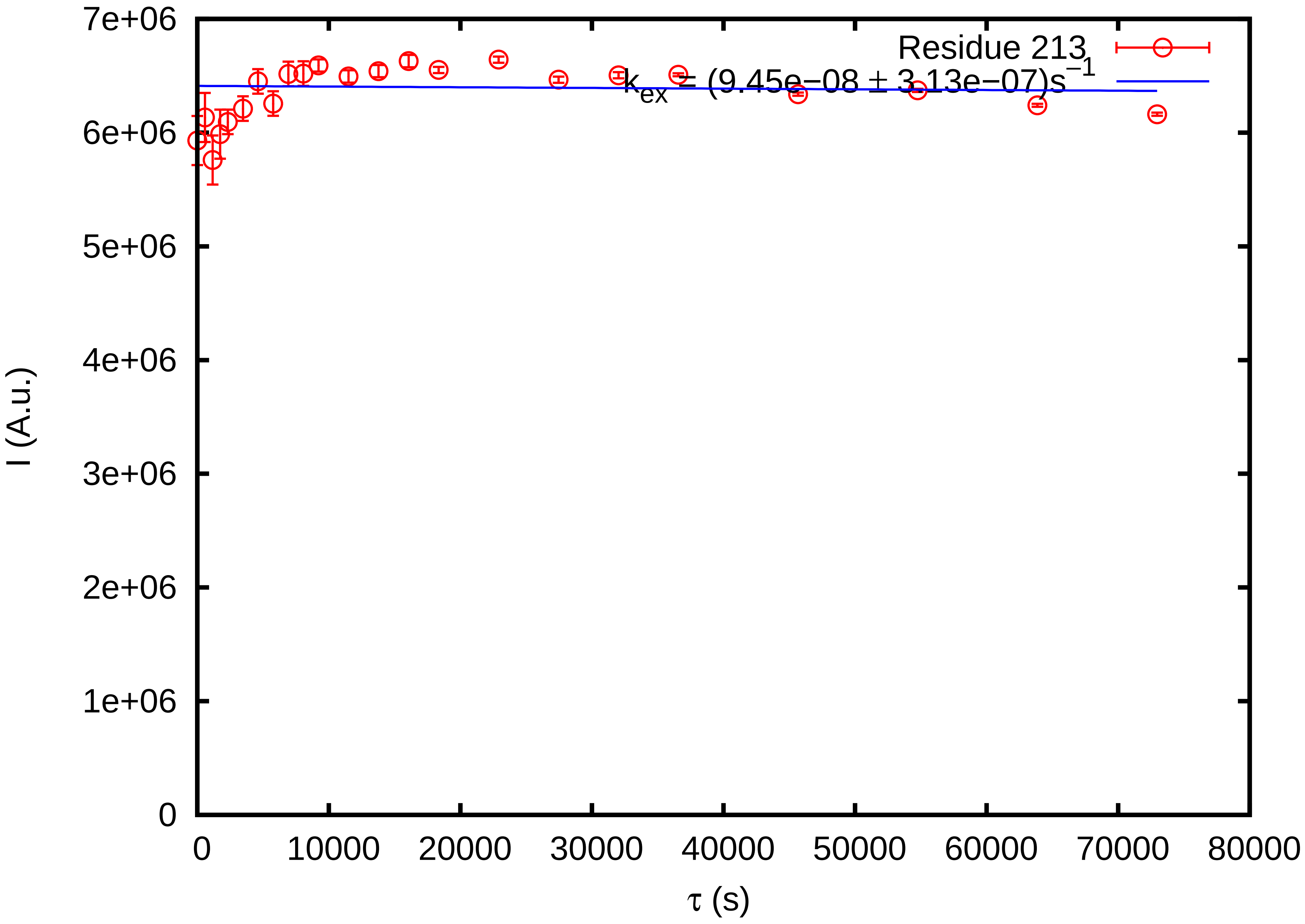
Residue 213
k_{ex} = (9.45e−08 ± 3.13e−07)s^{-1}
I (A.u.)
τ (s)
7e+06
6e+06
5e+06
4e+06
3e+06
2e+06
1e+06
0
0
10000
20000
30000
40000
50000
60000
70000
80000

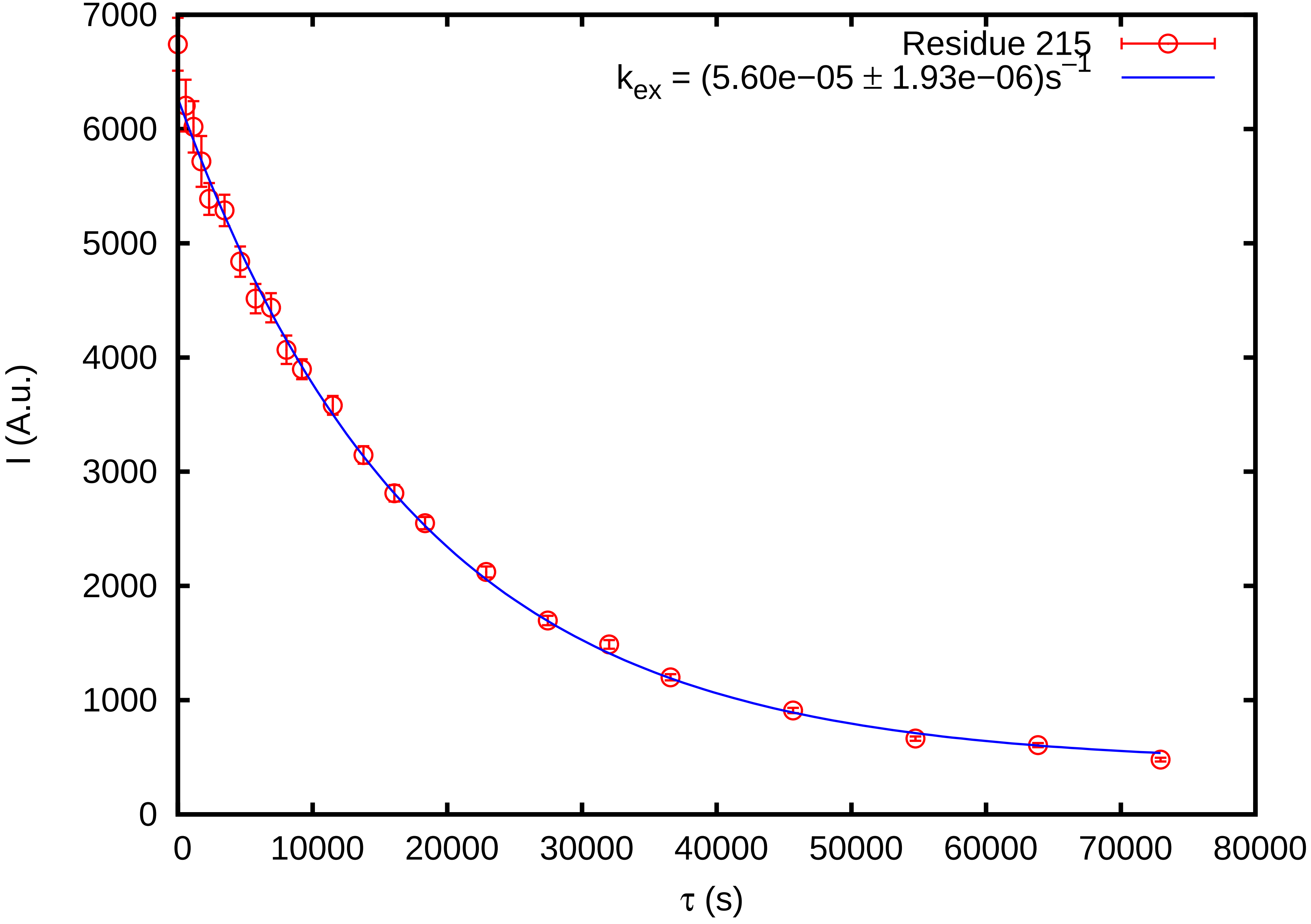

Residue 215
k_ex = (5.60e−05 ± 1.93e−06)s^−1
I (A.u.)
τ (s)
0
1000
2000
3000
4000
5000
6000
7000
0
10000
20000
30000
40000
50000
60000
70000
80000

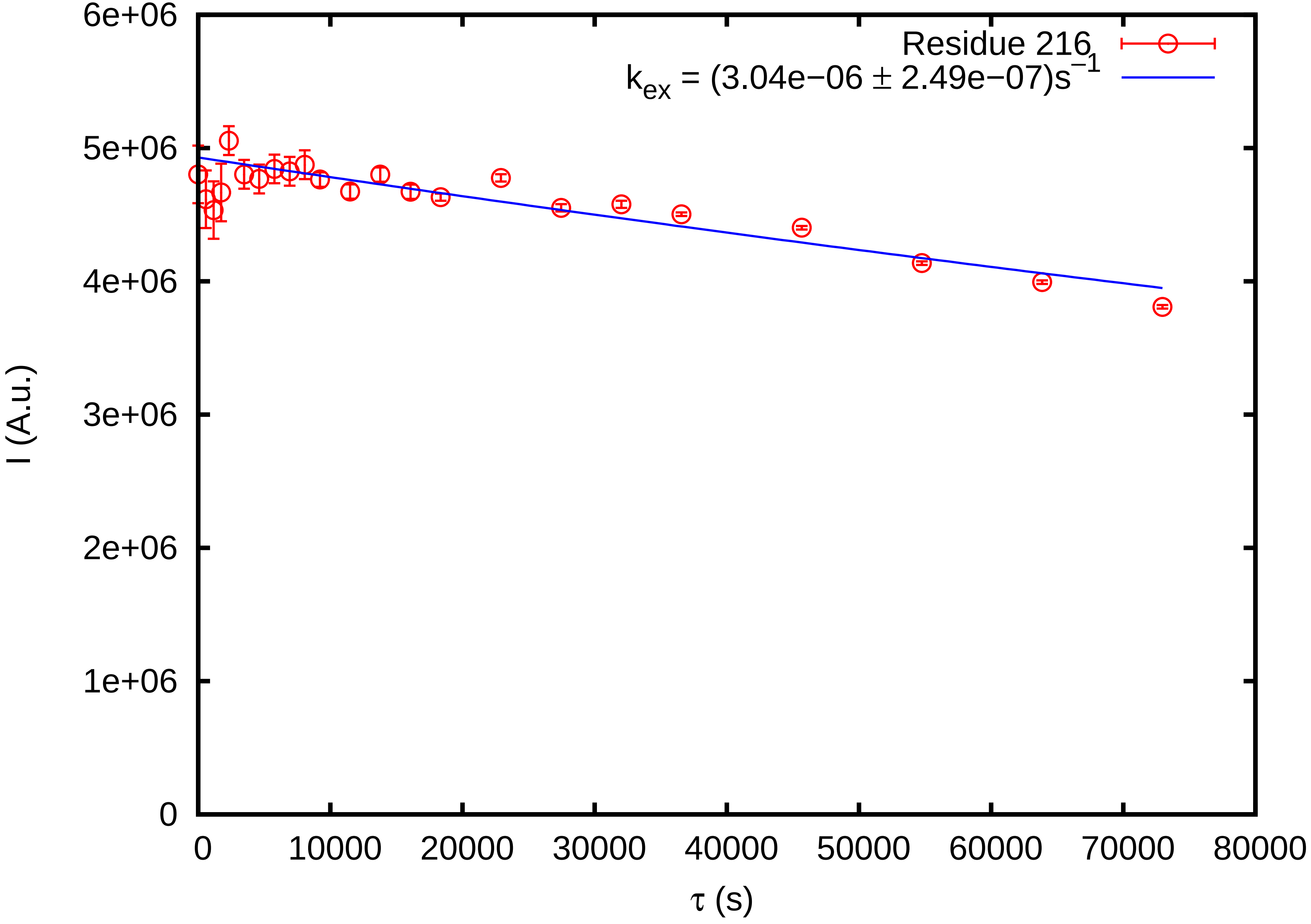

Residue 216
$k_{ex} = (3.04e-06 \pm 2.49e-07)s^{-1}$
6e+06
5e+06
4e+06
3e+06
2e+06
1e+06
0
I (A.u.)
0
10000
20000
30000
40000
50000
60000
70000
80000
τ (s)

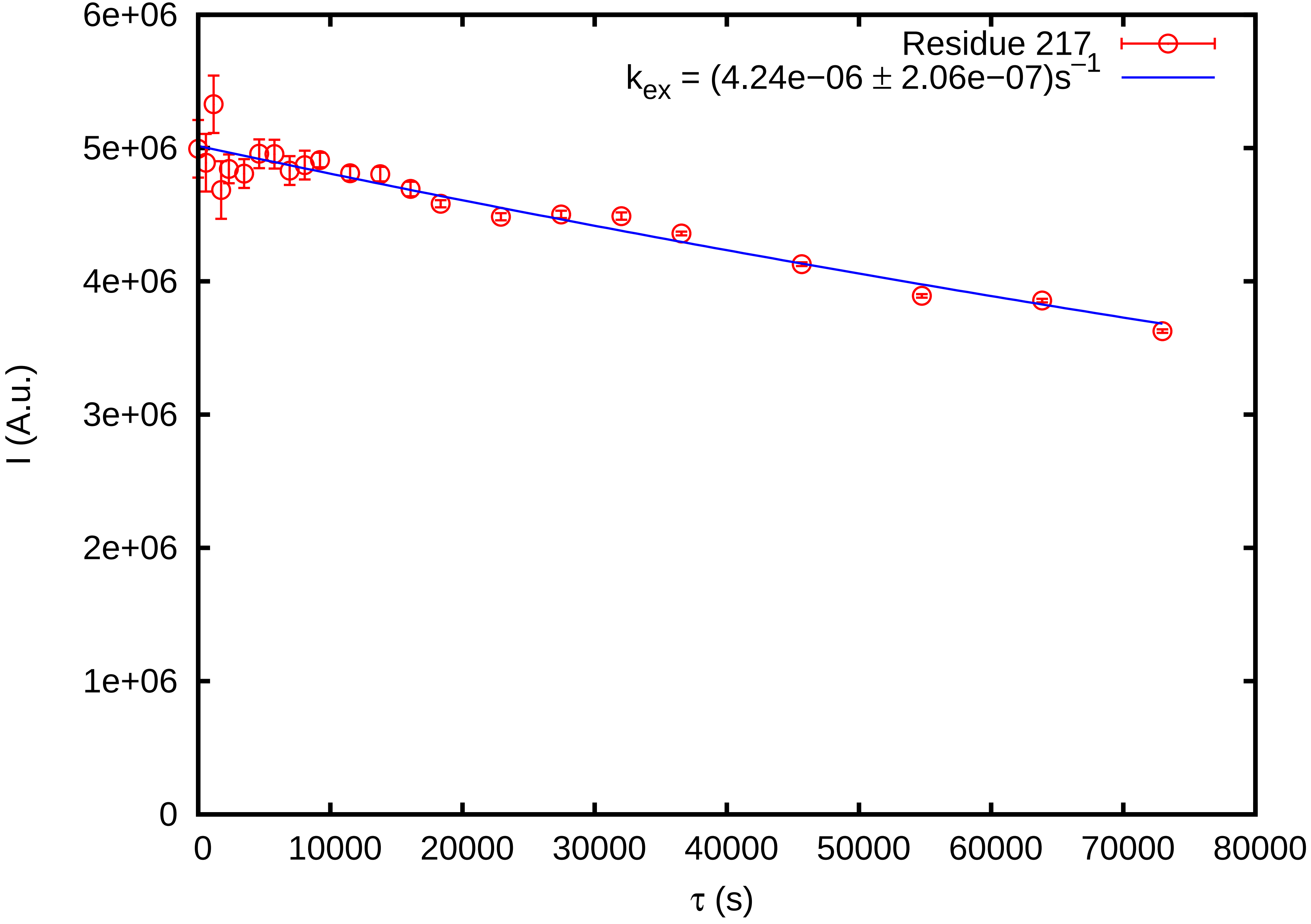

Residue 217
k_{ex} = (4.24e−06 ± 2.06e−07)s^{-1}
I (A.u.)
τ (s)
6e+06
5e+06
4e+06
3e+06
2e+06
1e+06
0
0
10000
20000
30000
40000
50000
60000
70000
80000
