# Supplementary material for: Interplay of buried histidine protonation and protein stability in prion misfolding
Source: Sci Rep. 2017 Apr 13;7:882. doi: 10.1038/s41598-017-00954-7 (PMC5429843; doi:10.1038/s41598-017-00954-7)
Supplement: Supplementary file 5 — Supplementary Figure S7 [file 41598_2017_954_MOESM5_ESM.pdf]

Interplay of buried histidine protonation and protein stability in prion misfolding

Supplementary Figure S7

A. Malevanets[1], P. Andrew Chong[1,2], D.F. Hansen[2,3,+], P. Rizk[1,2], Y. Sun[4], H. Lin[1], R. Muhandiram[2,3], A. Chakrabartty[4], Lewis E. Kay[1,2,3,5], J.D. Forman-Kay[1,2,**] and Shoshana J. Wodak[1,2,3§**]

[1]Program in Molecular Structure and Function, Hospital for Sick Children, 555 University Ave., Toronto, ON, Canada M5G 0A4

[2]Department of Biochemistry and [3]Department of Molecular Genetics, University of Toronto, Toronto, ON, Canada M5S 1A8

[4]Department of Medical Biophysics, University of Toronto, Toronto, ON, Canada M5G 2M9

[5]Department of Chemistry, University of Toronto, Toronto, ON, Canada, M5S 3H6

[+]Current address: University College London, Division of Biosciences, London WC1E 6BT UK

[§]Current address: VIB Structural Biology Research Center, VUB, Pleinlaan 2 1050 Brussels, Belgium

** Corresponding authors.

Figures S7. Hydrogen exchange decay curves for rabbit PrP at pH 5.

Decay curves of peak intensities in 15N-1H correlation maps (O) vertical bars represent the uncertainty of the obtained intensity. The intensities were obtained from a simultaneous analysis and global fit of all spectra with different tau as described previously (Hansen et al (2007)). The solid lines are obtained from best-fits of an exponential decay function $I(t)=I0*\exp(-k_{ex}*tau)$ + offset to the data shown. For peaks with a decay rate slower than ~1e-5 /s the offset and the k_{ex} rate are strongly correlated and the offset was therefore fixed to 0 for these peaks.

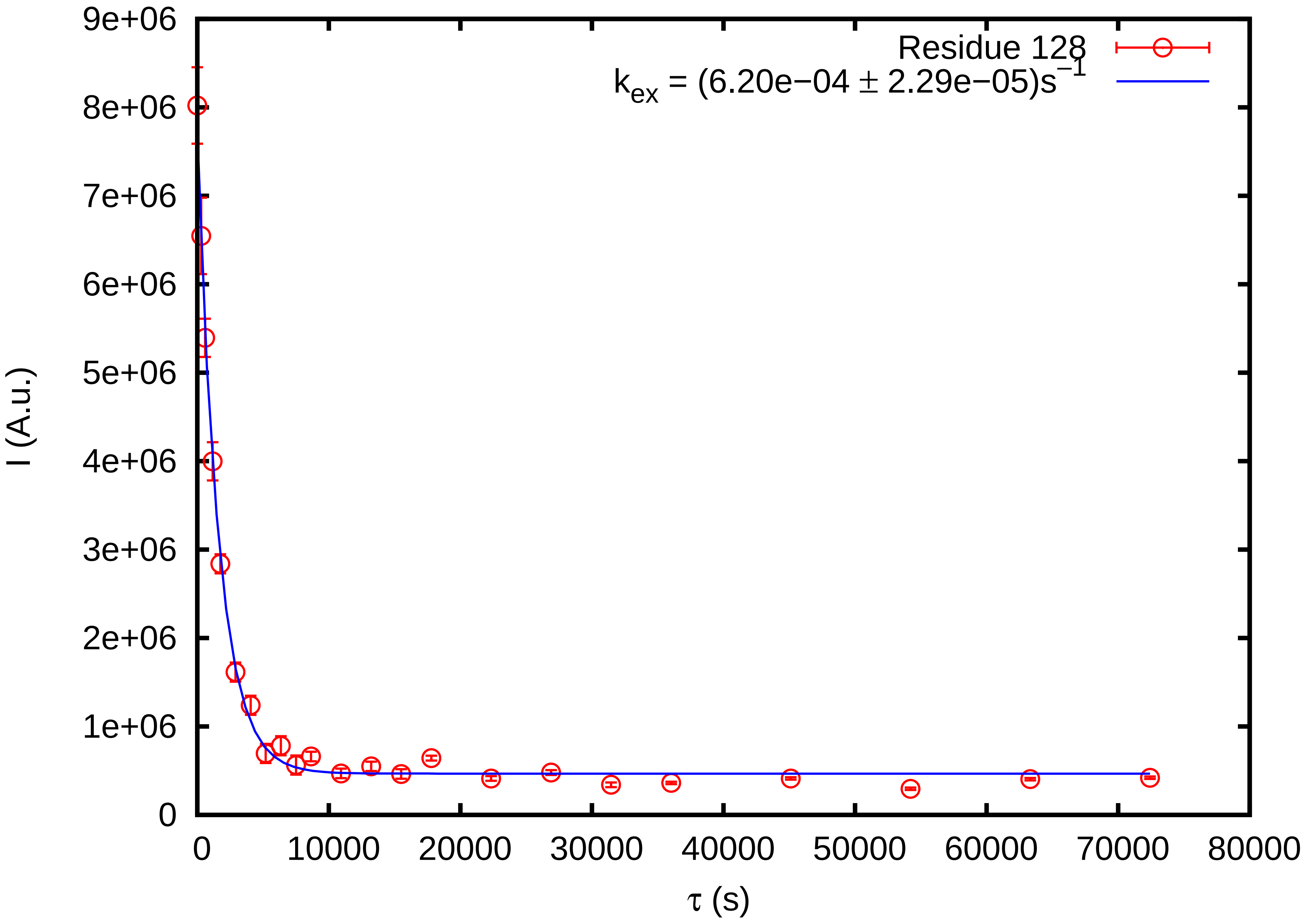

Residue 128
k_{ex} = (6.20e−04 ± 2.29e−05)s^{-1}
I (A.u.)
τ (s)
9e+06
8e+06
7e+06
6e+06
5e+06
4e+06
3e+06
2e+06
1e+06
0
0
10000
20000
30000
40000
50000
60000
70000
80000

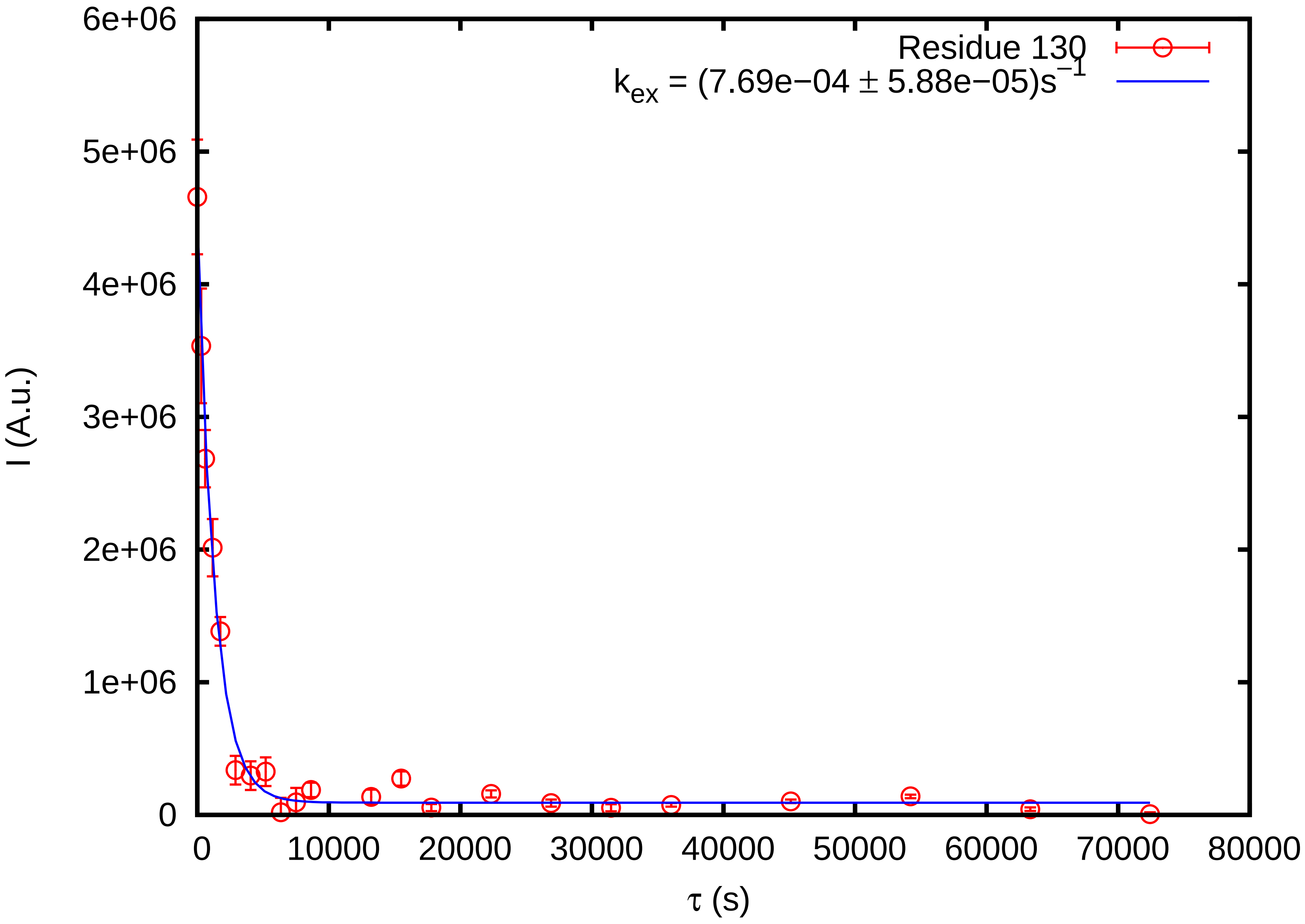

Residue 130
k_ex = (7.69e−04 ± 5.88e−05)s^−1
I (A.u.)
τ (s)
6e+06
5e+06
4e+06
3e+06
2e+06
1e+06
0
0
10000
20000
30000
40000
50000
60000
70000
80000

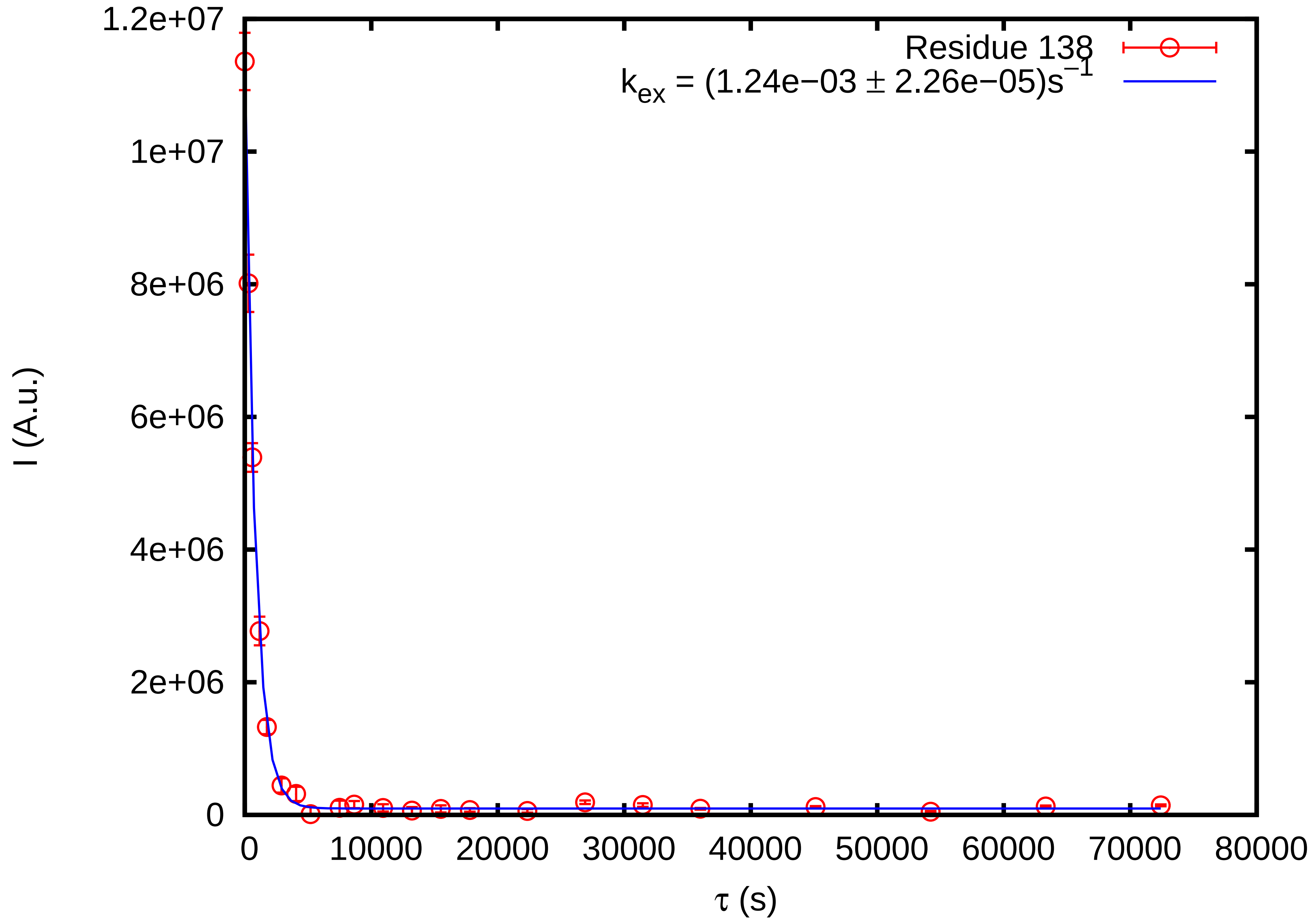

Residue 138
k_ex = (1.24e−03 ± 2.26e−05)s^−1
1.2e+07
1e+07
8e+06
6e+06
4e+06
2e+06
0
I (A.u.)
0
10000
20000
30000
40000
50000
60000
70000
80000
τ (s)

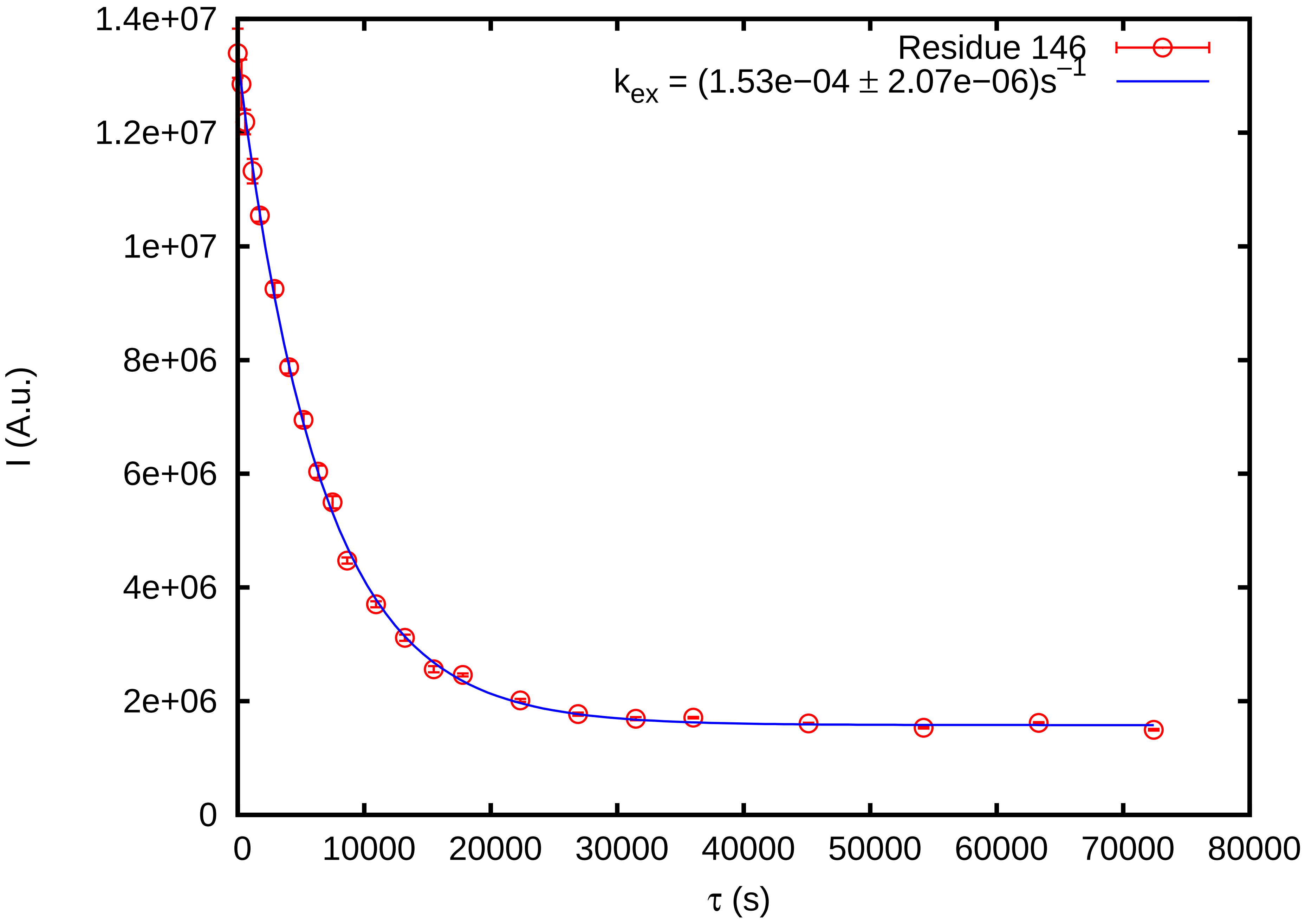

Residue 146
k_ex = (1.53e−04 ± 2.07e−06)s^−1
1.4e+07
1.2e+07
1e+07
8e+06
6e+06
4e+06
2e+06
0
I (A.u.)
0
10000
20000
30000
40000
50000
60000
70000
80000
τ (s)

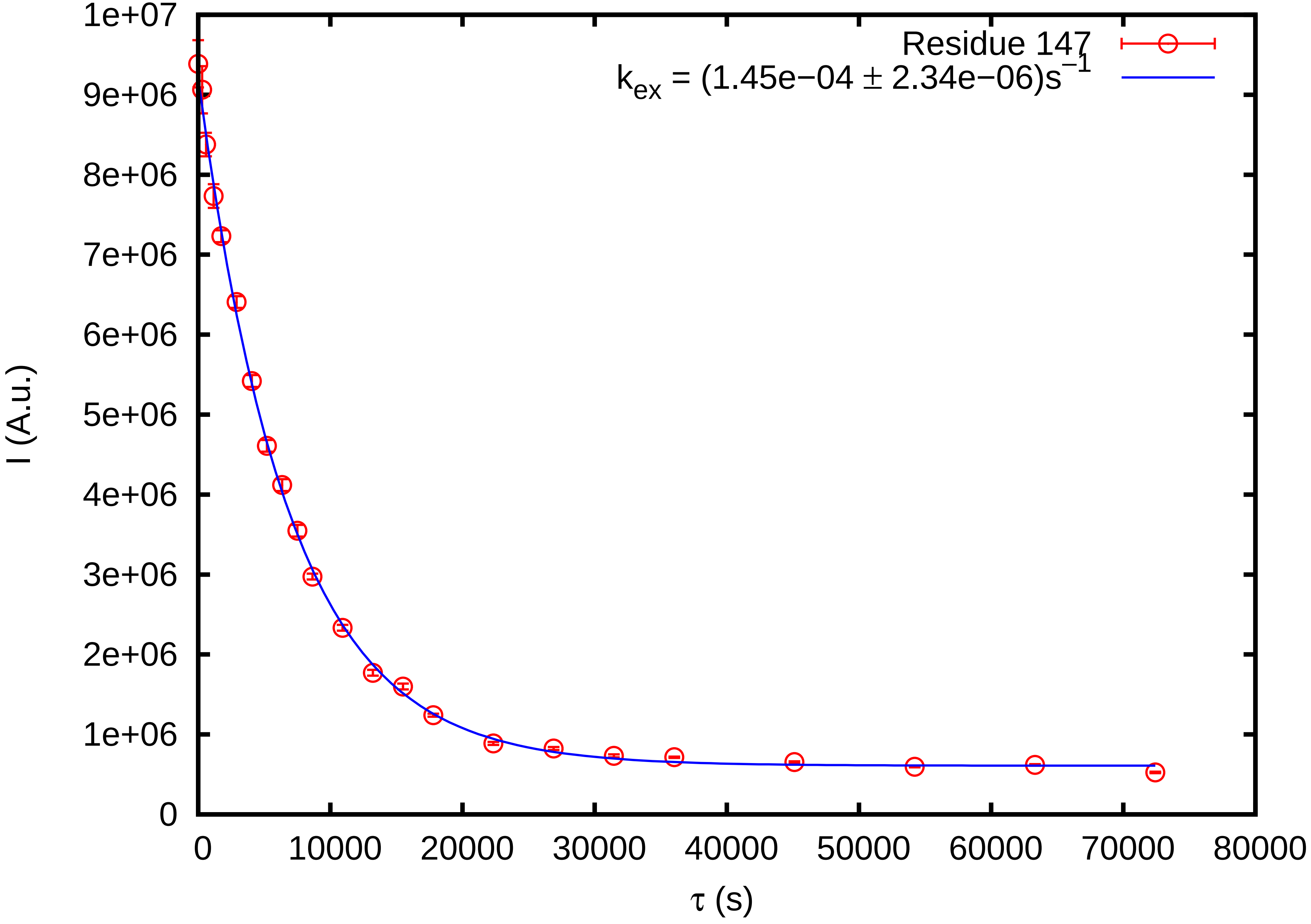

Residue 147
k_ex = (1.45e−04 ± 2.34e−06)s^−1
1e+07
9e+06
8e+06
7e+06
6e+06
5e+06
4e+06
3e+06
2e+06
1e+06
0
I (A.u.)
0
10000
20000
30000
40000
50000
60000
70000
80000
τ (s)

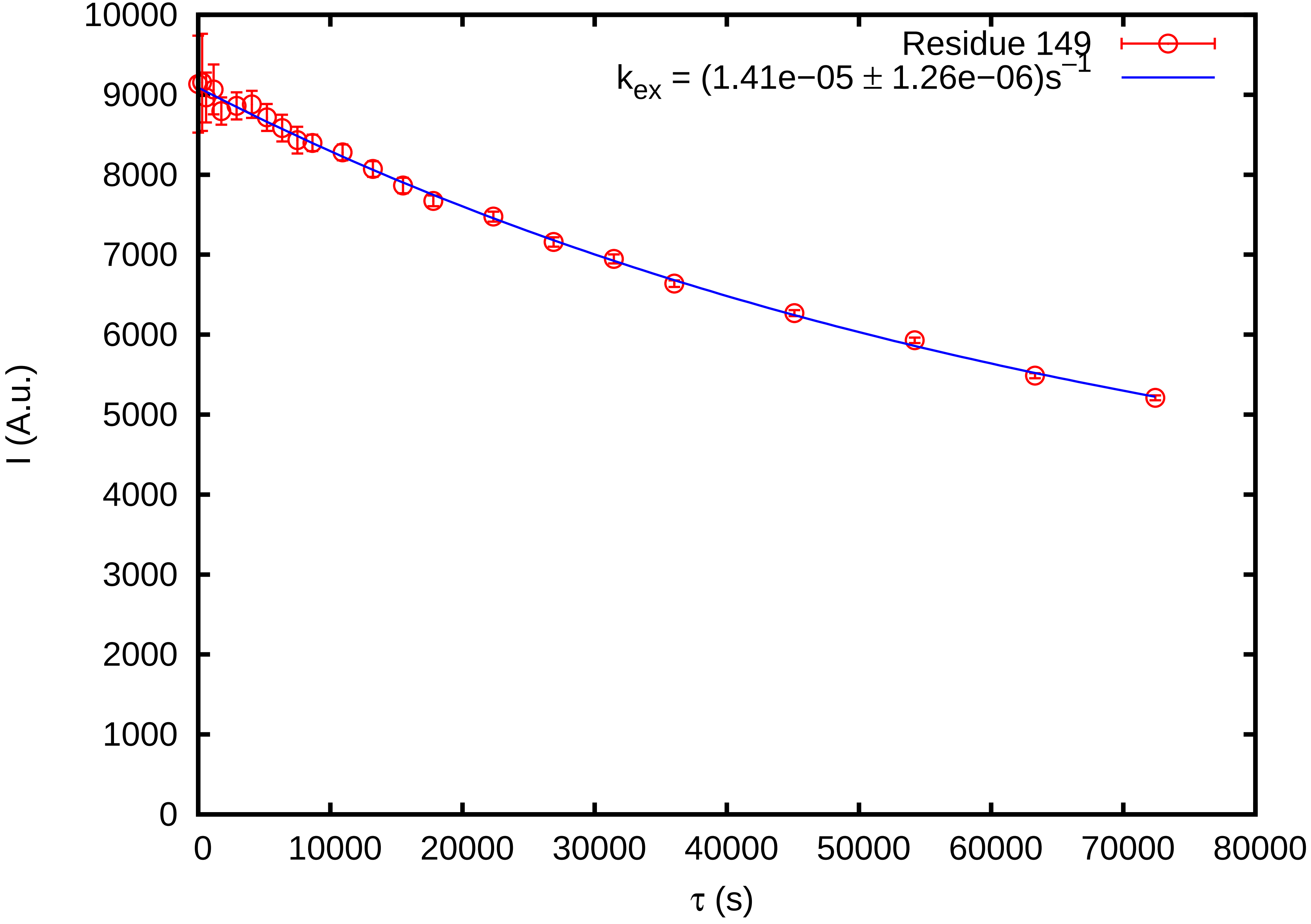

Residue 149
k_{ex} = (1.41e−05 ± 1.26e−06)s^{-1}
I (A.u.)
τ (s)
0
1000
2000
3000
4000
5000
6000
7000
8000
9000
10000
0
10000
20000
30000
40000
50000
60000
70000
80000

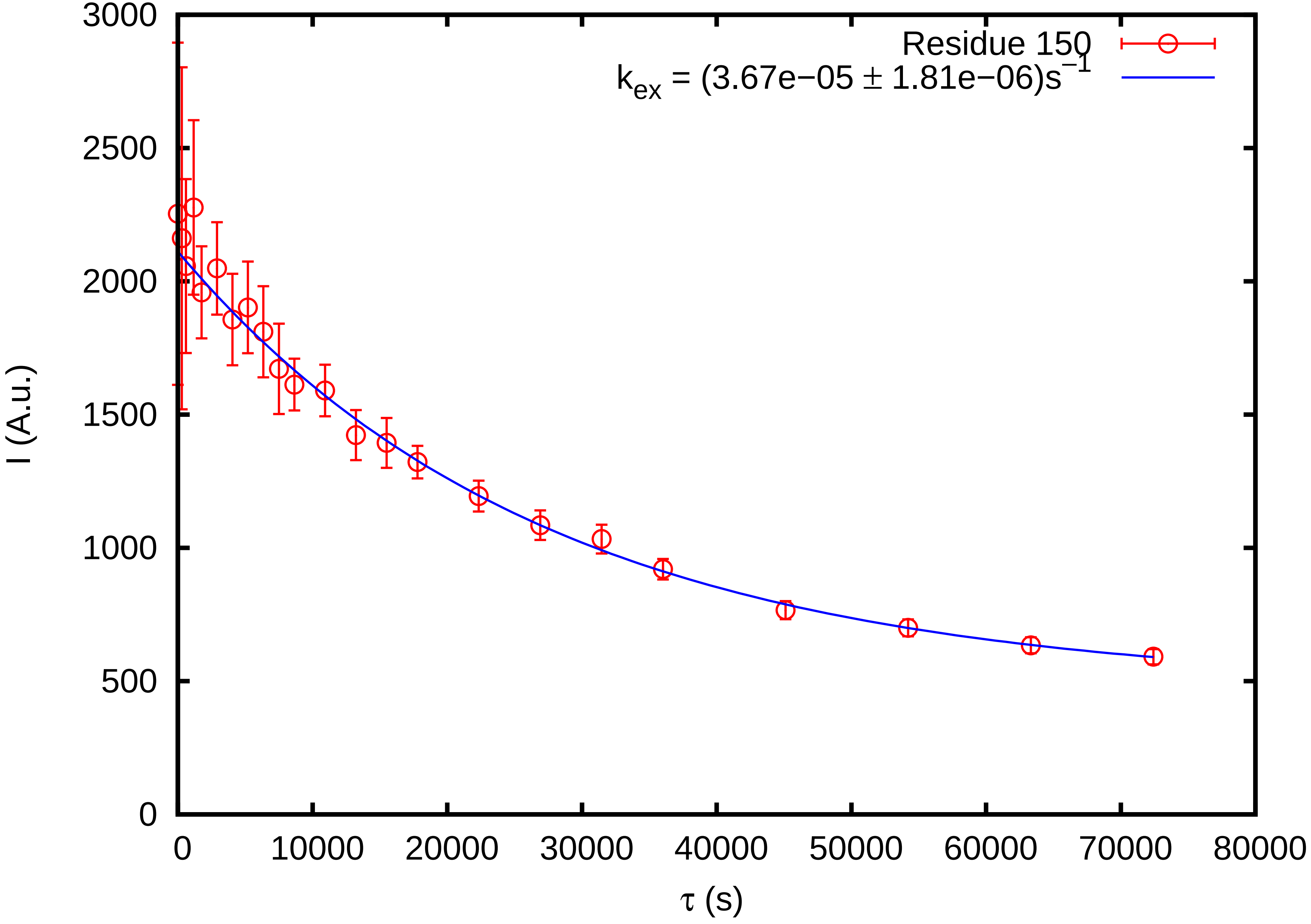

Residue 150
k_ex = (3.67e−05 ± 1.81e−06)s^−1
I (A.u.)
τ (s)
0
500
1000
1500
2000
2500
3000
0
10000
20000
30000
40000
50000
60000
70000
80000

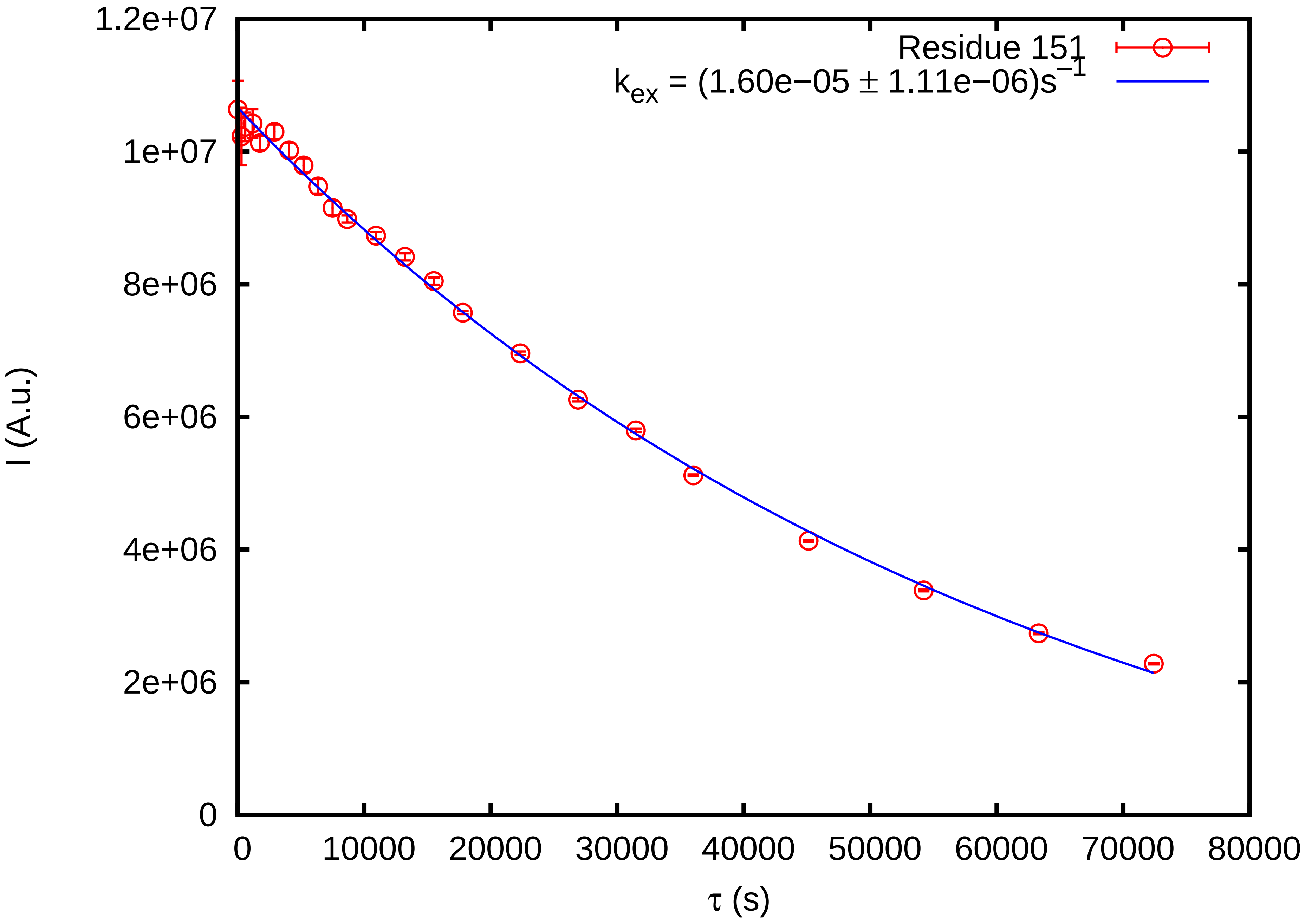

Residue 151
k_{ex} = (1.60e−05 ± 1.11e−06)s^{-1}
I (A.u.)
τ (s)
1.2e+07
1e+07
8e+06
6e+06
4e+06
2e+06
0
0
10000
20000
30000
40000
50000
60000
70000
80000

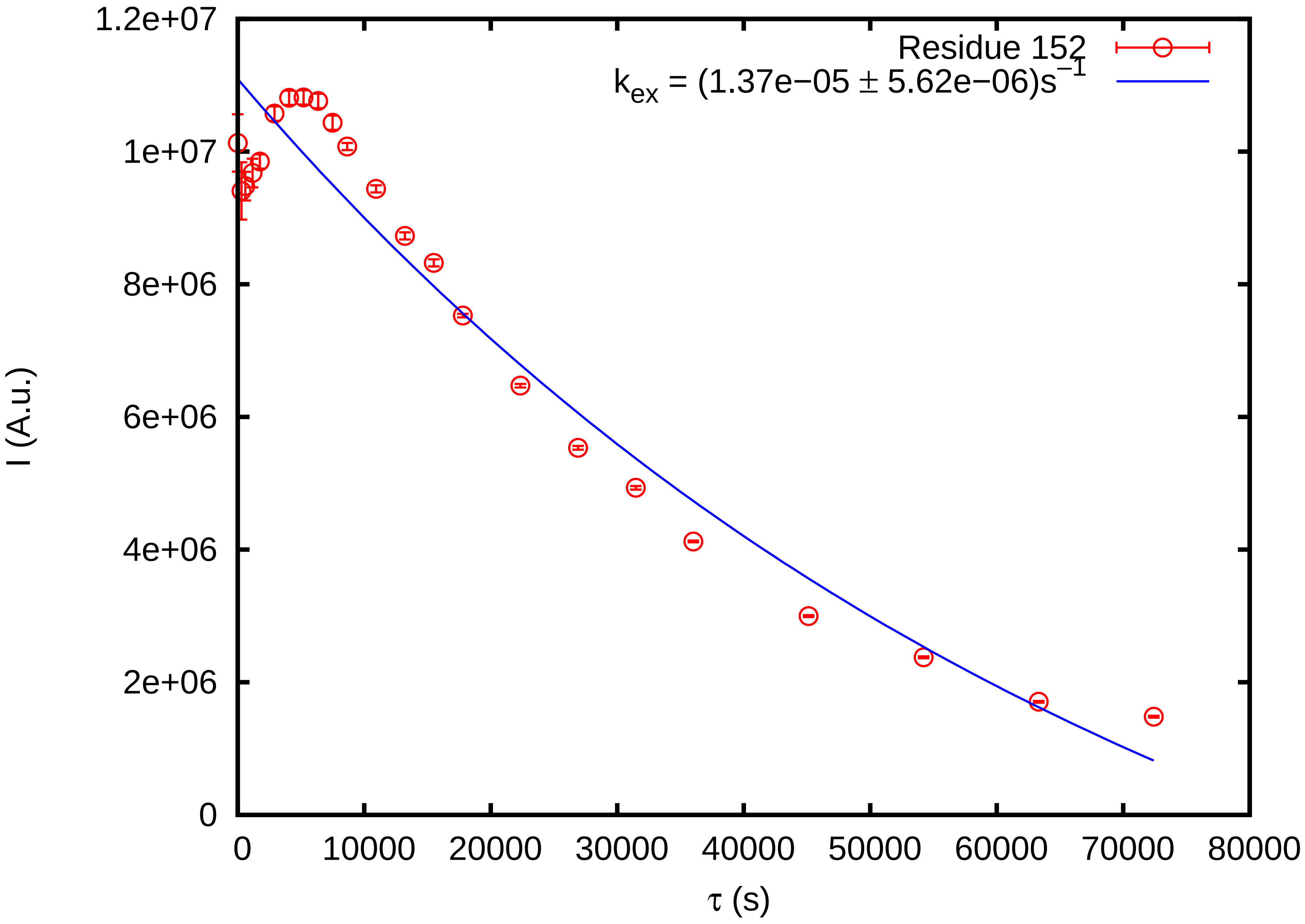

Residue 152
k_ex = (1.37e−05 ± 5.62e−06)s^−1
1.2e+07
1e+07
8e+06
6e+06
4e+06
2e+06
0
0
10000
20000
30000
40000
50000
60000
70000
80000
I (A.u.)
τ (s)

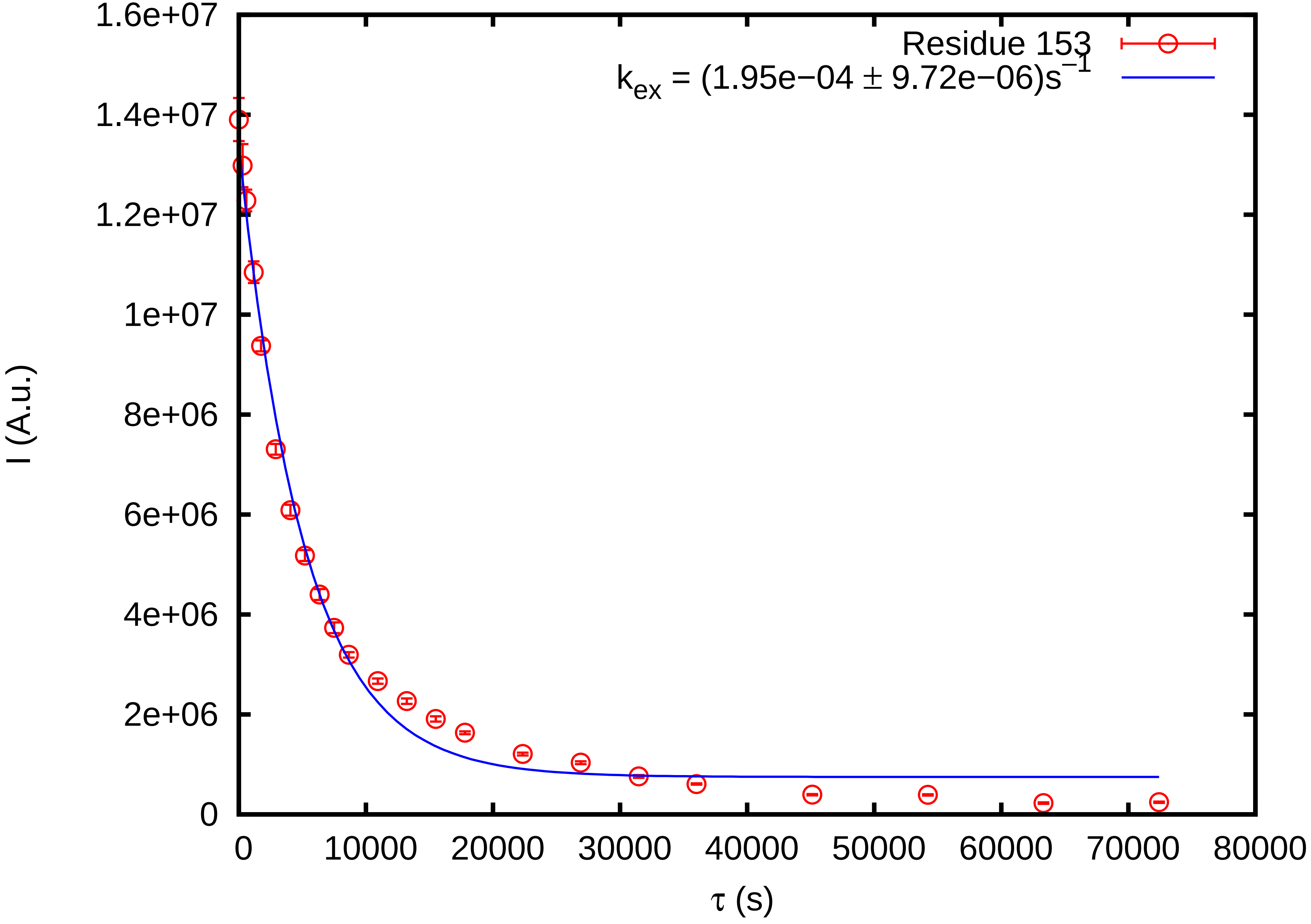
Residue 153
k_{ex} = (1.95e−04 ± 9.72e−06)s^{-1}
1.6e+07
1.4e+07
1.2e+07
1e+07
8e+06
6e+06
4e+06
2e+06
0
I (A.u.)
0
10000
20000
30000
40000
50000
60000
70000
80000
τ (s)

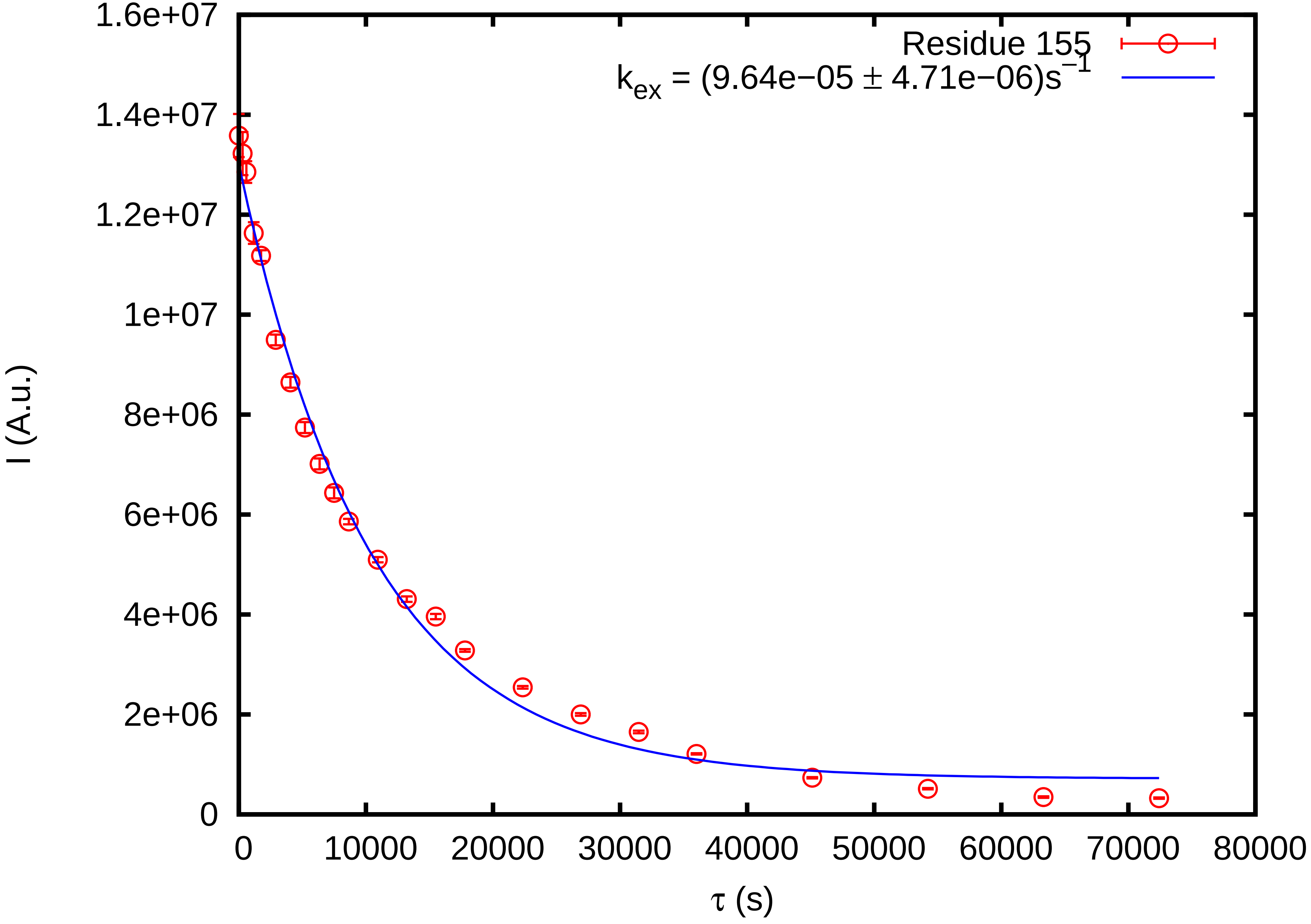

Residue 155
k_ex = (9.64e−05 ± 4.71e−06)s^−1
I (A.u.)
τ (s)
1.6e+07
1.4e+07
1.2e+07
1e+07
8e+06
6e+06
4e+06
2e+06
0
10000
20000
30000
40000
50000
60000
70000
80000

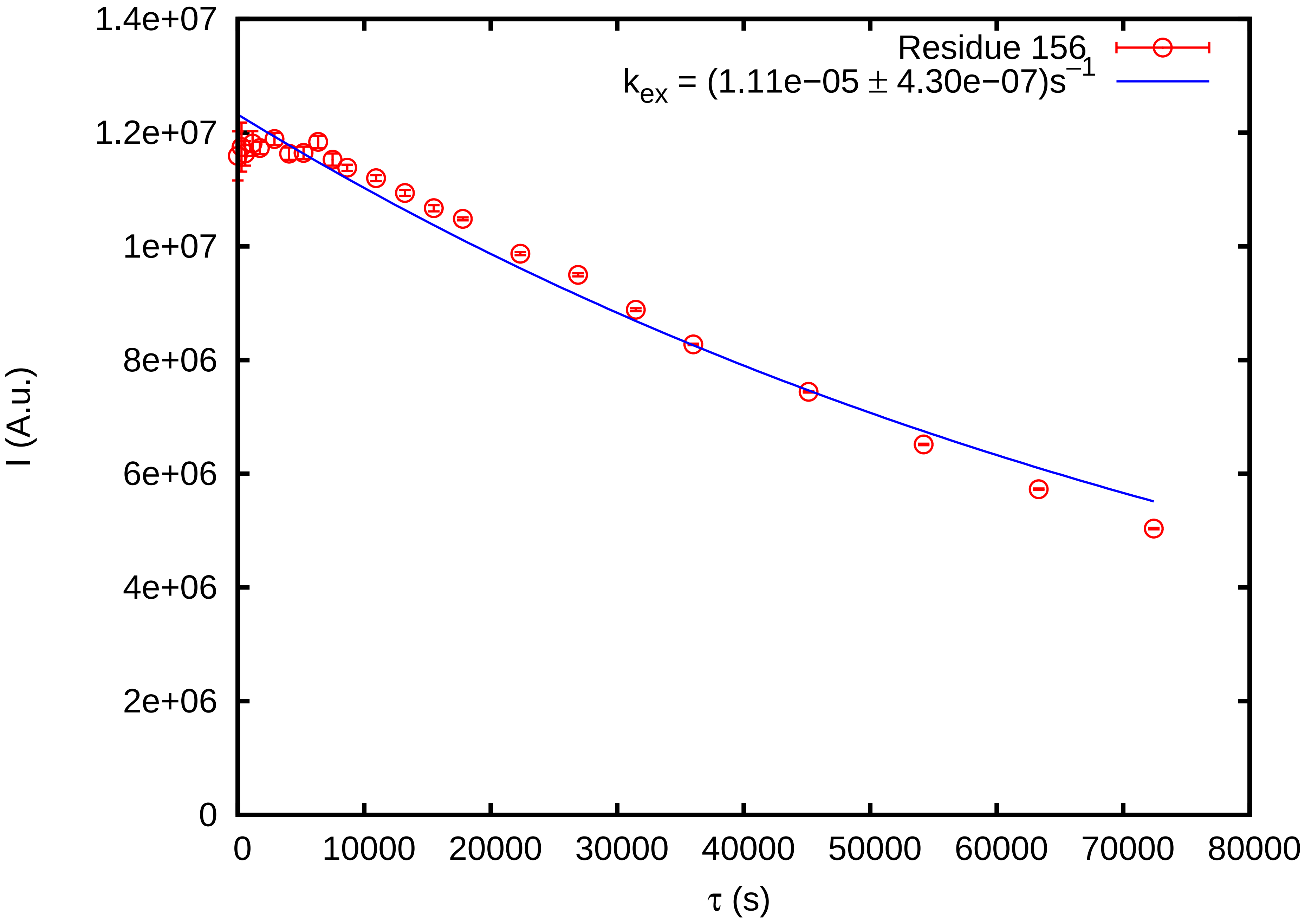

Residue 156
k_{ex} = (1.11e−05 ± 4.30e−07)s^{-1}
1.4e+07
1.2e+07
1e+07
8e+06
6e+06
4e+06
2e+06
0
I (A.u.)
0
10000
20000
30000
40000
50000
60000
70000
80000
τ (s)

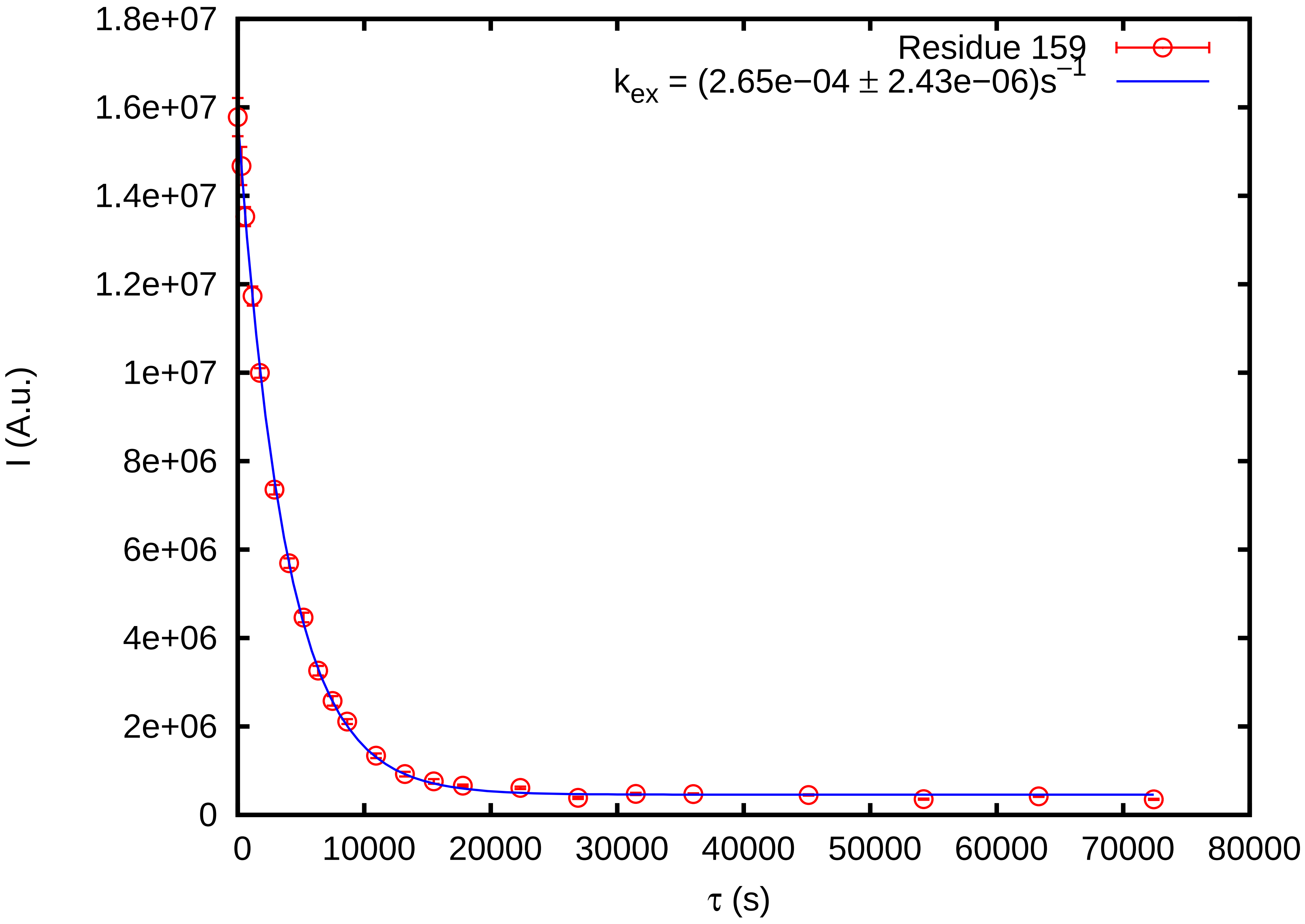

Residue 159
k_ex = (2.65e−04 ± 2.43e−06)s^−1
I (A.u.)
τ (s)
1.8e+07
1.6e+07
1.4e+07
1.2e+07
1e+07
8e+06
6e+06
4e+06
2e+06
0
0
10000
20000
30000
40000
50000
60000
70000
80000

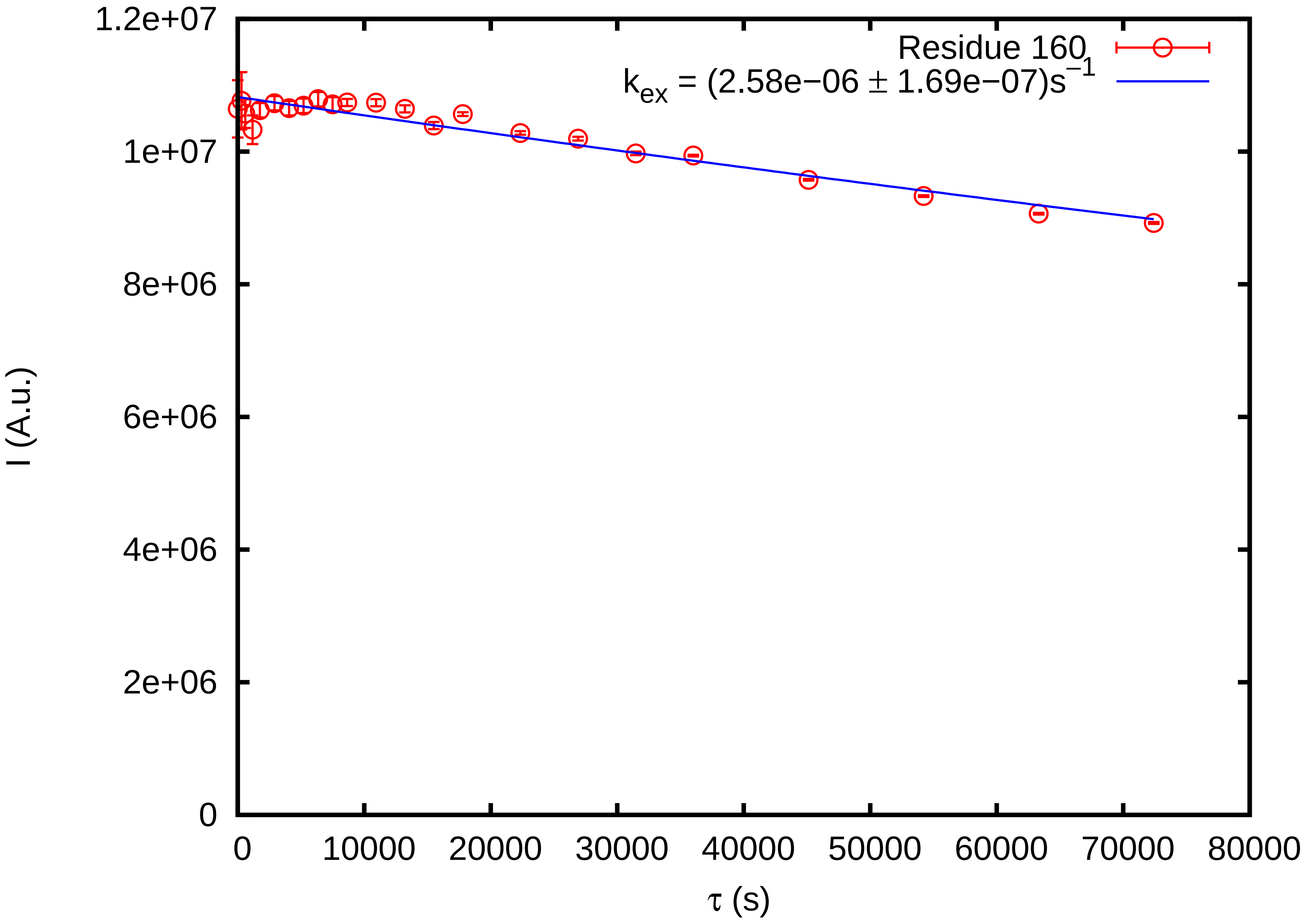

Residue 160
k_ex = (2.58e−06 ± 1.69e−07)s^−1
1.2e+07
1e+07
8e+06
6e+06
4e+06
2e+06
0
0
10000
20000
30000
40000
50000
60000
70000
80000
I (A.u.)
τ (s)

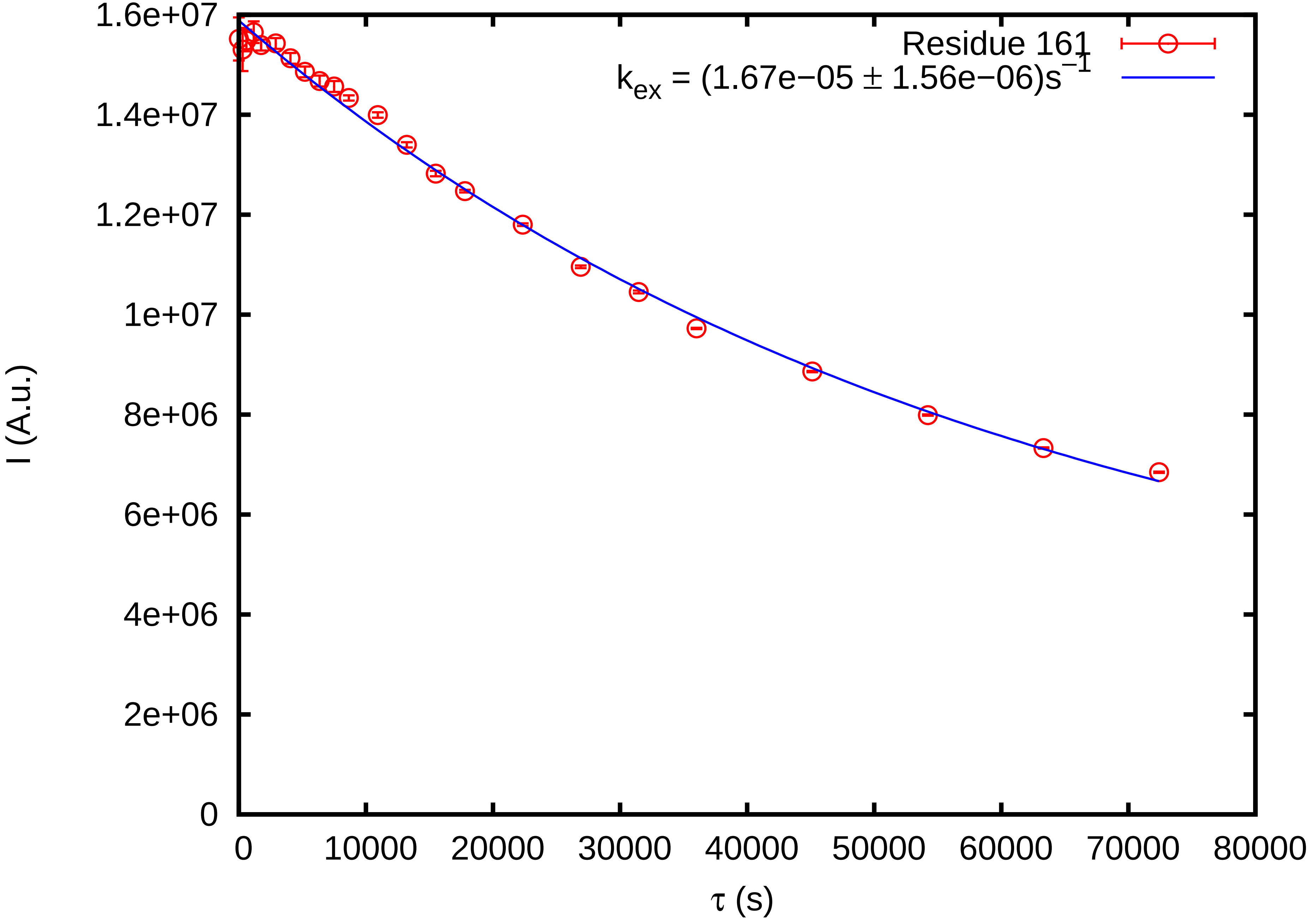

Residue 161
k_{ex} = (1.67e−05 ± 1.56e−06)s^{-1}
I (A.u.)
τ (s)
1.6e+07
1.4e+07
1.2e+07
1e+07
8e+06
6e+06
4e+06
2e+06
0
0
10000
20000
30000
40000
50000
60000
70000
80000

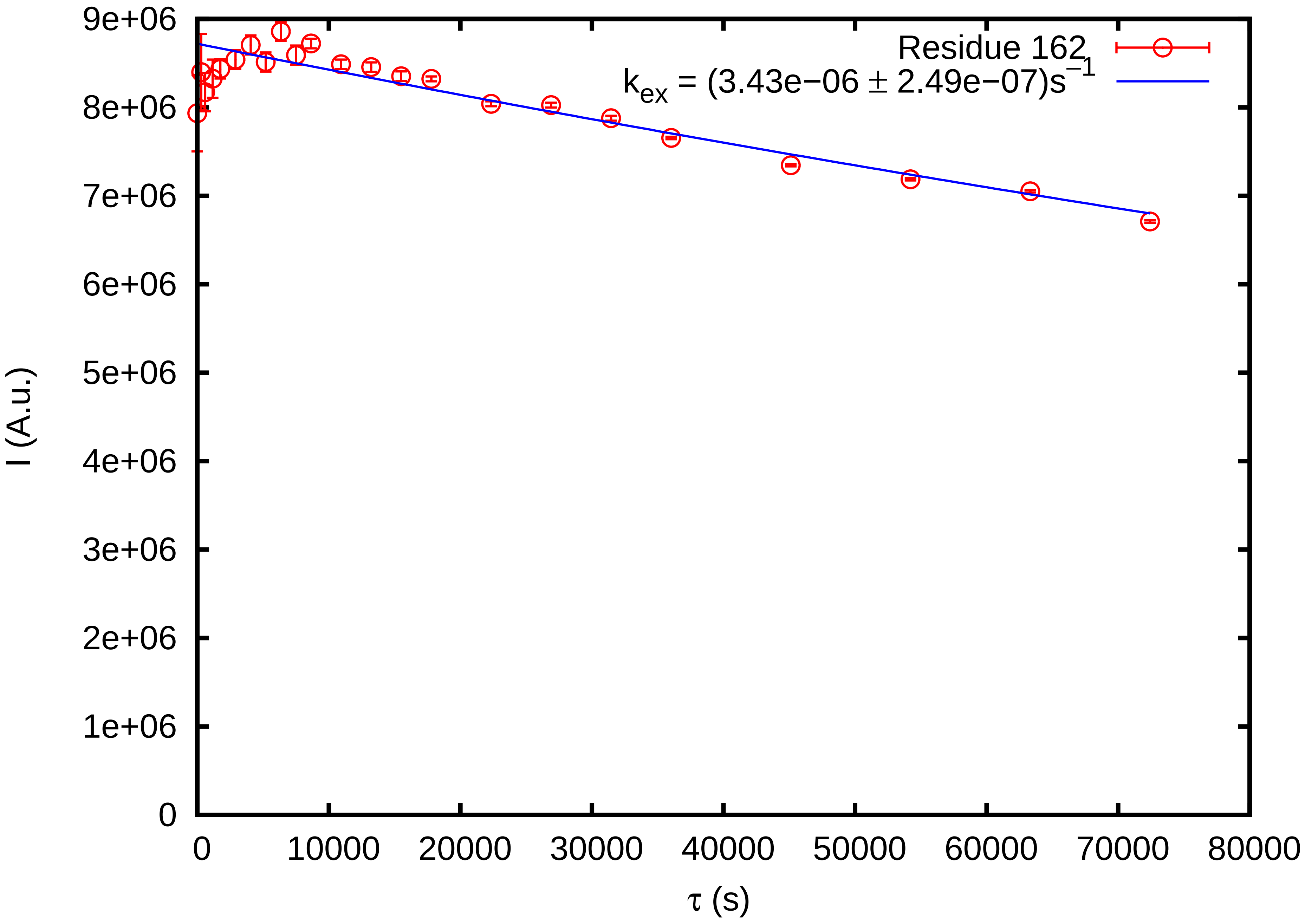

Residue 162
k_{ex} = (3.43e−06 ± 2.49e−07)s^{-1}
I (A.u.)
τ (s)
9e+06
8e+06
7e+06
6e+06
5e+06
4e+06
3e+06
2e+06
1e+06
0
0
10000
20000
30000
40000
50000
60000
70000
80000

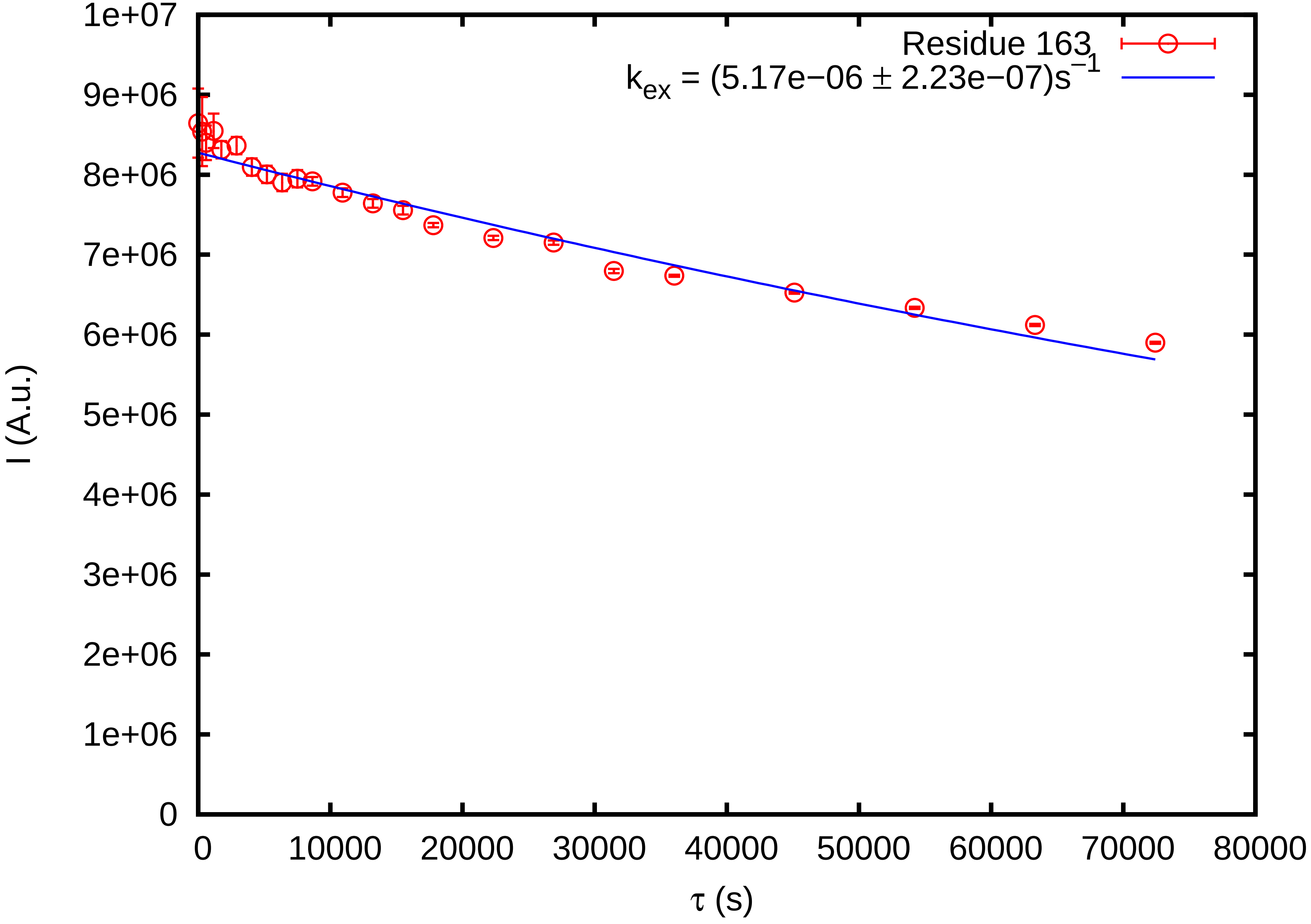

Residue 163
k_{ex} = (5.17e−06 ± 2.23e−07)s^{-1}
1e+07
9e+06
8e+06
7e+06
6e+06
5e+06
4e+06
3e+06
2e+06
1e+06
0
I (A.u.)
0
10000
20000
30000
40000
50000
60000
70000
80000
τ (s)

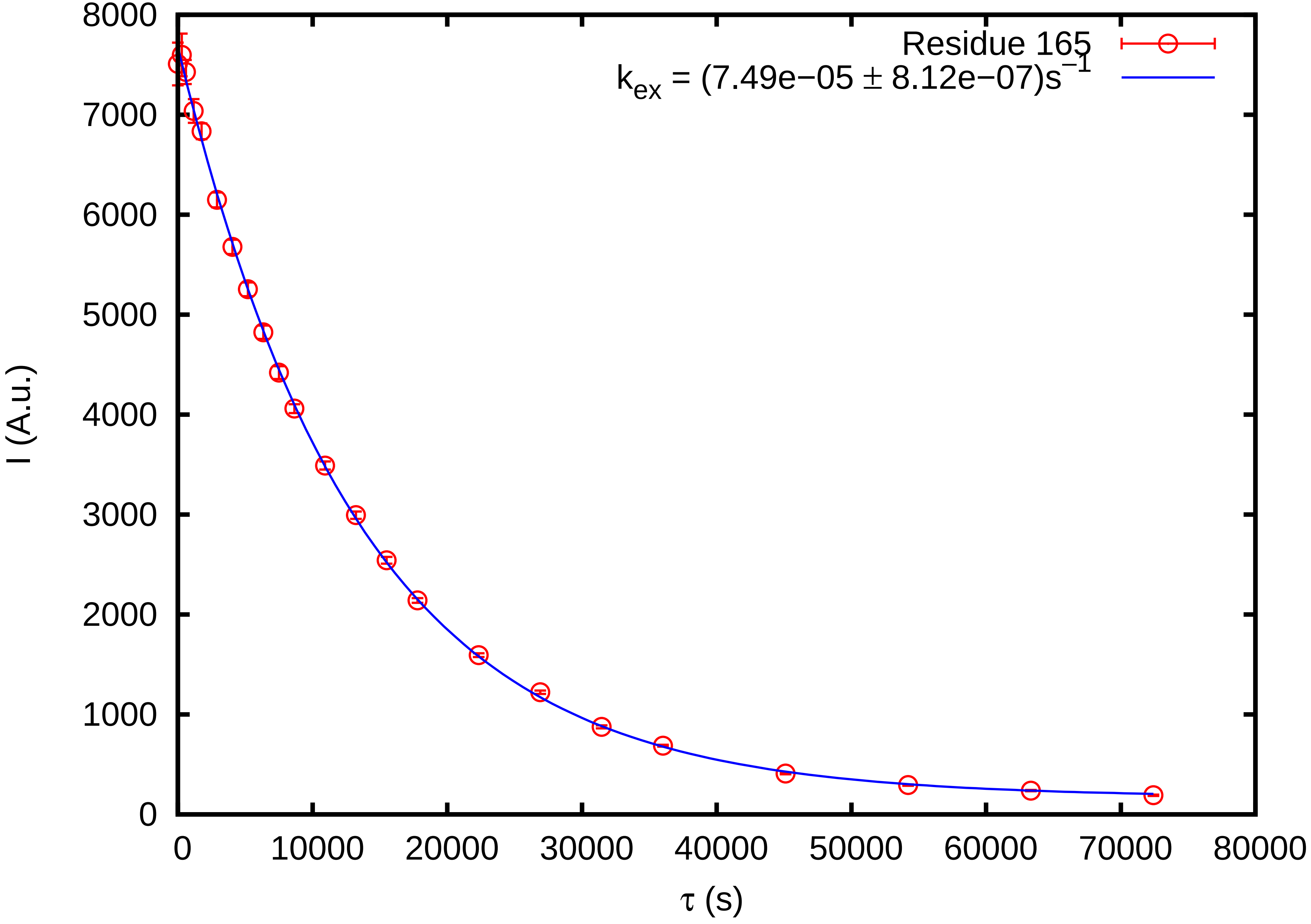

Residue 165
k_{ex} = (7.49e−05 ± 8.12e−07)s^{-1}
I (A.u.)
τ (s)
0
1000
2000
3000
4000
5000
6000
7000
8000
0
10000
20000
30000
40000
50000
60000
70000
80000

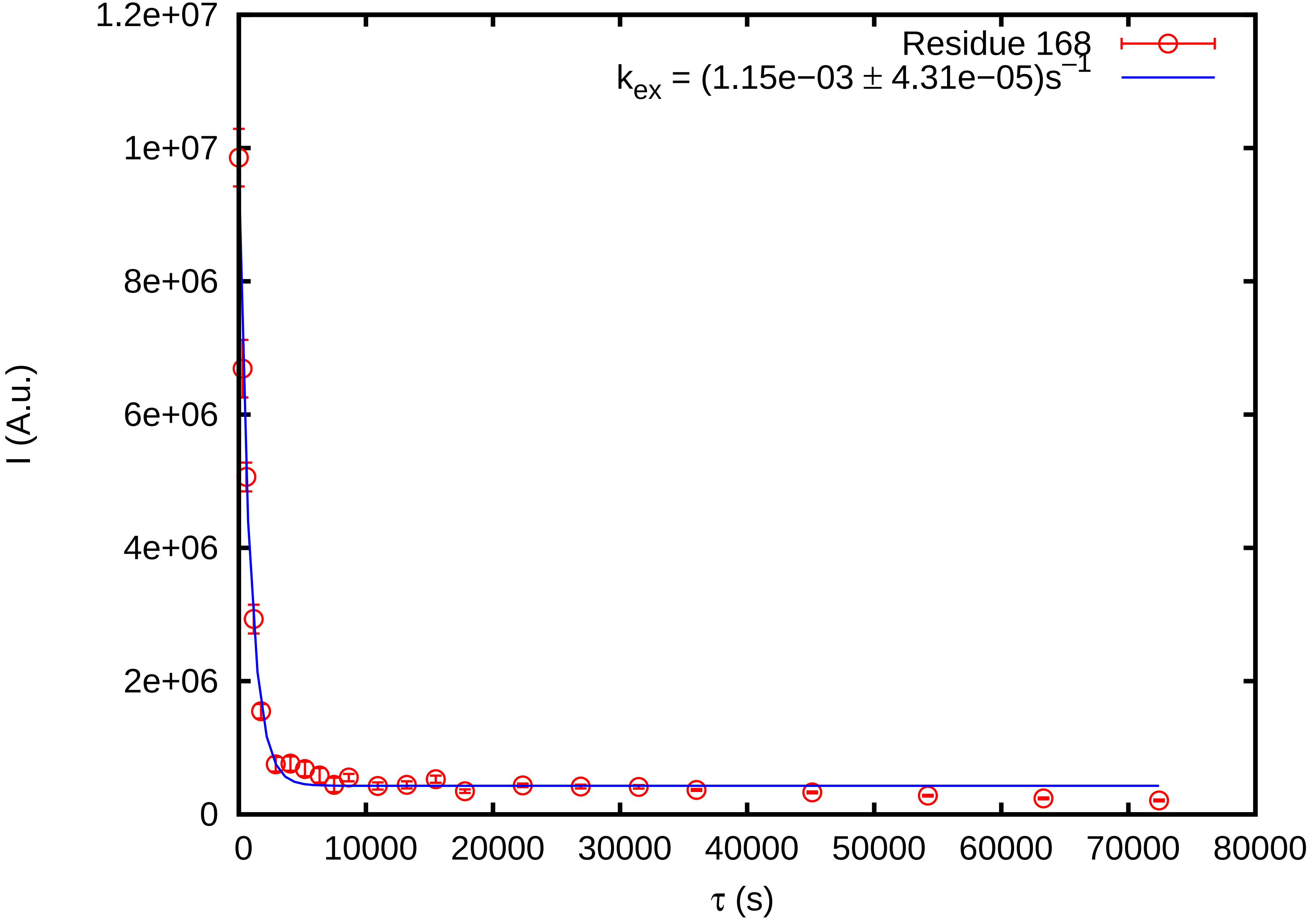

Residue 168
k_{ex} = (1.15e−03 ± 4.31e−05)s^{-1}
1.2e+07
1e+07
8e+06
6e+06
4e+06
2e+06
0
I (A.u.)
0
10000
20000
30000
40000
50000
60000
70000
80000
τ (s)

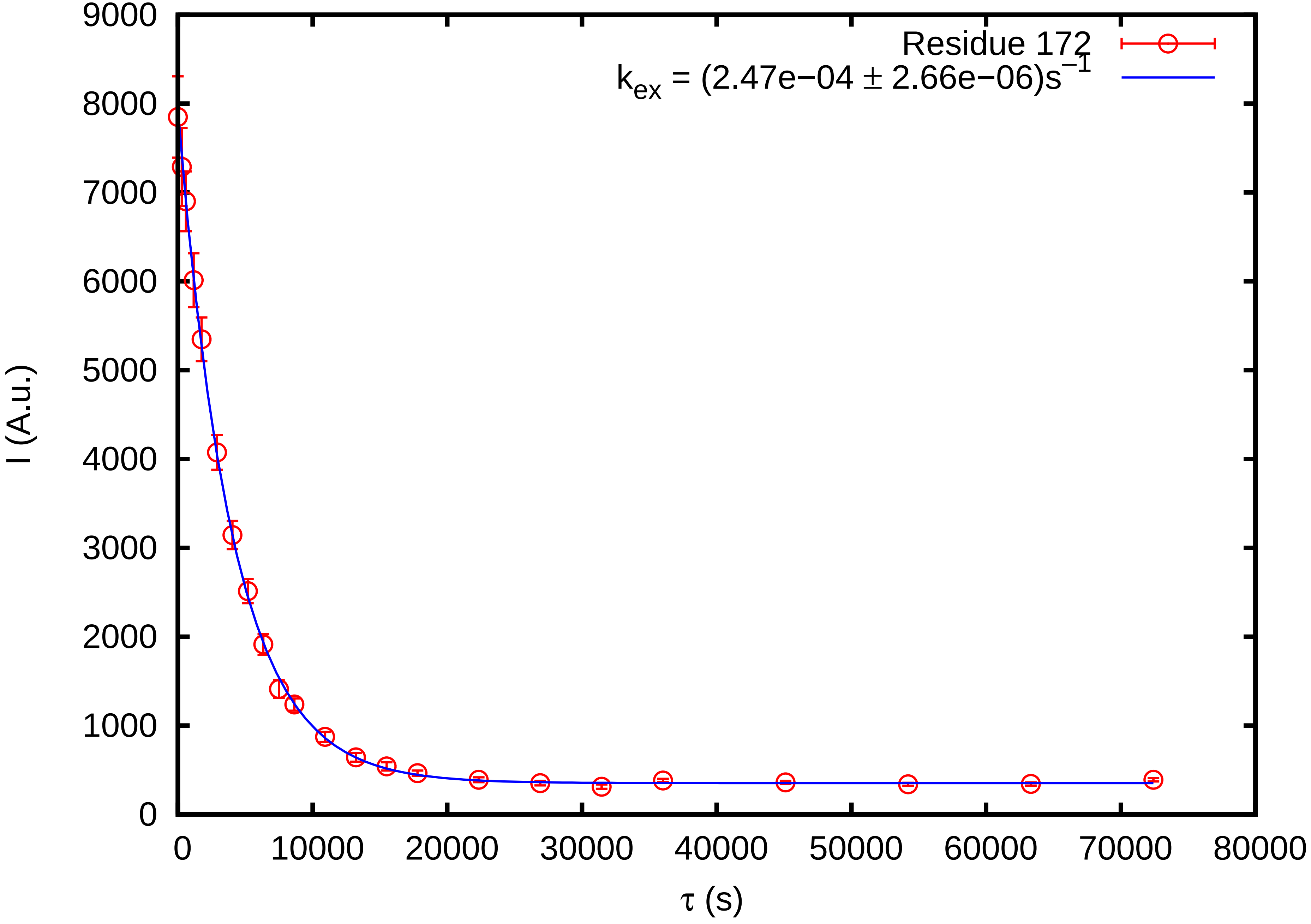

Residue 172
k_{ex} = (2.47e−04 ± 2.66e−06)s^{-1}
I (A.u.)
τ (s)
0
1000
2000
3000
4000
5000
6000
7000
8000
9000
0
10000
20000
30000
40000
50000
60000
70000
80000

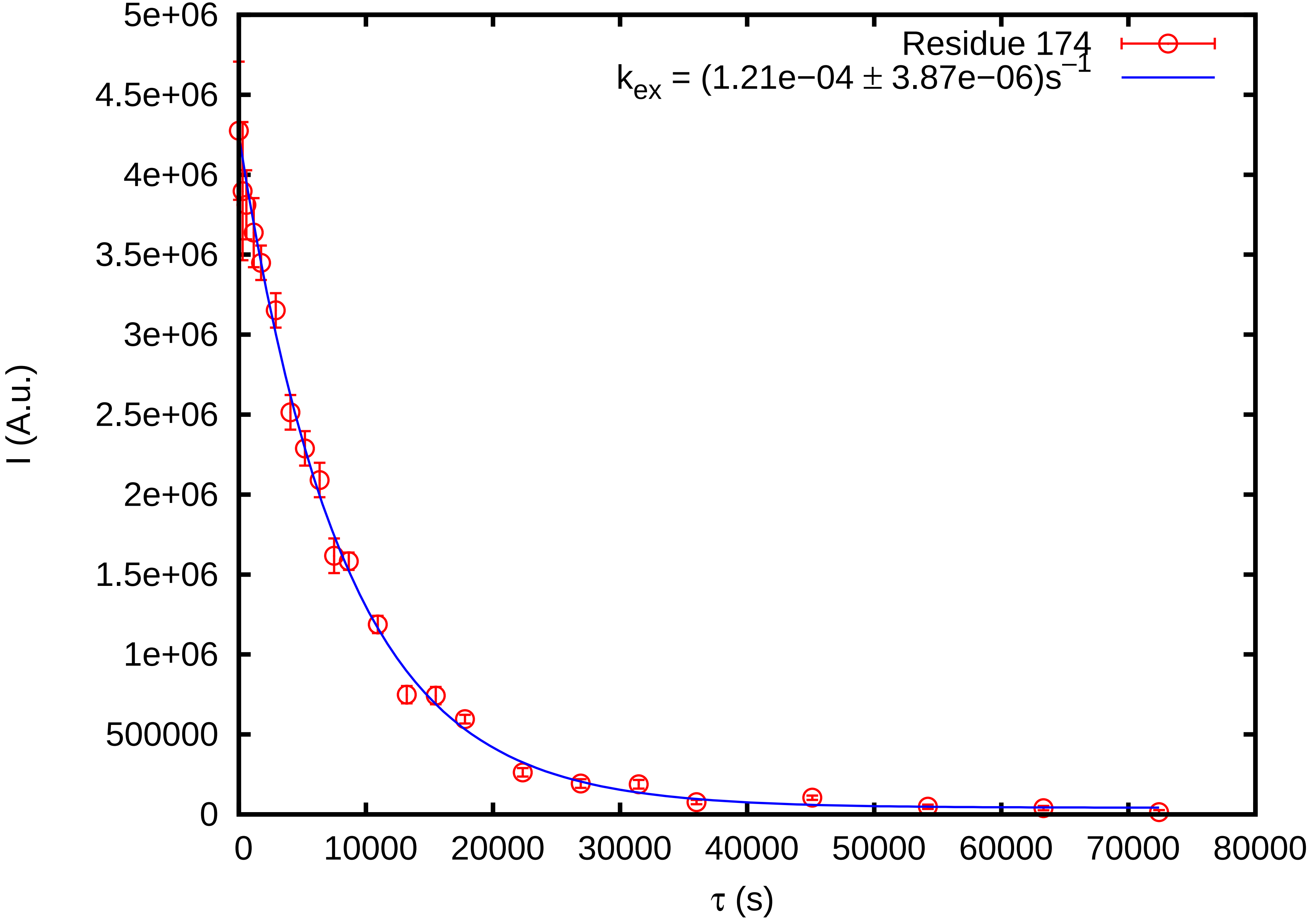

Residue 174
k_ex = (1.21e−04 ± 3.87e−06)s^−1
I (A.u.)
τ (s)
5e+06
4.5e+06
4e+06
3.5e+06
3e+06
2.5e+06
2e+06
1.5e+06
1e+06
500000
0
0
10000
20000
30000
40000
50000
60000
70000
80000

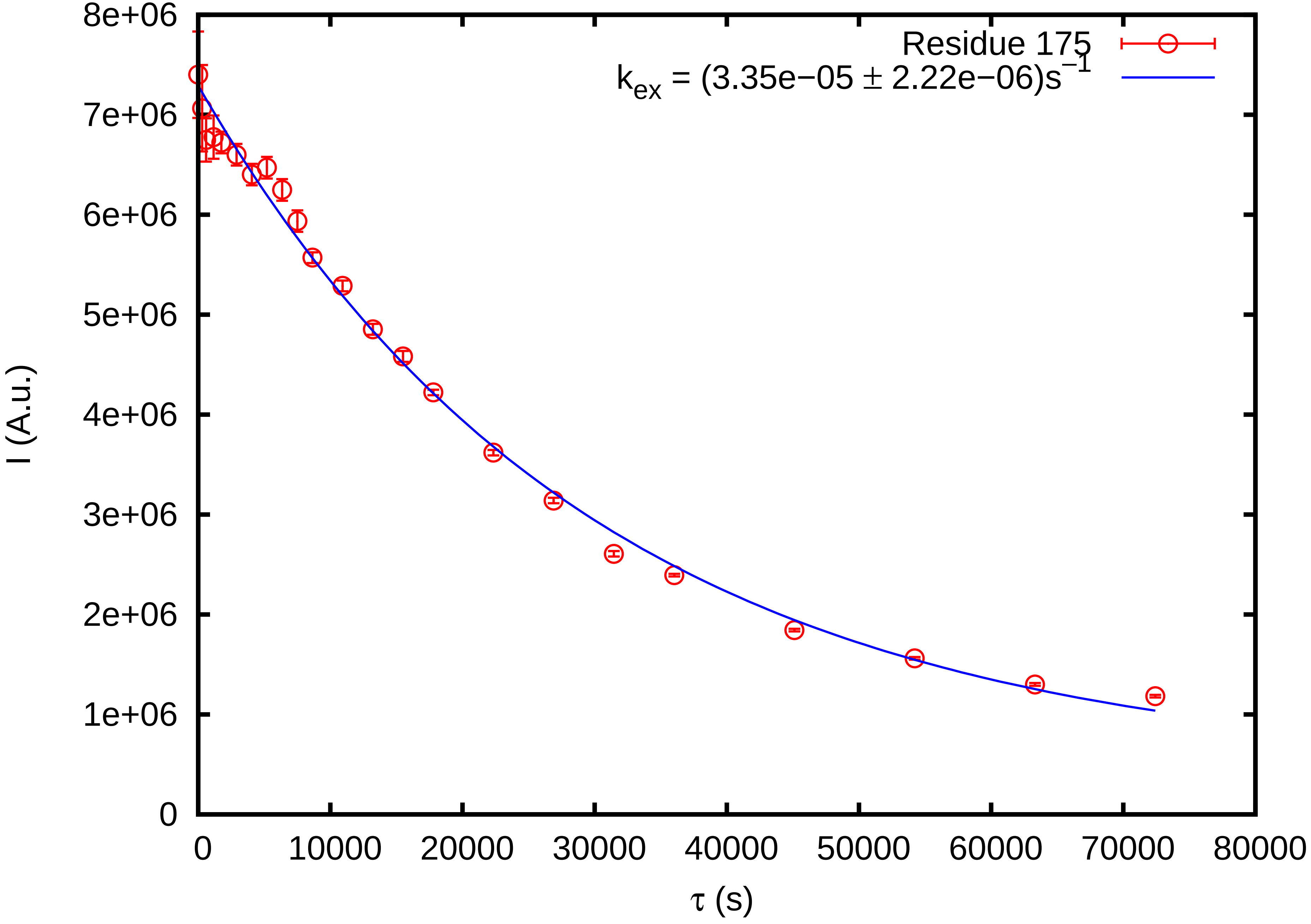

Residue 175
k_ex = (3.35e−05 ± 2.22e−06)s^−1
I (A.u.)
τ (s)
0
1e+06
2e+06
3e+06
4e+06
5e+06
6e+06
7e+06
8e+06
0
10000
20000
30000
40000
50000
60000
70000
80000

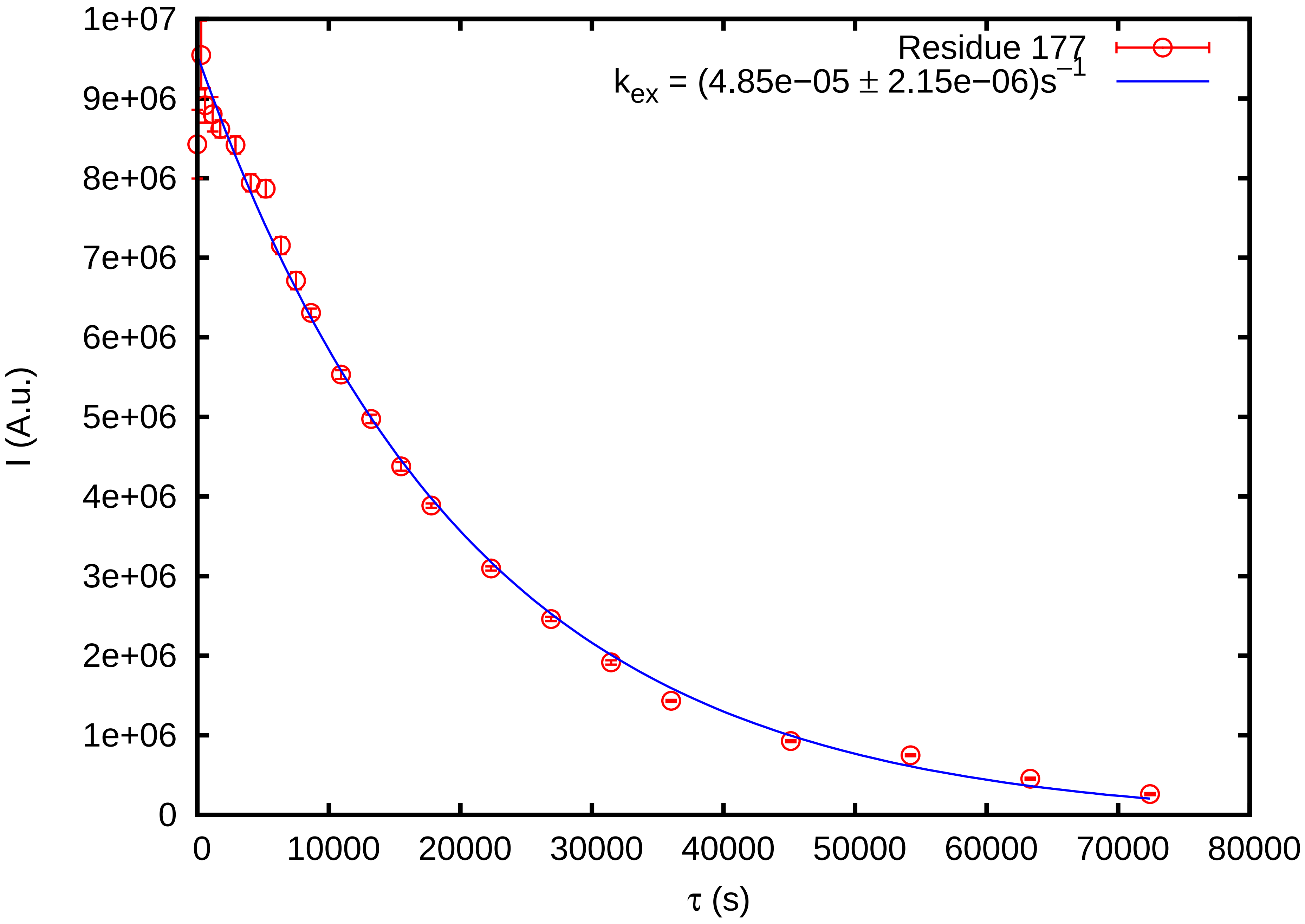

Residue 177
k_ex = (4.85e−05 ± 2.15e−06)s^−1
I (A.u.)
τ (s)
0
1e+06
2e+06
3e+06
4e+06
5e+06
6e+06
7e+06
8e+06
9e+06
1e+07
0
10000
20000
30000
40000
50000
60000
70000
80000

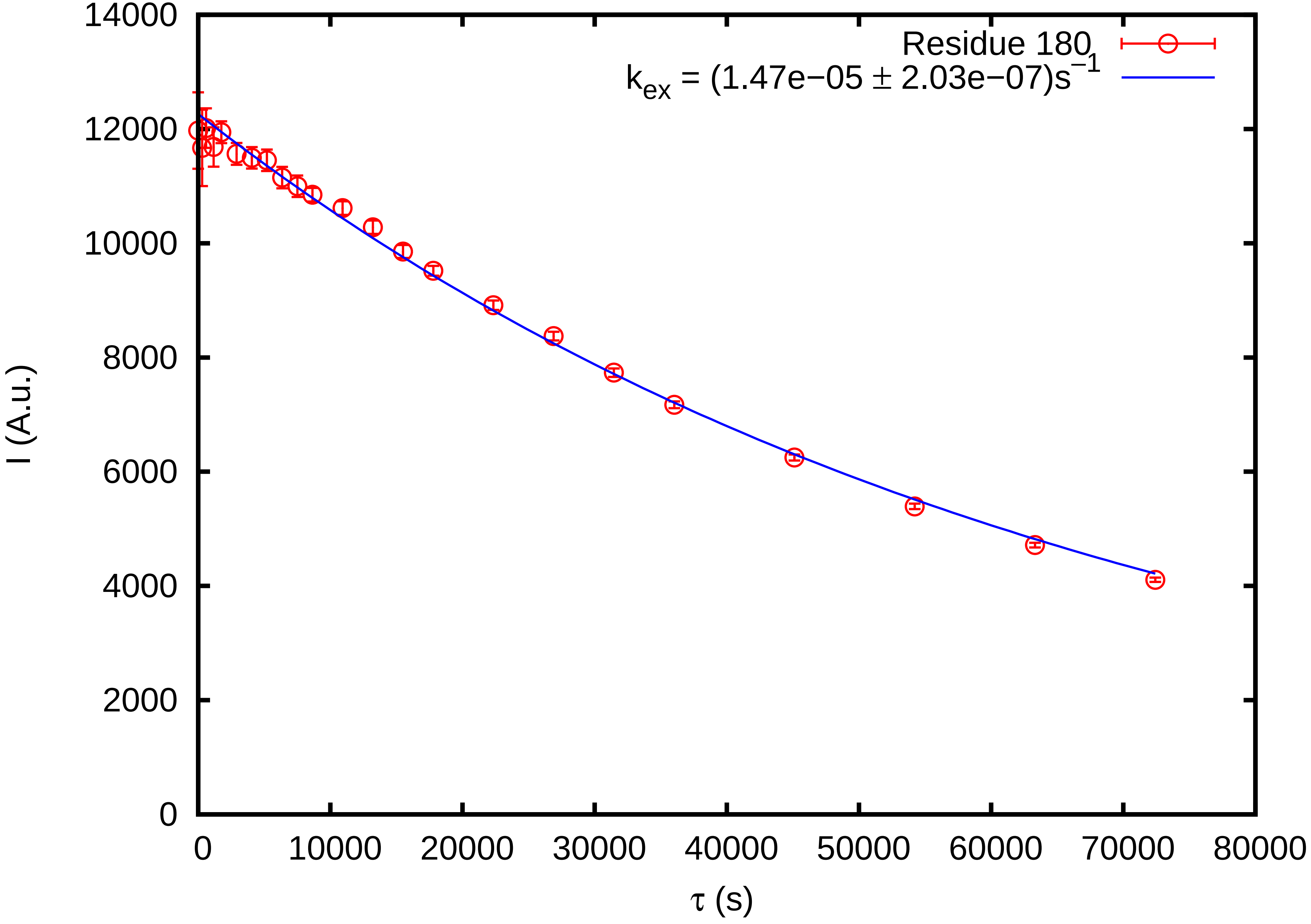

Residue 180
kex = (1.47e−05 ± 2.03e−07)s−1
14000
12000
10000
8000
6000
4000
2000
0
0
10000
20000
30000
40000
50000
60000
70000
80000
I (A.u.)
τ (s)

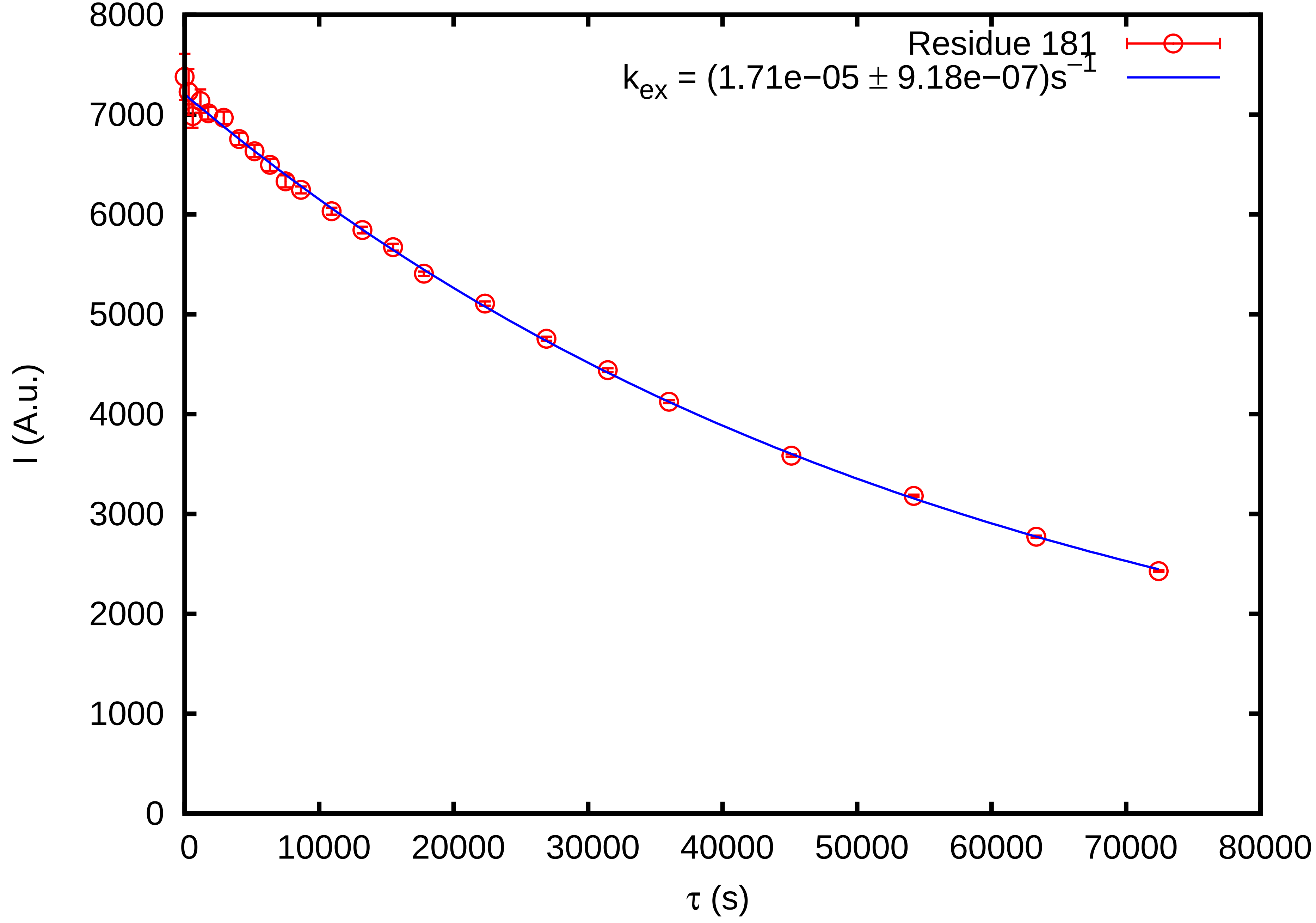

Residue 181
k_{ex} = (1.71e−05 ± 9.18e−07)s^{-1}
I (A.u.)
τ (s)
0
1000
2000
3000
4000
5000
6000
7000
8000
0
10000
20000
30000
40000
50000
60000
70000
80000

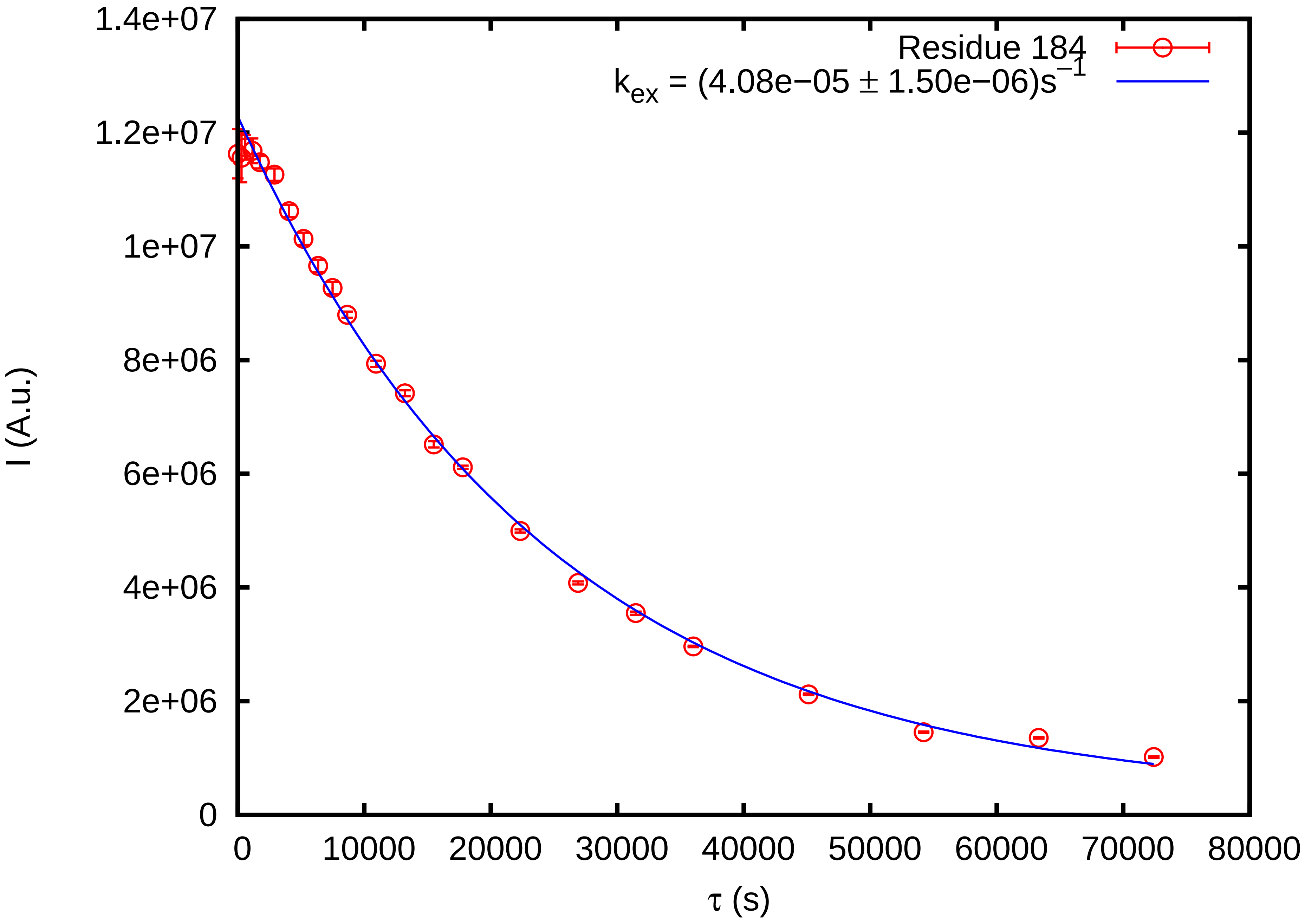

Residue 184
k_ex = (4.08e−05 ± 1.50e−06)s^−1
I (A.u.)
τ (s)
1.4e+07
1.2e+07
1e+07
8e+06
6e+06
4e+06
2e+06
0
0
10000
20000
30000
40000
50000
60000
70000
80000

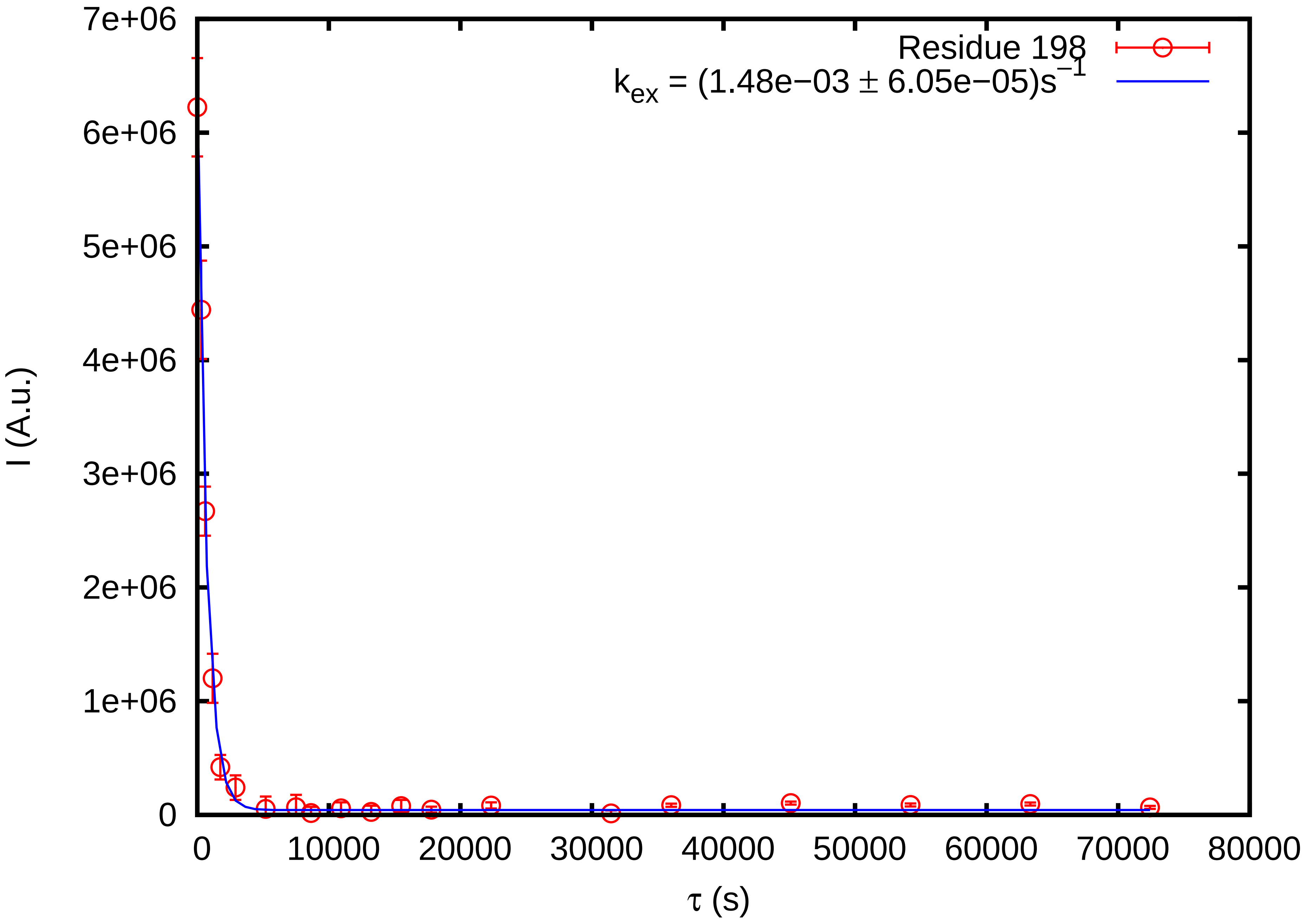

Residue 198
k_{ex} = (1.48e−03 ± 6.05e−05)s^{-1}
I (A.u.)
τ (s)
0
1e+06
2e+06
3e+06
4e+06
5e+06
6e+06
7e+06
0
10000
20000
30000
40000
50000
60000
70000
80000

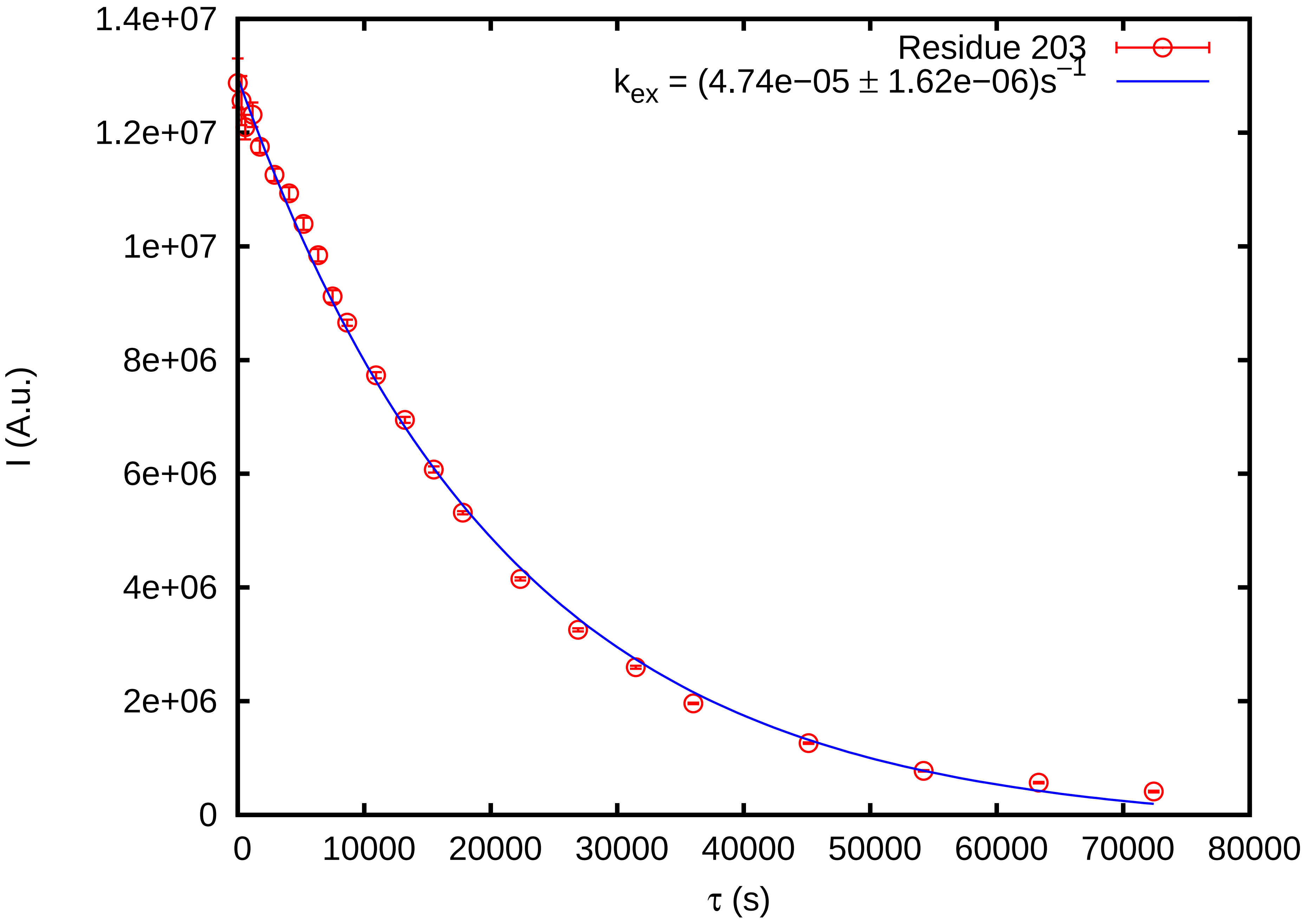

Residue 203
k_{ex} = (4.74e−05 ± 1.62e−06)s^{-1}
1.4e+07
1.2e+07
1e+07
8e+06
6e+06
4e+06
2e+06
0
I (A.u.)
0
10000
20000
30000
40000
50000
60000
70000
80000
τ (s)

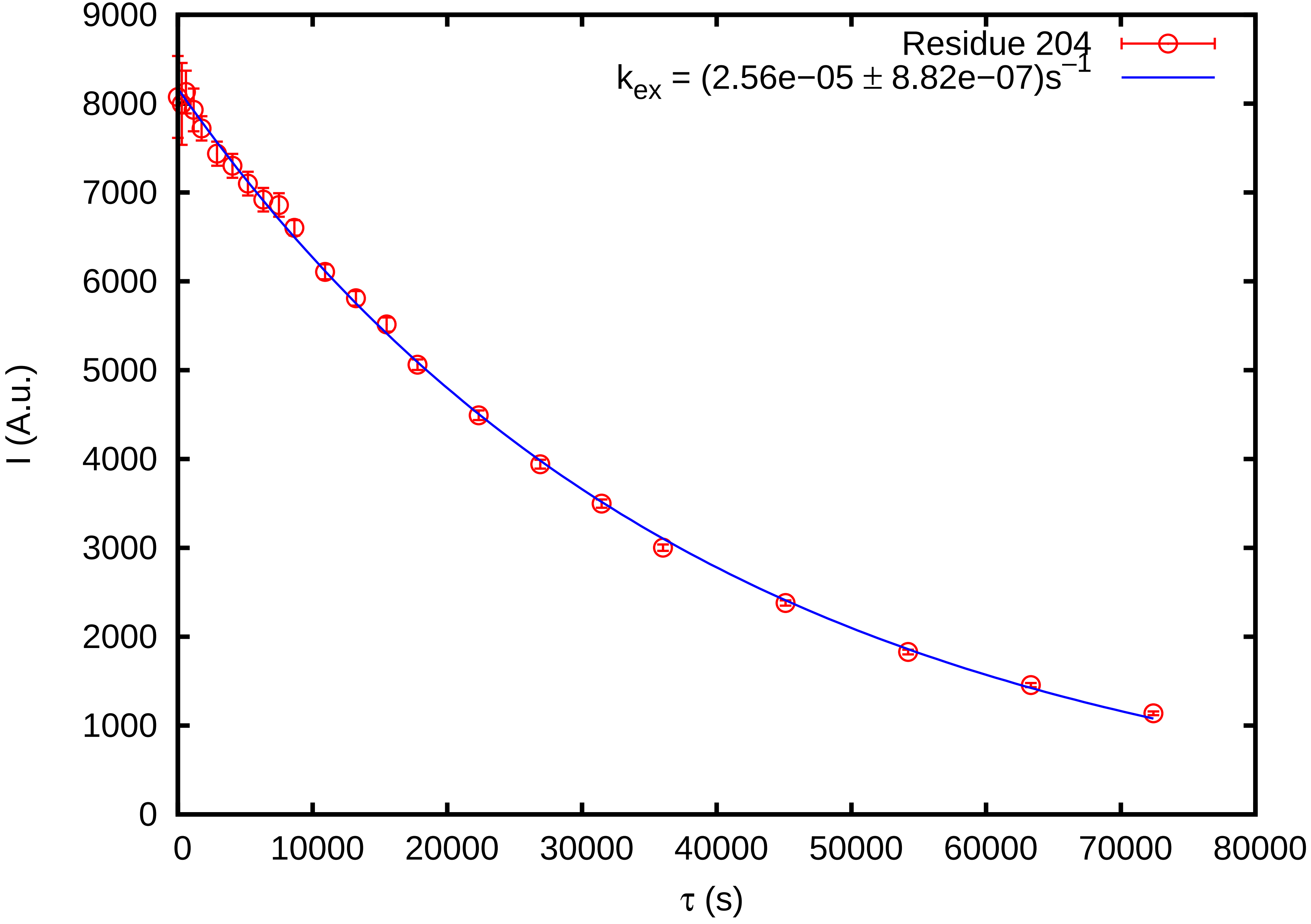

Residue 204
k_ex = (2.56e−05 ± 8.82e−07)s^−1
I (A.u.)
τ (s)
0
1000
2000
3000
4000
5000
6000
7000
8000
9000
0
10000
20000
30000
40000
50000
60000
70000
80000

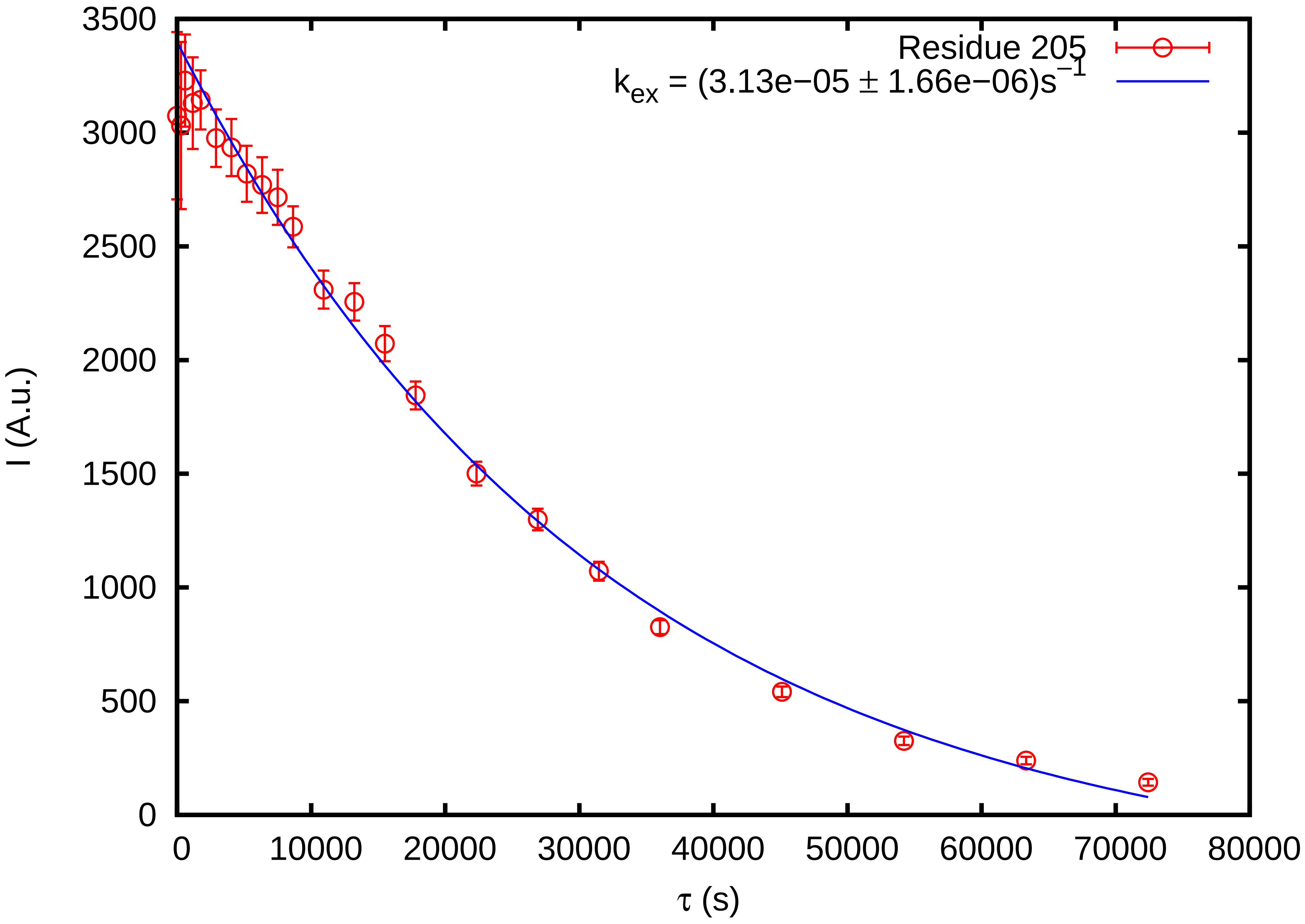

Residue 205
k_{ex} = (3.13e−05 ± 1.66e−06)s^{-1}
I (A.u.)
τ (s)
3500
3000
2500
2000
1500
1000
500
0
0
10000
20000
30000
40000
50000
60000
70000
80000

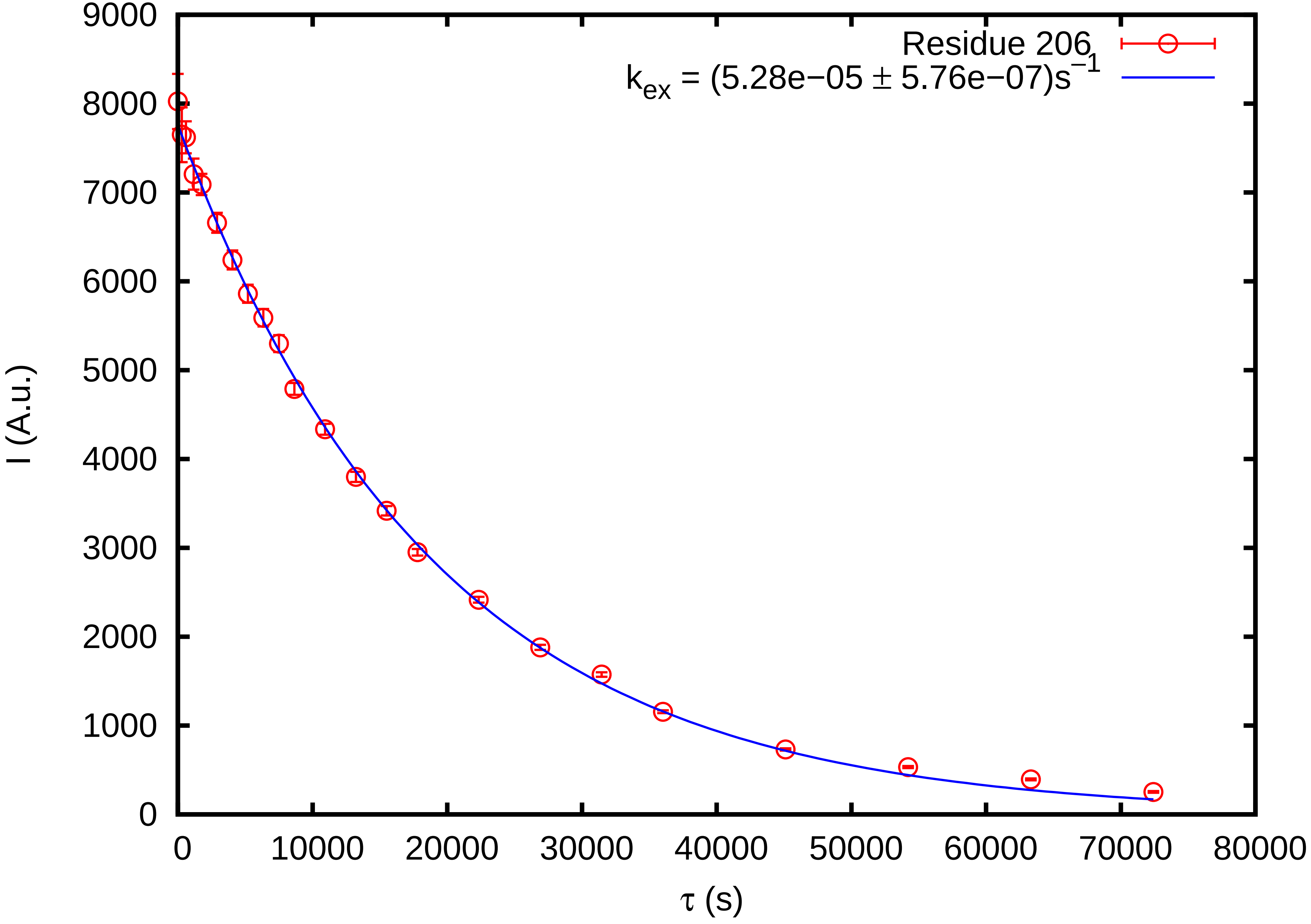

Residue 206
k_{ex} = (5.28e−05 ± 5.76e−07)s^{-1}
I (A.u.)
τ (s)
9000
8000
7000
6000
5000
4000
3000
2000
1000
0
0
10000
20000
30000
40000
50000
60000
70000
80000

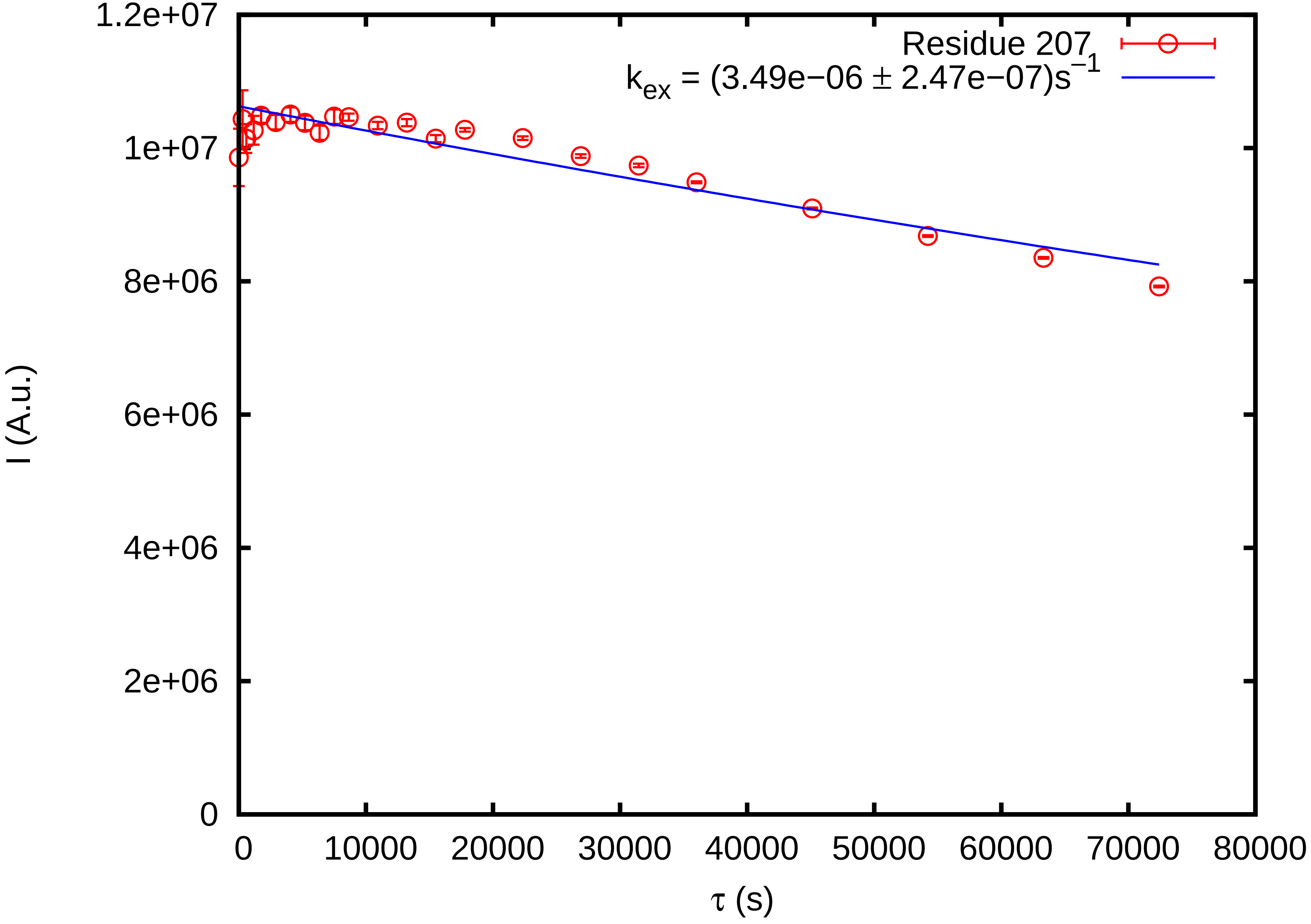

Residue 207
k_{ex} = (3.49e−06 ± 2.47e−07)s^{-1}
I (A.u.)
τ (s)
1.2e+07
1e+07
8e+06
6e+06
4e+06
2e+06
0
0
10000
20000
30000
40000
50000
60000
70000
80000

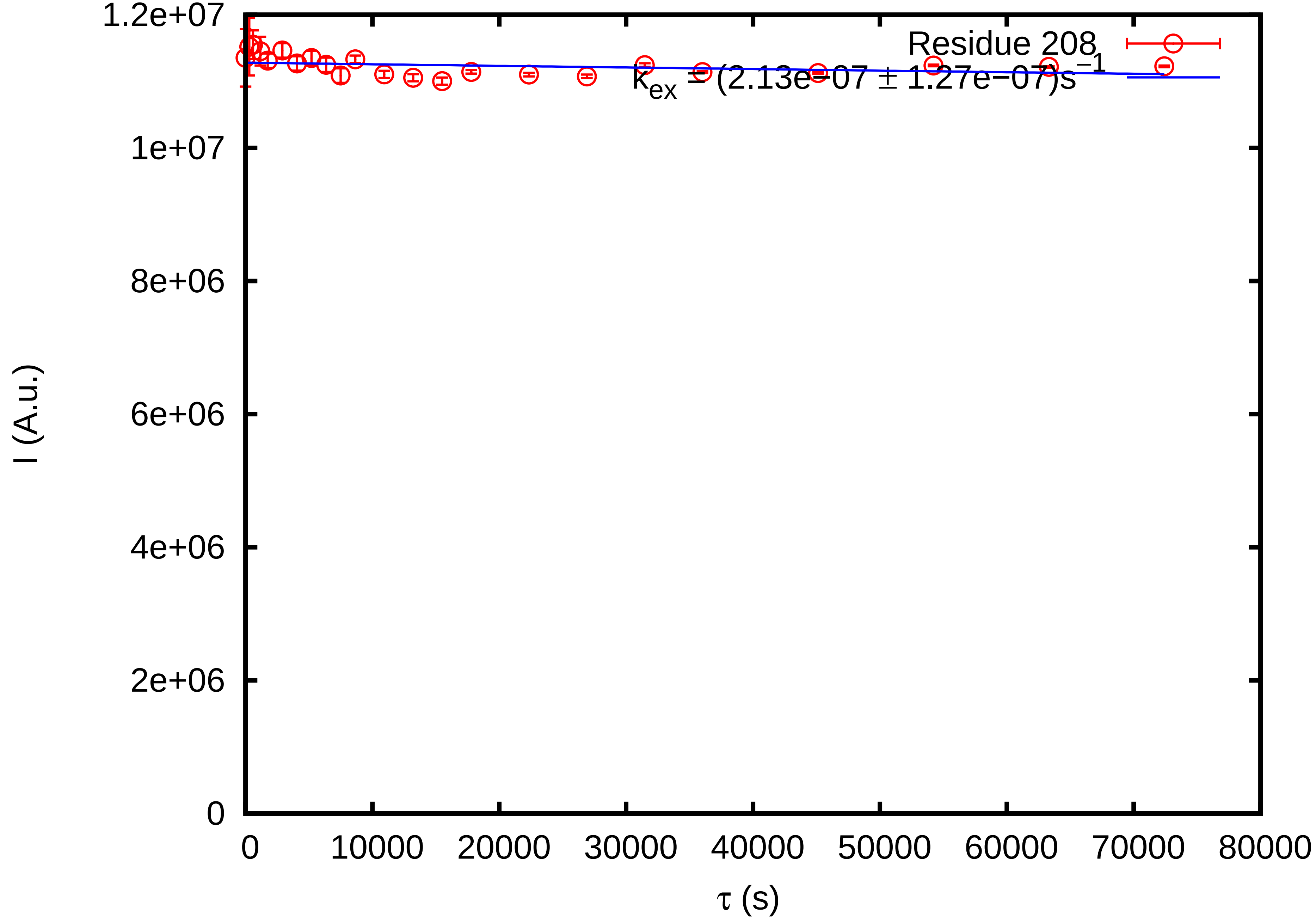

Residue 208
k_{ex} = (2.13e−07 ± 1.27e−07)s^{-1}
1.2e+07
1e+07
8e+06
6e+06
4e+06
2e+06
0
0
10000
20000
30000
40000
50000
60000
70000
80000
I (A.u.)
τ (s)

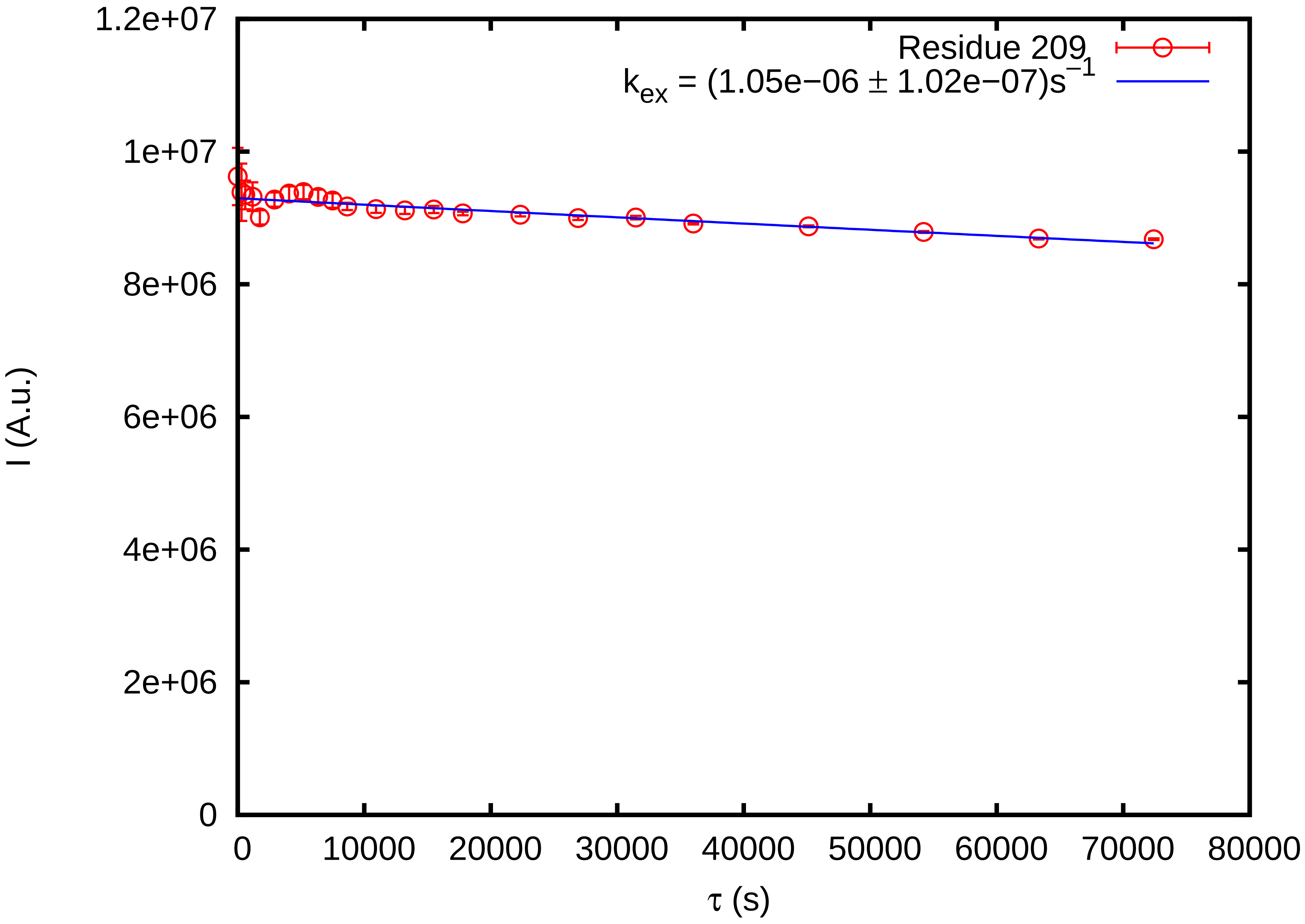

Residue 209
k_{ex} = (1.05e−06 ± 1.02e−07)s^{-1}
1.2e+07
1e+07
8e+06
6e+06
4e+06
2e+06
0
I (A.u.)
0
10000
20000
30000
40000
50000
60000
70000
80000
τ (s)

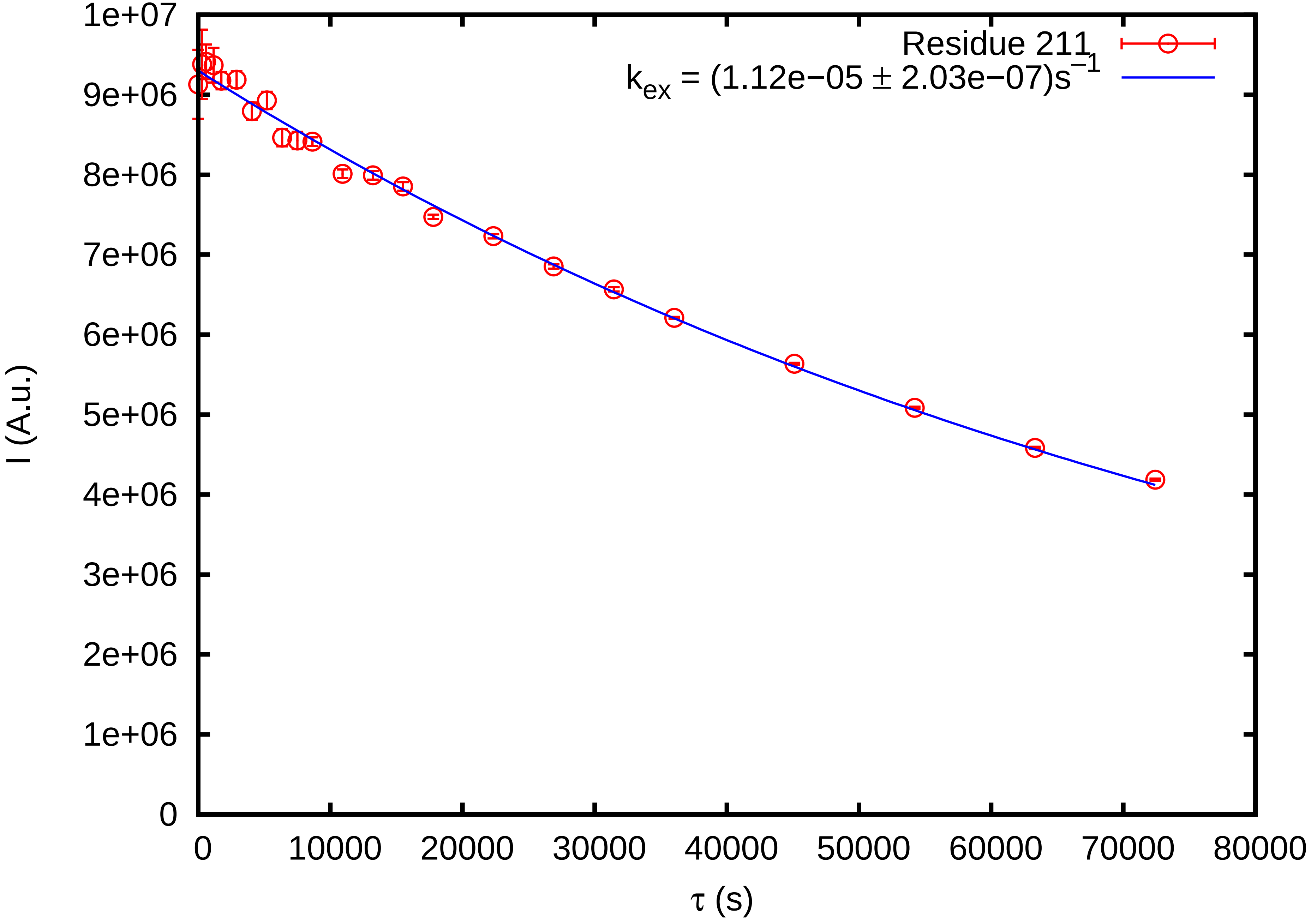

Residue 211
k_{ex} = (1.12e−05 ± 2.03e−07)s^{-1}
1e+07
9e+06
8e+06
7e+06
6e+06
5e+06
4e+06
3e+06
2e+06
1e+06
0
0
10000
20000
30000
40000
50000
60000
70000
80000
I (A.u.)
τ (s)

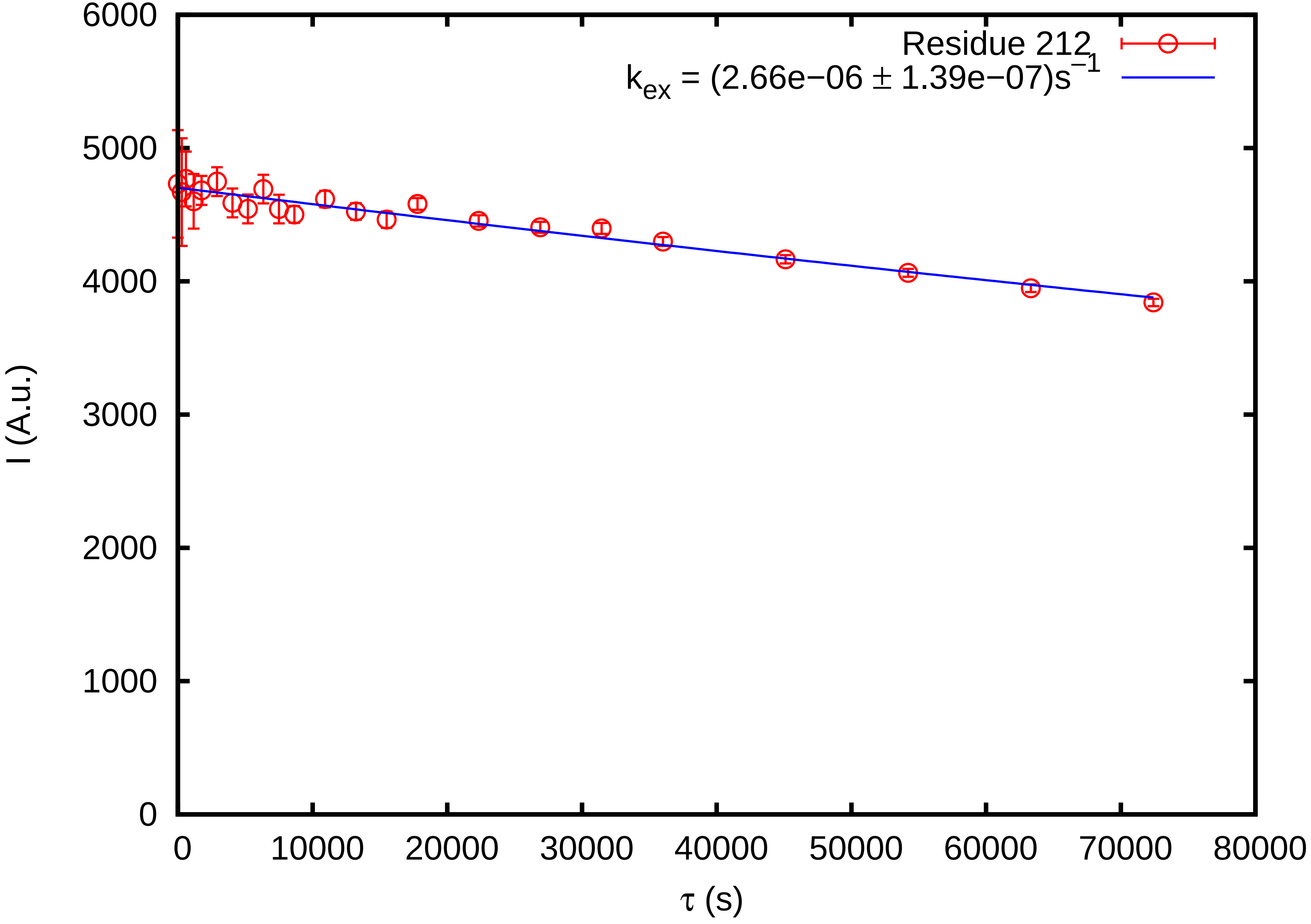

Residue 212
k_{ex} = (2.66e−06 ± 1.39e−07)s^{-1}
I (A.u.)
τ (s)
6000
5000
4000
3000
2000
1000
0
0
10000
20000
30000
40000
50000
60000
70000
80000

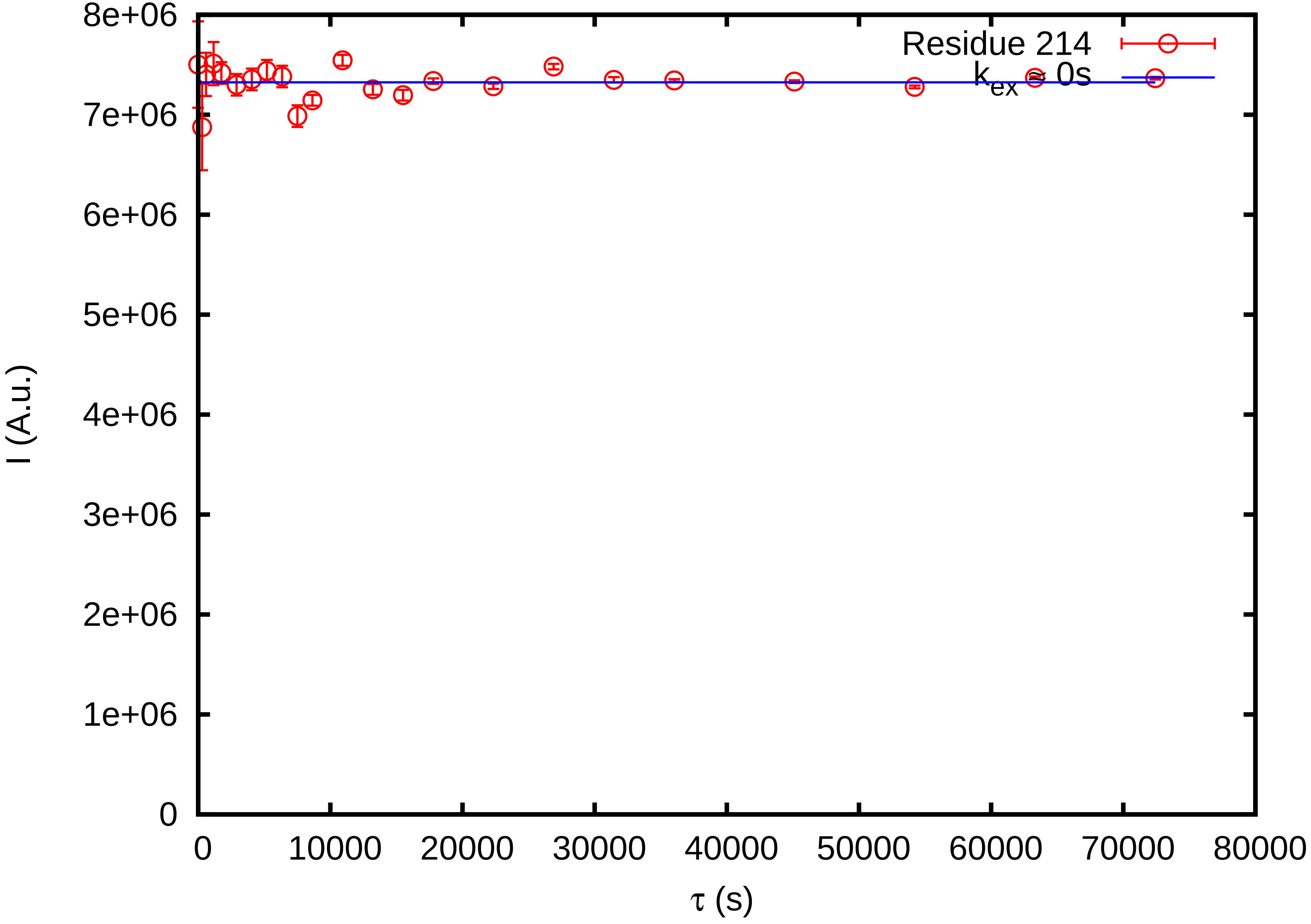

Residue 214
k_ex ≈ 0s
I (A.u.)
τ (s)
8e+06
7e+06
6e+06
5e+06
4e+06
3e+06
2e+06
1e+06
0
0
10000
20000
30000
40000
50000
60000
70000
80000

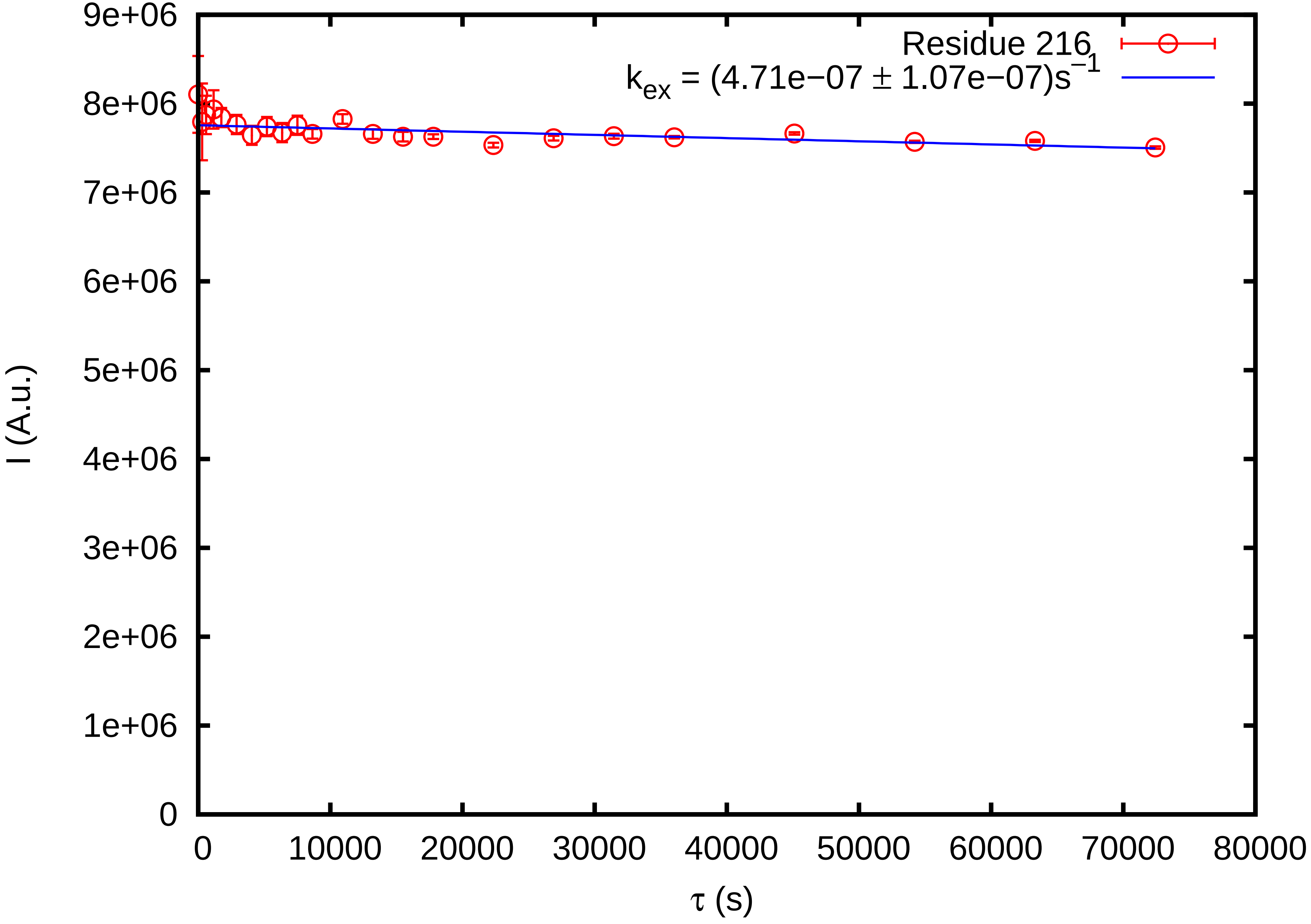

Residue 216
k_{ex} = (4.71e−07 ± 1.07e−07)s^{-1}
I (A.u.)
τ (s)
0
1e+06
2e+06
3e+06
4e+06
5e+06
6e+06
7e+06
8e+06
9e+06
0
10000
20000
30000
40000
50000
60000
70000
80000

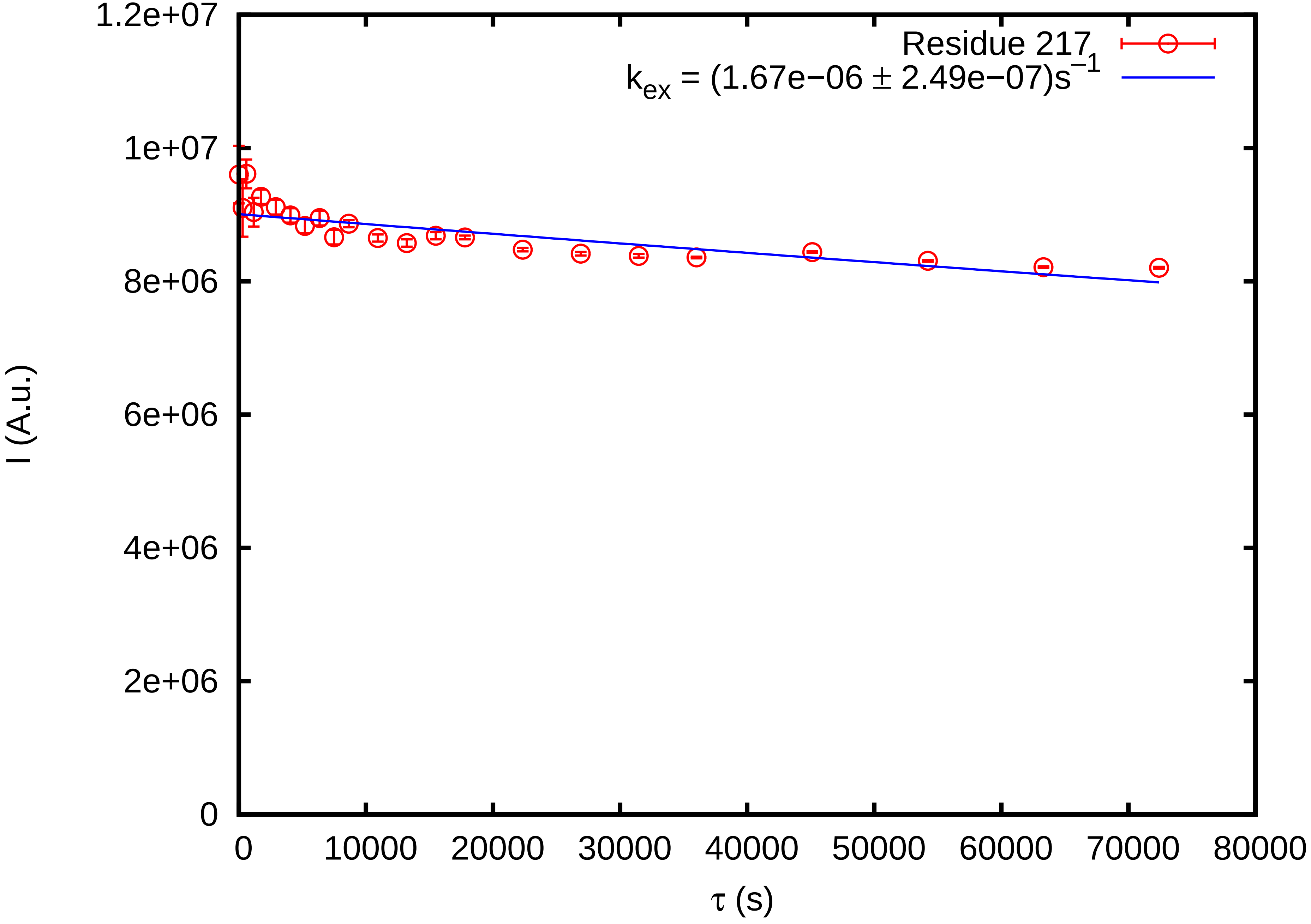

Residue 217
k_ex = (1.67e−06 ± 2.49e−07)s^−1
I (A.u.)
τ (s)
1.2e+07
1e+07
8e+06
6e+06
4e+06
2e+06
0
0
10000
20000
30000
40000
50000
60000
70000
80000
